# Prior Local Therapy and First-Line Apalutamide in Patients With Nonmetastatic Castration-Resistant Prostate Cancer: A Secondary Analysis of the SPARTAN Randomized Clinical Trial

**DOI:** 10.1001/jamanetworkopen.2024.39434

**Published:** 2024-10-15

**Authors:** Soumyajit Roy, Shawn Malone, Kevin Wing, Simon Chowdhury, Amar U. Kishan, Yilun Sun, Christopher J. D. Wallis, Osama Mohamad, Angela Y. Jia, Umang Swami, Nicholas G. Zaorsky, Scott C. Morgan, Michael Ong, Neeraj Agarwal, Daniel E. Spratt, Eric J. Small, Fred Saad

**Affiliations:** 1 Department of Radiation Oncology, Rush University Medical Center, Chicago, Illinois; 2 Usher Institute, University of Edinburgh, Edinburgh, United Kingdom; 3 Ottawa Hospital Cancer Centre, University of Ottawa, Ottawa, Ontario, Canada; 4 Public Health, School of Health and Wellbeing, University of Glasgow, Glasgow, United Kingdom; 5 Guy’s and St Thomas’ NHS Foundation Trust and Sarah Cannon Research Institute, London, United Kingdom; 6 University of California, Los Angeles; 7 University Hospitals–Seidman Cancer Center, Case Western Reserve University, Cleveland, Ohio; 8 Mount Sinai Hospital, University Hospital Network, University of Toronto, Toronto, Ontario, Canada; 9 MD Anderson Cancer Center, Houston, Texas; 10 Huntsman Cancer Institute, University of Utah, Salt Lake City; 11 Department of Medical Oncology, University of California, San Francisco; 12 Department of Surgery, Centre Hospitalier de l’Université de Montréal, Montréal, Quebec, Canada

## Abstract

**Importance:**

Preclinical studies suggest that exposure to prostate-directed local therapy (LT) may influence the efficacy of subsequent systemic therapy including androgen receptor pathway inhibitors. However, there is insufficient clinical evidence to support this premise in patients with nonmetastatic castrate-resistant prostate cancer (nmCRPC).

**Objective:**

To determine whether exposure to prior prostate-directed LT (radical prostatectomy [RP], radiation therapy [RT], or both) played any effect-modifying role in the treatment effect of apalutamide on metastasis-free survival (MFS) and overall survival (OS) in patients with nmCRPC.

**Design, Setting, and Participants:**

This post hoc secondary analysis used individual patient data from SPARTAN (Study of Apalutamide [ARN-509] in Men With Non-Metastatic Castration-Resistant Prostate Cancer), a phase 3, double-blinded, placebo-controlled randomized clinical trial conducted at 332 sites in 26 countries. Between October 14, 2013, and December 15, 2016, patients with nmCRPC and a prostate-specific antigen doubling time of 10 months or less were randomly assigned to apalutamide vs placebo; all patients received androgen deprivation therapy. The final data analysis was performed on December 31, 2023.

**Exposure:**

Prior prostate-directed LT.

**Main Outcomes and Measures:**

Separate Cox proportional hazards regression models were constructed for OS and MFS, which included prior LT, treatment group, and an interaction term, in addition to a minimally sufficient set of confounders. Adjusted hazard ratios (HRs) with 95% CIs for MFS and OS were determined for the apalutamide groups with or without prior LT.

**Results:**

Among the 1179 evaluable patients included in this analysis, 795 received prior LT and 384 did not. The median age of patients with and without prior LT was 70 (IQR, 45-90) years and 75 (IQR, 50-95) years, respectively. The median follow-up was 52.0 (IQR, 51.5-52.8) months. A differential treatment effect of apalutamide on MFS was observed between patients with and without prior LT (*P* for interaction = .009), with greater benefits for those with prior LT (adjusted HR, 0.22 [95% CI, 0.17-0.27]) compared with those without prior LT (adjusted HR, 0.35 [95% CI, 0.25-0.51]). However, there was insufficient evidence of a differential treatment effect on OS among subgroups stratified by exposure to prior LT (*P* for interaction = .23), with improved OS in the subgroup with prior LT (adjusted HR, 0.72 [95% CI, 0.57-0.92]) but no significant difference in OS in the subgroup without prior LT (adjusted HR, 0.92 [95% CI, 0.64-1.31]).

**Conclusions and Relevance:**

This post hoc analysis of the SPARTAN trial provides evidence of an interaction between prior LT and apalutamide in patients with nmCRPC, with a clinically significant and more favorable treatment effect from apalutamide on MFS among patients with prior LT. Further studies are needed to validate these findings.

**Trial Registration:**

ClinicalTrials.gov Identifier: NCT01946204

## Key Points

**Question:**

Does prior prostate-directed local therapy (LT) modify the response to subsequent androgen receptor pathway inhibitors (ARPIs) among patients with nonmetastatic castration-resistant prostate cancer (nmCRPC)?

**Findings:**

In this secondary analysis of a randomized clinical trial of 1179 men, a differential treatment effect of apalutamide on metastasis-free survival (MFS) was observed between patients with and without prior LT, with greater MFS benefit among those with prior LT. There was no difference in treatment effect on overall survival between the 2 subgroups.

**Meaning:**

Although these findings are hypothesis generating, they indicate a possible synergy between LT and ARPIs in patients with nmCRPC.

## Teaser

This secondary analysis of a randomized clinical trial examines the association of prior prostate-directed local therapy and first-line apalutamide with survival among patients with nonmetastatic castration-resistant prostate cancer (nmCRPC).

## Introduction

Approximately one-third of patients with localized prostate cancer eventually develop biochemical recurrence despite curative treatment.^1,2,3,4^ Overall, approximately 20% to 30% of patients receiving androgen deprivation therapy (ADT) for biochemical recurrence eventually develop resistance to ADT and develop castration-resistant prostate cancer (CRPC) within approximately 6 to 10 years.^5,6^ Although some patients with prostate cancer have distant metastases at the time of development of castration resistance, many do not demonstrate any signs of metastases on conventional imaging, which is referred to as *nonmetastatic CRPC* (nmCRPC). In the absence of a change of therapy, approximately 30% of these patients develop distant metastasis or die within a short span of time.^4^

Three large, phase 3, placebo-controlled randomized clinical trials provided evidence for use of an androgen receptor pathway inhibitor (ARPI) added to ADT to delay the development of metastases in patients with nmCRPC with a prostate-specific antigen doubling time (PSADT) of 10 months or less. These 3 studies—SPARTAN, PROSPER, and ARAMIS—tested apalutamide, enzalutamide, and darolutamide, respectively.^7,8,9^

In the SPARTAN trial, 1207 patients with nmCRPC with a PSADT of 10 months or less were randomly assigned (2:1) to receive apalutamide vs placebo in addition to ADT. The addition of apalutamide to ADT resulted in significantly improved metastasis-free survival (MFS) (hazard ratio [HR], 0.28 [95% CI, 0.23-0.35]) and overall survival (OS) (73.9 vs 59.9 months; HR, 0.78 [95% CI, 0.64-0.96]).^9,10^

Although the role of ARPIs in nmCRPC is well demonstrated, the effect of antecedent local therapy (LT) on outcomes and treatment response to ARPIs is less clear. Prior LT may be important in this context for several reasons. First, in vitro studies suggest that prostate radiation therapy (RT) can induce cellular changes such as neuroendocrine differentiation and loss of expression of androgen receptors (AR) and may result in resistance to AR-directed therapy.^11,12^ Furthermore, minimal residual disease after radical prostatectomy (RP) also can give rise to treatment-resistant clones through several potential mechanisms.^13,14,15,16,17,18^ These factors may potentially result in inferior response to subsequent systemic therapy when the cancer progresses to a nonmetastatic castration-resistant phase.

Clinical studies report conflicting findings with respect to the effect of prior prostate-directed LT. In a secondary analysis of the COU-AA-302 trial, there was no evidence of significant time-dependent effect modification by receipt of prior RT or RP.^19^ However, in an exploratory analysis of the TITAN trial, treatment with ADT plus apalutamide resulted in a relatively greater OS benefit for patients who received prior LT relative to those who did not.^20^

Thus, it remains unclear whether prior LT plays a role in subsequent response to first-line ARPI, specifically in patients with nmCRPC. Furthermore, it also remains unclear whether prior LT is independently associated with outcomes among patients with nmCRPC independent of subsequent treatment. We performed a post hoc secondary analysis of the SPARTAN trial to determine whether the response to first-line apalutamide among patients with nmCRPC varied based on receipt of prior LT for prostate cancer.

## Methods

This post hoc secondary analysis of the SPARTAN (A Study of Apalutamide [ARN-509] in Men With Non-Metastatic Castration-Resistant Prostate Cancer) trial was approved by the Usher Masters Research Ethics Group at the University of Edinburgh. The trial protocol is presented in Supplement 1. This analysis included patients in the SPARTAN trial database (at the Yale University Open Data Access Project) with available information on outcome, treatment regimen, potential effect modifiers, and additional confounders. Requirement for informed consent for this study was waived because publicly available data were used (per the Common Rule). This analysis followed the Strengthening the Reporting of Observational Studies in Epidemiology (STROBE) reporting guideline.

### Study Population and Design

The SPARTAN trial was conducted from October 14, 2013, to December 15, 2016, at 332 sites in 26 countries. Eligible patients were aged 18 years or older; had prostate adenocarcinoma that was castration resistant and nonmetastatic, determined by bone scans and computed tomography (CT) scans; and had a PSADT of 10 months or less during continuous ADT. Patients were randomly assigned (2:1) to receive apalutamide vs placebo in addition to ADT. Disease assessments, including CT scans and bone scans, were performed every 16 weeks and at additional time points if distant metastases were suspected. The primary end point of the SPARTAN trial was MFS, and OS was a secondary end point.

### Statistical Analysis

This study aimed to determine whether exposure to prior prostate-directed LT had a causal interaction with the treatment effect from ADT plus apalutamide (relative to ADT alone) on OS and MFS, separately. For this analysis, prior LT included RP, definitive prostate RT, or both. To analyze such causal interaction, confounding between the potential effect modifier and the outcome of interest was taken into consideration.^21^

Descriptive statistics were used to characterize the study cohort. Given previous literature on racial disparity in outcome of prostate cancer, data on race and ethnicity were collected for our study. We categorized race and ethnicity as Asian, Black, White, or other race or ethnicity (ie, including American Indian or Alaska Native, unreported, or other race or ethnicity; these racial and ethnic categories were combined due to their very small representation). To determine the effect-modifying role of prior LT on the treatment effect of ADT plus apalutamide on MFS, we constructed a multivariable Cox proportional hazards regression model with inclusion of randomized treatment regimen, prior LT, and an interaction term between the randomized treatment regimen and prior LT, in addition to a minimally sufficient list of confounders associated with both the potential effect modifier and the outcome of interest (eResults in Supplement 2). For confounders with a borderline association between the potential effect modifier and the hazard of the outcome of interest, we applied clinical assumptions to determine whether they were suitable for inclusion in the multivariable Cox proportional hazards regression model. The interaction between receipt of prior LT and randomized treatment regimen was also evaluated on an additive scale using the relative excess risk due to interaction (RERI).^22,23^ The RERI is calculated as the difference between the expected effect based on the sum of the separate effects of the 2 exposures and the observed effect in those with joint exposure. A RERI of 0 indicates no interaction, less than 0 indicates subadditivity, and greater than 0 indicates positive interaction or supraadditivity.^24^ A similar approach was taken to determine the effect-modifying role of prior LT on the treatment effect of ADT plus apalutamide on OS on both multiplicative and additive scales. As sensitivity analyses, we tested interaction for each pair of potential effect modifiers and time-to-event outcomes, in which we included all available variables from the trial database as confounders. For the Cox proportional hazards regression models, proportionality assumption was checked by visually inspecting the Schoenfeld residuals.^25^ A similar approach was taken to determine the effect modification by prior RP and prior RT, separately.

To determine the inverse probability of treatment weighting (IPTW)-based average treatment effect of exposure to 3 types of prior LT (RT alone, RP alone, or RP plus RT, respectively) on MFS and OS, we applied covariable balancing propensity score weighting to determine the propensity score and weights using a multinomial model with exposure to any LT exposures (no LT vs prior RP vs prior RT vs prior RP plus RT) as a dependent variable.^26^ Subsequently, a weighted Cox proportional hazards regression model was applied to determine the association of prior RP, prior RT, or prior RP plus RT (relative to no prior LT) with MFS and OS (expressed in terms of hazard ratios [HRs] with 95% CIs), respectively.^27,28,29^ *P* < .05 (2-sided) was considered statistically significant.

All statistical analyses were performed using R, version 4.2.2 (R Project for Statistical Computing). The final data analysis was performed on December 31, 2023.

## Results

Of the 1207 SPARTAN participants, 1179 (97.7%) had information available in the trial database on outcome, treatment regimen, potential effect modifiers, and additional confounders and were included in this study. The ADT plus apalutamide group comprised 789 patients, and the ADT plus placebo group comprised 390 patients. Overall, in the available database, 795 patients (67.4%) had received prior LT and 384 (32.6%) had no exposure to prior LT. The median age of patients with and without prior LT was 70 (IQR, 45-90) years and 75 (IQR, 50-95) years, respectively. A total of 138 patients (11.7%) were Asian, 64 (5.4%) were Black, 783 (66.4%) were White, and 194 (16.5%) were of other race or ethnicity. Among those with prior LT, 526 received ADT plus apalutamide and 269 received ADT alone (eFigure 1 in Supplement 2). Patients who received prior LT were younger and had a longer time between their diagnosis of prostate cancer and randomization. Among patients with prior exposure to LT, a relatively higher proportion had lower-risk disease. Nodal stage and exposure to prior ADT were well balanced between patients with and without prior LT (Table 1). Among the 795 patients with prior LT, 399 (50.2%) received RT alone, 283 (35.6%) received RP and RT, and 113 (14.2%) received RP alone. Baseline characteristics of patients with and without exposure to prior RT and prior RP are summarized in eTables 1 and 2 in Supplement 2. The median follow-up of the study cohort was 52.0 (IQR, 51.5-52.8) months.

**Table 1.** Baseline Patient Characteristics by Exposure to Prior Prostate-Directed LT and Randomized Treatment Regimen^a^

Characteristic	No prior LT (n = 384)		Prior LT (n = 795)		*P* value
	ADT alone (n = 121)^b^	ADT plus apalutamide (n = 263)	ADT alone (n = 269)^b^	ADT plus apalutamide (n = 526)	
Age at randomization, y					
Mean (SD)	74 (8.6)	74 (8.6)	71 (7.6)	71 (8.0)	<.001
Median (range)	75 (50-95)	75 (50-95)	70 (50-90)	70 (45-90)	
PSADT, mo					
Mean (SD)	5.0 (2.3)	5.0 (2.3)	4.6 (2.2)	4.6 (2.3)	.03
Median (range)	4.5 (1.5-9.9)	4.7 (1.1-9.7)	4.4 (0.7-10.0)	4.1 (0.8-10.0)	
Time since diagnosis, y					
Mean (SD)	6.4 (4.3)	5.8 (3.8)	9.3 (5.1)	9.9 (5.2)	<.001
Median (range)	5.0 (1.0-25.0)	5.0 (0.5-19.0)	9.0 (1.0-26.0)	9.0 (1.0-30.0)	
Race and ethnicity					
Asian	18 (14.9)	50 (19.0)	29 (10.8)	41 (7.8)	<.001
Black	7 (5.8)	8 (3.0)	11 (4.1)	38 (7.2)	
White	78 (64.5)	165 (62.7)	191 (71.0)	349 (66.4)	
Other^c^	18 (14.9)	40 (15.2)	38 (14.1)	98 (18.6)	
Nodal stage					
N0	103 (85.0)	213 (81.0)	223 (82.9)	444 (84.4)	.60
N1	18 (15.0)	50 (19.0)	46 (17.1)	82 (15.6)	
Prior bone-sparing agent					
No	111 (91.7)	240 (91.3)	240 (89.2)	470 (89.4)	.70
Yes	10 (8.3)	23 (8.7)	29 (10.8)	56 (10.6)	
Primary Gleason score					
1-3	37 (30.6)	81 (30.8)	104 (38.7)	244 (46.4)	.001
4-5	77 (63.6)	169 (64.3)	155 (57.6)	263 (50.0)	
Unassigned	7 (5.8)	13 (4.9)	10 (3.7)	19 (3.6)	
Secondary Gleason score					
1-3	40 (33.1)	70 (26.6)	103 (38.3)	194 (36.9)	.08
4-5	74 (61.2)	179 (68.1)	156 (58.0)	310 (59.0)	
Unassigned	7 (5.8)	14 (5.3)	10 (3.7)	22 (4.1)	
Tumor stage group					
T1-T2	57 (47.1)	111 (42.2)	128 (47.6)	294 (55.9)	<.001
T3-T4	51 (42.1)	120 (45.6)	126 (46.8)	207 (39.4)	
TX	13 (10.7)	32 (12.2)	15 (5.6)	25 (4.8)	
Prior ADT					
No	43 (35.5)	78 (29.7)	78 (29.0)	132 (25.1)	.11
Yes	78 (64.5)	185 (70.3)	191 (71.0)	392 (74.9)	

Abbreviations: ADT, androgen deprivation therapy; LT, local therapy; PSADT, prostate-specific antigen doubling time.

^a^ Unless indicated otherwise, values are presented as No. (%) of patients.

^b^ Placebo plus ADT.

^c^ Includes American Indian or Alaska Native (n = 4), unreported (n = 188), or other race or ethnicity (n = 2).

### Metastasis-Free Survival

We observed a difference in the adjusted association between ADT plus apalutamide and MFS among subgroups stratified by exposure to prior LT (*P* for interaction = .009). Treatment with ADT plus apalutamide was associated with a 78.0% reduction in risk for distant metastasis or death among patients with prior LT (adjusted HR, 0.22 [95% CI, 0.17-0.27]) compared with a 65.0% reduction among those without prior LT (adjusted HR, 0.35 [95% CI, 0.25-0.51]) (Table 2). Similarly, on an additive scale, we observed evidence of increased relative excess risk of metastasis or death among patients with joint exposure to prior LT and ADT alone (RERI, 2.10 [95% CI, 1.00-3.21]).

**Table 2.** Survival Outcomes Among Patients Receiving Apalutamide Plus ADT vs ADT Alone, Stratified by Receipt of Prior LT

Outcome	ADT alone, No. of events/patients^a^	ADT plus apalutamide, No. of events/patients	Adjusted HR (95% CI)	*P* value for interaction
**Metastasis-free survival**					
With prior LT	178/269	160/526	0.22 (0.17-0.27)	.009
Without prior LT	59/121	72/263	0.35 (0.25-0.51)	
**Overall survival**					
With prior LT	105/269	172/526	0.72 (0.57-0.92)	.23
Without prior LT	45/121	98/263	0.92 (0.64-1.31)	

Abbreviations: ADT, androgen deprivation therapy; HR, hazard ratio; LT, local therapy.

^a^ Placebo plus ADT.

We then considered effect modification by specific LT modalities. We observed a difference in the adjusted association between ADT plus apalutamide and MFS among subgroups stratified by prior RT exposure, although this did not reach the conventional level of significance (*P* for interaction = .09). Treatment with ADT plus apalutamide was associated with improved MFS in both subgroups with vs without prior RT (adjusted HR, 0.22 [95% CI, 0.17-0.28] vs 0.30 [95% CI, 0.22-0.40]) (Table 3). On the additive scale, there was an increased relative risk of distant metastasis or death in patients with ADT alone but without prior RT (RERI, 1.65 [95% CI, 0.52-2.79]).

**Table 3.** Survival Outcomes Among Patients Receiving Apalutamide Plus ADT vs ADT Alone, Stratified by Receipt of Prior RP and Prior RT

Outcome	ADT alone, No. of events/patients^a^	ADT plus apalutamide, No. of events/patients	Adjusted HR (95% CI)	*P* value for interaction
**Metastasis-free survival**				
Prior RP				
With	87/127	78/269	0.18 (0.13-0.24)	.005
Without	150/263	154/520	0.29 (0.23-0.37)	
Prior RT				
With	152/230	142/452	0.22 (0.17-0.28)	.09
Without	85/160	90/337	0.30 (0.22-0.40)	
**Overall survival**				
Prior RP				
With	47/127	72/269	0.61 (0.42-0.89)	.11
Without	103/263	198/520	0.87 (0.69-1.11)	
Prior RT				
With	91/230	154/452	0.77 (0.59-0.99)	.66
Without	59/160	116/337	0.84 (0.61-1.15)	

Abbreviations: ADT, androgen deprivation therapy; HR, hazard ratio; RP, radical prostatectomy; RT, radiation therapy.

^a^ Placebo plus ADT.

We observed a difference in the adjusted association of ADT plus apalutamide with MFS between patient groups stratified by exposure to prior RP (*P* for interaction = .005). Among patients with prior RP, treatment with ADT plus apalutamide had a relatively more favorable effect on MFS (adjusted HR, 0.18 [95% CI, 0.13-0.24]) compared with a treatment effect of ADT plus apalutamide among those without prior RP (adjusted HR, 0.29 [95% CI, 0.23-0.37]) (Table 3). There was a subadditive interaction between prior RP and the randomized treatment regimen (RERI in patients with ADT alone but without prior RP, −2.03 [95% CI, −3.57 to −0.48]).

For each interaction analysis with respect to MFS, a fully adjusted model was constructed as a sensitivity analysis. The findings were consistent with the model including the minimal set of confounders (eResults in Supplement 2).

On IPTW-based Kaplan-Meier analysis, the median MFS for patients with vs without prior LT was 29.2 (IQR, 11.0 to not reached [NR]) months vs 25.8 (IQR, 7.5 to NR) months (Figure A), respectively, with no significant evidence of a between-group difference on the weighted log-rank test (*P* = .72). The balance of covariables and propensity score distribution is illustrated in eFigures 2 and 3 in Supplement 2. Relative to patients without prior LT, we did not find sufficient evidence that exposure to prior RP alone, prior RT alone, or prior RP plus RT was associated with MFS (Table 4). The balance of covariables is illustrated in eFigure 4 in Supplement 2.

**Figure.**
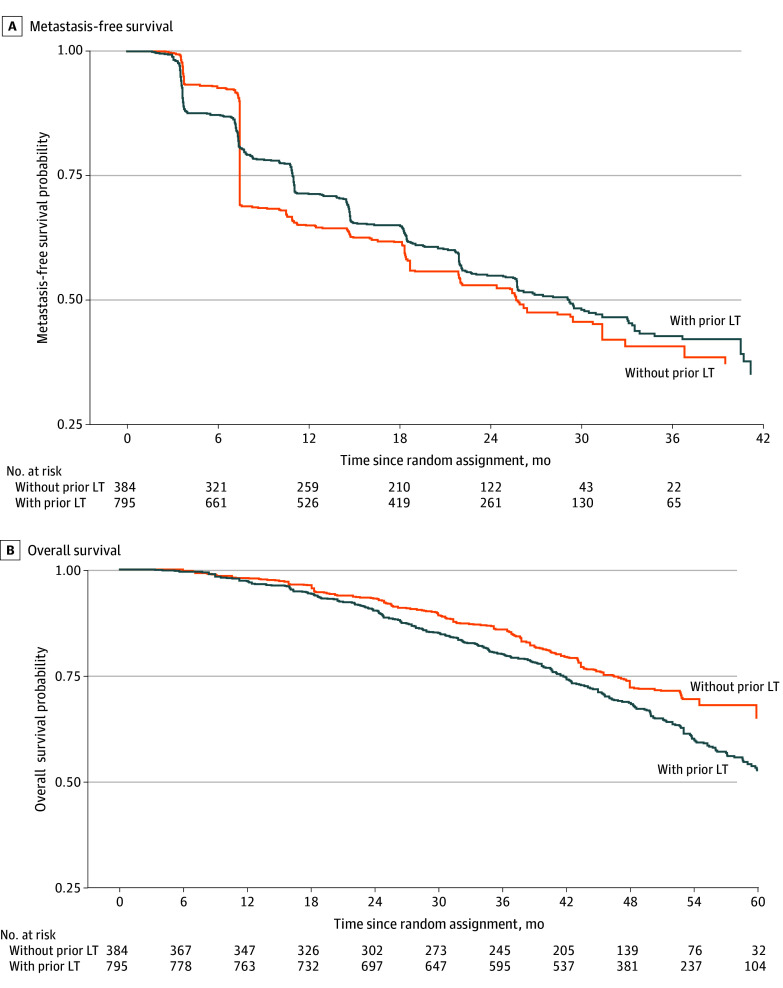
Inverse Probability of Treatment Weighting–Based Kaplan-Meier Survival Curves for Metastasis-Free Survival and Overall Survival for Patients With and Without Prior Prostate-Directed Local Therapy (LT) A, Metastasis-free survival. B, Overall survival.

**Table 4.** Association of Prior LT Modalities With MFS and OS Relative to Lack of Prior LT

LT modality	MFS		OS	
	HR (95% CI)	*P* value	HR (95% CI)	*P* value
Prior RP vs no prior LT	1.02 (0.67-1.53)	.94	0.71 (0.46-1.08)	.10
Prior RP plus RT vs no prior LT	1.13 (0.81-1.58)	.48	0.80 (0.57-1.13)	.21
Prior RT vs no prior LT	1.29 (0.95-1.73)	.10	1.18 (0.88-1.56)	.27

Abbreviations: HR, hazard ratio; LT, local therapy; MFS, metastasis-free survival; OS, overall survival; RP, radical prostatectomy; RT, radiation therapy.

### Overall Survival

We did not observe a significant difference in the adjusted association between ADT plus apalutamide and OS among subgroups stratified by exposure to prior LT (*P* for interaction = .23) (Table 2). Treatment with ADT plus apalutamide was associated with a 28.0% reduction in the risk of death among patients with prior LT (adjusted HR, 0.72 [95% CI, 0.57-0.92]), but there was no significant difference in risk of death among those without prior LT (adjusted HR, 0.92 [95% CI, 0.64-1.31]). There was insufficient evidence of an interaction between prior LT and the randomized treatment regimen on the additive scale (RERI with ADT alone but without prior RT, −0.30 [95% CI, −0.88 to 0.28]). Similarly, we did not find sufficient evidence of a differential association of ADT plus apalutamide with OS among patients stratified by receipt of prior RT (*P* for interaction = .66) or prior RP (*P* for interaction = .11) (Table 3). Treatment with ADT plus apalutamide was associated with improved OS among patients with prior RT (adjusted HR, 0.77 [95% CI, 0.59-0.99]), but there was no significant difference in OS among patients without prior RT (adjusted HR, 0.84 [95% CI, 0.61-1.15]). There was insufficient evidence of an interaction between prior RT and randomized treatment regimen on the additive scale (RERI with ADT alone but without prior RT, −0.11 [95% CI, −0.59 to 0.37]). Similarly, treatment with ADT plus apalutamide was associated with superior OS among patients with prior RP (adjusted HR, 0.61 [95% CI, 0.42-0.89]), but there was no significant difference in OS among patients without prior RP (adjusted HR, 0.87 [95% CI, 0.69-1.11]). There was insufficient evidence of an interaction between prior RP and randomized treatment regimen on the additive scale (RERI with ADT alone but without prior RT, −0.40 [95% CI, −1.11 to 0.30]).

For each interaction analysis with respect to OS, a fully adjusted model was constructed as a sensitivity analysis. The findings were consistent with the model including the minimal set of confounders (eResults in Supplement 2).

The median OS was NR for patients without prior LT, whereas the median OS was 62.0 (IQR, 41.5 to NR) months for patients with prior LT (Figure B). On the weighted log-rank test, there was no statistically significant difference in adjusted OS among patients with and without prior LT (*P* = .29). Similarly, relative to no prior LT, there was insufficient evidence of an association of prior exposure to RP, RP plus RT, or RT alone with risk of death, respectively (Table 4).

## Discussion

In this secondary analysis of the SPARTAN trial, we noted greater benefit from ADT plus apalutamide in terms of MFS among patients who received prior prostate-directed LT compared with those without prior definitive LT. When stratified by treatment modality, this effect was statistically significant among patients who underwent prior RP and not prior RT, although the clinical magnitude of benefit was comparable between the 2 groups. We did not find sufficient evidence of effect modification or additive interaction between prior LT (or type of prior LT) and randomized treatment regimen for OS, although apalutamide was associated with a 28.0% reduction in risk of death among patients with prior LT relative to an 8.0% reduction among those without prior LT. This difference in the effect of apalutamide on OS may still be clinically relevant. There was insufficient evidence of any adjusted association of prior RT, RP, or RT plus RP (relative to prior LT) with risk of distant metastasis or death, respectively.

The interaction with exposure to prior RT resulted in a more favorable treatment effect of ADT plus apalutamide on MFS, but this interaction did not reach the conventional level of statistical significance. These findings suggest a complex interplay between RT exposure and type of subsequent systemic therapy. Although preclinical studies raise a possibility of neuroendocrine differentiation or a treatment-resistant phenotype driven by exposure to prior RT,^30,31^ we may hypothesize that such lineage plasticity has a reduced effect on treatment efficacy when an ARPI is added to ADT, because ARPIs can mediate proapoptotic modulation in these neuroendocrine cells and thus mitigate treatment resistance.^32^

Taken together with a contemporary secondary analysis of the TITAN trial,^20^ our findings suggest that there may be potential synergy between LT and apalutamide. Patients who received prior LT may have had a reduced burden of occult local or pelvic disease, which is seen in approximately 30% to 40% of patients with nmCRPC when imaged with prostate-specific membrane antigen positron emission tomography.^33,34^ This decreased burden, in turn, potentiated the effect of apalutamide on MFS. The lack of effect modification for OS may potentially reflect the effect of crossover to ADT plus apalutamide for patients initially treated with ADT alone after distant metastasis, thus diluting any substantive differential treatment effect. Furthermore, exposure to prior LT may be a surrogate for better access to treatment, lower-risk disease, superior overall performance status, and functional reserve.^35,36^ If this were the case, we may hypothesize that patients with exposure to prior LT were at equally less risk of cancer-related and non–cancer-related deaths in both treatment groups, further contributing to the lack of difference in treatment effect on OS. Our findings are consistent with a secondary analysis of the ARAMIS trial, in which estimates of OS among darolutamide-treated patients were consistent across all subgroups treated with different types of prior prostate-directed LT.^37^

### Limitations

Our study has several limitations, including those inherent to any post hoc secondary analysis of a clinical trial. These limitations include susceptibility to selection bias, false discoveries with no plausible underlying biological mechanism, and limited power to detect statistically significant between-subgroup differences. Given that this is a post hoc secondary analysis of a historical observational cohort, the findings may be subject to misclassification bias related to the effect modifiers. Residual confounding could also not be ruled out. Interpretation of the findings related to MFS requires caution due to interval censoring. The external validity and generalizability of these findings are limited due to small numbers of racial and ethnic minority individuals and those with poor performance status in this clinical trial. Overall, without a clear understanding of the reasons for not receiving prior prostate-directed LT, it remains challenging to derive any conclusions regarding the differences in outcomes between the 2 subgroups. Therefore, the findings are, at best, hypothesis generating and warrant further validation.

## Conclusions

In this secondary post hoc analysis of the SPARTAN randomized clinical trial, we observed an interaction between exposure to prior prostate-directed LT and a treatment effect from a combination of ADT plus apalutamide on MFS. Treatment with ADT plus apalutamide conferred improved MFS in both subgroups, but the effect was significantly more favorable among patients with prior LT. That said, there was no evidence of any difference in treatment effect from ADT plus apalutamide on OS among subgroups stratified by exposure to prior LT. Of note, the treatment effect from apalutamide plus ADT was numerically more favorable among patients exposed to prior LT. Although these findings require further validation, they are reassuring and provide evidence of the efficacy of first-line ADT plus an ARPI across subgroups of patients with nmCRPC who received different types of prior LT.

## Notes

Supplement 1.
Trial Protocol

Supplement 2. **eFigure 1.** CONSORT Diagram **eTable 1.** Baseline Characteristics by Exposure to Prior Radiation Therapy and Randomized Treatment Regimen **eTable 2.** Baseline Characteristics by Exposure to Prior Radical Prostatectomy and Randomized Treatment Regimen
eResults
**eFigure 2.** Love Plot Summarizing the Balance of Covariables Between the Unadjusted and Weighted Dataset for Prior Local Therapy **eFigure 3.** Histogram Summarizing the Distribution of Propensity Scores (Left Panel) and Weights (Right Panel) Among Patients With and Without Prior Prostate-Directed Local Therapy **eFigure 4.** Love Plot Summarizing the Balance of Covariables Between the Unadjusted and Weighted Dataset for Prior Local Therapy (LT) Modalities (Prior Radical Prostatectomy [RP], Prior Radiation Therapy [RT], or Prior RP Plus RT Compared to Prior LT)

Supplement 3.
Data Sharing Statement
