# Supplementary material for: Prior Local Therapy and First-Line Apalutamide in Patients With Nonmetastatic Castration-Resistant Prostate Cancer: A Secondary Analysis of the SPARTAN Randomized Clinical Trial
Source: JAMA Netw Open. 2024 Oct 15;7(10):e2439434. doi: 10.1001/jamanetworkopen.2024.39434 (PMC11581638; doi:10.1001/jamanetworkopen.2024.39434)
Supplement: Supplement 1. — Trial Protocol [file jamanetwopen-e2439434-s001.pdf]

Protocol

This trial protocol has been provided by the authors to give readers additional information about their work.

Protocol for: Smith MR, Saad F, Chowdhury S, et al. Apalutamide treatment and metastasis-free survival in prostate cancer. N Engl J Med 2018;378:1408-18. DOI: 10.1056/NEJMoa1715546

The supplement contains the following items:

1. Original protocol, final protocol, and summary of changes (included in the final protocol).
2. Original statistical analysis plan, final statistical analysis plan, and summary of changes. (Note: US approaches used in the manuscript)

Clinical Study Protocol

SPARTAN
(**S**elective **P**rostate **AR** **T**argeting with **ARN-509)**

A Multicenter, Randomized, Double-Blind, Placebo-Controlled, Phase III Study of ARN-509 in Men with Non-Metastatic (M0) Castration-Resistant Prostate Cancer

Protocol Number:	ARN-509-003
Version Date:	Version 1.0 (05 November 2012)
Investigational Product:	ARN-509
IND Number:	104676
EudraCT Number:	2012-004322-24
Development Phase:	3
Sponsor:	Aragon Pharmaceuticals<br>12780 El Camino Real, Suite 301<br>San Diego, CA 92130

The confidential information in the following document is provided to you as an investigator, potential investigator, or consultant, for review by you, your staff, and appropriate ethical review committee. By accepting this document, you agree that the information contained herein will not be disclosed to others without written authority from Aragon Pharmaceuticals, Inc., except to the extent necessary to obtain approval of this protocol by an ethical review committee.

SPONSOR APPROVALS

Protocol: ARN-509-003, Version 1.0 (05 November 2012)

Protocol Title: SPARTAN: A Multicenter, Randomized, Double-Blind, Placebo-Controlled, Phase III Study of ARN-509 in Men with Non-Metastatic (M0) Castration-Resistant Prostate Cancer

[redacted]

Isan Chen, [redacted]
Chief Medical Officer

09 NOV 2012
Date

[redacted]

Edna [redacted]
Senior Director, Clinical Development

09 Nov 2012
Date

PROTOCOL AGREEMENT

I confirm that I have read this protocol. I will comply with the protocol and the principles of Good Clinical Practice (GCP), as described in the United States Code of Federal Regulation (CFR) 21 Parts 11, 50, 54, 56, and 312 and the appropriate International Conference on Harmonisation guidance documents.

Protocol: ARN-509-003, Version 1.0 (05 November 2012)

Protocol Title: SPARTAN: A Multicenter, Randomized, Double-Blind, Placebo-Controlled, Phase III Study of ARN-509 in Men with Non-Metastatic (M0) Castration-Resistant Prostate Cancer

____ ____________________

Investigator Signature *Date*

____

Print Name and Title

Site # ___________

Site Name ______________________________________

PROTOCOL SYNOPSIS

**Title**	A Multicenter, Randomized, Double-Blind, Placebo-Controlled, Phase III Study of ARN-509 in Men with Non-Metastatic (M0) Castration-Resistant Prostate Cancer
**Sponsor**	Aragon Pharmaceuticals
**Development Phase**	3
**Number of Sites**	Approximately 250
**Rationale**	Prostate cancer is the second most frequently diagnosed cancer and the sixth leading cause of cancer death in males, accounting for 14% (903,500) of the total new cancer cases and 6% (258,400) of the total cancer deaths in males worldwide. The course of prostate cancer from diagnosis to death is best categorized as a series of clinical stages based on the extent of disease, hormonal status, and absence or presence of detectable metastases: localized disease, rising levels of prostate-specific antigen (PSA) after radiation therapy or surgery with no detectable metastases, and clinical metastases in the non-castrate or castrate stage. Although surgery, radiation, or a combination of both can be curative for patients with localized disease, a significant proportion of these patients have recurrent disease as evidenced by a rising level of PSA, which can lead to the development of metastases, especially in the high risk group – a transition to the lethal stage of the disease.<br><br>Androgen depletion is the standard treatment with a generally predictable outcome: decline in PSA, a period of stability in which the tumor does not proliferate, followed by rising PSA and regrowth as castration-resistant disease. Molecular profiling studies of castration-resistance prostate cancers commonly show increased androgen receptor (AR) expression, which can occur through AR gene amplification or other mechanisms.<br><br>ARN-509 is a second-generation anti-androgen that binds directly to the ligand-binding domain of AR, impairing nuclear translocation and DNA binding. ARN-509 binds AR with [redacted] greater affinity than bicalutamide, and induces partial or complete tumor regression in both castration-sensitive and castration-resistant human prostate cancer xenograft models.<br><br>A Phase I/II study (Protocol ARN-509-001) was designed to assess the safety, pharmacokinetics, and anti-tumor activity of ARN-509 in men with CRPC. The Phase I portion of the study has been completed and the Phase II is currently ongoing. In the Phase II, a total of 97 patients were enrolled across 3 different patient population subsets: high risk NM-CRPC, treatment-naïve metastatic

	CRPC, and metastatic CRPC after failure with abiraterone acetate.<br>In the NM-CRPC subset (n = 47), the most frequent, treatment-related, adverse events observed in more than 5% of the patients as of 20 August 2012 are fatigue (30%), diarrhea (28%), nausea (17%), rash (13%), abdominal pain (11%), flatulence (9%), abdominal discomfort (6%), constipation (6%), and dysgeusia (6%). Four Grade 3 events (diarrhea, hypertension, and 2 cases of rash) have been reported and 3 (6%) patients have discontinued the study due to adverse event (rash), consent withdrawal, and compliance issues, respectively. To date, the 12-week PSA response (≥ 50% decline from baseline) is 91%.
**Study Design**	This is a randomized (2:1), multicenter, double-blind, placebo-controlled, Phase III clinical trial evaluating the efficacy and safety of ARN-509 (treatment arm A) versus placebo (treatment arm B) in men with high risk (M0) NM-CRPC, defined as PSA Doubling Time (PSADT) ≤ 10 months.<br>Patients will be stratified based on:<br>▪ PSADT: > 6 months vs. ≤ 6 months<br>▪ Bone-sparing agent use: Yes vs. No<br>▪ Loco-regional disease: N0 vs. N1<br>Patients will be followed for safety and efficacy as per the schedule of activities and will remain on study treatment until documented radiographic progression (development of distant metastases as assessed by blinded independent central review) or the development of unacceptable toxicity.<br>Patients discontinuing treatment due to documented radiographic progression will enter the survival follow-up period, where they will be followed for the development of symptomatic progression and initiation of subsequent anti-cancer therapies (in particular, cytotoxic chemotherapy) every 4 months until death, loss of follow-up, or withdrawal of consent, whichever comes first.<br>Patients discontinuing treatment prior to documented radiographic progression will also enter the survival follow-up period where they will continue to have scheduled disease assessments every 4 months until documented radiographic progression, and will be followed for the development of symptomatic progression and initiation of subsequent anti-cancer therapies (in particular, cytotoxic chemotherapy) every 4 months until death, loss of follow-up, or withdrawal of consent, whichever comes first.

**Primary Objective**	To demonstrate superiority in the metastasis-free survival (MFS) of men with high risk NM-CRPC treated with ARN-509 versus placebo
**Key Secondary Objective**	To compare the overall survival (OS) of men with high risk NM-CRPC treated with ARN-509 versus placebo
**Other Secondary Objectives**	▪ To compare the time to symptomatic progression in men with high risk NM-CRPC treated with ARN-509 versus placebo<br>▪ To compare the time to initiation of cytotoxic chemotherapy in men with high risk NM-CRPC treated with ARN-509 versus placebo<br>▪ To compare the radiographic progression-free survival (PFS) of men with high risk NM-CRPC treated with ARN-509 versus placebo<br>▪ To compare the time to metastasis (TTM) in men with high risk NM-CRPC treated with ARN-509 versus placebo<br>▪ To compare patient reported outcomes (PROs) of health-related quality of life and prostate cancer-specific symptoms in men with high risk NM-CRPC treated with ARN-509 versus placebo<br>▪ To evaluate the safety and tolerability of ARN-509<br>▪ To evaluate the population pharmacokinetics of ARN-509<br>▪ To evaluate the effect of ARN-509 on ventricular repolarization in a subset of patients from selected clinical sites [Appendix 8]
**Number of Patients**	1200 (ARN-509: 800; placebo: 400)
**Enrollment Criteria**	**Inclusion Criteria**<br>1. Histologically or cytologically confirmed adenocarcinoma of the prostate without neuroendocrine differentiation or small cell features, with high risk for development of metastases, defined as PSADT ≤ 10 months<br>2. Castration-resistant prostate cancer demonstrated during continuous androgen deprivation therapy (ADT)/post orchiectomy, defined as 3 consecutive rises of PSA, 1 week apart, resulting in two 50% increases over the nadir, with the last PSA > 2 ng/mL<br>3. Maintain castrate levels of testosterone (< 50 ng/dL [1.72 nmol/L]) within 4 weeks prior to randomization and throughout the study<br>4. Patients currently receiving bone loss prevention treatment with bone-sparing agents (e.g., bisphosphonates, denosumab

	[Prolia®]) must be on stable doses for at least 4 weeks prior to randomization<br>5. Patients who received a first generation anti-androgen (e.g., bicalutamide, flutamide, nilutamide) as part of an initial combined androgen blockade therapy or as second-line hormonal therapy must show continuing disease (PSA) progression off the anti-androgen for at least 4 weeks prior to randomization<br>6. At least 4 weeks must have elapsed from the use of 5-α reductase inhibitors (e.g., dutasteride, finasteride, aminoglutethamide), estrogens, and any other anti-cancer therapy prior to randomization, including chemotherapy given in the adjuvant/neoadjuvant setting (e.g., clinical trial)<br>7. At least 4 weeks must have elapsed from major surgery or radiation therapy prior to randomization<br>8. Age ≥ 18 years<br>9. Eastern Cooperative Oncology Group (ECOG) Performance Status 0 or 1<br>10. Resolution of all acute toxic effects of prior therapy or surgical procedure to Grade ≤ 1 or baseline prior to randomization<br>11. Adequate organ function as defined by the following criteria:<br>▪ Serum aspartate transaminase (AST; serum glutamic oxaloacetic transaminase [SGOT]) and serum alanine transaminase (ALT; serum glutamic pyruvic transaminase [SGPT]) ≤ 2.5 x upper limit of normal (ULN)<br>▪ Total serum bilirubin ≤ 1.5 x ULN<br>▪ Serum creatinine ≤ 2 x ULN<br>▪ Absolute neutrophil count (ANC) ≥ 1500/μL<br>▪ Platelets ≥ 100,000/μL<br>▪ Hemoglobin ≥ 9.0 g/dL<br>o Administration of growth factors or blood transfusions will not be allowed within 4 weeks of the hematology labs required to confirm eligibility<br>12. Signed and dated informed consent document indicating that the patient (or legally acceptable representative) has been informed of all pertinent aspects of the trial prior to randomization<br>13. Willingness and ability to comply with scheduled visits, treatment plans, laboratory and radiographic assessments, and other study procedures, including ability to swallow large

capsules, the completion of patient reported outcomes questionnaires and long-term follow-up visits

Exclusion Criteria

1. Presence of distant metastases, including CNS and vertebral or meningeal involvement. **Exception**: pelvic lymph nodes < 2 cm in short axis (N1) located below the iliac bifurcation are allowed
2. Symptomatic loco-regional disease requiring medical intervention, such as moderate or severe urinary obstruction or hydronephrosis due to primary tumor (e.g., tumor obstruction of bladder trigone)
3. Prior treatment with second generation anti-androgens (e.g., enzalutamide)
4. Prior treatment with CYP17 inhibitors (e.g., abiraterone, orteronel, galeterone, ketoconazole)
5. Prior treatment with radiopharmaceutical agents (e.g., Strontium-89), immunotherapy (e.g., sipuleucel-T), or any other investigational agent for NM-CRPC (e.g., denosumab [Xgeva®])
6. Prior chemotherapy, except if administered in the adjuvant/neoadjuvant setting
7. History of seizure or condition that may pre-dispose to seizure (e.g., stroke within 1 year prior to randomization, brain arteriovenous malformation, Schwannoma, meningioma, or other benign CNS or meningeal disease which may require treatment with surgery or radiation therapy)
8. Concurrent therapy with any of the following (all must have been discontinued or substituted for at least 4 weeks prior to randomization):
   - Medications known to lower the seizure threshold
   - Herbal and non-herbal products that may decrease PSA levels (i.e., saw palmetto, pomegranate juice)
   - Systemic (oral/IV/IM) corticosteroids. Short term use (≤ 4 weeks) of corticosteroids during the study is allowed if clinically indicated, but it should be tapered off as soon as possible
   - Any other experimental treatment on another clinical trial
9. History or evidence of any of the following conditions:
   - Any prior malignancy (other than adequately treated basal cell or squamous cell skin cancer, superficial bladder cancer, or any other cancer in situ currently in complete remission)

	within 5 years prior to randomization<br>▪ Severe/unstable angina, myocardial infarction, symptomatic congestive heart failure, arterial or venous thromboembolic events (e.g., pulmonary embolism, cerebrovascular accident including transient ischemic attacks), or clinically significant ventricular arrhythmias within 6 months prior to randomization<br>▪ Uncontrolled hypertension (≥ 160 mmHg systolic blood pressure and/or diastolic blood pressure ≥ 100 mmHg)<br>▪ Gastrointestinal disorder affecting absorption<br>▪ Active infection, such as human immunodeficiency virus (HIV)<br>▪ Any other condition that, in the opinion of the Investigator, would impair the patient's ability to comply with study procedures
**Treatment Arm, Dose, and Route of Administration**	ARN-509 Softgel Capsules will be administered orally on a continuous daily dosing regimen, at a starting dose of 240 mg QD (eight 30 mg capsules per day). Patients experiencing GI discomfort will be allowed to switch to a BID regimen (four 30 mg capsules in the morning and four 30 mg capsules in the evening) as needed.
**Control Arm, Dose, and Route of Administration**	Matched placebo will be administered orally on a continuous daily dosing regimen, at a starting dose of 240 mg QD (eight 30 mg capsules per day). Patients experiencing GI discomfort will be allowed to switch to a BID regimen (four 30 mg capsules in the morning and four 30 mg capsules in the evening) as needed.
**Safety Assessments**	Patients will be assessed for adverse events at each monthly clinic visit while on the study. Adverse events will be graded according to the NCI Common Terminology Criteria for Adverse Events (CTCAE) Version 4.0. Type, incidence, severity, timing, seriousness, and relatedness of adverse events and laboratory abnormalities will be reported.
**Data Monitoring Committee**	An independent third-party Data Monitoring Committee (DMC) will monitor the safety of the patients, with meetings at a minimum of 2 times per year to determine overall safety and benefit:risk assessment.<br>Periodic quarterly adverse event data review will also be performed by designated members of the Sponsor's primary study team and will be blinded to treatment assignment with adverse event from

	both treatment groups combined. Any safety issues of concern identified by the primary study team will be promptly reported to the DMC.
**Efficacy Assessments**	Disease assessments will be performed as scheduled according to the calendar, regardless of treatment delays resulting from toxicity. Disease assessments (CT scans of the chest, abdomen, and pelvis, plus bone scan) will be performed at baseline and at 16-week intervals until documented progression. All scans will be submitted for blinded independent central review (BICR) by a third-party core imaging laboratory to confirm patient eligibility (i.e., no presence of distant metastases) and disease progression during the study.<br><br>The DMC will also serve as the primary reviewers of efficacy. Its membership and governance will be outlined in a separate charter that will define the rules for early termination, modification and continuation of the study, as well as how those recommendations will be made to the Sponsor.
**Primary Endpoint**	▪ Metastasis-Free Survival (MFS)
**Key Secondary Endpoint**	▪ Overall Survival (OS)
**Other Secondary Endpoints**	▪ Time to symptomatic progression<br>▪ Time to initiation of cytotoxic chemotherapy<br>▪ Radiographic Progression-Free Survival (PFS)<br>▪ Time to Metastasis (TTM)
**Other Evaluations**	▪ Health-related quality of life and prostate cancer-specific symptoms<br>▪ Type, incidence, severity, timing, seriousness, and relatedness of adverse events and laboratory abnormalities<br>▪ Population pharmacokinetics<br>▪ Assessment of ventricular repolarization [Appendix 8]
**Statistical Analysis Plan and Rationale for Number of Patients**	The primary efficacy analysis will be event-driven. Based on results from a large Phase III study of denosumab versus placebo in high risk NM-CRPC patients, the expected median MFS in the control arm is 25 months.<br><br>Applying a 2:1 randomization, a planned accrual period of 24 months and a minimum follow-up period of [redacted] months, it is estimated that approximately 1200 patients will need to be enrolled in order to observe [redacted] MFS events. This sample size has 90%

	power to detect a [redacted] reduction in the risk of developing metastases (HR = [redacted]) for patients receiving ARN-509, with a 2-sided α of 0.05. This treatment effect corresponds to an increase in median MFS of approximately [redacted] months (from 25 to [redacted] months), which would be considered clinically relevant.<br><br>The study is also designed to provide 85% power to detect a [redacted] reduction (HR = [redacted]) in the risk of death for patients receiving ARN-509, based on an assumed median OS of [redacted] months in the placebo arm. This treatment effect represents an increase in median OS of approximately [redacted] months (from [redacted] to [redacted] months).<br><br>There will be one interim analysis for OS at the time of the efficacy analysis of MFS, when approximately [redacted] OS events are expected to be observed, representing [redacted] of the final OS analysis. The final analysis of OS will occur after approximately [redacted] deaths have occurred. In order to adjust for a single interim analysis of OS, which will occur only if the primary analysis of MFS is statistically significant, the levels of significance for the interim and final analyses of OS will be [redacted] and [redacted], respectively.
**Duration of Patient Participation and Duration of Study**	Patients will remain on study until disease progression, development of unacceptable toxicity, or withdrawal of consent.<br><br>With an estimated accrual duration of 24 months, it is assumed that patients are expected to be followed for a minimum of approximately [redacted] months beyond Last Patient In (LPI) for the primary endpoint of MFS, to approximately [redacted] months beyond LPI for the key secondary endpoint of OS. This corresponds to total projected study duration of 59 months.<br><br>If the study is not terminated beforehand per the recommendation of the DMC, the end of trial in all participating countries will be defined as the time at which the secondary endpoint of OS has been met. At that time, the protocol may be amended to minimize the number of protocol assessments to only collection of safety data for those patients remaining on study.

LIST OF ABBREVIATIONS

**ADT**	androgen deprivation therapy
**AE**	adverse event
**ALT**	alanine aminotransferase
**ANC**	absolute neutrophil count
**AR**	androgen receptor
**AST**	aspartate aminotransferase
**BICR**	blinded independent central review
**BID**	bis en die (twice daily)
**BSE**	bovine spongiform encephalopathy
**BUN**	blood urea nitrogen
**CFR**	Code of Federal Regulations
**CNS**	central nervous system
**CRF**	case report form
**CRPC**	castration-resistant prostate cancer
**CTCAE**	Common Terminology Criteria for Adverse Events
**DLT**	dose-limiting toxicity
**DMC**	data monitoring committee
**ECG**	electrocardiogram
**EDC**	electronic data capture
**EWB**	emotional well-being
**FACT-P**	Functional Assessment of Cancer Therapy-Prostate
**FDA**	Food and Drug Administration
**FWB**	functional well-being
**GCP**	Good Clinical Practice
**GI**	gastrointestinal
**GnRHa**	gonadotropin releasing hormone analog
**HIPAA**	Health Insurance Portability and Accountability Act of 1996
**HIV**	human immunodeficiency virus
**ICF**	informed consent form
**ICH**	International Conference on Harmonisation
**IEC**	Independent Ethics Committee
**IRB**	Institutional Review Board
**IV**	intravenous
**LDH**	lactate dehydrogenase

**MFS**	metastasis-free survival
**NCI**	National Cancer Institute
**NM-CRPC**	non metastatic castration-resistant prostate cancer
**OS**	overall survival
**PCWG2**	Prostate Cancer Clinical Trials Working Group 2
**PET**	positron emission tomography
**PFS**	progression-free survival
**PI**	principal investigator
**PD**	progressive disease
**PD**	pharmacodynamic
**PK**	pharmacokinetic
**PSA**	prostate-specific antigen
**PSADT**	prostate-specific antigen doubling time
**PWB**	physical well-being
**QD**	quaque die (once daily)
**QTc**	corrected QT interval
**QTcB**	corrected QT interval according to the Bazett correction
**QTcF**	corrected QT interval according to the Fridericia correction
**RECIST**	Response Evaluation Criteria in Solid Tumors
**RP2D**	recommended phase 2 dose
**SAE**	serious adverse event
**SGOT**	serum glutamic oxaloacetic transaminase
**SGPT**	serum glutamate pyruvate transaminase
**SOC**	system organ class
**SWB**	social well-being
**TPGS**	d-α-tocopheryl polyethylene glycol 1000 succinate
**TTM**	time to metastasis
**ULN**	upper limit of normal

TABLE OF CONTENTS

1. BACKGROUND 18
1.1 ARN-509 18
1.1.1 Molecular Formula and Chemical Class 19
1.1.2 Pre-Clinical Development Overview 19
Figure 1 Tumor Growth Inhibition in Castration-resistant LNCaP/AR-Luc Xenograft Model After 28 Days of Treatment with Bicalutamide or ARN-509 20
1.1.3 Overview of Clinical Studies 21
Figure 2 Waterfall Plot of the 12-week PSA Response in Patients with High Risk NM-CRPC 23
2. STUDY OBJECTIVES 24
2.1 PRIMARY OBJECTIVE 24
2.2 KEY SECONDARY OBJECTIVE 24
2.3 OTHER SECONDARY OBJECTIVES 24
3. STUDY DESIGN 25
3.1 STUDY OVERVIEW AND RATIONALE 25
3.1.1 Selection of the Primary Endpoint 26
3.2 STUDY OUTCOMES 27
3.2.1 Primary Efficacy Endpoint 27
3.2.2 Key Secondary Efficacy Endpoint 27
3.2.3 Other Secondary Endpoints 27
3.2.4 Other Evaluations 27
4. PATIENT SELECTION 28
4.1 INCLUSION CRITERIA 28
4.2 EXCLUSION CRITERIA 29
5. STUDY TREATMENTS 31
5.1 RANDOMIZATION CRITERIA 31
5.2 BLINDING 31
5.3 FORMULATION 32
5.3.1 ARN-509/Matched Placebo 32
5.3.2 Packaging, Storage, and Labeling 32
5.3.3 Drug Administration 33
5.3.4 Cycle Management 33
5.3.5 Dose Modifications 33
Table 1 ARN-509 Dose Levels 34
5.4 STUDY DRUG ACCOUNTABILITY 34
5.5 MEASURES OF TREATMENT COMPLIANCE 34
6. CONCURRENT MEDICATIONS 35
6.1 PROHIBITED MEDICATIONS AND TREATMENTS 35
6.2 RESTRICTED THERAPIES 35
6.3 LIFE STYLE GUIDELINES 35

7. STUDY PROCEDURES AND GUIDELINES ... 37
7.1 CLINICAL ASSESSMENTS ... 37
7.1.1 Demographics ... 37
7.1.2 Medical History ... 37
7.1.3 Physical Examination ... 37
7.1.4 Vital Signs ... 37
7.1.5 Performance Status ... 38
7.1.6 Adverse Events ... 38
7.1.7 Concomitant Medications ... 38
7.1.8 Tumor Assessments ... 38
7.1.8.1 Blinded Independent Central Review (BICR) ... 38
7.2 CLINICAL LABORATORY MEASUREMENTS ... 39
7.2.1 PSA ... 39
7.2.2 Electrocardiogram (ECG) ... 39
7.3 PHARMACOKINETIC MEASUREMENTS ... 39
7.4 PATIENT REPORTED OUTCOMES ... 39
8. STUDY ASSESSMENTS BY VISIT (APPENDIX 1) ... 41
8.1 SCREENING (WITHIN 28 DAYS OF THE FIRST DOSE OF STUDY DRUG) ... 41
8.2 CYCLE 1 DAY 1 ... 41
8.3 DAY 1 OF CYCLES N (+/- 2 DAYS) ... 41
8.4 EVERY 16 WEEKS (DAYS 1 OF CYCLES 5, 9, 13, ETC.) (+/- 2 DAYS) ... 42
8.5 END OF TREATMENT ... 42
8.6 SAFETY FOLLOW-UP (28 DAYS FOLLOWING THE LAST DOSE OF STUDY DRUG) ... 42
8.7 SURVIVAL FOLLOW-UP ... 42
9. ADVERSE EVENT REPORTING REQUIREMENTS ... 44
9.1 DEFINITIONS ... 44
9.1.1 Adverse Event (AE) ... 44
9.1.2 Serious Adverse Event (SAE) ... 44
9.1.3 Expectedness ... 45
9.1.4 Attribution ... 45
9.1.5 Severity ... 46
Table 2 AE Severity Grading ... 46
9.1.6 Exposure during Pregnancy ... 46
9.2 REPORTING REQUIREMENTS ... 48
9.2.1 SAE Reporting ... 48
9.2.2 Non-Serious AE Reporting ... 49
9.2.3 Sponsor Reporting Requirements to Regulatory Authorities ... 50
10. END OF TREATMENT ... 51
11. PROTOCOL VIOLATIONS ... 52
12. DATA MONITORING COMMITTEE ... 53
13. STATISTICAL METHODS AND CONSIDERATIONS ... 54

13.1 ANALYSIS POPULATIONS 54
13.2 EFFICACY ANALYSES 54
13.2.1 Analysis of Primary Endpoint 54
13.2.2 Analysis of Key Secondary Endpoint 55
13.2.3 Analyses of Other Secondary Endpoints 56
13.2.3.1 Time to Symptomatic Progression 57
13.2.3.2 Time to Initiation of Cytotoxic Chemotherapy 57
13.2.3.3 Radiographic Progression-Free Survival 57
13.2.3.4 Time to Metastasis 58
13.3 SAFETY EVALUATIONS 59
13.3.1 Analysis of Adverse Events 59
13.3.2 Analysis of Clinical Laboratory Results 60
13.3.3 PSA 60
13.3.4 Analysis of Vital Signs 60
13.3.5 Concomitant Medications/Treatments 60
13.4 OTHER EVALUATIONS 61
13.4.1 Health-Related Quality of Life and Prostate Cancer-Specific Symptoms 61
13.4.2 Population Pharmacokinetics (Pop PK) 61
13.4.3 Assessment of Ventricular Repolarization 61
13.5 INTERIM ANALYSIS 62
13.6 DETERMINATION OF SAMPLE SIZE 62
14. DATA COLLECTION, RETENTION AND MONITORING 63
14.1 DATA COLLECTION INSTRUMENTS 63
14.2 DATA MANAGEMENT PROCEDURES 63
14.3 DATA QUALITY CONTROL AND REPORTING 63
14.4 ARCHIVAL OF DATA 63
14.5 AVAILABILITY AND RETENTION OF INVESTIGATIONAL RECORDS 64
14.6 MONITORING 64
14.7 PATIENT CONFIDENTIALITY 64
15. ADMINISTRATIVE, ETHICAL, REGULATORY CONSIDERATIONS 65
15.1 PROTOCOL AMENDMENTS 65
15.2 INSTITUTIONAL REVIEW BOARDS AND INDEPENDENT ETHICS COMMITTEES65
15.3 INFORMED CONSENT FORM 66
15.4 REPORTING OF SAFETY ISSUES AND SERIOUS BREACHES OF THE PROTOCOL OR ICH GCP 66
15.5 END OF TRIAL IN ALL PARTICIPATING COUNTRIES 67
15.6 SPONSOR DISCONTINUATION CRITERIA 67
15.7 PUBLICATIONS 67
16. REFERENCES 68
17. APPENDICES 70
Appendix 1: Schedule of Activities 71
Appendix 2: Required Laboratory Tests 73
Appendix 3: ECOG Performance Status 74

Appendix 4: National Cancer Institute (NCI) Common Terminology Criteria for Adverse Events (CTCAE) 75
Appendix 5: Prohibited or Restricted Medications or Supplements While On Study 76
Appendix 6: FACT-P Questionnaire 77
Appendix 7: EQ-5D 79
Appendix 8: Ventricular Repolarization Sub-Study at Selected Sites 81

1. BACKGROUND

Prostate cancer is the second most frequently diagnosed cancer and the sixth leading cause of cancer death in males, accounting for 14% (903,500) of the total new cancer cases and 6% (258,400) of the total cancer deaths in males worldwide.[1] Treatment aimed at eradicating the primary tumor, typically with surgery or radiation, is unsuccessful in ~30% of men, who develop recurrent disease that usually manifests first as a rise in plasma prostate-specific antigen (PSA) followed by metastasis to distant sites.[2] Given that prostate cancer cells depend on androgen receptor (AR) for their proliferation and survival, the standard treatment for patients with recurrent disease is androgen deprivation therapy (ADT) with a gonadotropin releasing hormone analog (GnRHa) with or without an anti-androgen.

Treatment results with ADT are generally predictable: a decline in PSA followed by tumor regression, a period of stability in which the tumor does not proliferate and PSA remains stable, followed by rising PSA and regrowth as a castration-resistant disease. Nearly all men with progressive prostate cancer eventually develop castration-resistant disease. Prostate cancer progression despite castrate levels of testosterone represents a transition to a lethal disease stage.

Molecular profiling studies of castration-resistant prostate cancer (CRPC) commonly show increased AR gene expression.[3] The increased AR levels are sufficient to confer resistance to anti-androgen therapy in mouse models, shorten tumor latency and confer agonist properties to current FDA-approved AR antagonists, such as bicalutamide or flutamide.[4] The potential for agonist activity by these approved anti-androgens in the setting of increased AR expression is a potential liability, best illustrated by the observation of tumor regression and declines in PSA following discontinuation of either of these AR antagonists, the so-called anti-androgen withdrawal syndrome.[5] Collectively, these findings implicate increased AR levels as one mechanism of drug resistance. They also suggest that drugs retaining antagonism and not displaying agonism in cells over-expressing AR levels might be useful therapeutically.

ARN-509 is a new generation AR antagonist that has been developed to overcome the potential therapeutic deficiencies of first generation AR antagonists (e.g., bicalutamide).

1.1 ARN-509

ARN-509 is an orally available, potent and selective AR antagonist that acts by inhibiting the action of androgen, nuclear translocation of the AR and DNA binding to androgen response elements and unlike bicalutamide, it exhibits no significant agonist activity in AR-over-expressing prostate cancer cells.[6]

Complete information for ARN-509 can be found in the Investigator's Brochure, the safety reference document for this study

1.1.1 Molecular Formula and Chemical Class

ARN-509 drug substance is a white to off-white crystalline solid.

Chemical Name: (4-[7-(6-Cyano-5-trifluoromethylpyridin-3-yl)-8-oxo-6-thioxo-5,7-diazaspiro[3.4]oct-5-yl]-2-fluoro-*N*-methylbenzamide)

Chemical Structure:

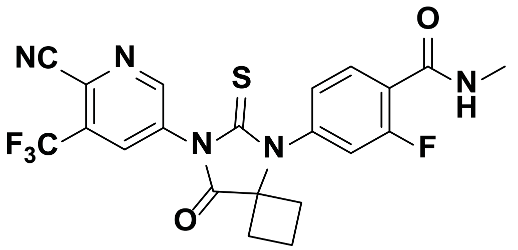

Molecular Formula: $C_{21}H_{15}F_4N_5O_2S$
Molecular Weight: 477.44

1.1.2 Pre-Clinical Development Overview

ARN-509's mechanism of action is through antagonism of androgen action and inhibition of AR nuclear translocation and DNA binding to androgen response elements, a mechanism that is distinct from the FDA-approved anti-androgen bicalutamide. Unlike bicalutamide, it shows no significant agonist properties in an in vitro model of CRPC (e.g., AR-over-expressing prostate cancer cells; LNCaP/AR cells). Gene transcription of the androgen-driven genes, PSA and TMPRSS2, is inhibited by ARN-509 and results in concentration-dependent reduction of these protein levels in vitro. ARN-509 was also shown to reduce proliferation of CRPC cells as well as increase apoptosis and necrosis in vivo. These effects are supported by the anti-tumor activity of ARN-509 observed in murine tumor models of CRPC. In these models, ARN-509 showed dose-dependent tumor growth inhibition and tumor regression that were superior to bicalutamide. Figure 1 depicts the percent change in tumor volume and plasma concentrations (filled circles above waterfall plot) of bicalutamide and ARN-509 on Day 28.

Figure 1 Tumor Growth Inhibition in Castration-resistant LNCaP/AR-Luc Xenograft Model After 28 Days of Treatment with Bicalutamide or ARN-509

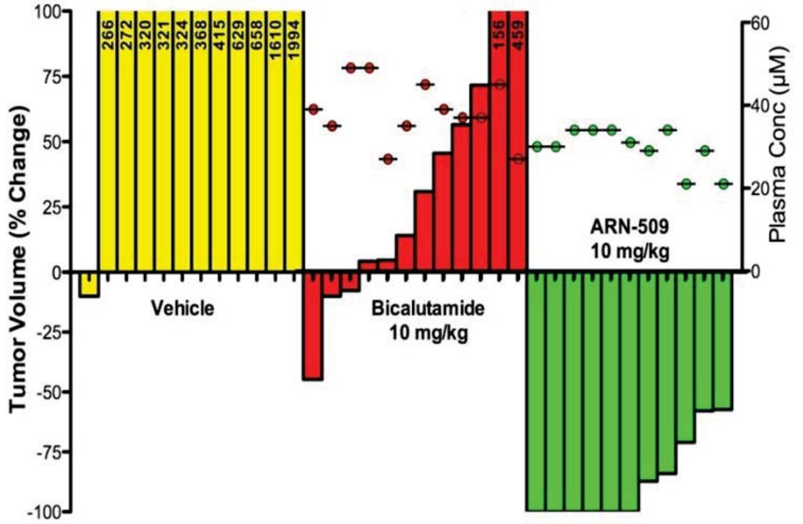

ARN-509 is a low clearance molecule with moderate volume of distribution in mice, rats and dogs. High oral bioavailability was obtained with the lipid-based formulation used in exploratory and definitive repeat-dose toxicology studies in rats and dogs. Plasma exposure in rats and dogs increased in a dose-proportional manner over a dose range of 50-300 mg/kg/day and 5-40 mg/kg/day, respectively. Exposure per unit dose was greatest in the dog, which also had the lowest clearance (less than 2% of hepatic blood flow) and the longest half-life (greater than 60 hours), resulting in significant accumulation with multiple doses and requiring 3 weeks of daily dosing for systemic exposure to achieve steady state plateau.

Four metabolites have been identified with different proportions between species. All four were assessed for their on-target effects against the androgen receptor. Metabolite M1 was found to be essentially inactive as an AR antagonist, while metabolites M2 and M4 were approximately [redacted] potent against AR than ARN-509. Metabolite M3 was the most potent AR antagonist, but was still [redacted] potent than ARN-509. M3 (ARN000308) is considered the predominant metabolite, with a longer elimination half-life than ARN-509.

Single-dose and repeat-dose toxicology studies have been conducted in male Sprague Dawley (SD) rats and Beagle dogs (repeat-dose studies only). Acute administration of ARN-509 at 1000 mg/kg was well tolerated in SD rats, with no morbidity, mortality or significant effects on body weight or serum chemistry markers. In repeat-dose toxicology studies, ARN-509 was well tolerated at doses up to 50 mg/kg/day in SD rats and 10 mg/kg/day in Beagle dogs. In male SD rats, lethality was observed at doses of 150 mg/kg/day and greater. The morbidity/mortality observed at these doses occurred within the first 5 days of dosing; however, animals that did survive at these higher doses, appeared to develop a tolerance for the test article with extended exposure. Clinical signs observed in the moribund animals were piloerection, hypothermia, breathing abnormalities, dehydration and decreased activity. The cause of the morbidity/mortality in male rats could not be

determined by pathologic examination. Key clinical pathology changes at doses of 150 mg/kg/day or greater included significant increases in cholesterol (greater than 200% from controls), decreases in erythrocytes, hemoglobin and hematocrit, and increases in reticulocytes, platelets, leukocytes, lymphocytes, basophils, and aPTT. The increase in cholesterol is attributed to the anti-androgen activity of ARN-509 and is believed to be responsible for the stated hematologic changes. Examination of red blood cell morphology revealed changes that were consistent with excess cholesterol being transferred to the outer membrane of the erythrocytes, resulting in a mild hemolytic anemia. Pharmacologic effects were also observed in the male accessory sex organs (epididymides, prostate, seminal vesicles and to a lesser degree, the testes) at ARN-509 doses as low as 50 mg/kg/day. Other target organs in the rat that were observed at ARN-509 doses of 150 mg/kg/day or higher included adrenals (also at 50 mg/kg/day), liver, pituitary, thyroid, spleen, salivary glands, mammary gland, and stomach. With the exception of the salivary glands and stomach, the effects on those organs are also believed to be due to the anti-androgen effect of ARN-509 and in many cases are specific to the physiology of the rat.

In male Beagle dogs, seizures necessitating humane euthanasia occurred at ARN-509 doses of 25 mg/kg/day and greater, 7-14 days after dosing was initiated. Clinical signs at 25 mg/kg/day included convulsions, intermittent tremors and decreased activity. It is likely that the convulsive seizures observed in dogs at very high doses are the result of ARN-509's functional antagonism of the $GABA_A$ receptor. This is similar to what has been observed with other second generation AR antagonists.[7, 8]

Other clinical pathology and target organ changes were limited to increases in cholesterol (up to 50% compared to controls) and effects on the epididymides, prostate and testes at all doses tested and attributed to the anti-androgen effect of ARN-509.

In the final week of the 28-day GLP repeat-dose toxicology studies, a full functional observation battery was performed to assess neurological and behavioral effects of ARN-509 on all surviving rats, and blood pressure and electrocardiogram assessments were performed on all surviving dogs to assess the cardiovascular effects of ARN-509. No test article-related effects on the parameters measured were identified in either of the assessments at the highest doses tested (150 mg/kg/day) in the rat and (10 mg/kg/day) in the dog. Based on these studies, 30 mg was defined as the starting dose for the first clinical study in human subjects.

1.1.3 Overview of Clinical Studies

A Phase I/II study (Protocol ARN-509-001) was designed to assess the safety, pharmacokinetics, and antitumor activity of ARN-509 in men with CRPC. The Phase I portion of the study has been completed and the Phase II is currently ongoing.

During Phase I, 30 patients with progressive, metastatic CRPC received ARN-509 at doses ranging from 30 to 480 mg given on a continuous daily dosing regimen. There was only one dose-limiting toxicity (DLT) observed in the 300 mg cohort: one patient experienced Grade 3 abdominal pain that resolved upon study drug interruption and subsequent dose reduction. As a result of this DLT, an additional 3 patients were enrolled in the 300 mg cohort. No additional DLTs were observed. The most frequent, treatment-related, adverse events

observed in > 10% of patients were fatigue (47%), nausea (30%), abdominal pain (20%), arthralgia (13%), diarrhea (13%), dyspnea (13%), and peripheral sensory neuropathy (13%). There were no serious adverse events or discontinuations due to adverse events. As of 20 August 2012, 9 patients remained on study, with the longest duration on study at 23 months. PSA response (at least 50% confirmed decline from baseline) at 12 weeks occurred in 48.3% of the patients across all dose levels.[9]

ARN-509 was rapidly absorbed, with measurable plasma concentrations within 30 minutes after ingestion of a single oral dose of 1 to 16 soft gelatin capsules (total ARN-509 dose, 30 to 480 mg). On average, peak plasma concentrations occurred 2 to 3 hours after administration in each dose group. The increases in plasma C_{max} values and in the area under the plasma concentration curve (AUC) were linear and dose proportional. Plasma ARN-509 concentrations declined slowly, with a mean half-life value at steady-state of 4 days.

Preliminary pharmacodynamic analysis of 18-fluorodihydrotestosterone positron emitting tomography (FDHT-PET) scans performed at baseline and at 4 weeks after starting treatment confirms robust AR blockade at doses of 120 mg/day. Together with preclinical projection of the optimal biological dose and combined with clinical safety and the observed pharmacokinetic profile, 240 mg was selected as the recommended Phase 2 Dose (RP2D); at that dose, mean C_{max} and AUC steady-state values were 7.6 ± 1.2 µg/mL and 127 ± 37 µg·h/mL, respectively.

In the Phase II portion of the study, a total of 97 patients were enrolled across 3 different patient population subsets: high risk NM-CRPC, metastatic CRPC, and metastatic CRPC with disease progression after abiraterone acetate.

To date in the NM-CRPC subset (n=47), the most frequent, treatment-related, adverse events observed in more than 3 (5%) patients are fatigue (30%), diarrhea (28%), nausea (17%), rash (13%), and abdominal pain (11%). Four Grade 3 events (diarrhea, hypertension, and two cases of rash) have been reported and 3 (6%) patients have discontinued the study due to adverse event, consent withdrawal, and compliance issues, respectively.

At 12 weeks post the start of ARN-509 treatment, PSA declines of ≥ 50% decline as compared to baseline have been observed in 91% of NM-CRPC patients (Figure 2).

Figure 2 Waterfall Plot of the 12-week PSA Response in Patients with High Risk NM-CRPC

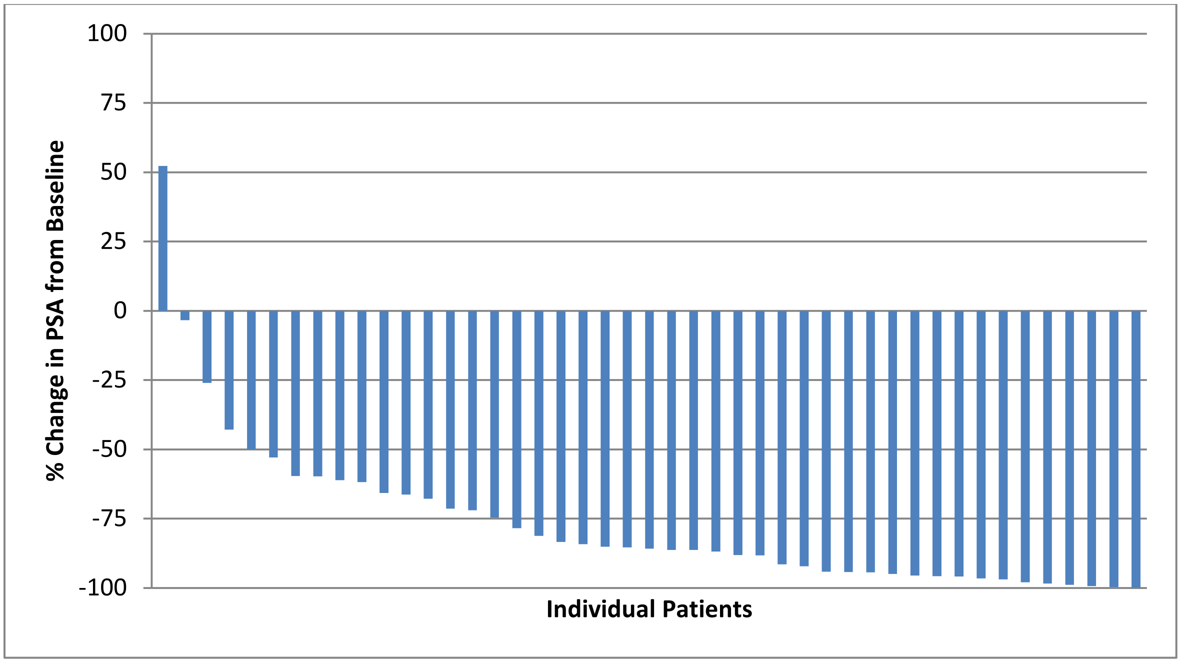

2. STUDY OBJECTIVES

2.1 PRIMARY OBJECTIVE

- To demonstrate superiority in the metastasis-free survival (MFS) of men with high risk NM-CRPC treated with ARN-509 versus placebo

2.2 KEY SECONDARY OBJECTIVE

- To compare the overall survival (OS) of men with high risk NM-CRPC treated with ARN-509 versus placebo

2.3 OTHER SECONDARY OBJECTIVES

- To compare the time to symptomatic progression in men with high risk NM-CRPC treated with ARN-509 versus placebo
- To compare the time to initiation of cytotoxic chemotherapy in men with high risk NM-CRPC treated with ARN-509 versus placebo
- To compare the radiographic progression-free survival (PFS) of men with high risk NM-CRPC treated with ARN-509 versus placebo
- To compare the time to metastasis (TTM) in men with high risk NM-CRPC treated with ARN-509 versus placebo
- To compare patient reported outcomes (PROs) of health-related quality of life and prostate cancer-specific symptoms in men with high risk NM-CRPC treated with ARN-509 versus placebo
- To evaluate the safety and tolerability of ARN-509
- To evaluate the population pharmacokinetics of ARN-509
- To evaluate the effect of ARN-509 on ventricular repolarization in a subset of patients from selected clinical sites [Appendix 8]

3. STUDY DESIGN

3.1 STUDY OVERVIEW AND RATIONALE

This is a randomized (2:1), multicenter, double-blind, placebo-controlled, Phase III clinical trial evaluating the efficacy and safety of ARN-509 versus placebo in men with high risk (M0) NM-CRPC, defined as PSA Doubling Time (PSADT) ≤ 10 months.

Short PSADT has been consistently associated with reduced time to first (bone) metastasis and death, thus the selected patient population represents one at high risk for development of (distant) metastasis and prostate cancer-specific death.[10-12]

Since there are no approved treatments available for the proposed patient population besides continuous administration of ADT (with or without a first generation anti-androgen) as part of the current *de facto* community standard practice[13,14], randomization to either ARN-509 or placebo is justified in this setting. Based on the Phase II preliminary results from Study ARN-509-001, ARN-509 is associated with a highly favorable safety profile, and the encouraging anti-tumor activity observed to date in the cohort of patients with high risk NM-CRPC indicates that ARN-509 might have the potential to be efficacious in this earlier line of therapy. The placebo arm will allow an objective comparison of safety and efficacy between treatment with ARN-509 and placebo.

A double-blind study design was chosen to preserve study integrity and minimize bias in the assessment of all study endpoints. A 2:1 randomization scheme will increase the probability that eligible patients will be randomized to receive ARN-509, thereby improving study feasibility.

Randomization will be stratified by PSADT, the use of a bone-sparing agent and the presence of loco-regional disease. These stratification factors were chosen on the basis that they may be sufficiently prognostic such that an imbalance may bias the results.

ARN-509 or matched placebo will be administered orally on a continuous daily dosing schedule at a starting dose of 240 mg per day, the RP2D selected based on preclinical projections of the optimal biological dose combined with safety and the pharmacokinetic/ pharmacodynamic profiles observed during Phase I.

Patients will be followed for safety and efficacy as per the schedule of activities and will remain on study treatment until documented radiographic progression (development of distant metastases as assessed by blinded independent central review) or the development of unacceptable toxicity. Treatment decisions will not be based on PSA as the clinical significance of PSA changes during treatment in the setting of NM-CRPC is unknown; therefore, PSA will be collected and analyzed by a central laboratory but Investigators, patients, and the Sponsor will be blinded to the results until the time of the primary analysis.

Patients discontinuing treatment due to documented radiographic progression will enter the survival follow-up period, where they will be followed for the development of symptomatic progression and initiation of subsequent anti-cancer therapies (in particular, cytotoxic

chemotherapy) every 4 months until death, loss of follow-up, or withdrawal of consent, whichever comes first.

Patients discontinuing treatment prior to documented radiographic progression will also enter the survival follow-up period where they will continue to have scheduled disease assessments every 4 months until documented radiographic progression, and will be followed for the development of symptomatic progression and initiation of subsequent anti-cancer therapies (in particular, cytotoxic chemotherapy) every 4 months until death, loss of follow-up, or withdrawal of consent, whichever comes first.

3.1.1 Selection of the Primary Endpoint

Men with high risk NM-CRPC, characterized by rapidly rising PSA in the absence of detectable metastases despite castrate levels of testosterone, are at significant risk for development of metastases and prostate cancer-specific death. As verified in a recently completed Phase III trial of denosumab, men with NM-CRPC who are at high risk for development of bone metastasis have a median overall survival of 44.8 months and a median bone-metastasis-free survival of 25.2 months.[15] To date there are no approved treatments or standard of care for men with high risk NM-CRPC and thus this patient population represents an area of unmet medical need.

In consideration of the relatively long median OS (~45 months) in this population and the opportunity to assess for the development of distant metastasis as a clinically important milestone, metastasis-free survival (MFS) was chosen as the primary objective of this study. Metastasis from prostate cancer, especially bone metastases, is the source of prostate cancer specific morbidity and mortality.[16, 17] In addition, it triggers the need for a change in therapy that can be associated with an increase in morbidity (e.g., toxicity associated with chemotherapy), and in some cases leading to a change in quality of life for that patient.

Delaying the emergence of radiographically detectable distant metastasis, or prolongation of MFS as proposed, is a clinically relevant outcome that can be robustly and reliably assessed for determination of the true impact of a treatment on the NM-CRPC disease. In a high risk cohort of patients with biochemical recurrence after radical prostatectomy and a PSADT < 15 months, metastatic prostate cancer was reported to account for an estimated 90% of all deaths[10,18]; it is therefore conceivable that delaying the development of metastases would likely translate to delaying prostate cancer-specific death. The proposed MFS definition incorporates not only time to (bone or soft tissue) distant metastasis but also time to death and thus MFS can be a reasonable correlate to overall survival. Of note, based on retrospective landmark analyses at 3 and 5 years from the Radiation Therapy and Oncology Group 92-02 randomized trial in patients with locally advanced prostate cancer who had been treated with ADT and external beam radiation therapy, distant metastasis has been shown to be consistent with all four of Prentice's criteria for being a potential surrogate endpoint for prostate cancer-specific survival at 10 years.[19]

3.2 STUDY OUTCOMES

3.2.1 Primary Efficacy Endpoint

- Metastasis-Free Survival (MFS)

3.2.2 Key Secondary Efficacy Endpoint

- Overall Survival (OS)

3.2.3 Other Secondary Endpoints

- Time to symptomatic progression
- Time to initiation of cytotoxic chemotherapy
- Radiographic Progression-Free Survival (PFS)
- Time to Metastasis (TTM)

3.2.4 Other Evaluations

- Health-related quality of life and prostate cancer-specific symptoms
- Type, incidence, severity, timing, seriousness, and relatedness of adverse events and laboratory abnormalities
- Population pharmacokinetics
- Assessment of ventricular repolarization [Appendix 8]

4. PATIENT SELECTION

This study can only fulfill its objectives if appropriate patients are enrolled. The following eligibility criteria are designed to select patients for whom protocol treatment is considered appropriate. All relevant medical and non-medical conditions should be taken into consideration when deciding whether this protocol is suitable for a particular patient.

No waivers will be granted for eligibility criteria deviations.

4.1 INCLUSION CRITERIA

1. Histologically or cytologically confirmed adenocarcinoma of the prostate without neuroendocrine differentiation or small cell features, with high risk for development of metastases, defined as PSADT ≤ 10 months
2. Castration-resistant prostate cancer demonstrated during continuous androgen deprivation therapy (ADT)/post orchiectomy, defined as 3 consecutive rises of PSA, 1 week apart, resulting in two 50% increases over the nadir, with the last PSA > 2 ng/mL
3. Maintain castrate levels of testosterone (< 50 ng/dL [1.72 nmol/L]) within 4 weeks of randomization and throughout the study
4. Patients currently receiving bone loss prevention treatment with bone-sparing agents (e.g., bisphosphonates, denosumab [Prolia®]) must be on stable doses for at least 4 weeks prior to randomization
5. Patients who received a first generation anti-androgen (e.g., bicalutamide, flutamide, nilutamide) as part of an initial combined androgen blockade therapy or as second-line hormonal therapy must show continuing disease (PSA) progression off the anti-androgen for at least 4 weeks prior to randomization
6. At least 4 weeks must have elapsed from the use of 5-α reductase inhibitors (e.g., dutasteride, finasteride, aminoglutethamide), estrogens, and any other anti-cancer therapy prior to randomization, including chemotherapy given in the adjuvant/neoadjuvant setting (e.g., clinical trial)
7. At least 4 weeks must have elapsed from major surgery or radiation therapy prior to randomization
8. Age ≥ 18 years
9. Eastern Cooperative Oncology Group (ECOG) Performance Status 0 or 1
10. Resolution of all acute toxic effects of prior therapy or surgical procedure to Grade ≤ 1 or baseline prior to randomization
11. Adequate organ function as defined by the following criteria:
    - Serum aspartate transaminase (AST; serum glutamic oxaloacetic transaminase [SGOT]) and serum alanine transaminase (ALT; serum glutamic pyruvic transaminase [SGPT]) ≤ 2.5 x upper limit of normal (ULN)

- Total serum bilirubin ≤ 1.5 x ULN
- Serum creatinine ≤ 2 x ULN
- Absolute neutrophil count (ANC) ≥ 1500/μL
- Platelets ≥ 100,000/μL
- Hemoglobin ≥ 9.0 g/dL
  - Administration of growth factors or blood transfusions will not be allowed within 4 weeks of the hematology labs required to confirm eligibility

12. Signed and dated informed consent document indicating that the patient (or legally acceptable representative) has been informed of all pertinent aspects of the trial prior to randomization
13. Willingness and ability to comply with scheduled visits, treatment plans, laboratory and radiographic assessments, and other study procedures, including ability to swallow large capsules, the completion of patient reported outcomes questionnaires and long-term survival follow-up visits

4.2 EXCLUSION CRITERIA

1. Presence of distant metastases, including CNS and vertebral or meningeal involvement. **Exception:** pelvic lymph nodes < 2 cm in short axis (N1) located below the iliac bifurcation are allowed
2. Symptomatic loco-regional disease requiring medical intervention, such as moderate or severe urinary obstruction or hydronephrosis due to primary tumor (e.g., tumor obstruction of bladder trigone)
3. Prior treatment with second generation anti-androgens (e.g., enzalutamide)
4. Prior treatment with CYP17 inhibitors (e.g., abiraterone acetate, orteronel, galeterone, ketoconazole)
5. Prior treatment with radiopharmaceutical agents (e.g., Strontium-89), immunotherapy (e.g., sipuleucel-T), or any other investigational agent for NM-CRPC (e.g., denosumab [Xgeva®])
6. Prior chemotherapy, except if administered in the adjuvant/neoadjuvant setting
7. History of seizure or condition that may pre-dispose to seizure (e.g., prior stroke within 1 year prior to randomization, brain arteriovenous malformation, Schwannoma, meningioma, or other benign CNS or meningeal disease which may require treatment with surgery or radiation therapy)
8. Concurrent therapy with any of the following (all must have been discontinued or substituted for at least 4 weeks prior to randomization):
   - Medications known to lower the seizure threshold (please see Appendix 5)
   - Herbal and non-herbal products that may decrease PSA levels (i.e., saw palmetto, pomegranate juice)

- Systemic (oral/IV/IM) corticosteroids. Short term use (≤ 4 weeks) of corticosteroids during the study is allowed if clinically indicated, but it should be tapered off as soon as possible
- Any other experimental treatment on another clinical trial

9. History or evidence of any of the following conditions:
    - Any prior malignancy (other than adequately treated basal cell or squamous cell skin cancer, superficial bladder cancer, or any other cancer in situ currently in complete remission) within 5 years prior to randomization
    - Severe/unstable angina, myocardial infarction, symptomatic congestive heart failure, arterial or venous thromboembolic events (e.g., pulmonary embolism, cerebrovascular accident including transient ischemic attacks), or clinically significant ventricular arrhythmias within 6 months prior to randomization
    - Uncontrolled hypertension (≥ 160 mmHg systolic blood pressure and/or diastolic blood pressure ≥ 100 mmHg)
    - Gastrointestinal disorder affecting absorption
    - Active infection, such as human immunodeficiency virus (HIV)
    - Any other condition that, in the opinion of the Investigator, would impair the patient’s ability to comply with study procedures

5. STUDY TREATMENTS

5.1 RANDOMIZATION CRITERIA

After patients have provided their written informed consent, completed all Screening assessments and received confirmation of eligibility, they will be randomized into the study using an Interactive Voice Randomization System (IVRS) and stratified based on:

- PSADT: > 6 months vs. ≤ 6 months
- Bone-sparing agent use: Yes vs. No
- Loco-regional disease: N0 vs. N1

In order to ensure accurate and consistent determination of PSADT across all sites, the IVRS will also provide PSADT calculations (using a linear regression model of the normal logarithm of PSA and time) based on PSA values obtained within 6 months prior to randomization that will be entered by the site into the IVRS.[20]

Those same PSA values should be used during Screening to determine whether the patient is eligible for the study (inclusion criterion #1). In order to pre-screen patients for possible enrollment into the study, PSADT can be calculated using the Memorial Sloan-Kettering Cancer Center (MSKCC) PSA Doubling Time prediction tool, available at the following website:
http://nomograms.mskcc.org/Prostate/PsaDoublingTime.aspx

5.2 BLINDING

Due to the objectives of the study, the identity of test and control treatments will not be known to investigators, research staff, or patients.

Only selected individuals not affiliated with the protocol will be unblinded to individual patient treatment assignment during the trial for the purposes of efficacy analyses and safety review. The randomization codes and all data sets will be stored in a secure area accessible only to these individuals, and only released on completion of the study and after the study database has been locked.

In emergency situations for reasons of patient safety (e.g., a serious unexpected/unlisted drug-related event; a medical emergency; a potentially life-threatening drug interaction), the blinding code may need to be broken. In those cases, whenever possible, a request for unblinding should be discussed with the Sponsor (or designee) prior to unblinding. Detailed instructions on the method for breaking the blind will be provided during site training and in the Investigator Site File.

5.3 FORMULATION

5.3.1 ARN-509/Matched Placebo

ARN-509/matched placebo is formulated as a nonaqueous, lipid-based solution that is filled into 30 mg strength, size 18 softgel oblong-shaped capsules (ARN-509 Softgel Capsules, 30 mg) which are non-printed, with a clear to hazy light yellow to yellow color. The only difference between ARN-509 and its matched placebo is the absence of the active ingredient in the matched placebo formulation.

Each 30 mg softgel capsule of ARN-509/matched placebo contains the following inactive ingredients: [redacted]

[redacted] is derived from bovine origin and certified in accordance with FDA's Guidance for Industry - The Sourcing and Processing of Gelatin to Reduce the Potential Risk Posed by Bovine Spongiform Encephalopathy (BSE) in FDA-regulated Products for Human Use (September 1997).

Each 30 mg capsule of ARN-509/matched placebo contains 54.2 to 65.0 IU (36.4 to 43.6 mg of d-a-tocopherol) of vitamin E in the form of Vitamin E TPGS. At 240 mg, patients will receive 434 to 520 IU (291 to 349 mg d-a-tocopherol) of vitamin E daily.

5.3.2 Packaging, Storage, and Labeling

ARN-509/matched placebo will be supplied as 30 mg, softgel capsules packaged in 30-ct, 100 cc HDPE bottles with child-resistant closures and tamper proof heat induction seals, and shipped in pre-qualified boxes designed to maintain a temperature of 2-8ºC (36-46ºF).

At the clinical site and at the patient's home, the study drug should be stored under refrigerated conditions between 2-8ºC (36-46ºF); however, it can be transported to and from the clinic at room temperature between 15-30°C (59-86°F).

The study drug will be periodically tested and monitored for its acceptable shelf life for at least the duration of the study. Any study drug that fails to comply with the manufactured specifications will be promptly removed from the clinical trial site and replaced with new supplies by the Sponsor (or designee).

Each bottle of study drug will be labeled with the required regulatory agency warning statement, the protocol number, the Sponsor's name, and directions for patient use and storage. The Investigator will ensure that the study drug is stored in appropriate conditions in a secure location with controlled access.

5.3.3 Drug Administration

ARN-509/matched placebo will be administered orally on a continuous daily dosing regimen at a dose of 240 mg per day (eight 30 mg capsules).

Except for the first dose on Cycle 1 Day 1 when patients will be administered study drug at the clinic, ARN-509/matched placebo will be self-administered on an outpatient basis during the study, once daily, with or without food.

5.3.4 Cycle Management

For the purposes of the study, a treatment cycle will consist of 4 weeks (28 days).

It is anticipated that individual patients may occasionally forget to take a dose. In those cases, missed doses should only be replaced if the patient remembers within a 12-hour window. After that, patients should just take the next dose the following day, without compensating for the missed dose.

In the event of dose delays due to transient toxicity, tumor assessments should remain on schedule independent of cycle length.

5.3.5 Dose Modifications

Intrapatient dose interruptions and/or reductions will be permitted provided that study discontinuation criteria have not been met (please see Section 10).

- Patients experiencing treatment-related seizure of any grade will have study drug permanently discontinued.
- At any given dose level, if patients experience gastrointestinal (GI) discomfort due to the number of capsules, they will be allowed to switch to a BID regimen as needed (Table 1).
- For patients experiencing Grades 1-2 treatment-related adverse events, short treatment breaks can be instituted as per the discretion of the Investigator until the severity of the toxicity decreases to Grade 1 or returns to baseline. If toxicity recurs, dose reductions to the next lower dose level will be allowed as per the discretion of the Investigator.
- For patients experiencing Grade ≥ 3 treatment-related adverse events other than seizure, study drug should be held until the severity of the toxicity decreases to Grade 1 or returns to baseline. If toxicity recurs at Grade 3 or higher, the dose of ARN-509 should be reduced to the next lower dose level. A maximum of 2 dose level reductions will be allowed.
- Any patient requiring > 28 days delay in treatment will meet the criteria for study treatment discontinuation.

Table 1 ARN-509 Dose Levels

Dose Level	Total Daily Dose	Number of 30 mg Capsules (QD)	Number of 30 mg Capsules (BID)*
0	240 mg	8	4 in the morning<br>4 in the evening
-1	180 mg	6	3 in the morning<br>3 in the evening
-2	120 mg	4	2 in the morning<br>2 in the evening

*** At any given dose level, if patients experience GI discomfort due to the number of capsules, they may switch to a BID regimen as needed.**

Doses reduced for study treatment-related toxicities should generally not be re-escalated, however, re-escalation back to the previous dose level may be permitted at the discretion of the Investigator and in consultation with the Sponsor (or designee).

5.4 STUDY DRUG ACCOUNTABILITY

The Sponsor (or designee) will ship study drug to the investigational sites. The initial study drug shipment will be shipped after all required regulatory documentation has been received by the Sponsor and a Clinical Trial Agreement fully executed. Subsequent study drug shipments will be made according to an automated resupply algorithm managed by the IVRS.

The study drug will only be dispensed to patients who meet the eligibility criteria and are randomized to a treatment arm in the trial. An accurate and current accounting of the dispensing and return of study drug for each patient will be maintained on an ongoing basis by the Investigator or his/her designated personnel. The number of study drug dispensed and returned by the patient will be recorded on the Investigational Product Accountability Log. The study monitor will verify these documents throughout the course of the study.

At the end of the study, the Sponsor will provide instructions as to the disposition of any unused study drug. If destruction at the site is authorized, the Investigator must ensure that all investigational product is destroyed in compliance with the applicable environmental regulations, institutional policy, and any other special instructions provided by the Sponsor. Drug destruction must be adequately documented.

5.5 MEASURES OF TREATMENT COMPLIANCE

At each clinic visit, patients will be asked to return any remaining study drug from the previous dosing cycle as well as all used and unused study drug containers.

Treatment compliance will be defined as the number of capsules taken divided by the expected number of capsules and reported as percentage. In case of dose reductions, the expected number of capsules should reflect the new dose level.

Capsules that are not returned will be considered to have been taken, unless otherwise specified in the case report form (CRF).

6. CONCURRENT MEDICATIONS

All patients should be maintained on the same medications throughout the entire study period, as medically feasible, with minimum introduction of new chronic therapies. Every medication or treatment taken by the patient during the trial and the reason for its administration must be recorded on the CRF. Standard medical treatment as applicable is allowed except for treatments noted in the exclusion criteria and/or listed in the prohibited medications section below.

6.1 PROHIBITED MEDICATIONS AND TREATMENTS

As a class effect, androgen receptor antagonists have been associated with seizures due to an off-target mechanism of action ($GABA_A$ inhibition).[7,8]

To date, no patients receiving ARN-509 have experienced seizures, however, in preclinical experiments, at very high doses, dogs treated with ARN-509 had tremors and generalized seizures. Patients will be closely monitored for seizures, but as a precautionary measure, drugs known to decrease the seizure threshold and/or cause seizure will be prohibited while on study. A list of these medications can be found in Appendix 5.

6.2 RESTRICTED THERAPIES

- Strong CYP3A4 inhibitors and inducers: the potential for drug-drug interactions with ARN-509 has not been tested clinically. ARN-509 is metabolized primarily by human CYP3A4, thus co-administration with strong inhibitors or inducers of CYP3A4 should be avoided as much as possible. A list of these medications can be found in Appendix 5.
- ARN-509 may also induce CYP3A4; therefore, caution should be taken when administered in conjunction with CYP3A4 substrates that have a narrow therapeutic index
- The potential for drug-drug interaction between ARN-509 and warfarin (e.g., Coumadin) is unknown at present. If a patient is taking Coumadin, re-assess PT/INR as clinically indicated and adjust the dose of Coumadin accordingly.
- Corticosteroids: due to possible resistance mechanisms which may be contributed by glucocorticoid receptor signalling, concurrent use of corticosteroids during the study is not recommended; short term use (≤ 4 weeks) will be allowed if clinically indicated, however, its use must be tapered off as soon as possible.
- Vitamin E: the ARN-509/matched placebo drug product formulation provides high daily doses of vitamin E. Patients should not take supplemental vitamin E while being treated on this study.

6.3 LIFE STYLE GUIDELINES

Patients of childbearing potential must agree to practice some form of effective contraception, such as vasectomy, double barrier contraception, or sexual abstinence prior to entering into the study and for 6 months following the last dose of study drug.

There are no dietary restrictions with the exception of grapefruit and pomegranate fruits and/or juice while the patient is on study (Appendix 5). Study treatment can be taken with or without food.

7. STUDY PROCEDURES AND GUIDELINES

A Schedule of Activities representing the required testing procedures to be performed during the study is diagrammed in Appendix 1.

Prior to conducting any study-related activities, written informed consent and any other authorizations must be signed and dated by the patient or patient's legal representative.

Every effort should be made to ensure that the protocol required tests and procedures are completed as described. However, it is anticipated that from time to time there may be circumstances outside of the control of the Investigator that may make it unfeasible to perform the test. In those cases, the Investigator should take all steps necessary to ensure the safety and wellbeing of the patient. When a protocol required test cannot be performed, the Investigator will document the reason for this and any corrective and preventive actions which he/she has taken to ensure that normal processes are adhered to as soon as possible. The study team should be informed of these incidents in a timely fashion.

7.1 CLINICAL ASSESSMENTS

7.1.1 Demographics

Demographic information (e.g., date of birth, gender, race) will be recorded at Screening, as allowed per local country privacy law regulations.

7.1.2 Medical History

Relevant medical history, including history of current disease, other pertinent clinical conditions, and information regarding underlying diseases will be recorded at Screening.

7.1.3 Physical Examination

A complete physical examination will be performed by either the Investigator or a sub-Investigator at Screening. Qualified staff (e.g., nurses or physician assistants) may complete the abbreviated physical exam at all other visits. New abnormal physical exam findings must be documented and followed by a physician or other qualified staff at the next scheduled visit.

The physical examination should include, but not limited to, general appearance, skin, neck, eyes, ears, nose, throat, breast, lungs, heart, abdomen, back, lymph nodes, extremities, and nervous system, as well as examination of known and suspected sites of disease. Height will be recorded at baseline only. Body weight will also be recorded at the start of each cycle.

7.1.4 Vital Signs

Body temperature, blood pressure, pulse and respirations will be performed after resting for 5 minutes at Screening and every subsequent clinic visit.

7.1.5 Performance Status

The Eastern Cooperative Oncology Group (ECOG) performance status scale will be used (Appendix 3) and will be assessed at Screening and every subsequent clinic visit.

7.1.6 Adverse Events

Assessment of adverse events will include type, incidence, severity (graded by the National Cancer Institute [NCI] Common Terminology Criteria for Adverse Events [CTCAE], Version 4.0), timing, seriousness, and relatedness (Appendix 4). Adverse events will be assessed at every clinic visit.

7.1.7 Concomitant Medications

All concomitant medication and concurrent therapies will be documented at Screening and every subsequent clinic visit. Dose, route, unit frequency of administration, and indication for administration and dates of medication will be captured.

7.1.8 Tumor Assessments

Disease assessments are to be performed as scheduled according to the calendar, regardless of treatment delays resulting from toxicity. Care must be taken in scheduling disease assessments to prevent the introduction of bias based on treatment delays.

Disease assessments will be performed at baseline (Screening) and at 16-week intervals thereafter. Imaging studies will include a CT scan of the chest, abdomen, and pelvis, plus a bone scan. At Screening, there will be an additional CT of the brain to rule out the presence of CNS metastases.

Radiographic confirmation of disease progression (appearance of distant metastasis) will be based on RECIST 1.1 and assessed by blinded independent central review (see below).[21] For new bone lesions detected on bone scans, a second imaging modality (e.g., CT or MRI) will be required to confirm progression.

The same method of assessment and the same technique should be used at Screening and during follow-up. Intravenous (IV) contrast is required when not medically contraindicated. Patients who have a contraindication to IV contrast may have MRI exams of the brain, abdomen and pelvis performed in lieu of CTs and a non-contrast CT of the chest. Tumor evaluation by positron emission tomography (PET) scan or by ultrasound may not substitute for CT or MRI scans, but the CT portion of a PET/CT may be submitted in lieu of a dedicated CT. Additional requirements are provided in the Imaging Site Manual.

7.1.8.1 Blinded Independent Central Review (BICR)

All scans will be submitted to a third-party core imaging laboratory for independent review of patient eligibility (within 3 days of receipt of imaging scans that pass quality assessment) and disease progression during the study according to an Independent Review Charter to be prepared by the core imaging laboratory in consultation with the Sponsor.

It is important to the integrity of the study that all imaging studies and pertinent clinical information (e.g., bone trauma, fracture, or infection) are forwarded to the core imaging laboratory throughout the study.

Further details regarding materials to be forwarded for central review can be found in the Imaging Manual and/or Investigator Site File.

7.2 CLINICAL LABORATORY MEASUREMENTS

Blood and urine will be obtained at the time points described in the Schedule of Activities and sent to a central lab for hematology, blood chemistry profile, and urinalysis, respectively. Appendix 2 lists all of the specific tests that will be performed. Complete details with regards to sample collection and shipment processes can be found in the Laboratory Manual and/or Investigator Site File.

Investigators may have additional blood and urine tests performed for the purpose of planning treatment administration, dose modification, or following adverse events.

7.2.1 PSA

PSA will also be analyzed at the central laboratory. Results will be kept blinded to the patients, the Investigators, and the Sponsor, in order to preserve the double-blind nature of this study.

7.2.2 Electrocardiogram (ECG)

A standard12-lead ECG (with a 10-second rhythm strip) will be collected at Screening and as clinically indicated. ECGs should be collected after the patient has rested quietly and is awake in a fully supine (or semi-recumbent, if supine is not tolerated) position for 10 minutes, and prior to any blood draw collection.

7.3 PHARMACOKINETIC MEASUREMENTS

Blood for determination of plasma concentrations of ARN-509 and its metabolites ARN000308 and ARN000066 will be collected as described in the Laboratory Manual on Day 1 of Cycles 1, 2, 3, 6, 12, 18, 24, 36, yearly thereafter, and at the end of treatment. All samples will be assayed using an analytical method validated for ARN-509 and both metabolites.

On Cycle 1 Day 1, the blood sample must be collected between 0.5 and 4 hours post the first dose of ARN-509. For all other PK blood collections, the blood sample can be collected at any time on the scheduled clinic visit day; all reasonable measures must be taken to ensure accurate recording of information on dosing, including the time of the last two doses of ARN-509 and whether they were taken based on a QD or BID schedule.

7.4 PATIENT REPORTED OUTCOMES

At the start of each new cycle of treatment (Day 1), at the end of treatment, and during survival follow-up, patients will be required to complete two self-administered quality of life

instruments: the Functional Assessment of Cancer Therapy-Prostate (FACT-P) and the Euro-QoL Group EQ-5D.[22, 23]

The FACT-P will be used to assess health-related quality of life and prostate cancer-specific symptoms. The FACT-P consists of the 27-item FACT-General (FACT-G) and 12 items for the prostate cancer specific concerns. The 27 items in FACT-G are grouped into 4 domains: physical well-being (PWB), social/family well-being (SWB), emotional well-being (EWB) and functional well-being (FWB). The 12 prostate cancer symptoms items focus on pain (3 items), urination problems (3 items), and sexual functions (2 items). In addition, it also contains items for weight loss, appetite, overall comfort, and bowel movement. The FACT-P can be completed in 15 minutes.

The EQ-5D is a validated and reliable self-administered instrument used to assess health status. It contains 6 items designed to assess health status in terms of a single index value or health utility score. The EQ-5D can be completed in less than 5 minutes.

8. STUDY ASSESSMENTS BY VISIT (APPENDIX 1)

8.1 SCREENING (WITHIN 28 DAYS OF THE FIRST DOSE OF STUDY DRUG)

- Review the study with the patient (patient's legal representative) and obtain written informed consent
- Calculate PSADT to confirm patient eligibility
- Obtain CT of the brain, chest, abdomen, and pelvis, plus bone scan and submit for BICR to confirm patient eligibility
- Record demographics data
- Record medical history, including history of prostate cancer, diagnosis date, and prior treatments
- Record concomitant medications
- Perform a complete physical examination (*Serious adverse events must be recorded from the time of signed informed consent*)
- Perform and record vital signs and ECOG performance status
- Perform and record standard 12-lead ECG
- Collect blood and urine for clinical laboratory assessments
- If patient is confirmed eligible, randomize patient and schedule Cycle 1 Day 1 visit

8.2 CYCLE 1 DAY 1

- Administer FACT-P and EQ-5D questionnaires
- Review concomitant medications
- Record any serious adverse events
- Perform abbreviated physical examination
- Perform and record vital signs and ECOG performance status
- Collect blood for hematology, blood chemistry, and PSA
- **Administer study drug in clinic**
- Collect blood for PK sample between 0.5 and 4 hours post-dose and record actual time of collection relative to dosing

8.3 DAY 1 OF CYCLES N (+/- 2 DAYS)

- Administer FACT-P and EQ-5D questionnaires
- Record any adverse events
- Record changes to concomitant medications
- Assess study drug compliance
- Perform abbreviated physical examination

- Perform and record vital signs and ECOG performance status
- Collect blood for hematology, blood chemistry, and PSA
- Collect blood for PK sample (*Cycles 2, 3, 6, 12, 18, 24, 36, and yearly thereafter; record time of last two doses of ARN-509, including whether they were taken on a QD or BID regimen*)

8.4 EVERY 16 WEEKS (DAYS 1 OF CYCLES 5, 9, 13, ETC.) (+/- 2 DAYS)

- Collect blood and urine for TSH, fasting lipid panel, testosterone, and urinalysis
- Obtain CT of the chest, abdomen, and pelvis, plus bone scan and submit for BICR to evaluate for disease progression (*tumor scans have +/- 7 days window*; *however, scans should continue on schedule according to Cycle 1, regardless if a +/- 7 days window was used or if there were treatment delays resulting from toxicity)*

8.5 END OF TREATMENT

- Administer FACT-P and EQ-5D questionnaires (*optional if performed within 2 weeks*)
- Record any adverse events (*optional if performed within 1 week*)
- Record changes to concomitant medications (*optional if performed within 1 week*)
- Assess study drug compliance
- Perform abbreviated physical examination (*optional if performed within 1 week*)
- Perform and record vital signs and ECOG performance status (*optional if performed within 1 week*)
- Collect blood and urine for clinical laboratory assessments (*optional if performed within 1 week*)
- Collect blood for PK sample and record time of last two doses of ARN-509 (*optional if performed within 1 week*)
- Obtain CT of the chest, abdomen, and pelvis, plus bone scan and submit for BICR to evaluate for disease progression (*optional if performed within 8 weeks*)

8.6 SAFETY FOLLOW-UP (28 DAYS FOLLOWING THE LAST DOSE OF STUDY DRUG)

- Record any adverse events
- Record changes to concomitant medications

8.7 SURVIVAL FOLLOW-UP

- Obtain survival status, including administration of FACT-P and EQ-5D questionnaires, recording of development of symptomatic progression and initiation of any new systemic anti-cancer therapies (in particular, cytotoxic chemotherapy) (*every 4 months via clinic visit or telephone contact*)

- **In addition, if patients discontinued study treatment prior to documented disease progression**, obtain CT of the chest, abdomen, and pelvis, plus bone scan and submit for BICR to evaluate for disease progression (*every 16 weeks according to schedule*)

9. ADVERSE EVENT REPORTING REQUIREMENTS

9.1 DEFINITIONS

9.1.1 Adverse Event (AE)

An adverse event (AE) is any untoward medical occurrence that develops or worsens in severity after starting the first dose of study drug or any procedure specified in the protocol, even if the event is not considered to be related to the study treatment. Examples of adverse events include but are not limited to:

- Abnormal test findings
- Clinically significant signs and symptoms
- Changes in physical examination findings
- Worsening of signs and symptoms of the malignancy under study. *Disease progression assessed by measurement of malignant lesions on radiographs or other methods should **not** be reported as an adverse event.*
- Signs or symptoms resulting from dose overdose, dependency, withdrawal, abuse, and/or misuse
- Drug interactions
- Exposure in utero (pregnancy)

For laboratory abnormalities, the criteria for determining whether an abnormal test finding should be reported as an adverse event are as follows:

- Test result is associated with accompanying symptoms, and/or
- Test result requires additional diagnostic testing or medical/surgical intervention, and/or
- Test result leads to a change in study dosing outside of protocol-stipulated dose adjustments or discontinuation from the study, significant additional concomitant drug treatment, or other therapy, and/or
- Test result is considered to be an adverse event by the Investigator

9.1.2 Serious Adverse Event (SAE)

A serious adverse event (SAE) is any adverse event, occurring at any dose and regardless of causality that:

- Results in death. *If the malignancy under study has a fatal outcome during the study or within the safety reporting period, the event leading to death should be reported as a Grade 5 SAE; death is an outcome and not the adverse event in itself.*

- Is life-threatening (i.e., immediate risk of death from the reaction as it occurred). *It does **not** include a reaction which hypothetically might have caused death had it occurred in a more severe form.*
- Requires or prolongs inpatient hospitalization (i.e., the event required at least a 24-hour hospitalization or prolonged a hospitalization beyond the expected length of stay). Hospitalization admissions and/or surgical operations scheduled to occur during the study period, but planned prior to study entry are not considered SAEs if the illness or disease existed before the person was enrolled in the trial, provided that it did not deteriorate in an unexpected manner during the trial (e.g., surgery performed earlier than planned)
- Results in persistent or significant disability/incapacity. Disability is defined as a substantial disruption of a person's ability to conduct normal life functions
- Results in a congenital anomaly or birth defect
- Is an important medical event when, based upon appropriate medical judgment, it may jeopardize the participant and require medical or surgical intervention to prevent one of the outcomes listed above. Examples of such medical events include allergic bronchospasm requiring intensive treatment in an emergency room or at home; blood dyscrasias or convulsions that do not result in inpatient hospitalization, or the development of drug dependency or drug abuse

Events **not** considered to be SAEs are hospitalizations for:

- Routine treatment or monitoring of the studied indication, not associated with any deterioration in condition
- Elective or pre-planned treatment for a pre-existing condition that did not worsen
- Emergency outpatient treatment for an event not fulfilling the serious criteria outlined above and not resulting in inpatient admission
- Respite care or social admissions

9.1.3 Expectedness

An adverse event or suspected adverse reaction is considered "unexpected" if it is not listed in the Investigator's Brochure or is not listed at the specificity or severity that has been observed.

Unexpected, as used in this definition, also refers to adverse events or suspected adverse reactions that are mentioned in the Investigator's Brochure as occurring with a class of drugs or as anticipated from the pharmacological properties of the drug, but are not specifically mentioned as occurring with the particular drug under investigation. Such events would be considered unexpected until they have been observed with the drug under investigation.

9.1.4 Attribution

A suspected adverse reaction means any adverse event for which there is reasonable possibility that the study drug caused the adverse event. For the purposes of IND safety

reporting, "reasonable possibility" means there is evidence to suggest a causal relationship between the drug and the adverse event.

The Investigator will assign attribution of the possible association of the event with the study drug using the following definitions:

- **Unrelated to study drug**: The adverse event is *clearly not related* or is *doubtfully related* to the study drug
- **Related to study drug**: The adverse event *may be related,* is *likely related,* or is *clearly related* to the study drug

9.1.5 Severity

Signs or symptoms should be graded and recorded by the Investigator according to the National Cancer Institute's Common Terminology Criteria for Adverse Events (CTCAE) Version 4.0 (Appendix 4). When specific adverse events are not listed in the CTCAE, they are to be graded as mild, moderate, severe, or life-threatening according to the following grades and definitions:

Table 2 AE Severity Grading

Severity (Toxicity Grade)	Description
Grade 1	Mild; asymptomatic or mild symptoms; clinical or diagnostic observations only; intervention not indicated
Grade 2	Moderate; minimal, local or noninvasive intervention indicated; limiting age-appropriate instrumental activities of daily living (ADL)
Grade 3	Severe or medically significant but not immediately life-threatening; hospitalization or prolongation of hospitalization indicated; disabling; limiting self-care ADL
Grade 4	Life-threatening consequences; urgent intervention indicated
Grade 5	Death related to AE

9.1.6 Exposure during Pregnancy

For investigational products and for marketed products, an exposure during pregnancy (also referred to as exposure in utero) occurs if:

- a female becomes, or is found to be, pregnant either while receiving or having been directly exposed to (e.g., environmental exposure) the investigational product, or the female becomes, or is found to be, pregnant after discontinuing and/or being directly exposed to the investigational product (maternal exposure);

- a male has been exposed, either due to treatment or environmental, to the investigational product prior to or around the time of conception and/or is exposed during his partner's pregnancy (paternal exposure).

If any study patient's partner becomes or is found to be pregnant during the patient's treatment with the investigational product, the Investigator must submit this information to Sponsor on a Pregnancy Report Form. In addition, the Investigator must submit information regarding environmental exposure to the investigational product in a pregnant woman (e.g., a nurse reports that she is pregnant and has been exposed to the investigational product) using the Pregnancy Report Form. This must be done irrespective of whether an adverse event has occurred and within 24 business hours of awareness of the pregnancy. The information submitted should include the anticipated date of delivery (see below for information related to an induced termination of pregnancy).

Follow-up is conducted to obtain pregnancy outcome information on all exposure during pregnancy reports with an unknown outcome. The Investigator will follow the pregnancy until completion or until pregnancy termination (i.e., induced abortion) and then notify the Sponsor of the outcome. The investigator will provide this information as a follow-up to the initial Pregnancy Report Form. The reason(s) for an induced abortion should be specified. A report is not created when an ectopic pregnancy report is received since this pregnancy is not usually viable. Rather, a serious adverse event case is created with the event of ectopic pregnancy.

If the outcome of the pregnancy meets the criteria for immediate classification as a serious adverse event (i.e., spontaneous abortion, stillbirth, neonatal death, or congenital anomaly [including that in an aborted fetus, stillbirth or neonatal death]), the Investigator should follow the procedures for reporting serious adverse events.

In the case of a live birth, the "normality" of the newborn can be assessed at the time of birth (i.e., no minimum follow-up period of a presumably normal infant is required before a Pregnancy Report Form can be completed). The "normality" of an aborted fetus can be assessed by gross visual inspection, unless pre-abortion test findings are suggestive of a congenital anomaly.

Additional information about pregnancy outcomes that are classified as serious adverse events follows:

- "Spontaneous abortion" includes miscarriage and missed abortion.
- All neonatal deaths that occur within 1 month of birth should be reported, without regard to causality, as serious adverse events. In addition, any infant death after 1 month that the Investigator assesses as possibly related to the exposure during pregnancy to the investigational product should be reported.

Additional information regarding the exposure during pregnancy may be requested by the Investigator. Further follow-up of birth outcomes will be handled on a case-by-case basis (e.g., follow-up on preterm infants to identify developmental delays). In the case of paternal exposure, the Investigator will provide the study subject with a release of information form to

deliver to his partner. The Investigator must document on the Pregnancy Report Form that the subject was given this letter to provide to his partner.

9.2 REPORTING REQUIREMENTS

All AEs and SAEs whether reported by the patient, discovered during questioning, directly observed, or detected by physical examination, laboratory test or other means, will be recorded in the patient's medical record and on the appropriate study-specific CRFs.

9.2.1 SAE Reporting

For serious adverse events, the reporting period begins from the time the patient provides informed consent, which is obtained prior to the patient's participation in the study, i.e., prior to undergoing any study-related procedure and/or receiving study drug, through and including 28 calendar days after the last dose of study drug.

All SAEs, irrespective of relationship to study treatment, and pregnancies must be reported to the Sponsor within 1 business day of awareness of the event by the Investigator. In particular, if the SAE is fatal or life-threatening, notification must be made immediately, irrespective of the extent of available information. The immediate report should be followed within 1 business day with a full SAE report form. This timeframe also applies to additional new information (follow-up).

SAEs and pregnancies should be reported by facsimile or email as follows:

	Inside North America	Outside North America	Europe and Australia
Facsimile	[redacted]	[redacted]	[redacted]
Email	[redacted]	[redacted]	[redacted]

For urgent SAE-related questions, investigational sites should contact the following:

	Inside North America	Outside North America	Europe and Australia
Telephone	[redacted]	[redacted]	[redacted]
Email	[redacted]	[redacted]	[redacted]

The Investigator is also responsible for notifying the Institutional Review Board/Independent Ethics Committee (IRB/IEC) in accordance with local regulations.

All events should be followed to their resolution, until the Investigator assesses them as stable, irreversible, or until the patient is lost to follow-up, whichever comes first. Any SAEs occurring any time after the reporting period must be promptly reported if a causal relationship to the study drug is suspected.

Reporting Deaths: Regardless of relationship to study drug, all deaths on study should be reported through and including 28 calendar days after the last dose of study. Deaths occurring after the safety follow-up period do not have to be reported as SAEs unless considered related to study drug.

For all SAEs, the Investigator is obligated to pursue and provide information to the Sponsor in accordance with the timeframes for reporting specified above. In addition, an Investigator may be requested to obtain specific additional follow-up information in an expedited fashion. This information may be more detailed than that captured on the AE CRF. In general, this will include a description of the adverse event in sufficient detail to allow for a complete medical assessment of the case and independent determination of possible causality. Information on other possible causes of the event, such as concomitant medications and illnesses must be provided. In the case of a patient death, a summary of available autopsy findings must be submitted as soon as possible to the Sponsor or its designated representative.

9.2.2 Non-Serious AE Reporting

Adverse events should be recorded on the AE CRF from the time the patient has taken at least one dose of study drug through and including 28 calendar days after the last dose of study drug. All events should be followed to their resolution, until the Investigator assesses them as stable, irreversible, or until the patient is lost to follow-up, whichever comes first.

If a patient begins a new systemic anti-cancer therapy, the adverse event reporting period for non-SAEs ends at the time the new treatment is started.

9.2.3 Sponsor Reporting Requirements to Regulatory Authorities

Adverse events reporting, including suspected serious unexpected adverse reactions, will be carried out in accordance with applicable local regulations. The Sponsor or its designee will be responsible for determining whether or not the suspected adverse reaction meets the criteria for expedited reporting.

- The Sponsor or its designee will be responsible for reporting relevant SAEs to the Competent Authority, other applicable regulatory authorities, and participating investigators, in accordance with ICH guidelines, FDA regulations, European Clinical Trials Directive (Directive 2001/20/EC), and/or local regulatory requirements.
- The Sponsor or its designee will be responsible for reporting unexpected fatal or life-threatening events associated with the use of the study drug to the regulatory authorities by telephone or fax within 7 calendar days after being notified of the event.
- The Sponsor or its designee will report other relevant SAEs associated with the use of the study drug to the appropriate regulatory authorities (according to local guidelines) and Investigators by a written safety report within 15 calendar days of notification.

10. END OF TREATMENT

A patient may be discontinued from study treatment at any time if the patient, the Investigator, or the Sponsor feels that it is not in the patient's best interest to continue on study. The following is a list of possible reasons for early discontinuation of study treatment:

- Disease progression *(patients should be highly encouraged to stay on study treatment until there is radiographic confirmation of progression; treatment decisions should not be based on PSA alone)*
- Any episode of seizure
- Any other adverse event that cannot be adequately managed with dose modifications, including dose interruption for up to 28 days
- Protocol violation requiring discontinuation of study treatment
- Patient is not compliant with study procedures
- Lost to follow-up
- Patient withdrawal of consent
- Sponsor request for early termination of study

Data to be collected for the end of treatment visit are described in Section 8.5. Patients will be followed for at least 28 calendar days after the last dose of study drug. If a patient is withdrawn from treatment due to an adverse event, the patient will be followed until the adverse event has resolved or stabilized as per Section 9.2.2.

All patients discontinuing study treatment will enter the survival follow-up period and will be followed for the development of symptomatic progression and initiation of subsequent anti-cancer therapies (in particular, cytotoxic chemotherapy) every 4 months until death, loss of follow-up, or withdrawal of consent, whichever comes first.

11. PROTOCOL VIOLATIONS

A protocol violation occurs when the patient or Investigator fails to adhere to significant protocol requirements affecting the inclusion, exclusion, patient safety and primary endpoint criteria. Protocol violations for this study include, but are not limited to, the following:

- Failure to meet inclusion/exclusion criteria [*no waivers will be granted to meet the eligibility criteria*]
- Use of a prohibited concomitant medication
- Dose modifications that are not within the protocol specifications
- Any other deviation that presents significant risk or safety concerns to the patient

Failure to comply with Good Clinical Practice (GCP) guidelines will also result in a protocol violation. The Sponsor, in consultation with the Investigator, will determine if a protocol violation should result in withdrawal of a patient.

When a protocol violation occurs, it will be discussed with the Investigator and appropriate forms detailing the violation will be generated. This form will be signed by representatives from the Sponsor and the site. A copy of the form will be filed in the site's regulatory binder and in the Sponsor's files.

12. DATA MONITORING COMMITTEE

An independent third-party Data Monitoring Committee (DMC) will be established to ensure the overall integrity and conduct of the study.

The DMC will review the progress of the study and cumulative unblinded safety data on a periodic basis (e.g., a minimum of two face to face review meetings per year) as well as serve as the primary reviewers of the efficacy analysis. Details regarding the timing and content of the primary endpoint review are described in the statistical section below. In addition to the formal face to face meetings, unblinded listings of serious adverse events will be provided to the DMC on a monthly basis.

Following each review meeting, the DMC will recommend to the Sponsor whether to continue the trial unchanged, modify the conduct of the study, or terminate the study early. Rules for early termination, modification and/or continuation of the study, as well as how these recommendations will be made to the Sponsor and Health Authorities will be outlined in a separate DMC Charter.

The DMC will be composed of 3 external members [2 physicians and 1 biostatistician] not associated with the conduct of the study. The Sponsor will also designate an independent biostatistician not affiliated with the project to prepare and provide study data to the DMC. Complete details regarding the composition and governance of the DMC will be outlined in the DMC Charter.

Periodic adverse event data review will also be performed by designated members of the Sponsor's primary study team and will be blinded to treatment assignment with adverse event data from both treatment groups combined. Any safety issues of concern identified by the primary study team that require notification of the DMC will be communicated as described in the DMC Charter.

13. STATISTICAL METHODS AND CONSIDERATIONS

A detailed methodology for summary and statistical analyses of the data collected in this study will be documented in a Statistical Analysis Plan (SAP) that will be dated, version-controlled and maintained by the Sponsor.

13.1 ANALYSIS POPULATIONS

Full Analysis (Intent-to-Treat) Population [ITT]: All eligible patients who are randomized into the study, with study drug assignments designated according to initial randomization, regardless of whether patients receive study drug or receive a different drug from that to which they were randomized to will be included in the analyses of all efficacy and clinical benefit endpoints and patient characteristics.

Safety Analysis Population [SAFETY]: All patients who receive at least one dose of study drug, with treatment assignments designated according to actual study treatment received will be the primary population for evaluating safety and treatment compliance and administration.

- **Patient Report Outcomes Population [PRO]**: Subset of the safety analysis population that has completed at least the baseline assessment (Cycle 1 Day 1) of either FACT-P or EQ-5D questionnaires.
- **Population Pharmacokinetics Populations [PK]**: Subset of the safety analysis population that was randomized to the ARN-509 treatment arm and that has at least one PK sample collected.

13.2 EFFICACY ANALYSES

Efficacy analyses will be performed on the ITT population, incorporating the randomization stratification factors as documented on the CRF, unless otherwise specified.

Analyses of efficacy endpoints which are based on radiographic tumor assessments (MFS, PFS, and TTM) will be based on the results of the blinded independent central review (BICR). Investigator assessments may be used for sensitivity analyses, as described in the Statistical Analysis Plan.

13.2.1 Analysis of Primary Endpoint

The primary endpoint for the study is metastasis-free survival (MFS) which is defined as the time from randomization to first evidence of BICR-confirmed radiographically detectable bone or soft tissue distant metastasis (simply referred to as "metastasis" from this point forward) or death due to any cause (whichever occurs earlier) + 1 day.

MFS data for patients without metastasis or death will be censored on the date of the last tumor assessment (or, if no tumor assessment was performed after the baseline visit, at the date of randomization + 1 day). Additional censoring rules will vary according to whether the analysis is performed for US or ex-US regulatory purposes, as shown below; both results will be provided in the clinical study report.

Scenario	US regulatory guidance	ex-US regulatory guidance[24]
Data from patients who are lost to follow-up or whose disease progression (development of metastasis) or death occurs after 2 or more consecutively missing or unevaluable tumor assessments	Censored on the date of the last tumor assessment that the patient was known to be metastasis-free	Time of progression will be determined using the first date when there is documented evidence of progression or death (whichever occurs earlier) regardless of missed or unevaluable tumor assessments
Patients that receive new systemic anti-cancer therapy prior to documented disease progression (development of metastasis) or death	Censored on the date of the last tumor assessment prior to the start of the new systemic anti-cancer therapy	Time of progression will be determined using the first date when there is documented evidence of progression or death (whichever occurs earlier) regardless of change of therapy

The primary efficacy analysis will be completed when approximately [redacted] MFS events have occurred. The primary analysis will compare the MFS distributions in the two treatment arms using a two-sided log-rank test, stratified by PSADT (> 6 months vs. ≤ 6 months), the use of a bone-sparing agent (Yes vs. No), and the presence of loco-regional disease (N0 vs. N1) at the 0.05 significance level. The unstratified log-rank test will be provided as a sensitivity analysis. A complete list of sensitivity analyses is provided in the Statistical Analysis Plan.

Kaplan-Meier methods will be used to estimate median MFS for each treatment arm. Cox proportional-hazard models, including the same factors as above, will be used to estimate the hazard ratio and its 95% confidence interval (CI).

13.2.2 Analysis of Key Secondary Endpoint

Overall survival (OS) is the key secondary endpoint. In order to control the overall level of significance, the analysis of OS will be carried out only if the analysis of MFS is statistically significant.

OS will be defined as the time from randomization to the date of death due to any cause + 1 day. Patients who are alive at the time of the analysis will be censored on the last known date that they were alive. In addition, the following censoring rules will apply:

Scenario	Date of Censoring
Patients with no post-baseline information	Censored on the date of randomization + 1 day
Patients who are lost to follow-up or who withdraw consent for further follow-up	Censored on the last known date that they were alive
Sensitivity Analysis: Patients that receive new systemic anti-cancer therapy	*Censored on the day before the start date of the new systemic anti-cancer therapy*

Data analysis will be similar to that for the primary endpoint (stratified two-sided log-rank test). The unstratified log-rank test will be provided as a sensitivity analysis.

The final analysis of OS will occur after approximately [redacted] deaths have occurred. In order to adjust for a single interim analysis of OS, which will occur at the time of the analysis for MFS, the levels of significance for the interim and final analyses of OS will be [redacted] and [redacted], respectively.

As additional analyses, the 1- and 2-year survival rates will be estimated using the Kaplan-Meier method.

13.2.3 Analyses of Other Secondary Endpoints

Following the primary analysis of MFS (and interim analysis of OS), the analyses of the other secondary endpoints will be prioritized in the order listed below such that if one of the endpoints does not reach statistical significance (two-sided $p < 0.05$), the results for all other secondary endpoints of a lower rank will be considered to be exploratory:

- Time to symptomatic progression
- Time to initiation of cytotoxic chemotherapy
- Radiographic progression-free survival
- Time to metastasis

Time-to-event-based secondary analyses (time to symptomatic progression, time to initiation of cytotoxic chemotherapy, radiographic progression-free survival, and time to metastasis) will be performed using a two-sided log-rank test, stratified by PSADT (> 6 months vs. ≤ 6 months), the use of a bone-sparing agent (Yes vs. No), and the presence of loco-regional disease (N0 vs. N1) at the 0.05 significance level. Unstratified log-rank tests will also be provided as sensitivity analyses.

Kaplan-Meier methods will be used to estimate medians for each treatment arm. Cox proportional-hazard models, including the same factors as above, will be used to estimate the hazard ratio and its 95% confidence interval (CI).

13.2.3.1 Time to Symptomatic Progression

Time to symptomatic progression will be defined as the time from randomization to documentation in the CRF of any of the following (whichever occurs earlier) + 1 day:

- Development of a skeletal-related event (SRE): pathologic fracture, spinal cord compression, or need for surgical intervention or radiation therapy to the bone.
- Pain progression or worsening of disease-related symptoms requiring initiation of a new systemic anti-cancer therapy.
- Development of clinically significant symptoms due to loco-regional tumor progression requiring surgical intervention or radiation therapy.

Adverse event, concomitant medication, concomitant treatment, or survival follow-up CRFs will be the source of these findings.

Time to symptomatic progression for patients who do not experience any of the events described above will be censored on the date on which they were last known to be event-free.

13.2.3.2 Time to Initiation of Cytotoxic Chemotherapy

Time to initiation of cytotoxic chemotherapy will be defined as the time from randomization to documentation in the CRF of a new cytotoxic chemotherapy being administered to the patient (e.g., survival follow-up CRF) + 1 day.

Time to initiation of cytotoxic chemotherapy for patients who do not start cytotoxic chemotherapy will be censored on the date of last contact.

13.2.3.3 Radiographic Progression-Free Survival

In order to capture loco-regional disease progression, a secondary endpoint of progression-free survival (PFS) will be assessed and defined as the time from randomization to first documentation of BICR-confirmed radiographic progressive disease or death due to any cause (whichever occurs first) + 1 day.

Progressive disease (PD) will be determined based on RECIST v1.1, and further defined as follows:

- For patients with at least one measurable lesion, PD will be defined as at least a 20% increase in the sum of diameters of target lesions taking as reference the smallest sum on study (this includes the baseline sum if that is the smallest on study). In addition to the relative increase of 20%, the sum must also demonstrate an absolute increase of at least 5 mm. Furthermore, the appearance of one or more new lesions is also considered progression.
- For patients with only non-measurable disease observed on CT or MRI scans, unequivocal progression (representative of overall disease status change) or the appearance of one or more new lesions will be considered progression. For new bone lesions detected on bone scans, a second imaging modality (e.g., CT or MRI) will be required to confirm progression.

Radiographic PFS data for patients without loco-regional disease will be censored on the date of the last tumor assessment (or, if no tumor assessment was performed after the baseline visit, at the date of randomization + 1 day). Additional censoring rules will vary according to whether the analysis is performed for US or ex-US regulatory purposes, as shown below; both results will be provided in the clinical study report.

**Scenario**	**US regulatory guidance**	**ex-US regulatory guidance**
Data from patients who are lost to follow-up or whose disease progression or death occurs after 2 or more consecutively missing or unevaluable tumor assessments	Censored on the date of the last tumor assessment that the patient was known to be progression-free	Time of progression will be determined using the first date when there is documented evidence of progression or death (whichever occurs earlier) regardless of missed or unevaluable tumor assessments
Patients that receive new systemic anti-cancer therapy prior to documented disease progression or death	Censored on the date of the last tumor assessment prior to the start of the new systemic anti-cancer therapy	Time of progression will be determined using the first date when there is documented evidence of progression or death (whichever occurs earlier) regardless of change of therapy

13.2.3.4 Time to Metastasis

Time to Metastasis (TTM) will be defined as the time from randomization to first evidence of BICR-confirmed radiographically detectable bone or soft tissue distant metastasis (simply referred to as “metastasis” from this point forward) + 1 day.

TTM data for patients without metastasis will be censored on the date of the last tumor assessment (or, if no tumor assessment was performed after the baseline visit, at the date of randomization + 1 day). Additional censoring rules will vary according to whether the analysis is performed for US or ex-US regulatory purposes, as shown below; both results will be provided in the clinical study report.

Scenario	US regulatory guidance	ex-US regulatory guidance
Data from patients who are lost to follow-up or whose disease progression (development of metastasis) occurs after 2 or more consecutively missing or unevaluable tumor assessments	Censored on the date of the last tumor assessment that the patient was known to be metastasis-free	Time of progression will be determined using the first date when there is documented evidence of progression regardless of missed or unevaluable tumor assessments
Patients that receive new systemic anti-cancer therapy prior to documented disease progression (development of metastasis)	Censored on the date of the last tumor assessment prior to the start of the new systemic anti-cancer therapy	Time of progression will be determined using the first date when there is documented evidence of progression regardless of change of therapy

13.3 SAFETY EVALUATIONS

The SAFETY population will be the primary population for evaluating safety.

13.3.1 Analysis of Adverse Events

Adverse events (AEs) will be graded according to the NCI Common Terminology Criteria for Adverse Events (CTCAE) Version 4.0 and coded to preferred term and system organ class (SOC) using the most recent version of MedDRA.

All AEs reported during the AE reporting period (inclusive of the 28-day post last dose of study drug period) will be considered as treatment-emergent adverse events and will be summarized by treatment arm as treated.

For each treatment arm, adverse event incidence rates will be summarized with frequency and percentage by MedDRA SOC and preferred term, with all patients treated in that treatment arm as the denominator, unless otherwise specified. In addition, AE incidence rates will also be summarized by severity and relationship to study drug. Treatment-related AEs are those judged by the Investigator to be at least possibly related to the blinded study drug. AEs with missing severity or relationship to study drug will be classified as severe and treatment-related, respectively. Patients with multiple occurrences of events will only be counted once at the maximum severity to study drug for each preferred team, SOC, and overall. Deaths that occur within 28 days after the last dose of study drug are defined as on-study deaths.

Summary tables and individual patient listings will be prepared as per the Statistical Analysis Plan.

13.3.2 Analysis of Clinical Laboratory Results

Only data collected by the central laboratory will be summarized. Local laboratory data collected for the purposes of planning treatment administration, dose modification, or monitoring adverse events, will not be summarized.

Normal ranges will be used to identify values that are outside the normal ranges and abnormal laboratory results will be graded according to the NCI CTCAE Version 4.0. Descriptive statistics will be provided for each test result and for the change from baseline by visit.

A shift summary of baseline grade by maximum post-baseline CTCAE grade will be presented, as appropriate. For each laboratory parameter, the baseline laboratory value will be defined as the last laboratory value collected on or prior to the first dose of study drug.

Patients who develop toxicities of Grade ≥ 3 will be summarized. Laboratory test results not having CTCAE grade will also be summarized. Parameters that have criteria available for both low and high values (e.g., hypercalcaemia vs. hypocalcaemia) will be summarized for both criteria. Patients will only be counted once for each criterion.

13.3.3 PSA

PSA kinetics (e.g., 12-week PSA response and time to PSA progression) will be assessed at the time of the primary analysis of MFS according to the Prostate Cancer Clinical Trials Working Group (PCWG2) recommendations.[25]

Summary tables and waterfall plots describing change in PSA relative to baseline will be reported at 12 weeks (or earlier for those who discontinue study treatment prior to 12 weeks), and separately, the maximum change at any time on study will also be reported for each patient using summary tables and waterfall plots.

The time to PSA progression will be calculated as the time from randomization to the time when the criteria for PSA progression according to PCWG2 are met + 1 day. Kaplan-Meier methods will be used to estimate the median time to PSA progression and 95% confidence intervals for each treatment arm.

13.3.4 Analysis of Vital Signs

Each vital sign (temperature, blood pressure (systolic and diastolic), respiration rate, and heart rate) and respective change from baseline will be summarized and presented by treatment arm and study visit. Patients with clinically significant abnormalities in vital signs as compared to baseline will be listed.

13.3.5 Concomitant Medications/Treatments

All medications and/or treatments received during the protocol treatment period will be considered as concomitant medications and/or concomitant treatments and will be coded by

WHO medical dictionary; patients who received concomitant medications and/or treatments will be listed.

13.4 OTHER EVALUATIONS

13.4.1 Health-Related Quality of Life and Prostate Cancer-Specific Symptoms

The FACT-P and EQ-5D data will be scored and handled as recommended in their respective User's manuals, including handling of missing data both within the subscales and overall. All the analysis for FACT-P and EQ-5D data will be performed in the PRO population.

Health-related quality of life response will be defined as a 16-point or higher improvement in the global FACT-P score as compared to baseline. The proportion of patients with a quality of life response will be summarized by treatment arm. The response rates between treatment arms will be compared using a Mantel-Haenszel test, stratified by PSADT (> 6 months vs. ≤ 6 months), the use of a bone-sparing agent (Yes vs. No), and the presence of loco-regional disease (N0 vs. N1) at a two-sided 0.05 significance level.

The EQ-5D data will be summarized descriptively by treatment group and study visit.

13.4.2 Population Pharmacokinetics (Pop PK)

Population PK analysis will utilize patient covariates to identify sub-populations where possible. The relationship of exposure to ARN-509 and its metabolites (ARN000308 and ARN000066) to measures of efficacy and adverse events will also be modeled to the extent possible. The PK population will be the primary population used for these analyses.

Descriptive statistics, including mean and median values will be summarized. Concentrations below the lower limit of quantification of the assay will be excluded or assigned a numeric value based on the lower reporting limit of the assay. Plasma levels of ARN-509 and its metabolites ARN000308 and ARN000066 will be listed by patient and summarized descriptively (mean, standard deviation, percent coefficient of variation, minimum, maximum). Individual and mean concentration versus time plots will be presented on both linear and logarithmic scales where possible. Non-compartmental, compartmental, and population analysis methods will be utilized as applicable.

Samples may also be used for metabolite identification, but the results of such analyses will be used for exploratory purposes and will not be included in the clinical study report. Selected samples may also be analyzed for concentrations of concurrent medications, and these data may be used to assess drug-drug interactions. Blood samples for PK will be collected from all patients due to the blinded nature of the study, but samples collected from placebo patients will not be analyzed in most cases.

13.4.3 Assessment of Ventricular Repolarization

The assessment of ventricular repolarization will be a sub-study conducted in a subset of patients from selected clinical sites and analyzed by an independent cardiac safety laboratory (Appendix 8).

13.5 INTERIM ANALYSIS

A single interim efficacy analysis of the key secondary endpoint (OS) will be carried out at the time of the efficacy analysis for MFS. At that time, approximately [redacted] OS events will be observed, representing [redacted] of the total number of events [redacted] at the final OS analysis. Based on this information fraction [redacted] and using the Lan-DeMets method for group sequential trials with O'Brien-Fleming boundaries, the levels of significance for the interim and final analyses of OS will be [redacted] and [redacted], respectively.

13.6 DETERMINATION OF SAMPLE SIZE

The primary efficacy analysis will be event-driven and will take place when approximately [redacted] MFS events have occurred. The study provides 90% power to detect a [redacted] reduction in the risk of developing metastases (HR = [redacted]) for patients receiving ARN-509, with a 2-sided α of 0.05. Based on an assumed median MFS of 25 months in the placebo arm, this treatment effect represents an increase in the median MFS of approximately [redacted] months (from 25 months to [redacted] months). Assuming an accrual period of [redacted] months (with [redacted] of the patients accrued in the second year), approximately 1200 patients will need to be enrolled.

The study is also sized to provide 85% power to detect a [redacted] reduction (HR = [redacted]) in the risk of death for patients receiving ARN-509, based on an assumed median OS of [redacted] months in the placebo arm. This treatment effect represents an increase in the median OS of approximately [redacted] months (from [redacted] to [redacted] months). The final analysis of OS will occur after approximately [redacted] deaths have occurred.

The total study duration (including the time it takes to reach the secondary endpoint of OS) will be approximately 59 months.

14. DATA COLLECTION, RETENTION AND MONITORING

14.1 DATA COLLECTION INSTRUMENTS

The Investigator will prepare and maintain adequate and accurate source documents designed to record all observations and other pertinent data for each patient treated with the study drug.

Study personnel at each site will enter data from source documents corresponding to a patient's visit into the protocol-specific electronic case report form (eCRF) when the information corresponding to that visit is available. Patients will not be identified by name in the study database or on any study documents to be collected by the Sponsor (or designee), but will be identified by a site number, patient number and initials.

If a correction is required for an eCRF, the time and date stamps track the person entering or updating eCRF data and creates an electronic audit trail.

The Investigator is responsible for reviewing all information collected on patients enrolled in this study for completeness and accuracy. A copy of the eCRF will remain at the Investigator's site at the completion of the study.

14.2 DATA MANAGEMENT PROCEDURES

The data will be entered into a validated database. The Sponsor-designated data management group will be responsible for data processing, in accordance with procedural documentation. Database lock will occur once quality assurance procedures have been completed.

All procedures for the handling and analysis of data will be conducted using good computing practices meeting FDA guidelines for the handling and analysis of data for clinical trials.

14.3 DATA QUALITY CONTROL AND REPORTING

After data have been entered into the study database, a system of computerized data validation checks will be implemented and applied to the database on a regular basis. Queries are entered, tracked, and resolved through the electronic data capture (EDC) system directly. The study database will be updated in accordance with the resolved queries. All changes to the study database will be documented.

14.4 ARCHIVAL OF DATA

The database is safeguarded against unauthorized access by established security procedures; appropriate backup copies of the database and related software files will be maintained. Databases are backed up by the database administrator in conjunction with any updates or changes to the database.

At critical junctures of the protocol (e.g., production of interim and final reports), data for analysis is locked and cleaned per established procedures.

14.5 AVAILABILITY AND RETENTION OF INVESTIGATIONAL RECORDS

To enable evaluations and/or audits from regulatory authorities or the Sponsor, the Investigator agrees to keep records, including the identity of all participating subjects (sufficient information to link records, e.g., CRFs and hospital records), all original signed informed consent forms, copies of all CRFs, safety reporting forms, source documents, and detailed records of treatment disposition, and adequate documentation of relevant correspondence (e.g., letters, meeting minutes, telephone calls reports). The records should be retained by the Investigator according to ICH, local regulations, or as specified in the Clinical Trial Agreement, whichever is longer, but at a minimum, all study documentation must be retained for 2 years after the last marketing application approval in an ICH region or after at least 2 years have elapsed since formal discontinuation of clinical development of ARN-509.

If the Investigator becomes unable for any reason to continue to retain study records for the required period (e.g., retirement, relocation), the Sponsor should be prospectively notified. The study records must be transferred to a designee acceptable to the Sponsor, such as another investigator, another institution, or to the Sponsor itself. The Investigator must obtain the Sponsor's written permission before disposing of any records, even if retention requirements have been met.

14.6 MONITORING

Monitoring visits will be conducted by representatives of the Sponsor according to the US CFR Title 21 Parts 50, 56, and 312 and ICH Guidelines for GCP (E6). By signing this protocol, the Investigator grants permission to the Sponsor (or designee), and appropriate regulatory authorities to conduct on-site monitoring and/or auditing of all appropriate study documentation.

14.7 PATIENT CONFIDENTIALITY

In order to maintain patient confidentiality, only a site number, patient number and patient initials will identify all study patients on CRFs and other documentation submitted to the Sponsor. Additional patient confidentiality issues (if applicable) are covered in the Clinical Trial Agreement.

15. ADMINISTRATIVE, ETHICAL, REGULATORY CONSIDERATIONS

The study will be conducted according in accordance with the US FDA regulations, the International Conference on Harmonisation (ICH) E6 Guidelines for GCP, and applicable local, state, and federal laws.

To maintain confidentiality, all laboratory specimens, evaluation forms, reports and other records will be identified by a coded number and initials only. All study records will be kept in a locked file cabinet and code sheets linking a patient's name to a patient identification number will be stored separately in another locked file cabinet. Clinical information will not be released without written permission of the patient, except as necessary for monitoring by the FDA. The Investigator must also comply with all applicable privacy regulations (e.g., Health Insurance Portability and Accountability Act of 1996, EU Data Protection Directive 95/46/EC).

15.1 PROTOCOL AMENDMENTS

Any amendment to the protocol will be written by the Sponsor. Protocol amendments cannot be implemented without prior written IRB/IEC approval except as necessary to eliminate immediate safety hazards to patients. A protocol amendment intended to eliminate an apparent immediate hazard to patients may be implemented immediately, provided the IRB/IECs are notified within five working days.

15.2 INSTITUTIONAL REVIEW BOARDS AND INDEPENDENT ETHICS COMMITTEES

The protocol, Investigator's Brochure, the consent forms, any information to be given to the patient (including patient recruitment materials) and relevant supporting information must be submitted to the IRB/IEC by the Investigator for review and approval before the study is initiated. Any member of the IRB/IEC who is directly affiliated with this study as an Investigator or as site personnel must abstain from the IRB/IEC vote on the approval of the protocol. The IRB/IECs unconditional approval statement will be transmitted by the Investigator to the Sponsor (or designee) prior to the shipment of study supplies to the site. This approval must refer to the study by exact protocol title and number and should identify the documents reviewed and the date of review.

Protocol and/or informed consent modifications or changes may not be initiated without prior written IRB/IEC approval except when necessary to eliminate immediate hazards to the patients or when the change(s) involves only logistical or administrative aspects of the study. Such modifications will be submitted to the IRB/IEC and written verification that the modification was submitted and subsequently approved should be obtained.

Investigators are required to promptly report to their respective IRB/IEC all unanticipated problems involving risk to human patients. Some IRBs/IECs may want prompt notification of all SAEs, whereas others require notification only about events that are serious, assessed to be related to study treatment, and are unexpected. Investigators may receive written IND safety reports or other safety-related communications from the Sponsor. Investigators are responsible for ensuring that such reports are reviewed and processed in accordance with

regulatory requirements and with the policies and procedures established by their IRB/IEC and archived in the site's study file.

Finally, the Investigator will keep the IRB/IEC informed as to the progress of the study, revisions to documents originally submitted for review, annual updates and/or request for re-approvals, and when the study has been completed.

15.3 INFORMED CONSENT FORM

Informed consent will be obtained in accordance with the ICH GCP, US Code of Federal Regulations for Protection of Human Subjects (21 CFR 50.25[a, b], CFR 50.27, and CFR Part 56, Subpart A), the Health Insurance Portability and Accountability Act (HIPAA, if applicable), and local regulations.

The Sponsor's master Informed Consent Form will be provided to each site. Sponsor or its designee must review and approve any proposed deviations from the master ICF or any alternate consent forms proposed by the site before IRB/IEC submission. Patients must be re-consented to the most current version of the consent forms during their participation in the study. The final IRB/IEC-approved consent forms must be provided to Sponsor for regulatory purposes.

The consent forms must be signed by the patient or the patient's legal representative before his participation in the study. The case history for each patient shall document the informed consent process and that written informed consent was obtained prior to participation in the study. A copy of each signed consent form must be provided to the patient or the patient's legal representative. If applicable, it will be provided in a certified translation of the local language.

All signed and dated consent forms must remain in each patient's study file and must be available for verification by study monitors at any time.

The Informed Consent Form should be revised whenever there are changes to procedures outlined in the informed consent or when new information becomes available that may affect the willingness of the patient to participate.

For any updated or revised consent forms, the case history for each patient shall document the informed consent process and that written informed consent was obtained for the updated/revised consent form for continued participation in the study. The final revised IRB/IEC-approved Informed Consent Form must be provided to Sponsor for regulatory purposes.

15.4 REPORTING OF SAFETY ISSUES AND SERIOUS BREACHES OF THE PROTOCOL OR ICH GCP

In the event of any prohibition or restriction imposed (i.e., clinical hold) by an applicable Competent Authority in any area of the World, or if the Investigator is aware of any new information which might influence the evaluation of the benefits and risks of the investigational product, the Sponsor should be informed immediately.

In addition, the Investigator will inform the Sponsor immediately of any urgent safety measures taken by the Investigator to protect the study subjects against any immediate hazard, and of any serious breaches of this protocol or of ICH GCP that the Investigator becomes aware of.

15.5 END OF TRIAL IN ALL PARTICIPATING COUNTRIES

Patients will remain on study until disease progression, development of unacceptable toxicity, or withdrawal of consent.

With an estimated accrual duration of 24 months, it is assumed that patients are expected to be followed for a minimum of approximately [redacted] months beyond Last Patient In (LPI) for the primary endpoint of MFS, to approximately [redacted] months beyond LPI for the key secondary endpoint of OS. This corresponds to total projected study duration of 59 months.

If the study is not terminated beforehand per the recommendation of the DMC, the end of trial in all participating countries will be defined as the time at which the secondary endpoint of OS has been met. At that time, the protocol may be amended to minimize the number of protocol assessments to only collection of safety data for those patients remaining on study.

15.6 SPONSOR DISCONTINUATION CRITERIA

Premature termination of this study may occur because of a regulatory authority decision, change in opinion of the IRB/IEC, drug safety problems, or at the discretion of the Sponsor. In addition, the Sponsor retains the right to discontinue development of ARN-509 at any time.

If a study is prematurely terminated or discontinued, the Sponsor will promptly notify the Investigator. After notification, the Investigator must notify the respective IRB/IEC, and contact all participating subjects and the hospital pharmacy (if applicable) within a 4-week time period. As directed by the Sponsor, all study materials must be collected and all CRFs completed to the greatest extent possible.

15.7 PUBLICATIONS

Publication of study results is discussed in the Clinical Trial Agreement. Details regarding production of manuscripts and conference presentations will adhere to the International Committee of Medical Journal Editors (ICMJE) requirements for authorship and contributorship.

http://www.icmje.org/ethical_1author.html

16. REFERENCES

1. Jemal A, Bray F, Center MM, et al. Global cancer statistics. Ca Cancer J Clin 2011; 61:69-90.

2. Stephenson AJ, Kattan MW, Eastham JA, et al. Defining biochemical recurrence of prostate cancer after radical prostatectomy: a proposal for a standardized definition. J Clin Oncol 2005; 23:8253-61.

3. Scher HI, Sawyers CL. Biology of progressive, castration-resistant prostate cancer: directed therapies targeting the androgen-receptor signaling axis. J Clin Oncol 2005; 23:8253-61.

4. Chen CD, Welsbie DS, Tran C, et al. Molecular determinants of resistance to antiandrogen therapy. Nat Med 2004; 10:33-9.

5. Kelly WK, Slovin S, Scher HI. Steroid hormone withdrawal syndromes: pathophysiology and clinical significance. Urol Clin North Am 1997; 24:421-33.

6. Clegg N, Wongvipat J, Joseph J, et al. Discovery and development of ARN-509, a novel anti-androgen for the treatment of prostate cancer. Cancer Res 2012; 72:1-10.

7. Foster WR, Car BD, Shi H, et al. Drug safety is a barrier to the discovery and development of new androgen receptor antagonists. Prostate 2011; 71:480-8.

8. Rathkopf D, Liu G, Carducci MA, et al. Phase I dose-escalation study of the novel antiandrogen BMD-641988 in patients with castration-resistant prostate cancer. Clin Cancer Res 2011; 17:880-7.

9. Rathkopf D, Danila DC, Morris MJ, et al. A Phase I study of the androgen signaling inhibitor ARN-509 in patients with metastatic castration-resistant prostate cancer (mCRPC). ASCO Meeting Abstracts 2012; 30:TPS4697.

10. Freedland SJ, Humphreys EB, Mangold LA, et al. Death in patients with recurrent prostate cancer after radical prostatectomy: prostate-specific antigen doubling time subgroups and their associated contributions to all-cause mortality. J Clin Oncol 2007; 25:1765-71.

11. Smith MR, Kabbinavar F, Saad F, et al. Natural history of rising serum prostate-specific antigen in men with castrate nonmetastatic prostate cancer. J Clin Oncol 2005; 23:2918-25.

12. Smith MR, Cook R, Lee KA, Nelson JB. Disease and host characteristics as predictors of time to first bone metastasis and death in men with progressive castration-resistant nonmetastatic prostate cancer. Cancer 2011; 117:2077-85.

13. National Comprehensive Cancer Network (NCCN) Practice Guidelines in Oncology – v.1.2010.

14. Pinover WH, Horwitz EM, Hanlon AL et al. Validation of a Treatment Policy for Patients with Prostate Specific Antigen Failure after Three-Dimensional Conformal Prostate Radiation Therapy. Cancer 2003; 97:1127-33.

15. Smith MR, Saad F, Coleman R, et al. Denosumab and bone-metastasis-free survival in men with castration-resistant prostate cancer: results of a phase 3, randomised, placebo-controlled trial. Lancet 2012; 379:39-46.

16. Norgaard M, Jensen AO, Jacobsen JB et al. Skeletal related events, bone metastasis and survival of prostate cancer: a population-based cohort study in Denmark (1999 to 2007). J Urol 2010; 184:162-67.

17. Sathiakumar N, Delzell E, Morrisey MA et al. Mortality following bone metastasis and skeletal-related events among men with prostate cancer: a population-based analysis of U.S. Medicare beneficiaries, 1999-2006. Prostate Cancer Prostat Dis 2011; 14:177-83.

18. Freedland SJ, Humphreys EB, Mangold LA et al. Risk of prostate cancer-specific mortality following biochemical recurrence after radical prostatectomy. JAMA 2005; 294:433-9.

19. Ray ME, Bae K, Hussain MH et al. Potential surrogate endpoints for prostate cancer survival: Analysis of a Phase III randomized trial. J Natl Cancer Inst 2009; 101:228-236.

20. Pound CR, Partin AW, Eisenberger MA, et al. Natural history of progression after PSA elevation following radical prostatectomy. JAMA 1999; 281:1591-7.

21. Eisenhauer EA, Therasse P, Bogaerts J, et al. New response evaluation criteria in solid tumors: revised RECIST guideline (version 1.1). Eur J Cancer 2009; 45:228-47.

22. Esper P, Mo F, Chodak G, et al. Measuring quality of life in men with prostate cancer using the Functional Assessment of Cancer Therapy-Prostate (FACT-P) instrument. Urology 1997; 50(6):920-8.

23. The EuroQol Group. EuroQol--a new facility for the measurement of health-related quality of life. Health Policy 1990; 16:199-208.

24. Appendix 1 to the guideline on the evaluation of anticancer medicinal products in man: Methodological consideration for using progression-free survival (PFS) or disease-free survival (DFS) in confirmatory trials. European Medicines Agency 2011; Doc. Ref. EMA/CHMP/27994/2008.

25. Scher HI, Halabi S, Tannock I, et al. Design and end points of clinical trials for patients with progressive prostate cancer and castrate levels of testosterone: recommendations of the prostate cancer clinical trials working group. J Clin Oncol 2008; 26:1148-59.

17. APPENDICES

Appendix 1	Schedule of Activities
Appendix 2	Required Laboratory Tests
Appendix 3	ECOG Performance Status
Appendix 4	National Cancer Institute (NCI) Common Terminology Criteria for Adverse Events (CTCAE)
Appendix 5	Prohibited or Restricted Medications or Supplements While On Study
Appendix 6	FACT-P Questionnaire
Appendix 7	EQ-5D
Appendix 8	Ventricular Repolarization Sub-Study at Selected Sites

APPENDIX 1: SCHEDULE OF ACTIVITIES

	Screening	One Cycle = 4 weeks (28 days)		Post- treatment [1]		
**Activities and Forms to be Completed**	**≤ 28 Days Prior to Dosing**	**Cycle 1 Day 1**	**Cycles n Day 1 (+/-2 days)**	**End of treatment [2]**	**Safety Follow-Up [3]**	**Survival Follow-Up [1]**
**Baseline Documentation**						
Informed Consent	X					
Medical/Oncological History [4]	X					
Physical Examination [5]	X	X	X	X		
**Laboratory Studies**						
Hematology [6]	X	X	X	X		
Blood Chemistry [6]	X	X	X	X		
PSA [6]	X	X	X	X		
Testosterone [7]	X		Every 16 weeks	X		
Thyroid-Stimulating Hormone [7]	X		Every 16 weeks	X		
Fasting Lipid Panel [7]	X		Every 16 weeks	X		
Urinalysis [7]	X		Every 16 weeks	X		
12-lead ECG [8]	X					
Study Randomization [9]	X					
ARN-509/Matched Placebo Administration [10]		X→	→			
**Tumor Assessments (+/- 7 days)**						
CT brain [11]	X					
CT chest, abdomen, and pelvis [12]	X		Every 16 weeks	X		**Every 16 weeks if needed**
Bone scan [12]	X		Every 16 weeks	X		
**Other Clinical Assessments**						
ECOG, Body Weight, and Vital Signs	X	X	X	X		
Adverse Events [13]	X	X	X	X	**28 days post last dose**	
Concomitant Medications/Treatments [14]	X	X	X	X		
Study Drug Compliance [15]			X	X		
Post-Study Survival Status [16]						**Every 4 months**
PK sample [17]		Between 0.5 and 4 hours post-dose	C2, C3, C6, C12, C18, C24, C36, yearly thereafter	X		
**Patient Reported Outcomes**						
FACT-P and EQ-5D [18]		X	X	X		**Every 4 months**

Footnotes

1.	**Post-Treatment:** Patients discontinuing study treatment will enter the survival follow-up period where they will be followed every 4 months and continue to have scheduled disease assessments (if they discontinued prior to documented radiographic progression); they will be followed for the development of symptomatic progression and initiation of subsequent anti-cancer therapies (in particular, cytotoxic chemotherapy) until death, loss of follow-up, or withdrawal of consent, whichever comes first.
2.	**End of treatment:** These assessments do not need to be completed if they have been performed within 1 week of study withdrawal (within the last 8 weeks for tumor assessments and 2 weeks for patient reported outcomes, respectively).
3.	**Safety Follow-Up:** Patients should be evaluated for safety up to 28 calendar days after the last dose of study drug. Adverse events should be followed up until all serious or study drug-related events have resolved or are determined to be “chronic” or “stable”, whichever is later.
4.	**Medical/Oncological History:** Includes oncologic history, demographics, history of other disease processes (active or resolved) and concomitant illnesses.
5.	**Physical Examination:** At Screening, a complete physical examination of major body systems, including known and suspected sites of disease, should be performed. During subsequent visits, abbreviated physical exams will be sufficient.
6.	**Samples for Hematology, Blood Chemistry, and PSA:** All laboratory assessments will be performed by a central laboratory. PSA results will be kept blinded until the analysis of the primary endpoint. Sites may perform additional local hematology and/or blood chemistry assays for the purposes of planning treatment administration, dose modification, or monitoring adverse events.
7.	**Samples for urinalysis, TSH, fasting lipid panel, and testosterone:** All laboratory assessments will be performed by a central laboratory every 16 weeks, at the time of tumor assessments. Sites may perform additional local assays for the purposes of planning treatment administration, dose modification, or monitoring adverse events.
8.	**ECG:** A standard 12-lead ECG will be collected at Screening and as clinically indicated.
9.	**Study Randomization:** Patient number and treatment assignment will be obtained via centralized randomization through the IVRS. PSADT will be calculated by the IVRS to ensure correct patient stratification.
10.	**ARN-509/Matched Placebo:** Patients will receive oral daily ARN-509 or matched placebo continuously, starting on Cycle 1 Day 1. One cycle consists of 28 days.
11.	**Brain Imaging:** CT (or MRI, if use of contrast agent is contraindicated) scan of the brain will be performed at Screening and **submitted for blinded independent central review to confirm patient eligibility** (absence of brain metastasis).
12.	**Tumor Imaging:** CT (or MRI, if use of contrast agent is contraindicated) scans of chest, abdomen and pelvis plus bone scan will be performed to assess disease status at Screening, every 16 weeks from the start of study drug independent of cycle length or whenever disease progression is suspected, and at the end of treatment. Note that tumor imaging should continue on this calendar schedule regardless of any delays in dosing. **All scans will be submitted for blinded independent central review to assess for disease progression on study.**
13.	**Adverse Events:** Patients must be followed for adverse events from the first day of study treatment until at least 28 days after the last dose of study treatment, or until all serious or study drug-related toxicities have resolved or are determined to be “chronic” or “stable”, whichever is later. **SAEs will be collected from the time of signed patient informed consent.**
14.	**Concomitant Medications/Treatments:** Concomitant medications and/or treatments will be recorded during the 28-day Screening period (prior to the start of study treatment), during the study, and up to 28 days post the last dose of study treatment.
15.	**Study Drug Compliance:** ARN-509 and placebo bottle(s) including any unused capsules will be returned at the beginning of every cycle starting at Cycle 2 Day 1 and at end of treatment for drug accountability.
16.	**Post-Study Survival Status:** Follow-up survival information will be collected by the site via clinic visit or telephone contact every 4 months until death, loss of follow-up, or withdrawal of consent, whichever comes first.
17.	**PK Samples for Population PK Analyses**: Plasma samples will be collected on Day 1 of Cycles 1 (between 0.5 and 4 hours post-dose), 2, 3, 6, 12, 18, 24, 36, yearly thereafter, and at the end of treatment.
18.	**FACT-P and EQ-5D questionnaires:** Patients will complete the FACT-P and EQ-5D at the clinic **PRIOR to any other clinical activity** on every Day 1 of each cycle, starting with Cycle 1, at the end of treatment, and during survival follow-up.

APPENDIX 2: REQUIRED LABORATORY TESTS

Hematology	Chemistry	Other
Hemoglobin<br>Platelet count<br>Red blood cell count<br>White blood cell count<br>White blood cell differential	Total bilirubin<br>Alanine transaminase (ALT)<br>Aspartate transaminase (AST)<br>Alkaline phosphatase<br>Total protein<br>Albumin<br>Sodium<br>Potassium<br>Chloride<br>Calcium<br>Phosphorus<br>Magnesium<br>Blood urea nitrogen (BUN) or urea<br>Creatinine<br>Uric acid<br>Glucose<br>LDH	Urinalysis (dipstick)<br>PSA<br>Testosterone<br>TSH<br>Fasting lipid panel

APPENDIX 3: ECOG PERFORMANCE STATUS

0	Fully active, able to carry on all pre-disease activities without restriction
1	Restricted in physically strenuous activity but ambulatory and able to carry out work of a light or sedentary nature, e.g., light house work or office work
2	Ambulatory and capable of all selfcare but unable to carry out any work activities. Up and about more than 50% of waking hours
3	Capable of only limited selfcare, confined to bed or chair more than 50% of waking hours
4	Completely disabled. Cannot carry on any selfcare. Totally confined to bed or chair
5	Dead

APPENDIX 4: NATIONAL CANCER INSTITUTE (NCI) COMMON TERMINOLOGY CRITERIA FOR ADVERSE EVENTS (CTCAE)

The NCI CTCAE (Version 4.0) may be reviewed on-line at the following NCI website:

http://ctep.cancer.gov/reporting/ctc.html

APPENDIX 5: PROHIBITED OR RESTRICTED MEDICATIONS OR SUPPLEMENTS WHILE ON STUDY

Medications/Supplements which are PROHIBITED while on study:

- Aminophylline/theophylline
- Atypical antipsychotics (e.g., clozapine, olanzapine, risperidone, ziprasidone)
- Buproprion
- Lithium
- Meperidine and pethidine
- Phenothiazine antipsychotics (e.g., chlorpromazine, mesoridazine, thioridazine)
- Tricyclic and tetracyclic antidepressants (e.g., amitriptyline, desipramine, doxepin, imipramine, maprotiline, mirtazapine)

Medications/Supplements which are NOT RECOMMENDED while on study (monitor for increased toxicity/potential drug interactions):

- ARN-509 (and its main metabolite ARN-309) are metabolized primarily by human CYP3A4, thus co-administration with any of the following agents has the potential to impact the pharmacokinetics of ARN-509 and alternative therapies should be used when available:
  - **Strong CYP3A4 inhibitors:** itraconazole, clarithromycin, erythromycin, diltiazem, verapamil, delavirdine, atazanavir, indinavir, nefazodone, nelfinavir, ritonavir, saquinavir, telithromycin, voriconazole, grapefruit juice (or grapefruits); co-administration with any of these agents may increase ARN-509 plasma concentrations
  - **Strong CYP inducers:** phenytoin, carbamazepine, rifampin, rifabutin, rifapentin, phenobarbital, efavirenz, tipranivir, St. John's wort; co-administration with any of these agents may decrease ARN-509 plasma concentrations
- ARN-509 may also induce CYP3A4; therefore, caution should be taken when administered in conjunction with CYP3A4 substrates that have a narrow therapeutic index
- The potential for drug-drug interaction between ARN-509 and warfarin (e.g., Coumadin) is unknown at present. If a patient is taking Coumadin, re-assess PT/INR as clinically indicated and adjust the dose of Coumadin accordingly.
- Due to possible resistance mechanisms which may be contributed by glucocorticoid receptor signaling, concurrent use of corticosteroids during the study is not recommended; short term use (≤ 4 weeks) will be allowed if clinically indicated, however, its use must be tapered off as soon as possible.
- ARN-509 is formulated with vitamin E; vitamin E may impair oral absorption of vitamin K. Avoid taking extra vitamin E.
- Avoid pomegranate juice or fruits

APPENDIX 6: FACT-P QUESTIONNAIRE

Version 4, US English, Copyright 1987, 1997

Below is a list of statements that other people with your illness have said are important. By circling one (1) number per line, please indicate how true each statement has been for you <u>during the past 7 days.</u>

	**PHYSICAL WELL -BEING**	**Not at all**	**A little bit**	**Some-what**	**Quite a bit**	**Very much**
GP1	I have a lack of energy	0	1	2	3	4
GP2	I have nausea	0	1	2	3	4
GP3	Because of my physical condition, I have trouble meeting the needs of my family	0	1	2	3	4
GP4	I have pain	0	1	2	3	4
GP5	I am bothered by side effects of treatment	0	1	2	3	4
GP6	I feel ill	0	1	2	3	4
GP7	I am forced to spend time in bed	0	1	2	3	4

	**SOCIAL/FAMILY WELL-BEING**	**Not at all**	**A little bit**	**Some-what**	**Quite a bit**	**Very much**
GS1	I feel close to my friends	0	1	2	3	4
GS2	I get emotional support from my family	0	1	2	3	4
GS3	I get support from my friends	0	1	2	3	4
GS4	My family has accepted my illness	0	1	2	3	4
GS5	I am satisfied with family communication about my illness	0	1	2	3	4
GS6	I feel close to my partner (or the person who is my main support)	0	1	2	3	4
Q1	*Regardless of your current level of sexual activity, please answer the following question. If you prefer not to answer it, please check this box ☐ and go to the next section.*	0	1	2	3	4
GS7	I am satisfied with my sex life	0	1	2	3	4

	EMOTIONAL WELL -BEING	Not at all	A little bit	Some-what	Quite a bit	Very much
GE1	I feel sad	0	1	2	3	4
GE2	I am satisfied with how I am coping with my illness	0	1	2	3	4
GE3	I am losing hope in the fight against my illness	0	1	2	3	4
GE4	I feel nervous	0	1	2	3	4
GE5	I worry about dying	0	1	2	3	4
GE6	I worry that my condition will get worse	0	1	2	3	4

	FUNCTIONAL WELL -BEING	Not at all	A little bit	Some-what	Quite a bit	Very much
GF1	I am able to work (include work at home)	0	1	2	3	4
GF2	My work (include work at home) is fulfilling	0	1	2	3	4
GF3	I am able to enjoy life	0	1	2	3	4
GF4	I have accepted my illness	0	1	2	3	4
GF5	I am sleeping well	0	1	2	3	4
GF6	I am enjoying the things I usually do for fun	0	1	2	3	4
GF7	I am content with the quality of my life right now	0	1	2	3	4

	ADDITIONAL CONCERNS	Not at all	A little bit	Some-what	Quite a bit	Very much
C2	I am losing weight	0	1	2	3	4
C6	I have a good appetite	0	1	2	3	4
P1	I have aches and pains that bother me	0	1	2	3	4
P2	I have certain parts of my body where I experience significant pain	0	1	2	3	4
P3	My pain keeps me from doing things I want to do	0	1	2	3	4
P4	I am satisfied with my present comfort level	0	1	2	3	4
P5	I am able to feel like a man	0	1	2	3	4
P6	I have trouble moving my bowels	0	1	2	3	4
P7	I have difficulty urinating	0	1	2	3	4
BL2	I urinate more frequently than usual	0	1	2	3	4
P8	My problems with urinating limit my activities	0	1	2	3	4
BL5	I am able to have and maintain an erection	0	1	2	3	4

APPENDIX 7: EQ-5D

By placing a checkmark in one box in each group below, please indicate which statements best describe your own health state today.

Mobility

I have no problems in walking about	❑
I have some problems in walking about	❑
I am confined to bed	❑

Self-Care

I have no problems with self-care	❑
I have some problems washing or dressing myself	❑
I am unable to wash or dress myself	❑

Usual Activities *(e.g., work, study, house work, family, or leisure activities)*

I have no problems with performing my usual activities	❑
I have some problems with performing my usual activities	❑
I am unable to perform my usual activities	❑

Pain/Discomfort

I have no pain or discomfort	❑
I have moderate pain or discomfort	❑
I have extreme pain or discomfort	❑

Anxiety/Depression

I am not anxious or depressed	❑
I am moderately anxious or depressed	❑
I am extremely anxious or depressed	❑

To help people say how good or bad a health state is, we have drawn a scale (rather like a thermometer) on which the best state you can imagine is marked 100 and the worst state you can imagine is marked 0.

We would like you to indicate on this scale how good or bad your own health is today, in your opinion. Please do this by drawing a line from the box below to whichever point on the scale indicates how good or bad your health state is today.

Your own health state today

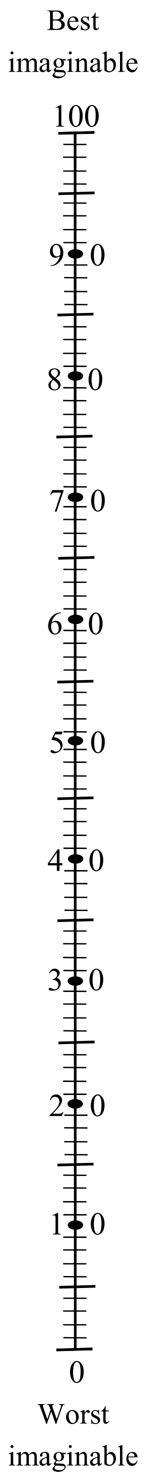

APPENDIX 8: VENTRICULAR REPOLARIZATION SUB-STUDY AT SELECTED SITES

Study Objectives

To evaluate the effect of ARN-509 on ventricular repolarization in a subset of patients from selected clinical sites:

- The primary objective of this sub-study is to evaluate whether ARN-509 has a threshold pharmacological effect on cardiac repolarization, as detected by changes in electrocardiogram (ECG) QT intervals corrected for heart rate by Fridericia's correction method (QTcF)
- The secondary objectives of this study sub-study are as follows:
  - To investigate the effect of ARN-509 on the following ECG parameters: PR, RR, QRS, QT, QTcB (Bazett's correction method), and T-Wave morphology
  - To further characterize the pharmacokinetic (PK) profile of ARN-509 and to assess the exposure-effect relationships (if any) between ARN-509 plasma concentrations and ECG interval change

Safety will be assessed as part of the main study. At the time of the analysis of this sub-study, all safety data from the main study will be provided along with data specific to this subset of patients. In particular, any cardiac-related adverse events will be summarized in both main study and sub-study populations.

Study Design and Patient Population

The effect of ARN-509 on ventricular repolarization will be centrally analyzed by a third-party cardiac safety laboratory in a subset of 100 patients enrolled at selected sites that will be participating in the main protocol. Both ARN-509 and placebo patients will be enrolled in a blinded manner as per the main protocol randomization criteria.

The subset of 100 patients will undergo the same screening procedures as the main protocol in order to be randomized into the study, following the same Inclusion/Exclusion criteria as per Section 4 of the protocol, **with the following additional enrollment criteria**:

Additional Inclusion Criteria	Additional Exclusion Criteria
▪ Enrollment in the main study<br>▪ Obtain separate informed consent for participation in the sub-study	▪ Heart rate outside of 50 to 100 beats/minute<br>▪ QTcF > 480 msec, determined by central assessment<br>▪ Diagnosed or suspected congenital long QT syndrome, or family history of congenital long QT syndrome or sudden death<br>▪ History of Mobitz II second degree or third degree heart block<br>▪ Implantable pacemaker or automatic implantable cardioverter defibrillator<br>▪ Complete Bundle Branch Block or ventricular conduction delay (QRS > 119 msec)<br>▪ Chronic or persistent atrial arrhythmia, including atrial fibrillation and atrial flutter.<br>▪ Concurrent therapy with medications known to prolong the QT interval and/or associated with TdP (Torsade de Pointes) arrhythmia [please refer to www.qtdrugs.org for the list of drugs to avoid]<br>▪ Smokers and planned nicotine replacement therapy users

Note: If patients do not qualify for the ventricular repolarization sub-study, they can still participate in the main study provided they meet all other inclusion/exclusion criteria as per Section 4 of the protocol.

Patient Withdrawal: A patient may withdraw his consent to participate in the sub-study at any time. If a patient withdraws such consent, the Investigator should inform the Sponsor (or designee) in writing and document in the Investigator Site File. The patient may continue participating in the main protocol.

Rationale for the Study Design

This sub-study has been designed to evaluate the potential of ARN-509 to prolong the QTc interval in a subset of patients with high risk non-metastatic castration-resistant prostate cancer participating in the main protocol Study ARN-509-003.

In accordance with ICH E14 guideline, QT evaluation is now expected to be routine in oncology drug development, and a Thorough QT (TQT) study should be conducted, if possible. In the case of ARN-509, in view of previous FDA advice and the observations to date that nonclinical (hERG and CV safety study) and clinical data (ECG collections in the Phase I/II Study ARN-509-001) suggest no apparent relationship between ARN-509 and QT prolongation, an alternative design to the TQT study has been chosen. In this sub-study, changes in QT interval following drug administration will be evaluated relative to the baseline measurement.

Rationale for the ECG Collection Time Points and PK Sampling Schedule

ECG time points have been selected to match the expected PK profile of ARN-509 and its metabolites. The ECG time points have also been selected to explore a potential shift between exposure and effect on QT/QTc.

In order to assess the QT interval prior to exposure to ARN-509, baseline triplicate ECG assessments will be performed twice prior to first study drug administration on Cycle 1 Day 1 (at Hour -1 and Hour 0 pre-dose). A baseline PK sample will also be collected at the Hour 0, immediately after the ECGs have been collected and just prior to the first dose of ARN-509 or placebo.

Subsequently, the purpose of all other ECGs is to assess for potential prolongation of the QT interval and other ECG changes as a result of ARN-509 administration, with ECG collection coinciding with PK measurements to establish correlations between ECG changes and drug exposure. Triplicate ECGs, followed by blood samples for PK assessment, will be collected at 2 and 4 hours post-dose on Cycle 1 Day 1 and Cycle 3 Day 1 (once steady-state can be assured). The 2- and 4-hour time points were selected because the oral plasma T_{max} of ARN-509 is generally between 2 and 4 hours post-dose. Although the two primary metabolites of ARN-509 (ARN000308 and ARN000066) typically have a later C_{max} than that of ARN-509 (i.e., [redacted] to [redacted] hours), the peak to trough fluctuation ratio of these metabolites in patient plasma has been shown to be minimal (less than [redacted] for ARN000308 and less than [redacted] for ARN000066). Therefore, the potential for significant effects from these metabolites at later time points not assessed by ECG is deemed very small.

Methods

Digital 12-lead ECG equipment will be provided to each clinical site participating in this sub-study by the central laboratory for the duration of the sub-study.

ECGs should be collected after the patient has rested quietly and is awake in a fully supine (or semi-recumbent, if supine is not tolerated) position for 10 minutes, and prior to any blood collection. Starting on Cycle 1 Day 1, time point matched blood samples for pharmacokinetic analyses will be collected immediately following the collection of ECGs and before the collection of blood for all other clinical evaluations. ECGs will be read by independent cardiologists from the central laboratory in a blinded manner and via single reader paradigm.

A detailed list of the required assessments is provided below. All other assessments at the other time points will follow the main protocol as per the Schedule of Activities in Appendix 1.

Schedule of Activities Specific to the Ventricular Repolarization Sub-Study:

- **Cycle 1 Day 1**
  - One hour prior to administering the first dose of ARN-509, collect a set of triplicate 12-lead ECGs, 2 minutes apart
  - At time 0, just prior to administering the first dose of ARN-509, collect a second set of triplicate 12-lead ECGs, 2 minutes apart, and one blood sample for PK analysis
  - Administer study drug in clinic
  - At 2 and 4 hours post-dose, collect a set of triplicate ECGs, 2 minutes apart, and one blood sample for PK analysis for each time point, respectively.

- **Cycle 3 Day 1 (Administer Study Drug in Clinic)**
  - At time 0, just prior to administering the dose of ARN-509, collect a set of triplicate 12-lead ECGs, 2 minutes apart, and one blood sample for trough PK analysis
  - Administer study drug in clinic
  - At 2 and 4 hours post-dose, collect a set of triplicate ECGs, 2 minutes apart, and one blood sample for PK analysis for each time point, respectively

	Cycle 1 Day 1				Cycle 3 Day 1		
Activities and Forms to be Completed Specific to the Sub-Study Only:	Hour -1 pre-dose	Hour 0 pre-dose	2 Hours post-dose	4 Hours post-dose	Hour 0 pre-dose	2 Hours post-dose	4 Hours post-dose
Triplicate 12-lead ECG	X	X	X	X	X	X	X
PK samples*		X	X	X	X	X	X

*** PK samples are to be collected immediately after each ECG; the main study PK sample between 0.5 and 4 hours post dose does NOT have to be collected for the 100 patients on this sub-study.**

Statistical Methods

A detailed Statistical Analysis Plan will be prepared prior to the planned analyses.

Two analyses will be performed: a primary analysis using the active treatment arm only, and a secondary analysis comparing the active and placebo arms, both accompanied by a pharmacokinetic/pharmacodynamics (PK/PD) analysis.

Standard ECG parameters will be determined for each ECG recording. Corrected QTc intervals will be determined using Fridericia's formula (QTcF) and Bazett's formula (QTcB). Changes in ECG intervals from baseline will be calculated. Baseline will be defined as the mean of the values for the triplicate ECG measurements taken pre-dose on Cycle 1 Day 1 (Hour -1 and Hour 0). In addition, QTcFs will be categorized based on ICH E14 guidelines. Tables will present the number and percentage of patients meeting or exceeding the following categories:

- QTc interval prolongation:
  - Absolute values > 450 to ≤ 480 msec
  - Absolute values > 480 to ≤ 500 msec
  - Absolute values > 500 msec

- QTc interval change from baseline:
  - Increase from baseline > 30 to ≤ 60 msec
  - Increase from baseline > 60 msec

PK/PD analyses will be performed using data from all subjects who have ECG data from at least one time point following the first dose. A linear modeling approach will be used to quantify the relationship (if any) between the plasma concentration of drug (ARN-509) and its principal metabolite (ARN000308), and the changes from baseline in the QTcF interval.

Sample Size Determination

A sample size of 100 patients will ensure that at least 60 patients treated with ARN-509 will be enrolled on the sub-study and 60 patients will provide at least 98.7% power to detect a true effect of 10 milliseconds (msec) change from baseline considering only the active group. Details for the power calculation and statistical assumptions are provided below.

Criteria for a "negative" result

It is assumed that the FDA criterion for regarding the outcome as negative (i.e., no QT prolongation concerns) will be that the mean QTcF increase observed in the active group should be significantly lower than a threshold of 20 msec at all on-study time points. "Significantly lower" means that the upper one-sided 95% confidence limit should be below the threshold.

For the primary analysis, the quantity of interest is the mean change from baseline (ΔQTcF). For the secondary analysis, the quantity of interest is the difference between the mean changes from baseline in the active and placebo groups (ΔΔQTcF), which should remove any fluctuations due to diurnal variation and/or changes due to time-on-study (e.g., disease progression).

Statistical Assumptions

[redacted]

[redacted]

Clinical Study Protocol

SPARTAN

(Selective **P**rostate **AR T**argeting with **ARN-509)**

A Multicenter, Randomized, Double-Blind, Placebo-Controlled, Phase III Study of ARN-509 in Men with Non-Metastatic (M0) Castration-Resistant Prostate Cancer

Protocol Number:	ARN-509-003<br>Amendment 8
Investigational Product:	JNJ-56021927 (apalutamide)
IND Number:	104676
EudraCT Number:	2012-004322-24
Development Phase:	3
Sponsor:	Aragon Pharmaceuticals, Inc*

*Aragon Pharmaceuticals, Inc. is a wholly-owned subsidiary of Johnson & Johnson. Janssen Research & Development, LLC is part of the Janssen Pharmaceutical Companies of Johnson & Johnson and provides various services to its affiliated company, Aragon Pharmaceuticals, Inc.

This study will be conducted under US Food & Drug Administration IND regulations (21 CFR Part 312).

Status: Approved
Date: 15 March 2017
Prepared by: Janssen Research & Development, LLC
Document No.: EDMS-ERI-70784696; 10

GCP Compliance: This study will be conducted in compliance with Good Clinical Practice, and applicable regulatory requirements.

The confidential information in the following document is provided to you as an investigator, potential investigator, or consultant, for review by you, your staff, and appropriate ethical review committee. By accepting this document, you agree that the information contained herein will not be disclosed to others without written authority from Aragon Pharmaceuticals, Inc., except to the extent necessary to obtain approval of this protocol by an ethical review committee.

SPONSOR APPROVALS

Protocol ARN-509-003 Amendment 8 Page 2 of 113

SPONSOR APPROVALS

Protocol: ARN-509-003 Amendment 8

Protocol Title: SPARTAN: A Multicenter, Randomized, Double-Blind, Placebo-Controlled, Phase III Study of ARN-509 in Men with Non-Metastatic (M0) Castration-Resistant Prostate Cancer

[redacted] 3/15/2017

Margaret Yu, MD
Clinical Leader

Aragon Pharmaceuticals, Inc - Confidential

PROTOCOL AGREEMENT

I confirm that I have read this protocol. I will comply with the protocol and the principles of Good Clinical Practice (GCP), as described in the United States Code of Federal Regulation (CFR) 21 Parts 11, 50, 54, 56, and 312 and the appropriate International Conference on Harmonisation guidance documents.

Protocol: ARN-509-003 Amendment 8

Protocol Title: SPARTAN: A Multicenter, Randomized, Double-Blind, Placebo-Controlled, Phase III Study of ARN-509 in Men with Non-Metastatic (M0) Castration-Resistant Prostate Cancer

_______________________________________ _______________

Investigator Signature *Date*

____

Print Name and Title

Site # ___________

Site Name _______________________________________

PROTOCOL SYNOPSIS

**Title**	A Multicenter, Randomized, Double-Blind, Placebo-Controlled, Phase III Study of ARN-509 in Men with Non-Metastatic (M0) Castration-Resistant Prostate Cancer
**Sponsor**	Aragon Pharmaceuticals, Inc
**Development Phase**	3
**Rationale**	Prostate cancer is the second most frequently diagnosed cancer and the sixth leading cause of cancer death in males, accounting for 14% (903,500) of the total new cancer cases and 6% (258,400) of the total cancer deaths in males worldwide. The course of prostate cancer from diagnosis to death is best categorized as a series of clinical stages based on the extent of disease, hormonal status, and absence or presence of detectable metastases: localized disease, rising levels of prostate-specific antigen (PSA) after radiation therapy or surgery with no detectable metastases, and clinical metastases in the non-castrate or castrate stage. Although surgery, radiation, or a combination of both can be curative for patients with localized disease, a significant proportion of these patients have recurrent disease as evidenced by a rising level of PSA, which can lead to the development of metastases, especially in the high risk group – a transition to the lethal stage of the disease.<br><br>Androgen depletion is the standard treatment with a generally predictable outcome: decline in PSA, a period of stability in which the tumor does not proliferate, followed by rising PSA and regrowth as castration-resistant disease. Molecular profiling studies of castration-resistance prostate cancers commonly show increased androgen receptor (AR) expression, which can occur through AR gene amplification or other mechanisms.<br><br>ARN-509 (JNJ-56021927; apalutamide, hereafter referred to as apalutamide) is a next-generation anti-androgen that binds directly to the ligand-binding domain of AR, impairing nuclear translocation and DNA binding. Apalutamide binds AR with [redacted] greater affinity than bicalutamide, and induces partial or complete tumor regression in both castration-sensitive and castration-resistant human prostate cancer xenograft models.<br><br>A Phase I/II study (Protocol ARN-509-001) was designed to assess the safety, pharmacokinetics, and anti-tumor activity of apalutamide in men with castration-resistant prostate cancer (CRPC). The Phase I portion of the study has been completed and the Phase II is currently ongoing. In the Phase II, 97 patients were enrolled across 3 different patient population subsets: high risk NM-CRPC, treatment-naïve metastatic CRPC (no prior treatment with abiraterone acetate or chemotherapy), and metastatic CRPC after

	failure with abiraterone acetate.<br>In the NM-CRPC subset (n =51), the most frequent treatment-related adverse events reported in >10% of subjects as of 30 November 2013 are fatigue (43%), diarrhea (31%), nausea (20%), increased thyroid stimulating hormone (12%), dysgeusia (12%), and hot flush 12%. Eight (16%) patients have discontinued the study due to adverse events. Seven (14%) patients have discontinued due to disease progression. No deaths were reported in this subgroup as of the safety cutoff. The 12-week PSA response (≥ 50% decline from baseline) is 91%.
**Study Design**	This is a randomized (2:1), multicenter, double-blind, placebo-controlled, Phase III clinical trial evaluating the efficacy and safety of apalutamide versus placebo in men with high risk (M0) NM-CRPC, defined as PSA Doubling Time (PSADT) ≤ 10 months.<br>Patients will be stratified based on:<br>▪ PSADT: > 6 months vs. ≤ 6 months<br>▪ Bone-sparing agent use: Yes vs. No<br>▪ Loco-regional disease: N0 vs. N1<br>Patients will be followed for safety and efficacy as per the schedule of activities and will remain on study treatment until documented radiographic progression (development of distant metastases as assessed by blinded independent central review) or the development of unacceptable toxicity.<br>Patients discontinuing treatment due to documented radiographic progression will enter the Long-term Follow-up Phase, where they will be followed for the development of symptomatic progression and initiation of subsequent anti-cancer therapies (in particular, cytotoxic chemotherapy) every 4 months until death, loss of follow-up, or withdrawal of consent, whichever comes first.<br>Patients discontinuing treatment prior to documented radiographic progression will also enter the Long-term Follow-up Phase where they will continue to have scheduled disease assessments every 16 weeks until documented radiographic progression, and will be followed for the development of symptomatic progression and initiation of subsequent anti-cancer therapies (in particular, cytotoxic chemotherapy) every 4 months until death, loss of follow-up, or withdrawal of consent, whichever comes first.<br>At the time of study unblinding and in the event of a positive study result, all subjects currently receiving placebo will have the opportunity to receive active therapy with apalutamide.

**Primary Objective**	To demonstrate superiority in the metastasis-free survival (MFS) of men with high risk NM-CRPC treated with apalutamide versus placebo
**Secondary Objectives**	▪ To compare the overall survival (OS) of men with high risk NM-CRPC treated with apalutamide versus placebo<br>▪ To compare the time to symptomatic progression in men with high risk NM-CRPC treated with apalutamide versus placebo<br>▪ To compare the time to initiation of cytotoxic chemotherapy in men with high risk NM-CRPC treated with apalutamide versus placebo<br>▪ To compare the progression-free survival (PFS) of men with high risk NM-CRPC treated with apalutamide versus placebo<br>▪ To compare the time to metastasis (TTM) in men with high risk NM-CRPC treated with apalutamide versus placebo<br>▪ To evaluate the safety and tolerability of apalutamide
**Other Objectives**	▪ To compare patient reported outcomes (PROs) of health-related quality of life and prostate cancer-specific symptoms in men with high risk NM-CRPC treated with apalutamide versus placebo<br>▪ To evaluate the population pharmacokinetics (PK) of apalutamide<br>▪ To evaluate the effect of apalutamide on ventricular repolarization in a subset of patients from selected clinical sites<br>▪ To evaluate exploratory biomarkers predictive of response and resistance to apalutamide treatment
**Number of Patients**	1,200 (apalutamide: 800; placebo: 400)
**Enrollment Criteria**	The study population includes men 18 years of age or older with NM-CRPC at high-risk for metastases defined as a prostate-specific antigen doubling time (PSADT) of 10 months or less. Patients are excluded if blinded independent central review (BICR) confirms the presence of distant metastases including CNS and vertebral or meningeal involvement or a history of distant metastases. Patients who present at Screening with lymph nodes less than 2 cm in the short axis (N1) located below the iliac bifurcation, are eligible. Any patient who received first generation anti-androgens must show continuing disease (PSA) progression after discontinuation of the anti-androgen for at least 4 weeks prior to randomization. Bone sparing therapies for the treatment of osteoporosis are allowed if the patient were on stable doses for at least 4 weeks before randomization. Patients with symptomatic loco-regional disease

	requiring medical intervention are excluded. An Eastern Cooperative Oncology Group (ECOG) Performance Status (PS) grade of 0 or 1 is required
**Dose, and Route of Administration**	Apalutamide/matched placebo tablets will be administered orally on a continuous daily dosing regimen, at a starting dose for apalutamide of 240 mg once daily (4 x 60-mg tablets). The only difference between apalutamide tablet and its matched placebo tablet is the absence of the active ingredient in the matched placebo tablet.
**Safety Assessments**	Patients will be assessed for adverse events (AEs) at each monthly clinic visit while on the study. Adverse events will be graded according to the National Cancer Institute (NCI) Common Terminology Criteria for Adverse Events (CTCAE) Version 4.0. Type, incidence, severity, timing, seriousness, and relatedness of AEs, and laboratory abnormalities will be reported.
**Data Monitoring Committee**	An Independent Data Monitoring Committee (IDMC) will be established to monitor data on an ongoing basis to ensure the safety of the subjects enrolled in this study. The committee will meet periodically to review interim safety data. After the review, the IDMC will make recommendations regarding the conduct of the study. The IDMC will serve as the primary reviewers of the efficacy analysis. Details are provided in a separate IDMC charter.
**Efficacy Assessments**	Disease assessments will be performed as scheduled according to the calendar, regardless of treatment delays resulting from toxicity. Disease assessments (CT scans of the chest, abdomen, and pelvis, plus bone scan) will be performed at baseline and at 16-week intervals from Cycle 1 Day 1 until documented progression. All scans will be submitted for BICR by a third-party core imaging laboratory to confirm patient eligibility (i.e., no presence of distant metastases) and disease progression during the study.
**Primary Endpoint**	▪ Metastasis-free survival (MFS)
**Secondary Endpoints**	▪ Time to Metastasis (TTM)<br>▪ PFS<br>▪ Time to symptomatic progression<br>▪ Overall survival (OS)<br>▪ Time to initiation of cytotoxic chemotherapy
**Other Evaluations**	▪ Health-related quality of life and prostate cancer-specific symptoms

	▪ Type, incidence, severity, timing, seriousness, and relatedness of adverse events and laboratory abnormalities<br>▪ PSA Response<br>▪ Time to PSA progression<br>▪ Population PK<br>▪ Assessment of ventricular repolarization<br>▪ Second progression-free survival (PFS2)<br>▪ Medical resource utilization (MRU)
**Statistical Analysis Plan and Rationale for Number of Patients**	The primary efficacy analysis will be event-driven. Based on results from a large Phase III study of denosumab versus placebo in high risk NM-CRPC patients, the expected median MFS in the control arm is 25 months.<br><br>Applying a 2:1 randomization, a planned accrual period of 24 months and a minimum follow-up period of [redacted] months, it is estimated that approximately 1,200 patients will need to be enrolled in order to observe [redacted] MFS events. This sample size has 90% power to detect a [redacted] reduction in the risk of developing metastases (HR = [redacted]) for patients receiving apalutamide, with a 2-sided α of 0.05. This treatment effect corresponds to an increase in median MFS of approximately [redacted] months (from 25 to [redacted] months), which would be considered clinically relevant.<br><br>The study was also designed to provide 85% power to detect a [redacted] reduction (HR = [redacted]) in the risk of death for patients receiving apalutamide, based on an assumed median OS of [redacted] months in the placebo arm. This treatment effect represents an increase in median OS of approximately [redacted] months (from [redacted] to [redacted] months).

**Duration of Patient Participation and Duration of Study**	Patients will remain on study treatment until BICR-confirmed radiographic disease progression (ie, distant metastasis), development of unacceptable toxicity, or withdrawal of consent. Patients discontinuing study treatment will enter the Long-term Follow-up Phase and remain on study until death, loss of follow-up, or withdrawal of consent, whichever comes first.<br><br>With an estimated accrual duration of 24 months, it is assumed that patients are expected to be followed for a minimum of approximately [redacted] months beyond Last Patient In (LPI) for the primary endpoint of MFS, to approximately [redacted] months beyond LPI for the key secondary endpoint of OS. This corresponds to a projected study duration of approximately 65 months.<br><br>If the study is not terminated beforehand per the recommendation of the IDMC, the end of trial in all participating countries will be defined as the time at which the secondary endpoint of OS has been met.

LIST OF ABBREVIATIONS

**ADT**	androgen deprivation therapy
**AE**	adverse event
**ALT**	alanine aminotransferase
**ANC**	absolute neutrophil count
**AR**	androgen receptor
**AST**	aspartate aminotransferase
**BICR**	blinded independent central review
**BSE**	bovine spongiform encephalopathy
**BUN**	blood urea nitrogen
**CFR**	Code of Federal Regulations
**CNS**	central nervous system
**CRF**	case report form
**CRPC**	castration-resistant prostate cancer
**CTCAE**	Common Terminology Criteria for Adverse Events
**DLT**	dose-limiting toxicity
**ECG**	electrocardiogram
**FFPE**	formalin-fixed paraffin-embedded
**EDC**	electronic data capture
**EWB**	emotional well-being
**FACT-P**	Functional Assessment of Cancer Therapy-Prostate
**FDA**	Food and Drug Administration
**FDHT**	16β-[18F] fluoro-α-dihydrotestosterone
**FWB**	functional well-being
**GCP**	Good Clinical Practice
**GnRHa**	gonadotropin releasing hormone analog
**HIPAA**	Health Insurance Portability and Accountability Act of 1996
**HIV**	human immunodeficiency virus
**ICF**	informed consent form
**ICH**	International Conference on Harmonisation
**IDMC**	Independent Data Monitoring Committee
**IEC**	Independent Ethics Committee
**IRB**	Institutional Review Board
**MFS**	metastasis-free survival
**NCI**	National Cancer Institute

**NM-CRPC**	non metastatic castration-resistant prostate cancer
**NOAEL**	no observed adverse effect level
**OS**	overall survival
**PCWG2**	Prostate Cancer Clinical Trials Working Group 2
**PET**	positron emission tomography
**PFS**	progression-free survival
**PK**	pharmacokinetics
**PQC**	product quality complaint
**PSA**	prostate-specific antigen
**PSADT**	prostate-specific antigen doubling time
**PWB**	physical well-being
**QTc**	corrected QT interval
**QTcB**	corrected QT interval according to the Bazett correction
**QTcF**	corrected QT interval according to the Fridericia correction
**RECIST**	Response Evaluation Criteria in Solid Tumors
**SAE**	serious adverse event
**SGOT**	serum glutamic oxaloacetic transaminase
**SGPT**	serum glutamate pyruvate transaminase
**SOC**	system organ class
**SWB**	social well-being
**TPGS**	d-α-tocopheryl polyethylene glycol 1000 succinate
**TTM**	time to metastasis
**ULN**	upper limit of normal

TABLE OF CONTENTS

PROTOCOL AMENDMENTS 16
1. BACKGROUND 38
1.1 APALUTAMIDE 38
1.1.1 Molecular Formula and Chemical Class 39
1.1.2 Pre-Clinical Development Overview 39
1.1.3 Overview of Clinical Studies 42
2. STUDY OBJECTIVES 44
2.1 PRIMARY OBJECTIVE 44
2.2 SECONDARY OBJECTIVES 44
2.3 OTHER OBJECTIVES 44
3. STUDY DESIGN 45
3.1 STUDY OVERVIEW AND RATIONALE 45
3.1.1 Crossover Option in the Event of Unblinding 46
3.1.2 Selection of the Primary Endpoint 46
3.2 STUDY OUTCOMES 47
3.2.1 Primary Endpoint 47
3.2.2 Secondary Endpoints 47
3.2.3 Other Evaluations 47
4. PATIENT SELECTION 47
4.1 INCLUSION CRITERIA 48
4.2 EXCLUSION CRITERIA 50
5. STUDY TREATMENTS 52
5.1 RANDOMIZATION CRITERIA 52
5.2 BLINDING 52
5.3 FORMULATION 53
5.3.1 Apalutamide/Matched Placebo 53
5.3.2 Packaging, Storage, and Labeling 53
5.3.3 Drug Administration 53
5.3.4 Cycle Management 53
5.3.5 Dose Modifications 53
5.4 STUDY DRUG ACCOUNTABILITY 54
5.5 MEASURES OF TREATMENT COMPLIANCE 55
6. CONCURRENT MEDICATIONS 55
6.1 PROHIBITED MEDICATIONS AND TREATMENTS 55
6.2 RESTRICTED THERAPIES 55
6.3 LIFE STYLE GUIDELINES 56
7. STUDY PROCEDURES AND GUIDELINES 58
7.1 CLINICAL ASSESSMENTS 58
7.1.1 Demographics 58
7.1.2 Medical History 58

7.1.3 Physical Examination....58
7.1.4 Vital Signs....58
7.1.5 Performance Status....59
7.1.6 Adverse Events....59
7.1.7 Concomitant Medications/Therapies....59
7.1.8 Tumor Assessments....59
7.1.8.1 Blinded Independent Central Review (BICR)....59
7.2 CLINICAL LABORATORY MEASUREMENTS....60
7.2.1 PSA....60
7.2.2 Electrocardiogram (ECG)....60
7.3 PHARMACOKINETIC MEASUREMENTS....60
7.4 PATIENT-REPORTED OUTCOMES....61
7.5 EXPLORATORY BIOMARKERS....61
8. STUDY ASSESSMENTS BY VISIT (APPENDIX 1)....62
8.1 PRESCREENING....62
8.2 SCREENING (WITHIN 35 DAYS OF RANDOMIZATION)....62
8.3 CYCLE 1 DAY 1....62
8.4 DAY 1 OF CYCLES 1-6, THEN DAY 1 OF EVERY 2 CYCLES STARTING FROM CYCLE 7 UP TO C13 THEN DAY 1 OF EVERY 4 CYCLES (±2 DAYS)....63
8.5 EVERY 16 WEEKS STARTING FROM CYCLE 1 DAY 1 (±2 DAYS)....63
8.6 EVERY 16 WEEKS STARTING FROM CYCLE 1 DAY 1 (±7 DAYS)....63
8.7 END-OF-TREATMENT VISIT....63
8.8 SAFETY FOLLOW-UP (28 DAYS FOLLOWING THE LAST DOSE OF STUDY DRUG)....64
8.9 LONG-TERM FOLLOW-UP....64
8.10 SUBSEQUENT THERAPY WITH ABIRATERONE ACETATE....64
9. ADVERSE EVENT REPORTING REQUIREMENTS....66
9.1 DEFINITIONS....66
9.1.1 Adverse Events (AE)....66
9.1.2 Serious Adverse Events (SAE)....67
9.1.3 Expectedness....67
9.1.4 Attribution....68
9.1.5 Severity....68
9.1.6 Pregnancy....69
9.2 REPORTING REQUIREMENTS....69
9.2.1 SAE Reporting....69
9.2.2 Non-Serious AE Reporting....70
9.2.3 Sponsor Reporting Requirements to Regulatory Authorities....70
9.2.4 Product Quality Complaint Handling....71
9.2.4.1 Procedures....71
9.2.4.2 Contacting Sponsor Regarding Product Quality....71
10. END OF TREATMENT....71
11. PROTOCOL VIOLATIONS....72

12. DATA MONITORING COMMITTEE 73
13. STATISTICAL METHODS AND CONSIDERATIONS 73
13.1 ANALYSIS POPULATIONS 73
13.2 EFFICACY ANALYSES 73
13.2.1 Analysis of Primary Endpoint 74
13.2.2 Analyses of Secondary Endpoints 75
13.2.2.1 OS 76
13.2.2.2 Time to Symptomatic Progression 76
13.2.2.3 Time to Initiation of Cytotoxic Chemotherapy 76
13.2.2.4 Progression-Free Survival 77
13.2.2.5 Time to Metastasis 78
13.3 SAFETY EVALUATIONS 78
13.3.1 Analysis of Adverse Events 78
13.3.2 Analysis of Clinical Laboratory Results 79
13.3.3 Analysis of Vital Signs 79
13.3.4 Concomitant Medications/Treatments 79
13.4 OTHER EVALUATIONS 80
13.4.1 Second Progression-free Survival (PFS2) 80
13.4.2 PSA 80
13.4.3 Health-Related Quality of Life and Prostate Cancer-Specific Symptoms Analysis 80
13.4.4 Population Pharmacokinetics (Pop PK) Analysis 80
13.4.5 Exploratory Biomarkers Analysis 81
13.4.6 Assessment of Ventricular Repolarization 81
13.4.7 Medical Resource Utilization Analysis 81
13.5 INTERIM ANALYSIS 82
13.6 DETERMINATION OF SAMPLE SIZE 82
14. DATA COLLECTION, RETENTION AND MONITORING 82
14.1 DATA COLLECTION INSTRUMENTS 82
14.2 DATA MANAGEMENT PROCEDURES 82
14.3 DATA QUALITY CONTROL AND REPORTING 83
14.4 ARCHIVAL OF DATA 83
14.5 AVAILABILITY AND RETENTION OF INVESTIGATIONAL RECORDS 83
14.6 MONITORING 83
14.7 PATIENT CONFIDENTIALITY 84
15. ADMINISTRATIVE, ETHICAL, REGULATORY CONSIDERATIONS 84
15.1 INVESTIGATOR RESPONSIBILITIES 84
15.2 PROTOCOL AMENDMENTS 84
15.3 INSTITUTIONAL REVIEW BOARDS AND INDEPENDENT ETHICS COMMITTEES 85
15.4 INFORMED CONSENT FORM 85
15.5 REPORTING OF SAFETY ISSUES AND SERIOUS BREACHES OF THE PROTOCOL OR ICH GCP 86
15.6 END OF TRIAL IN ALL PARTICIPATING COUNTRIES 86
15.7 SPONSOR DISCONTINUATION CRITERIA 86
15.8 PUBLICATIONS 87

16. REFERENCES 87
17. APPENDICES 89

PROTOCOL AMENDMENTS

Protocol Version	Issue Date
Original Protocol	5 November 2012
Amendment INT-1*	11 January 2013
Amendment INT-2*	8 May 2013
Amendment INT-3	11 March 2014
Amendment INT-4	16 June 2014
Amendment INT-5	1 July 2014
Amendment INT-6	18 May 2015
Amendment 7	1 June 2016
Amendment 8	15 March 2017

*These amendment changes are provided in a separate document (EDMS-ERI-71567787)
Amendments are listed beginning with the most recent amendment.

Amendment 8

This amendment is considered to be substantial based on the criteria set forth in Article 10(a) of Directive 2001/20/EC of the European Parliament and the Council of the European Union.

The overall reason for this amendment is to revise the statistical sections of the protocol. The current statistical analysis plan for analysis of [redacted]; therefore, the Sponsor is revising the [redacted]. In addition, text was added to allow, in the event of a positive study result and unblinding, for subjects receiving placebo to begin receiving active therapy with apalutamide. The rationale for and description of the changes are listed below, when revisions are provided verbatim, bold font denotes new text, and strikethrough denotes deleted text.

Applicable Section(s)	Description of Change(s)
**Rationale:** The current statistical analysis plan for analysis of [redacted] therefore, the Sponsor is revising the [redacted]	
Section 13.2.2, Analyses of Secondary Endpoints	Deleted:<br>~~Following the primary analysis of MFS, the analyses of the secondary endpoints will be performed at the time of the expected total number of MFS events ([redacted] events).~~<br>~~The secondary endpoints will be subdivided into the following 2 groups:~~<br>~~Group 1:~~<br>[redacted]<br>~~Group 2:~~<br>[redacted]<br>~~The statistical testing of the 2 groups of secondary endpoints will be performed by allocating~~ [redacted] ~~to Group 1 and~~ [redacted] ~~for the~~ [redacted] ~~endpoint in Group 2 with an overall familywise type I error rate of 0.05. Details of the testing procedure will be provided in the statistical analysis plan.~~

Applicable Section(s)	Description of Change(s)
Section 13.2.2, Analyses of Secondary Endpoints (cont's)	Replaced with:<br>**For the secondary endpoints, [redacted] testing will be performed in the following order:**<br>• [redacted]<br>• [redacted]<br>• [redacted]<br>• [redacted]<br>• [redacted]<br>**each [redacted] point will have a [redacted] analysis but there will be [redacted] for [redacted] an [redacted] The testing of [redacted] endpoints will utilize [redacted] method, according to the [redacted] alpha spending function with [redacted] number of events necessary for the [redacted] analysis to maintain the [redacted] power. [redacted]**<br>**The final analysis of [redacted] OS and [redacted] will [redacted] be performed at [redacted] the primary analysis of MFS (approximately [redacted] events).**<br>**Full details about the [redacted] procedure will be provided in the statistical analysis plan.**<br><br>Time-to-event-based secondary endpoint analyses ([redacted] will be performed using a two-sided log-rank test, stratified by PSADT (> 6 months vs. ≤ 6 months), the use of a bone-sparing agent (Yes vs. No), and the presence of loco-regional disease (N0 vs. N1).
**Rationale:** The next 3 changes were made in the sections identified as a result of changes made to the analysis of secondary endpoints (covered in Section 13.2.2).	
Section 13.2.2.1, OS	Deleted (text that was following table in this section):<br>~~The final analysis of OS will occur after approximately [redacted] deaths have occurred. Two interim analyses are also planned (see Section 13.5). Additional analyses may be conducted as appropriate.).~~
Section 13.4.2, PSA	PSA kinetics (e.g., 12-week PSA response and time to PSA progression) will be assessed ~~at the time of the primary analysis of MFS~~ according to the Prostate Cancer Clinical Trials Working Group (PCWG2) recommendations.

Applicable Section(s)	Description of Change(s)
Section 13.5, Interim Analysis	Deleted:<br>~~Two interim analyses and 1 final analysis are planned for the OS endpoint. The testing of the OS endpoint will be performed according to the O'Brien-Fleming boundary as implemented by the Lan-DeMets alpha spending function with a planned alpha of 0.04. For the OS endpoint, the maximum expected total number of death events is~~ [redacted] ~~(final analysis). At the time of the first interim analysis, approximately~~ [redacted] ~~) of the death events will be observed with an alpha spend of~~ [redacted] ~~.~~ ~~The second interim analysis will be performed after observing approximately~~ [redacted] ~~of the death events with a cumulative alpha spend of~~ [redacted] ~~. The actual alpha spent for the 2 interim analyses will be based on the number of death events observed at the time of the analyses.~~<br><br>Replaced with:<br>**There will be** [redacted] **see Section 13.2.2 for an outline of analysis methods**.
**Rationale:** Added details for placebo subjects to begin receiving active treatment with apalutamide, in the event of a positive study result and the study is unblinded.	
Synopsis, Study Design; Section 3.1.1, Crossover Option in the Event of Unblinding; Section 4, Patient Selection; and Appendix 10, Crossover to Open Label Apalutamide After Study Unblinding	**Synopsis and Section 3.1.1: At the time of study unblinding and in the event of a positive study result, all subjects currently receiving placebo will have the opportunity to receive active therapy with apalutamide (refer to Appendix 10).**<br><br>**Section 4: Treatment criteria for placebo subjects who crossover to active treatment with apalutamide (in the event of a positive study result and unblinding) are described in Appendix 10.**<br><br>New Appendix 10 added (not included here in summary of changes due to size).
**Rationale:** Changed 'is' to 'was' to clarify that the text in this section reflects thinking at the time the original protocol was written; deleted text that is no longer applicable.	
Section 13.6, Determination of Sample Size	The study ~~is~~ **was** also sized to provide 85% power to detect a [redacted]% reduction (HR = [redacted]) in the risk of death for patients receiving apalutamide, based on an assumed median OS of [redacted] months in the placebo arm. This treatment effect represents an increase in the median OS of approximately [redacted] months (from [redacted] months). ~~The final analysis of OS will occur after approximately~~ [redacted] ~~deaths have occurred.~~<br>~~The total study duration (including the time it takes to reach the secondary endpoint of OS) will be approximately 65 months.~~
Synopsis, Statistical Analysis Plan and Rationale for Number of Patients	The study ~~is~~ **was** also designed to provide 85% power to detect a [redacted] reduction (HR = [redacted]) in the risk of death for patients receiving apalutamide, based on an assumed median OS of [redacted] months in the placebo arm. This treatment effect represents an increase in median OS of approximately [redacted] months (from [redacted] to [redacted] months).<br>~~Two interim analyses and 1 final analysis will be performed for the OS endpoint. The final analysis for OS will occur after approximately~~ [redacted] ~~deaths have been observed.~~ ~~The first interim analysis of OS (approximately~~ [redacted] ~~% of total death events) will occur at the time of the final analysis of the primary endpoint, MFS, and the second interim analysis will occur when approximately~~ [redacted] ~~% of total death events are observed.~~

Applicable Section(s)	Description of Change(s)
**Rationale:** Correction of the median OS time in the study population.	
Section 3.1.2, Selection of the Primary Endpoint	In consideration of the relatively long median OS [redacted]) in this population and the opportunity to assess for the development of distant metastasis as a clinically important milestone, metastasis-free survival (MFS) was chosen as the primary objective of this study.
**Rationale:** Correction was made to note that email can be used for reporting of SAEs in addition to fax.	
Section 9.2.1, SAE Reporting	The initial and follow-up reports of an SAE should be made by facsimile (fax) **or email.**
**Rationale:** Clarification for criteria of disease progression for administration of subsequent therapy with abiraterone acetate, if the study is unblinded.	
Section 8.10, Subsequent therapy with Abiraterone Acetate (second bullet)	• documented disease progression (ie, meet criteria for the primary endpoint [distant metastasis], see Section 13.2.1). **If the study is unblinded, the requirement for meeting disease progression by BICR in Section 13.2.1 will not be required.**
**Rationale:** Minor errors were corrected, minor revisions were made.	
Throughout the protocol	Minor grammatical, formatting, or spelling changes were made. Minor organizational changes were made.

Amendment 7 (1 June 2016)

This amendment is considered to be non-substantial based on the criteria set forth in Article 10(a) of Directive 2001/20/EC of the European Parliament and the Council of the European Union, in that it does not significantly impact the safety or physical/mental integrity of subjects, nor the scientific value of the study.

The overall reason for this amendment is to reduce the frequency of visits after Cycle 7 per request of the investigators and patients. The revised visit schedule is consistent with other apalutamide Phase 3 protocols. The tablet formulation with improved shelf-life allows for fewer visits. Collection of medical resource utilization (MRU) should continue during the Long-term Follow-up Phase.

The rationale for and description of the changes are listed below, when revisions are provided verbatim, bold font denotes new text, and strikethrough denotes deleted text.

Applicable Section(s)	Description of Change(s)
**Rationale:** A reduction in the clinic visits will reduce burden to patients and the improved shelf-life of the tablets also allows for fewer clinic visits.	
Section 8.4 Day 1 of Cycles N (±2 Days); Appendix 1, Schedule of Activities	Visit frequency revised to the following: Every cycle up to Cycle 6, starting at Cycle 7 reduce to every 2 cycles (eg, C9, C11, etc), starting at Cycle 13 reduce to every 4 cycles (eg, C17, C21, etc).

Applicable Section(s)	Description of Change(s)
**Rationale:** The PK and biomarker sample collection timings were revised to align with the revised visit schedule.	
Section 7.3 Pharmacokinetic Measurements; Section 8.4 Day 1 of Cycles N (±2 Days); Section 13.4.5 Exploratory Biomarker Analysis; Appendix 1, Schedule of Activities	To align the PK sample collection with the revised visit schedule, the visits for PK collection were changed from Cycle 12 to Cycle 11, Cycle 18 to Cycle 17, and Cycle 24 to Cycle 25. To align the biomarker sample collection with the revised visit schedule, the visits for biomarker sample collection were changed from Cycle 12 to Cycle 11, Cycle 18 to Cycle 17, from Cycle 24 to Cycle 25, and from Cycle 36 to Cycle 37.
**Rationale:** The frequency for completion of the FACT-P and EQ-5D questionnaires will coincide with the revised clinic visit schedule during the Treatment Phase.	
Appendix 1 Schedule of Activities	The collection timing of the questionnaires was aligned with the reduced number of clinic visits.
**Rationale:** Collection of MRU should continue during the Long-term Follow-up Phase.	
Section 8.9 Long-term Follow-up; Appendix 1, Schedule of Activities	Added collection of MRU to the Long-term Follow-up Phase.
**Rationale:** The Survival Follow-up includes collection of other information and referring to this phase as Survival Follow-up was not accurate.	
Synopsis Study Design, Duration of Patient Participation and Duration of Study; Section 4.1 Inclusion Criteria; Section 8.9 Long-term Follow-up; Section 10 End of Treatment; Section 15.6 End of Trial in all Participating Countries; Appendix 1, Schedule of Activities	Revised the name of this phase of the study to "Long-term Follow-up Phase."<br>Inclusion Criterion #13.2 was revised to the following: Willingness and ability to comply with scheduled visits, treatment plans, laboratory and radiographic assessments, and other study procedures, including ability to swallow study drug tablets, the completion of patient reported outcomes questionnaires and ~~survival~~ **long-term** follow-up ~~visits~~

Applicable Section(s)	Description of Change(s)
**Rationale:** If thyroid stimulating hormone (TSH) levels are abnormal, additional reflex testing must be performed.	
Section 8.5 Every 16 weeks…; Appendix 1 Schedule of Activities: Footnote 11; Appendix 2	Added the following statement to Appendix 2:<br>**If TSH is abnormal; total T3, free T4 (direct), and total T4 are required.**<br>Added reference to Appendix 2 in Footnote 11 of Appendix 1
**Rationale:** SAE reporting requirements were not aligned with the current Janssen protocol template.	
Section 9.2.1 SAE Reporting	Made the following revision: Information regarding SAEs will be transmitted to the Sponsor using the Serious Adverse Event Form, which must be completed and signed by a physician from the study site, and transmitted to the Sponsor within 24 hours.**The initial and follow-up reports of an SAE should be made by facsimile (fax).** This timeframe also applies to additional new information (follow-up). SAEs should be reported by facsimile or email.
**Rationale:** All patients will have switched from softgel capsules to tablets at the time of implementation of this amendment therefore guidance for the switching, is no longer necessary.	
Section 5.3.3 Drug Administration	Removed guidance for the switch from softgel capsules to tablets.
**Rationale:** A generic name for ARN-509 is now available.	
Throughout the protocol, where appropriate	The designation of ARN-509 is changed to the generic name, apalutamide.
**Rationale:** Updated drug-drug interaction information is available	
Section 6.2 Restricted Therapies; Attachment 5	Updated drug-drug interaction information.
**Rationale:** All Janssen Phase 3 protocols must now include a list of anticipated events.	
Section 9.2 Reporting Requirements; Section 9.2.3 Sponsor Reporting Requirements to Regulatory Authorities; Appendix 9	Added details for reporting and a list of anticipated events in Appendix 9. A statement to reference the Appendix was added to Section 9.2 of the protocol. Additional information in cases of serious anticipated events was added to Section 9.2.3 as follows: **For anticipated events reported as individual serious adverse events the Sponsor will make a determination of relatedness in addition to and independent of the investigator's assessment. The Sponsor will periodically evaluate the accumulating data and, when there is sufficient evidence and the sponsor has determined there is a reasonable possibility that the drug caused a serious anticipated event, they will submit a safety report in narrative format to the investigators (and the head of the institute).**

Applicable Section(s)	Description of Change(s)
**Rationale:** Minor errors or clarifications were noted	
Synopsis, Duration of Patient Participation…; Section 8.10 Subsequent Therapy with Abiraterone Acetate; Section 8.6 Every 16 Weeks…	Added “distant metastasis” after the term disease progression.
Protocol Synopsis Study Design; Section 3.1 Study Overview and Rationale; Section 8.9 Long-term Follow-up Appendix 1 Schedule of Activities, Footnotes 4 and 13	When referring to scan frequency revised from 4 months to 16 weeks for consistency with the scan frequency in the Schedule of Activities.
Section 8.9 Long-term Follow-up; Appendix 1 Schedule of Activities, Footnotes 4 and 21	Added “an alternative contact method per institution policy/practice.”
Section 4.1 Inclusion Criteria; Section 4.2 Exclusion Criteria	Corrected the formatting per template
Throughout the protocol	Removed redundancy and made minor corrections.

<u>Amendment INT-6</u> (18 May 2015)

This amendment is considered to be substantial based on the criteria set forth in Article 10(a) of Directive 2001/20/EC of the European Parliament and the Council of the European Union.

The overall reason for this amendment is to switch softgel capsules to tablets (commercial formulation) for patients currently receiving the softgel capsules and to administer tablets to newly enrolled patients. Added the restriction that Section 8.10 does not apply in Japan. Additional minor changes were made as outlined below.

Applicable Section(s)	Description of Change(s)
**Rationale:** Switch of softgel capsules to tablets is incorporated. Vitamin E is not included in the tablet formulation.	

Applicable Section(s)	Description of Change(s)
Throughout	Changed “softgel capsules” or “capsules” to “tablets.”
Section 5.3.1 ARN-509/Matched Placebo; Section 5.3.2 Packaging, Storage, and Labeling	Removed information on formulation, packaging, and storage of the softgel capsules. Replaced with appropriate wording for the tablets.
Section 5.3.1 ARN-509/Matched Placebo; Section 6.2, Restricted Therapies; Appendix 5	Removed Vitamin E information, no longer applicable with the tablet formulation.
Section 5.3.3 Study Drug Administration	Added a new table showing the conversion from capsule to tablet and clarifying that the conversion is 2:1. Provided timing for the switch from the softgel capsules to tablets. Revised Table 2.
**Rationale:** The twice daily dosing is no longer relevant for the tablet formulation.	
Synopsis Dose and Route of Administration; Section 5.3.4 Cycle Management; Section 5.3.5 Dose Modifications	Removed the twice daily dosing option. Removed wording on dose modification due to gastrointestinal discomfort.
**Rationale:** Revised treatment compliance to account for capsules or tablets	
Section 5.5 Measures of treatment compliance; Schedule of Activities (Footnote 8)	Changed from “capsules” to “capsules or tablets.” Revised compliance to account for the difference in number of capsules versus tablets.
**Rationale:** The Statistical Analysis Plan will include additional analyses for safety and for the primary endpoint by formulation subgroup.	
Section 13.2.1 Analysis of Primary Endpoint	Added the following sentence: Additional analyses by formulation subgroups will be performed for the MFS endpoint as described in the Statistical Analysis Plan.
Section 13.3.1 Analysis of Adverse Events	Added the following sentence: An additional analysis by formulation subgroups will be performed as outlined in the Statistical Analysis Plan.
**Rationale:** The population PK analysis will include an additional analysis to explore the effect of the formulation switch.	
Section 13.4.4 Population Pharmacokinetics (PopPK) Analysis	Added the following wording: “the effect of the formulations will also be explored in the covariate analysis.”

Applicable Section(s)	Description of Change(s)
**Rationale:** To clarify that patients who experience loco-regional tumor progression should continue on study drug until documentation of distant metastastic disease. Local therapies such as surgery or radiation are allowed with study treatment. This was not clearly stated in the protocol.	
Section 6 Concurrent Medications	Added the following:<br>Salvage radiation for locoregional pelvic disease and surgical procedures (eg, transurethral resection of the prostate [TURP], urethral and ureteral stent placement) to treat localized progression or symptoms are allowed. Patients receiving these therapies may continue on study drug.
**Rationale:** To incorporate a local restriction in Japan into the global protocol in order to avoid the need for a separate country-specific protocol. The Sponsor will not provide abiraterone acetate as subsequent therapy. The criteria outlined in Section 8.10 are not applicable to sites in Japan.	
Section 8.10 Subsequent Therapy with Abiraterone Acetate; Schedule of Activities (Footnote 3)	Due to local restrictions in Japan, the Sponsor will not provide abiraterone acetate and the criteria outlined in this section do not apply.
**Rationale:** It is not necessary to administer study drug within 1 hour onsite after the trough PK sampling.	
Section 7.3 Pharmacokinetic Measurements; Section 8.4 Day 1 of Cycles (±2 days); Schedule of Activities (Footnote 20)	Removed the requirement that the PK sample be taken up to 1 hour prior to administering the study drug in the clinic.
**Rationale:** Patients who have a delay of study drug administration for ≥28 days due to toxicity will no longer automatically meet the criteria for discontinuation from the study.	
Section 5.3.5 Dose Modifications; Section 10 End of Treatment	Revised to indicate that subjects may discontinue study treatment if delayed more than 28 days due to toxicity and must be discussed with the Sponsor.
**Rationale:** Changes were made to improve clarity or correct errors	
Section 4.1 Inclusion Criteria; Section 4.2 Exclusion Criteria; Section 6 Concurrent Medications; Section 6.2 Restricted Therapies; Section 8.2 Screening (within 35 Days of Randomization); Section 8.9 Survival Follow-Up; Schedule of Activities	Minor changes were made to following eligibility criteria: Inclusion #3, 11, and 13; Exclusion #8 and #9. In Section 6.2, the bullet describing corticosteroid therapy was made consistent with other sections. Revised the wording in the last bullet of Section 8.2. Clarified in last bullet of Section 8.9 that disease progression refers to distant metastasis. Removed 1.7 nM for testosterone concentration.<br>Corrected error of footnote for biomarkers in the Schedule of Events and revised Footnote 6 to match revision in Section 8.2.

<u>Amendment INT-5</u> (1 July 2014)

This amendment is considered to be substantial based on the criteria set forth in Article 10(a) of Directive 2001/20/EC of the European Parliament and the Council of the European Union

The overall reason for the amendment: The overall reason for the amendment is to incorporate a time cutoff for the earliest prostate specific antigen (PSA) values used in the calculation of PSA doubling time (PSADT).

NOTE: Amendment INT-4 was never implemented and has been superseded by Amendment INT-5. The only change that affects study conduct between Amendments INT-5 and INT-4 is the addition of a 24 month collection time period during which PSA values used to calculate the PSADT can be obtained. This change was made shortly after finalization of the previous amendment. Please refer below to the table of changes for Amendment INT-4. All changes have been incorporated, as appropriate, into the protocol text.

Applicable Section(s)	Description of Change(s)
**Rationale:** The previous amendment did not include information on a timeframe for PSA values that should be used for the calculation of PSADT. A collection period during which PSA values used to calculate the PSADT can be obtained, is now specified. Therefore, this amendment will incorporate a cutoff that allows inclusion of PSA values collected within 24 months prior to the subject's randomization.	
4.1 Inclusion Criteria, Inclusion Criterion #1	Simplified Criterion #1 and referred to Section 5.1 for details on timing and calculation of PSADT.<br>Revised the criterion to read:<br>Histologically or cytologically confirmed adenocarcinoma of the prostate without neuroendocrine differentiation or small cell features with high risk for development of metastases, defined as PSADT ≤ 10 months. PSADT is calculated using at least 3 PSA values obtained during continuous ADT (see Section 5.1).
5.1 Randomization	Added the requirement for PSA values to be within 24 months prior to randomization with the specification that at least 3 values be used. Clarified that the earliest PSA value and all subsequent PSA values be entered into the Interactive Voice Randomization System (IVRS).
**Rationale:** Minor revision for consistency with Janssen Research & Development protocol template to numbering of eligibility criteria.	
4.1 Inclusion Criteria, Inclusion Criteria #1; #5, and #6	Revised the numbering to incorporate the current template style.
**Rationale:** Remove the inclusion of the number of sites as this information is not necessary in the protocol and is subject to change..	
Synopsis, Number of Sites, 3.1 Study Overview and Rationale	Removed the information on the number of sites from the synopsis and study overview.

Amendment INT-4 (16 June 2014)

This amendment is considered to be substantial based on the criteria set forth in Article 10(a) of Directive 2001/20/EC of the European Parliament and the Council of the European Union

The overall reason for the amendment: The overall reason for the amendment is to incorporate investigator feedback for Inclusion criteria #1 and 2 in order to clarify the definitions of prostate specific antigen doubling time (PSADT) and castration-resistance so that the intended homogenous and high-risk patient population is enrolled. Section 5.1 is also being revised to reflect the change to Inclusion Criterion #1. The addition of an optional prescreening period is being incorporated to allow investigators time to obtain the required number of prostate-specific antigen (PSA) values for meeting the study eligibility criteria.

Applicable Section(s)	Description of Change(s)
**Rationale:** The window of requiring prostate specific antigen (PSA) values collected within 6 months prior to randomization for calculation of the PSADT has the unintended consequence of excluding patients who are otherwise eligible, but who may have had one or more of the required PSAs collected outside the 6 month window. Currently, we are asking sites to continue to monitor and collect more recent PSAs from these patients; this delay increases the risk of screen fails as these patients are at high risk for metastasis. This requirement window has been removed from Inclusion Criterion #1 and a reference to Section 5.1 of the protocol, which details the calculation of PSADT for eligibility, has been added.	
4.1 Inclusion Criteria, Inclusion Criterion #1	Revised the criterion to read:<br>Histologically or cytologically confirmed adenocarcinoma of the prostate without neuroendocrine differentiation or small cell features with high risk for development of metastases, defined as PSADT ≤ 10 months. PSADT is calculated using at least 3 PSA values obtained during continuous ADT. The first and last PSA values used in the calculation must be separated by at least 8 weeks (see Section 5.1).
5.1 Randomization	Removed the requirement for 3 PSA values to be within 6 months prior to randomization. For consistency with the eligibility criteria the description of PSADT calculation is based on "at least" 3 PSA values. Also added the sentence that the first and last PSA values used in the calculation must be separated by at least 8 weeks.
**Rationale:** Inclusion Criterion # 2 requires that castration resistant prostate cancer is defined as 3 *consecutive* rises of PSA. This is not consistent with the Prostate Cancer Clinical Trials Working Group 2 (PCWG2) criteria,[24] and has the unintended consequence of excluding patients who are otherwise eligible, but who have had one or more PSA in a consecutive sequence not rise although there may be 3 rises in total. Currently, we are asking sites to continue to monitor and collect more PSAs until the three *consecutive* rises are met; this delay increases the risk of screen fails as these patients are at high-risk for metastasis. The requirement for two 50% increases of PSA above the nadir is also considered too stringent and is not consistent with the PCWG2 criteria.[24]	
4.1 Inclusion Criteria; Inclusion Criterion #2	Revised the criterion to read:<br>Castration-resistant prostate cancer demonstrated during continuous ADT, defined as 3 PSA rises, at least 1 week apart, with the last PSA > 2 ng/mL.

Applicable Section(s)	Description of Change(s)
**Rationale:** The washout period following anti-androgen may be longer than 4 weeks depending on the anti-androgen. Clarified that the washout period must be at least 4 weeks.	
4.1 Inclusion Criteria; Inclusion Criterion #5	Revised the criterion to read:<br>Patients who received a first generation anti-androgen (e.g., bicalutamide, flutamide, nilutamide) must have at least a 4-week washout prior to randomization AND must show continuing disease (PSA) progression (an increase in PSA) after washout.
**Rationale:** Sampling for PSA may not be frequent enough at some sites. In order to ensure eligible patients are not "missed," the patient may be asked to participate in the optional Prescreening Phase and sign a prescreening informed consent. The prescreening PSA evaluations will be performed by a local laboratory. As no study medication is being introduced at this point, only collection of serious adverse events related to the PSA blood draw will be collected.	
3.1 Study Overview and Rationale; 7.2.1 PSA;<br>8.1 Prescreening;<br>9.2.1 SAE Reporting;<br>9.2.2 Non-Serious AE Reporting;<br>Appendix 1:Schedule of Events; Footnote 1, new Footnote 10; new Footnote 18.	Added the prescreening details to appropriate sections of the protocol body and Appendix 1. For clarity, added a separate footnote for PSA evaluations to Appendix 1. Added a new section (8.1 Prescreening). Added Footnote 18 to clarify that only SAEs related to the PSA blood draw will be collected.<br>All other footnotes were renumbered accordingly.<br>Section 9.2.1 was modified to add the SAE reporting requirement during the Prescreening Period.<br>Section 9.2.2 was modified to clarify the reporting period and an error was corrected (strikethrough text removed):<br>Adverse events should be recorded on the AE CRF from the time the patient has signed the informed consent at screening (see Section 8.2) ~~taken at least one dose of study drug~~ until 28 days after the last dose of study drug.
**Rationale:** The description of androgen deprivation therapy as ADT/post -surgery castration is redundant, wording was revised, as applicable.	
Throughout the protocol	ADT/post-surgical castration was revised to ADT
**Rationale:** The term radiographic progression-free survival or rPFS originated with the ZYTIGA COU-AA-302 study and has specific criteria for the determination of radiographic progression that is not applicable to the patient population in this study. To avoid confusion, the term radiographic PFS or rPFS is being revised to progression-free survival or PFS.	
Throughout the protocol	Revised radiographic progression-free survival or radiographic PFS or rPFS terminology to progression-free survival or PFS.
**Rationale:** Aminoglutethimide was erroneously included under Inclusion Criterion # 6 and should be under Exclusion Criterion #4.	
4.1 Inclusion Criteria, Inclusion Criterion# 6<br>4.2 Exclusion Criteria, Exclusion Criterion# 4.	Moved aminoglutethimide to the Exclusion Criterion # 4 after ketoconazole.

Applicable Section(s)	Description of Change(s)
**Rationale:** Update to pregnancy section for consistency with the Janssen Template wording	
9.1.6 Exposure During Pregnancy	Updated with Janssen template wording
**Rationale:** Clarified timing of submitting the FFPE archival tumor samples or slides.	
Footnote 21	Updated the footnote to indicate that FFPE samples can be submitted anytime starting from Cycle 1 Day 1.
**Rationale:** The number of sites is planned to increase.	
Synopsis, Number of Sites; 3.1 Study Design Overview and Rationale	Increased number of sites from 330 to 400.
**Rationale:** Fasting period requirement for the ventricular repolarization study is very long, subjects will be now be allowed to have an approved snack.	
Appendix 8, Additional Inclusion Criteria	Added that the subject can have an approved snack:<br>Must agree to fast at least 3 hours prior to dose and continue fasting (except for approved snack) until completion of the 4 hour post dose assessments on Cycle 1 Day 1 and Cycle 3 Day 1.
**Rationale:** Minor errors or clarifications were noted	
Throughout the protocol	Minor grammatical, typographical or clarification changes were made.

Amendment 3 (11 March 2014)

This amendment is considered to be substantial based on the criteria set forth in Article 10(a) of Directive 2001/20/EC of the European Parliament and the Council of the European Union

The overall reason for the amendment: The overall reasons for the amendment are the following: to incorporate modifications to the statistical analysis plan for the secondary endpoints; incorporate provision of abiraterone acetate as subsequent therapy for eligible patients; addition of exploratory biomarkers; reduction of population pharmacokinetic sample collection and modification of the population PK analysis, and modification of the patient-reported outcomes analysis. Additional changes were also implemented based on Health Authority and Investigator feedback. Updated nonclinical and clinical data were also incorporated.

Applicable Section(s)	Description of Change(s)
**Rationale:** Statistical analysis of the secondary endpoints was modified and will incorporate multiple testing method in agreement with the FDA proposal.	
Synopsis, Secondary Objectives and Endpoints, Other Objectives and Evaluations; 2.2 Secondary Objectives; 2.3 Other Objectives; 3.2.2 Secondary Endpoints; 3.2.3 Other Evaluations; Synopsis, Statistical Analysis Plan and Rationale for Number of Patients; 13.2.2 Analysis of Secondary Endpoints	Overall survival (OS) is no longer a separate key secondary objective or endpoint. Removed "key" and "other" secondary endpoints sections and regrouped into 2 groups of "Secondary Endpoints." Group 1 includes: [redacted]. Group 2 includes [redacted].<br>Added "Other " objectives and evaluations (see additional rationale below).<br>The statistical testing of the 2 groups of secondary endpoints will be performed by allocating [redacted] to Group 1 and [redacted] for the [redacted] endpoint in Group 2 with an overall familywise type I error rate of 0.05. Details of the testing procedure will be provided in the statistical analysis plan.
**Rationale:** Modification of the interim analysis of OS, 2 interim analyses and 1 final analysis are now planned.	
Synopsis, Statistical Analysis Plan and Rationale for Number of Patients; 13.5 Interim Analysis; 13.6 Determination of Sample Size; Synopsis, Duration of Patient Participation and Duration of Study; 15.6 End of Trial Notification in All Participating Countries	Two interim analyses and 1 final analysis are planned for the OS endpoint. With the decrease in alpha from [redacted] to [redacted], the number of death events is now [redacted] (final analysis). At the time of the first interim analysis, approximately [redacted]) of the death events will be observed with an alpha spend of [redacted]. The second interim analysis will be performed after observing approximately [redacted]) of the death events with a cumulative alpha spend of [redacted]. The actual alpha spent for the 2 interim analyses will be based on the number of death events observed at the time of the analyses.
**Rationale:** Added "Other" Objectives and Evaluations	
Synopsis, Other Objectives; Other Evaluations; 2.2 Other Objectives; 3.2.3 Other Evaluations	Also added PSA response and time to PSA progression, analyses are included in Section 13.4.1, but they were not listed as evaluations in the previous versions of the protocol.<br>Added medical resource utilization and exploratory biomarkers<br>Previous endpoints listed as "Other Secondary" Objectives and Evaluations were renamed "Other" Objectives and Evaluations.

Applicable Section(s)	Description of Change(s)
**Rationale:** Provision of abiraterone acetate as subsequent therapy for those patients who meet the protocol-specified eligibility criteria.	
8.9 Subsequent Therapy with abiraterone acetate; 9.2.1 Serious Adverse Event Reporting; 10 End-of-Treatment	Addition of a new section (8.9) incorporating the provision of abiraterone acetate by the Sponsor.
**Rationale:** Population pharmacokinetic (PK) sampling and analysis were modified. The number of sparse samples for population PK analysis was decreased as steady state of ARN-509 and metabolite ARN000308 can be reached within approximately 3 months. Pharmacokinetic samples collected up to 24 months should be sufficient to characterize the kinetics of both compounds, and no relevant information would be obtained from later timepoints. Analysis of the metabolite ARN000066 is being removed as this is a minor metabolite in humans with minimal activity.	
7.3 Pharmacokinetic Measurements; 8.2 Cycle 1 Day 1; 8.3 Day 1 of Cycles n..; Appendix 1; Footnote 17; 13.4.3 Population PK Analysis; Appendix 8	Removed analysis for the ARN000066 metabolite. Removed sample collection from Cycle 36 onwards. Sparse PK samples will not be taken from patients participating in the ventricular repolarization substudy (Appendix 8).
**Rationale:** Exploratory biomarker research was added to study the potential mechanisms of resistance to ARN-509. Based on limited published data, approximately 10% of patients with late stage disease may develop resistance over the course of treatment. There is not sufficient data to indicate whether resistance develops at a different frequency in patients with NM-CRPC. In the original plan proposed to the CHMP and FDA, a sample size of 300 patients was considered sufficient. Based on follow-up advice from the FDA and assuming lower frequencies of mutation emergence in the NM-CRPC, the number of samples was increased from 300 to 400 in order to have sufficient power for correlative analysis based on calculation on a sliding scale. To capture the F876L mutation status in patients progressing in the later course of treatment, the collection of samples was modified to include Day 1 of Cycle 36, the Day 1 of Cycle 6 assessment was removed.	
7.5 Exploratory Biomarkers; 13.1 Analysis Populations; 13.4.4 Exploratory Biomarkers; References; 14.7 Patient Confidentiality	Section 7.5 was added to explain background and rationale for the exploratory biomarker research. A new analysis population was added. Long-term storage details for biomarker samples was incorporated into Section 14.7.
8.6 End-of-Treatment Visit; Appendix 1 and added Footnote 19.	Sample collection details for exploratory biomarkers were incorporated. Archival tumor formalin-fixed paraffin embedded (FFPE) tumor blocks or tumor slides will be retrieved from consenting patients on Cycle 1 Day 1, blood samples will be collected before dosing on Day 1 of Cycles 1, 12, 18, 24, 36, and end-of-treatment. Footnote 19 indicates blood volumes collected during each cycle.

Applicable Section(s)	Description of Change(s)
**Rationale:** The previous patient-reported outcomes analysis incorporated a 16-point improvement in the Functional Assessment of Cancer Therapy-Prostate (FACT-P) total score as the definition for a health-related quality of life improvement. Improvement of the total score is not applicable for this study population. The magnitude of a clinically meaningful change was redefined.	
13.4.2 Health-Related Quality of Life and Prostate Cancer-Specific Symptoms Analysis	A 10-point change in the FACT-P total score is considered clinically meaningful. Therefore, any patient experiencing a 10-point decrement in FACT-P total scores from baseline will be considered to have experienced clinically meaningful deterioration in functional status.
**Rationale:** The completion of the PRO questionnaires was reduced after Cycle 13 to every other cycle to relieve the burden to the patient and for consistency with other Sponsor protocols.	
7.4 Patient-Reported Outcomes; 8.3 Day 1 of Cycles n…; Appendix 1, Footnote 18	Reduced the collection of FACT-P and EQ-5D questionnaires after Cycle 13 to every other cycle.
**Rationale:** Added second progression-free survival (PFS2) as an evaluation per agreement with health authorities.	
Synopsis, Other Evaluations; 3.2.3 Other Evaluations; 8.8 Survival Follow-Up; 13.4.1 Second progression-free survival (PFS2); Appendix 1	This endpoint is defined as the time from randomization to second documentation (progression on first subsequent therapy) investigator-assessed disease progression (PSA, radiographic, symptomatic, or any combination) or death from any cause.
**Rationale:** Inclusion criterion was modified for consistency with Section 5.1 of the protocol and based on Investigator feedback.	
4.1 Inclusion Criteria, Criterion #1	Clarified determination of PSADT
**Rationale:** Inclusion of patients with documented Gilbert's disease is allowed	
4.1 Inclusion Criteria, Criterion #11	Added exception to elevated bilirubin in cases of documented Gilbert's disease.
**Rationale:** Bone sparing therapies indicated for the treatment of skeletal events due to bone metastases are not allowed as concurrent therapy.	
4.1 Inclusion Criteria, Inclusion Criterion #4; 4.2 Exclusion Criteria, Exclusion Criterion 8	Clarified inclusion Criterion #4 to allow bone sparing therapies for the treatment of osteoporosis if on stable doses for at least 4 weeks prior to randomization. Modified Exclusion Criterion #8 to exclude bone sparing therapy (eg., denosumab [Xgeva®] indicated for the treatment of skeletal events due to bone metastases.

Applicable Section(s)	Description of Change(s)
**Rationale:** Exclusion criterion #1 was modified to clarify that a history of distant metastases is also exclusionary.	
4.2 Exclusion Criteria, Exclusion Criterion #1	Presence of distant metastases confirmed by blinded independent central review (BICR), including CNS and vertebral or meningeal involvement, or history of distant metastases. Exception: pelvic lymph nodes < 2 cm in short axis (N1) located below the iliac bifurcation are allowed
**Rationale:** Inclusion Criterion #5 was modified to clarify the criteria for eligibility following previous anti-androgen treatment to include the 4 week washout period prior to randomization and documented prostate specific antigen (PSA) progression.	
4.1 Inclusion Criteria; Inclusion Criterion #5	Patients who received a first generation anti-androgen (e.g., bicalutamide, flutamide, nilutamide) must have a 4-week washout prior to randomization AND must show continuing disease (PSA) progression (an increase in PSA) once they are off the anti-androgen.
**Rationale:** Inclusion Criteria #6 to clarify that prior estrogen treatment discontinuation is irrespective of dose used.	
4.1 Inclusion Criteria, Inclusion Criterion #6	At least 4 weeks must have elapsed from the use of 5-α reductase inhibitors (e.g., dutasteride, finasteride, aminoglutethamide), estrogens (irrespective of dose used), and any other anti-cancer therapy prior to randomization, including chemotherapy given in the adjuvant/neoadjuvant setting (e.g., clinical trial)
**Rationale:** Clarified that patients with a history of uncontrolled blood pressure are eligible if controlled with anti-hypertensive treatment.	
4.2 Exclusion Criteria, Exclusion Criterion #9	Uncontrolled hypertension (systolic blood pressure ≥160 mmHg or diastolic BP ≥100 mHg). Patients with a history of uncontrolled hypertension are allowed provided blood pressure is controlled by anti-hypertensive treatment
**Rationale:** Gonadotropin releasing hormone analogs (GnRHa) (or surgical castration) for patients is required as a concomitant therapy and should be continuous throughout treatment.	
Inclusion Criterion #3; 3.1 Study Design Overview and Rationale; 6 Concurrent Medications	Revised Inclusion Criterion #3 and incorporated text in relevant sections throughout the protocol to reiterate the requirement for GnRHa treatment (or surgical castration) for all patients (both treatment groups).

Applicable Section(s)	Description of Change(s)
**Rationale:** Per Sponsor policy adverse events (AEs) should be collected from the signing of the informed consent form.	
8.1 Screening, 8.2 Cycle 1 Day 1; 8.3 Day 1 of Cycles n; 8.6 End-of-Treatment Visit; 8.7 Safety Follow-up; 9.1.1 Adverse Events; 9.2.2 Non-serious AE Reporting; Appendix 1	Modified these sections to include collection of AEs from the signing of the informed consent form and not at the time of the first dose of study drug. General addition, where needed, for collection of AEs at all times from informed consent up to 28 days after the last dose of study drug.
**Rationale:** Analysis of vital signs and concomitant medications should be under safety evaluations and not under other evaluations	
13.3.3 Vital Signs Analysis; 13.3.4 Concomitant Medications	Moved from the Other Evaluations section to Safety Evaluations Section
**Rationale:** Clarification that patients are expected to be treated with local standard of care before enrollment. Rescreening information for screen failures was added.	
4 Patient Selection	It is expected that all patients will be treated according to local standard of care, including radiation therapy if needed for local disease, prior to enrolling.<br>Patients considered screen failures may be subsequently rescreened. Rescreening must be discussed with and approved by the Sponsor on a case by case basis. Patients who are determined to be eligible for the study after rescreening must sign a new ICF and be assigned a new patient number.
**Rationale:** Timing of tumor assessments and collection of blood for thyroid stimulating hormone (TSH), testosterone, and fasting lipid panel was modified from every 16 weeks starting from the date of randomization to starting from Cycle 1 Day 1. Urinalysis was removed as a required laboratory assessment.	
8.4 and 8.5 Every 16 weeks from Cycle 1 Day 1; Appendix 1	Change timing of the start of assessments from date of randomization to Cycle 1 Day 1. A separate column for these assessments was added to the Schedule of Events.
**Rationale:** Incorporated the Danish Health Authority request for inclusion of detailed methods of contraception and required duration of contraceptive methods or donation of sperm after discontinuation of the study drug.	
6.3 Life Style Guidelines	Added more detailed information for patients and partners of childbearing potential. Also added that effective contraception is required during the study and for 3 months after the last dose of study drug. Donation of sperm is not allowed during the study and for 3 months following the last dose of study drug.

<table>
<tr><th>Applicable Section(s)</th><th>Description of Change(s)</th></tr>
<tr><td colspan="2">Rationale: Updated nonclinical data</td></tr>
<tr><td>Synopsis; 1.1.2 Preclinical Development Overview</td><td>Revised and updated in vitro metabolism data. Summarized the final 13-week rat and dog GLP toxicity results, removed the dog 28-day toxicity summary. Updated the genotoxic, clastogenic, and phototoxicity data for ARN-509 and the metabolites (ARN000308 and ARN000066).</td></tr>
<tr><td colspan="2">Rationale: Updated clinical information</td></tr>
<tr><td>1.13 Overview of Clinical Studies</td><td>Updated with general overview of ongoing clinical studies and data from Study ARN-509-001.</td></tr>
<tr><td colspan="2">Rationale: The protocol had no information regarding product quality complaint handling. Added a Product Quality Complaint section to the protocol</td></tr>
<tr><td>9.2.4 Product Quality Complaint Handling</td><td>Added new section per Sponsor template.</td></tr>
<tr><td colspan="2">Rationale: Patient initials will not be used for any documents in this study.</td></tr>
<tr><td>14.1 Data Collection Instruments; 14.7 Patient Confidentiality; 15.1 Investigator Responsibilities</td><td>All reference to patient initials was removed.</td></tr>
<tr><td colspan="2">Rationale: Per request of Danish Health Authority, investigator responsibilities section was updated to include a Declaration of Helsinki statement. Wording consistent with the current Sponsor template was incorporated.</td></tr>
<tr><td>15.1 Investigator Responsibilities; 15.4 Informed Consent Form</td><td>Incorporated Sponsor template language, which meets the requirements of the HA request.</td></tr>
<tr><td colspan="2">Rationale: Added instruction that subsequent ECG readings should be collected using same position as the baseline reading.</td></tr>
<tr><td>7.2.2 Electrocardiogram (ECG)</td><td>Subsequent ECG readings should be collected with the patient in the same position (e.g., fully supine or semi-recumbent).</td></tr>
<tr><td colspan="2">Rationale: Protocol violation forms do not exist.</td></tr>
<tr><td>11 Protocol Violations</td><td>Removed paragraph explaining that protocol violation forms would be completed and archived. There are no such forms.</td></tr>
</table>

Applicable Section(s)	Description of Change(s)
**Rationale:** At the time for the final analysis OS, a decision will be made regarding whether or not an amendment of the protocol may be needed.	
Synopsis, Duration of Patient Participation and Duration of Study; 15.6 End of Trial in All Participating Countries	Removed wording indicating that the protocol may be amended to minimize the number of assessments.
**Rationale:** Time from screening to randomization was increased from 28 days to 35 days because the required 28 day washout period for eligibility was not allowing enough time to complete screening assessments in some patients. The time from randomization to Cycle 1 Day 1 was also increased by 1 day from 3 days to 4 days to accommodate unforeseen delays due to holidays, weather, etc.	
Appendix 1; 8.1 Screening Within 35 Days of Randomization; 8.3 Cycle 1 Day 1	Change timing from screening to randomization to 35 days, also revised the timing from randomization to Cycle 1 Day 1 from 3 days to 4 days
**Rationale:** Laboratory assessments deemed nonessential for safety or efficacy were removed	
Appendix 2	Removed phosphorus , total protein, chloride, uric acid, and lactate dehydrogenase.
Appendix 1; Appendix 2; 7.2 Clinical Laboratory Measurements; 8.1 Screening within 35 Days of Randomization; 8.4 Every 16 Weeks Starting From Cycle 1 Day 1 …;8.6 End-of-Treatment Visit	Removed collection of urine samples and urinalysis.

Applicable Section(s)	Description of Change(s)
**Rationale:** Prostate-specific antigen (PSA) was listed under the wrong column in the Appendix 2 table	
Appendix 2	Moved PSA measurements to the every cycle column for consistency with the Schedule of Events.
**Rationale:** The approximate number of sites has increased from 250 to 330.	
Synopsis, Number of Sites; 3.1 Study Overview and Rationale	Revised from approximately 250 in the synopsis to 330 and added this information to Section 3.1.
**Rationale:** Additional eligibility criteria for participation in the QT substudy were incorporated. Holter monitoring assessment included at Screening. Informed consent requirement was previously not included in the Appendix 8 Schedule of Activities for Screening.	
Appendix 8	Screening assessment incorporated with the addition of the Holter monitoring and requirement for signing of informed consent<br>Added required fasting conditions for participation in the substudy, exclusion of patients if they required a dose reduction at any time from Cycle 1 Day 1 through Cycle 3 Day 1.<br>Made a minor wording to the sample size determination: A sample size of at least 100 patients will ensure that at least 60 patients treated with ARN-509 will be enrolled on the substudy and 60 patients will provide at least 98.7% power to detect a true effect of 10 milliseconds (msec) change from baseline considering only the active group.<br>Informed consent was added to Table 1 of Appendix 8.
**Rationale:** Inclusion of all criteria in the synopsis was unnecessary.	
Synopsis, Enrollment Criteria	Replace the individual criteria with a summary.
**Rationale:** Clarified that pomegranate is excluded (not just juice)	
Section 4.2 Exclusion Criteria, Criterion #8; Appendix 5	Removed the word "juice."
**Rationale:** Added that a patient eligibility form must be completed and submitted to the medical monitor before randomization	
8.1 Screening Within 35 Days of Randomization	Added a bullet to Section 8.1. Also added to the subsequent bullet that the medical monitor must confirm eligibility before randomization
**Rationale:** In order to avoid a protocol amendment in the case where contact information may change, the contact information was removed.	
9.1.2 Serious Adverse Events	Removed contact information, which will be provided separately.

Applicable Section(s)	Description of Change(s)
**Rationale:** The Schedule of Events was confusing to follow and not well organized	
Appendix 1	Revised the entire table and footnotes.
**Rationale:** Modified wording throughout the document for conciseness or clarity.	
Throughout the protocol	Included were modifications to the Schedule of Activities to simplify the presentation ensure consistency with the body of the protocol, and remove redundant footnotes. The Independent Data Monitoring Committee section (Section 12) was condensed.
**Rationale:** Minor errors were noted	
Throughout the protocol	Minor grammatical, formatting, or spelling changes were made.

1. BACKGROUND

Prostate cancer is the second most frequently diagnosed cancer and the sixth leading cause of cancer death in males, accounting for 14% (903,500) of the total new cancer cases and 6% (258,400) of the total cancer deaths in males worldwide.[9] Treatment aimed at eradicating the primary tumor, typically with surgery or radiation, is unsuccessful in ~30% of men, who develop recurrent disease that usually manifests first as a rise in plasma prostate-specific antigen (PSA) followed by metastasis to distant sites.[29] Given that prostate cancer cells depend on androgen receptor (AR) for their proliferation and survival, the standard treatment for patients with recurrent disease is androgen deprivation therapy (ADT) with a gonadotropin releasing hormone analog (GnRHa) with or without an anti-androgen.

Treatment results with ADT are generally predictable: a decline in PSA followed by tumor regression, a period of stability in which the tumor does not proliferate and PSA remains stable, followed by rising PSA and regrowth as a castration-resistant disease. Nearly all men with progressive prostate cancer eventually develop castration-resistant disease. Prostate cancer progression despite castrate levels of testosterone represents a transition to a lethal disease stage.

Molecular profiling studies of castration-resistant prostate cancer (CRPC) commonly show increased AR gene expression.[23] The increased AR levels are sufficient to confer resistance to anti-androgen therapy in mouse models, shorten tumor latency and confer agonist properties to first generation AR antagonists, such as bicalutamide or flutamide.[2] The potential for agonist activity by these approved anti-androgens in the setting of increased AR expression is a potential liability, best illustrated by the observation of tumor regression and declines in PSA following discontinuation of either of these AR antagonists, the so-called anti-androgen withdrawal syndrome.[11] Collectively, these findings implicate increased AR levels as one mechanism of drug resistance. They also suggest that drugs retaining antagonism and not displaying agonism in cells over-expressing AR levels might be useful therapeutically.

ARN-509 (JNJ-56021927; apalutamide, hereafter referred to as apalutamide) is a next generation AR antagonist that has been developed to overcome the potential therapeutic deficiencies of first-generation AR antagonists (e.g., bicalutamide).

1.1 APALUTAMIDE

Apalutamide is an orally available, potent and selective AR antagonist that acts by inhibiting the action of androgen, nuclear translocation of the AR and DNA binding to androgen response elements and unlike bicalutamide, it exhibits no significant agonist activity in AR-overexpressing prostate cancer cells.[3]

Complete information for apalutamide (JNJ-56021927) can be found in the Investigator’s Brochure, the safety reference document for this study.

1.1.1 Molecular Formula and Chemical Class

Apalutamide drug substance is a white to off-white crystalline solid.

Chemical Name: (4-[7-(6-Cyano-5-trifluoromethylpyridin-3-yl)-8-oxo-6-thioxo-5,7-diazaspiro[3.4]oct-5-yl]-2-fluoro-*N*-methylbenzamide)

Chemical Structure:

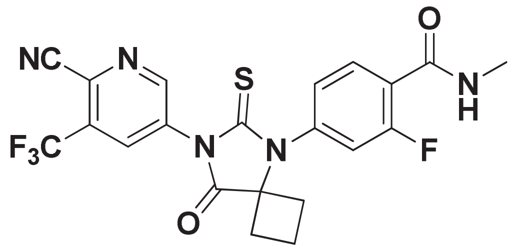

Molecular Formula: $C_{21}H_{15}F_4N_5O_2S$

Molecular Weight: 477.44

1.1.2 Pre-Clinical Development Overview

The mechanism of action of apalutamide is through antagonism of androgen action and inhibition of AR nuclear translocation and DNA binding to androgen response elements, a mechanism that is distinct from the first generation anti-androgen, bicalutamide. Unlike bicalutamide, it shows no significant agonist properties in an in vitro model of CRPC (e.g., AR-over-expressing prostate cancer cells; LNCaP/AR cells).[3] Gene transcription of the androgen-driven genes, PSA and TMPRSS2, is inhibited by apalutamide and results in concentration-dependent reduction of these protein levels in vitro. Apalutamide was also shown to reduce proliferation of CRPC cells as well as increase apoptosis and necrosis in vivo. These effects are supported by the anti-tumor activity of apalutamide observed in murine tumor models of CRPC. In these models, apalutamide showed dose-dependent tumor growth inhibition and tumor regression that were superior to bicalutamide.[3] Figure 1 depicts the percent change in tumor volume and plasma concentrations (filled circles above waterfall plot) of bicalutamide and apalutamide on Day 28.

Figure 1 Tumor Growth Inhibition in Castration-resistant LNCaP/AR-Luc Xenograft Model After 28 Days of Treatment with Bicalutamide or apalutamide (ARN-509)

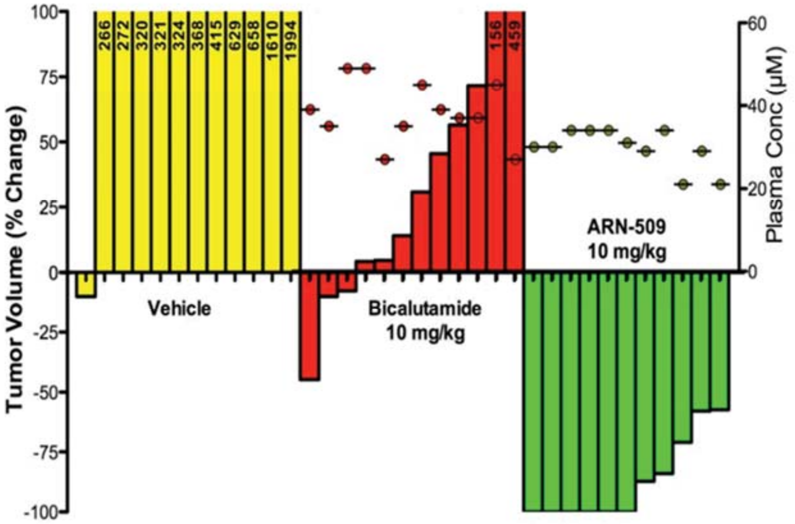

Apalutamide is a low clearance molecule with a moderate volume of distribution and high bioavailability (when dosed with a lipid-based formulation). Apalutamide was found to have a very low turnover when incubated for up to 120 minutes with rat, dog, and human liver S9 fraction and liver microsomes. The primary in vivo metabolites were formed by N-demethylation and amide hydrolysis in the rat and dog. In vitro, CYP3A4 may be partially involved in the metabolism of apalutamide.

Apalutamide and its primary metabolite ARN000308 (M3) are inducers of human CYP2B6 and CYP3A4 in hepatocytes at concentrations up to 30 µM. Apalutamide is a moderately potent inhibitor of human cytochrome P450 isoform CYP2C8 (IC_{50}=13.9 µM), but a weak inhibitor of the other major isoforms (IC_{50}>25 µM); M3 is also a weak inhibitor of CYP major isoforms (IC_{50}>25 µM).

Four metabolites have been identified with different proportions between species. All four were assessed for their on-target effects against the androgen receptor. Metabolite M1 was found to be essentially inactive as an AR antagonist, while metabolites M2 and M4 were approximately [redacted] less potent against AR than apalutamide. Metabolite M3 was the most potent AR antagonist, but was still [redacted] less potent than apalutamide. Metabolite M3 is considered the predominant metabolite, with a longer elimination half-life than apalutamide.

In vitro and in vivo studies to assess cardiovascular, CNS, and respiratory system effects of apalutamide did not reveal any concerns.

Single-dose and repeat-dose toxicology studies up to 13 weeks of dosing have been conducted in male Sprague Dawley (SD) rats and male Beagle dogs (repeat-dose studies only).

Acute administration of apalutamide at 1,000 mg/kg was well tolerated in SD rats, with no morbidity, mortality or significant effects on body weight or serum chemistry markers.

In repeat-dose toxicology studies, apalutamide was well tolerated at doses up to 100 mg/kg/day in the 13-weeks study in SD rats and 10 mg/kg/day in Beagle dogs. In male SD rats, lethality was observed at doses of 150 mg/kg/day and greater. The morbidity/mortality observed at these doses occurred within the first 5 days of dosing; however, animals that did survive at these higher doses, appeared to develop a tolerance for the test article with extended exposure. Clinical signs observed in the moribund animals were piloerection, hypothermia, breathing abnormalities, dehydration, and decreased activity. The cause of the morbidity/mortality in male rats could not be determined by pathologic examination. Key clinical pathology changes at doses of 150 mg/kg/day or greater included significant increases in cholesterol (greater than 200% from controls), decreases in erythrocytes, hemoglobin and hematocrit, and increases in reticulocytes, platelets, leukocytes, lymphocytes, basophils, and aPTT. The increase in cholesterol is attributed to the anti-androgen activity of apalutamide and is believed to be responsible for the stated hematologic changes. Examination of red blood cell morphology revealed changes that were consistent with excess cholesterol being transferred to the outer membrane of the erythrocytes, resulting in a mild hemolytic anemia. Pharmacologic effects were also observed in the male accessory sex organs (epididymides, prostate, seminal vesicles and to a lesser degree, the testes) at apalutamide doses as low as 50 mg/kg/day. Other target organs in the rat that were observed at apalutamide doses of 150 mg/kg/day or higher included adrenals (also at 50 mg/kg/day), liver, pituitary, thyroid, spleen, salivary glands, mammary gland, and stomach. With the exception of the salivary glands and stomach, the effects on those organs are also believed to be due to the anti-androgen effect of apalutamide and in many cases are specific to the physiology of the rat.

Once daily oral gavage dosing of apalutamide for 13 weeks was well tolerated in male rats up to 100 mg/kg/day, i.e. the highest dose tested. Pharmacologic changes characteristic of anti-androgen compounds were noted in the adrenal gland, pituitary gland, spleen, mammary gland, seminal vesicles, testes, prostate, and epididymides, while changes in the spleen and bone marrow correlated with a mild regenerative anemia.The 100 mg/kg/day dose level was considered to be the no observed adverse effect level (NOAEL) and was associated with steady-state (Day 91) plasma C_{max} and AUC_{0-24h} values of 30.1 µg/mL and 521 µg•h/mL, respectively, for the parent compound.

In male Beagle dogs, seizures necessitating humane euthanasia occurred at apalutamide doses of 25 mg/kg/day and greater, 7 to 14 days after dosing was initiated. Daily administration of 25 mg/kg/day of apalutamide resulted in tremors and seizures in 3 of 8 animals after 1 week of dosing. The average apalutamide concentration at the time of first observation of central nervous system (CNS) toxicity was determined to be 30.2 µg/mL, which was about 4-fold higher than the mean apalutamide steady-state C_{max} (7.55 µg/mL) at the Phase 3 dose of 240 mg/day measured during repeated dosing in subjects with CRPC. It is likely that the convulsive seizures observed in dogs at very high doses are the result of apalutamide's functional antagonism of the $GABA_A$ receptor. This is similar to what has been observed with other second generation AR antagonists.[6,18] The 10 mg/kg/day dose was considered to be the NOAEL in the 28-day study, and was associated with an apalutamide

C_{max} of 13.2 µg/mL and an AUC_{0-24} of 290 µg•h/mL. Other clinical pathology and target organ changes were limited to increases in cholesterol (up to 50% compared to controls) and effects on the epididymides, prostate and testes at all doses tested and attributed to the anti-androgen effect of apalutamide.

Once daily oral capsule administration of apalutamide for 13 weeks was well tolerated in male dogs up to 10 mg/kg/day, i.e. the highest dose tested. Gross and microscopic pathology changes and organ weight changes characteristic of anti-androgen compounds were noted in the pituitary gland, prostate, testes, and epididymides; these changes were reversible and were attributable to the expected pharmacologic effect of apalutamide. Based upon the lower body weight performance in the group receiving 10 mg/kg/day, the 5 mg/kg/day dose was considered to be the NOAEL. Corresponding steady-state (Day 91) plasma C_{max} and AUC_{0-24h} values were 10.3 µg/mL and 202 µg•h/mL, respectively, for the parent compound.

Preclinical studies have demonstrated the absence of genotoxic, clastogenic, and phototoxic properties for apalutamide and its pharmacologically active metabolite ARN000308 (M3). ARN000066 (M4), an inactive metabolite of apalutamide, tested negative in an in vitro bacterial reverse mutation (Ames) assay, but was weakly positive in an in vitro chromosome aberration test in human peripheral blood lymphocytes. However, the totality of nonclinical data supports the lack of an in vivo genotoxic potential of ARN000066 (M4).

1.1.3 Overview of Clinical Studies

In addition to Study ARN-509-003, apalutamide is also being evaluated in Phase I studies in healthy men and Phase I/II, Phase II, and Phase III studies in patients with prostate cancer.

Study ARN-509-001 is an ongoing Phase I/II study in patients with progressive advanced CRPC. In the Phase I portion of the study, 30 patients with mCRPC received at least 1 dose of apalutamide at escalating dose levels: 3 patients each at 30, 60, 90, 120, 180, 240, 390, 480 mg/day, and 6 patients at 300 mg/day.[19,20] Three subgroups are being evaluated in the Phase 2 portion (Group 1: NM-CRPC; Group 2: mCRPC without previous ketoconazole, abiraterone acetate, enzalutamide or chemotherapy [for mCRPC]; and Group 3: mCRPC post abiraterone acetate, no previous chemotherapy [for mCRPC]). All patients received apalutamide orally once daily, except those in the 300, 390, and 480 mg cohorts who received a twice-daily dosing regimen due to the higher pill burden at those levels. The results of the Phase 1 portion of the study from 30 patients with mCRPC demonstrated that treatment with apalutamide resulted in PSA declines at all dose levels tested. Eighteen subjects (60%) had a ≥50% or higher maximum decrease in PSA from baseline and 6 (20%) had ≥90% maximum decrease. Ten patients had measureable soft tissue disease at baseline; 5 (50%) subjects maintained stable disease response for more than 6 months, 1 (10%) subject experienced disease progression, and the remaining 4 subjects had indeterminate responses. Apalutamide was well tolerated, with only 1 dose-limiting toxicity (DLT) at the 300 mg dose level (Grade 3 treatment-related abdominal pain). The event lasted 6 days and resolved with dose interruption and subsequent dose reduction to 240 mg (120 mg twice daily). Three additional subjects were treated at the 300 mg dose level with no reported DLTs. No seizures were reported at any dose level. The maximum tolerated dose (MTD) was not determined. The PK profile is linear and dose-proportional.

Sixteen subjects participated in an evaluation of AR binding in vivo using 16β-[18F] fluor-α-dihydrotestosterone (FDHT) positron emission tomography (PET)/CT scan. Apalutamide treatment reduced FDHT uptake across all dose levels (30 to 390 mg dose levels). A plateau was reached at approximately 120 mg (with FDHT uptake near background) indicating saturation of AR binding. The mean plasma trough levels associated with this dose (2.5 µg/mL) were at the lower end of the range that produced tumor regression in the LNCaP/AR mouse model. Steady state plasma levels at the 240 mg dose level (3 to 6 µg/mL) were more in the range sufficient to elicit a tumor response in the mouse xenograft model. Therefore, the 240 mg daily regimen was chosen for Phase 2 and Phase 3 dosing.

Apalutamide was rapidly absorbed, with measurable plasma concentrations within 30 minutes after ingestion of a single oral dose of 1 to 16 soft gelatin capsules (total apalutamide dose, 30 to 480 mg). On average, peak plasma concentrations occurred 2 to 3 hours after administration in each dose group. The increases in plasma C_{max} values and in the area under the plasma concentration curve (AUC) were linear and dose proportional. Plasma apalutamide concentrations declined slowly, with a mean half-life value at steady-state of 4 days.

The percentage of patients with PSA reductions of ≥50% at 12 weeks were 91% for the Group 1, 88% for the Group 2, and 24% for Group 3. Similar data were observed after 24 weeks. The data available to date indicate that apalutamide shows durable PSA responses in NM-CRPC and early mCRPC (before treatment with abiraterone acetate or chemotherapy). Apalutamide also has activity in a subgroup of patients with mCRPC that have progressed on abiraterone actetate. For the NM-CRPC subgroup, the metastasis-free survival at 12 months was 87% and with a median follow-up of 15 months the time to PSA progression had not been reached.[28]

In the NM-CRPC subgroup (n =51), the most frequent treatment-related adverse events reported in >10% as of 30 November 2013 are fatigue (43%), diarrhea (31%), nausea (20%), increased thyroid stimulating hormone (12%), dysgeusia (12%), and hot flush 12%. Eight (16%) patients have discontinued the study due to adverse events. Seven (14%) patients have discontinued due to disease progression. No deaths were reported in this subgroup as of the safety cutoff.

Refer to the Investigator's Brochure for most up to date information.

2. STUDY OBJECTIVES

2.1 PRIMARY OBJECTIVE

- To demonstrate superiority in the metastasis-free survival (MFS) of men with high risk NM-CRPC treated with apalutamide versus placebo

2.2 SECONDARY OBJECTIVES

- To compare the overall survival (OS) of men with high risk NM-CRPC treated with apalutamide versus placebo
- To compare the time to symptomatic progression in men with high risk NM-CRPC treated with apalutamide versus placebo
- To compare the time to initiation of cytotoxic chemotherapy in men with high risk NM-CRPC treated with apalutamide versus placebo
- To compare the progression-free survival (PFS) of men with high risk NM-CRPC treated with apalutamide versus placebo
- To compare the time to metastasis (TTM) in men with high risk NM-CRPC treated with apalutamide versus placebo
- To evaluate the safety and tolerability of apalutamide

2.3 OTHER OBJECTIVES

- To compare patient reported outcomes (PROs) of health-related quality of life and prostate cancer-specific symptoms in men with high risk NM-CRPC treated with apalutamide versus placebo
- To evaluate the population pharmacokinetics (PK) of apalutamide
- To evaluate the effect of apalutamide on ventricular repolarization in a subset of patients from selected clinical sites
- To evaluate exploratory biomarkers predictive of response and resistance to apalutamide treatment

3. STUDY DESIGN

3.1 STUDY OVERVIEW AND RATIONALE

This is a randomized (2:1), multicenter, double-blind, placebo-controlled, Phase III clinical trial evaluating the efficacy and safety of apalutamide versus placebo in men with high risk (M0) NM-CRPC, defined as PSA Doubling Time (PSADT) ≤ 10 months.

Short PSADT has been consistently associated with reduced time to first (bone) metastasis and death, thus the selected patient population represents one at high risk for development of (distant) metastasis and prostate cancer-specific death.[7,25,26]

No approved treatments are available for the proposed patient population besides continuous administration of ADT (with or without a first generation anti-androgen) as part of the current *de facto* community standard practice, therefore randomization to either apalutamide or placebo is justified in this setting.[14,16] Based on the Phase II preliminary results from Study ARN-509-001, apalutamide is associated with a highly favorable safety profile, and the encouraging anti-tumor activity observed to date in the subgroup of patients with high risk NM-CRPC indicates that apalutamide might have the potential to be efficacious in this earlier line of therapy. The placebo arm will allow an objective comparison of safety and efficacy between treatment with apalutamide and placebo.

Additional PSA evaluations may be necessary to determine eligibility. In order to ensure investigators can obtain these additional evaluations, there is an optional Prescreening Phase with a separate informed consent (see Section 8.1).

The study will consist of a Screening Phase; a Treatment Phase, and a Posttreatment Follow-up Phase. A double-blind study design was chosen to preserve study integrity and minimize bias in the assessment of all study endpoints. A 2:1 randomization scheme will increase the probability that eligible patients will be randomized to receive apalutamide, thereby improving study feasibility.

Randomization will be stratified by PSADT, the use of a bone-sparing agent and the presence of loco-regional disease. These stratification factors were chosen on the basis that they may be sufficiently prognostic such that an imbalance may bias the results.

Apalutamide or matched placebo will be administered orally on a continuous daily dosing schedule at a starting dose of 240 mg per day, the recommended Phase II dose selected based on preclinical projections of the optimal biological dose combined with safety and the PK/pharmacodynamic profiles observed during Phase I. Apalutamide or matched placebo will be given with continuous GnRHa (if patient has not been surgically castrated).

Patients will be followed for safety and efficacy as per the schedule of activities and will remain on study treatment until documented radiographic progression (development of distant metastases as assessed by blinded independent central review [BICR]) or the development of unacceptable toxicity. Treatment decisions will not be based on PSA as the clinical significance of PSA changes during treatment in the setting of NM-CRPC is unknown; therefore, PSA will be collected and analyzed by a central laboratory but

Investigators, patients, and the Sponsor will be blinded to the results until the time of the primary analysis.

Patients discontinuing treatment due to documented radiographic progression will enter the Long-term Follow-up Phase (See Section 8.9 and Appendix 1). Patients discontinuing treatment prior to documented radiographic progression will also enter the Long-term Follow-up Phase where they will continue to have scheduled disease assessments every 16 weeks until documented radiographic progression.

3.1.1 Crossover Option in the Event of Unblinding

At the time of study unblinding and in the event of a positive study result, all subjects currently receiving placebo will have the opportunity to receive active therapy with apalutamide (refer to Appendix 10).

3.1.2 Selection of the Primary Endpoint

Men with high risk NM-CRPC, characterized by rapidly rising PSA in the absence of detectable metastases despite castrate levels of testosterone, are at significant risk for development of metastases and prostate cancer-specific death. As verified in a recently completed Phase III trial of denosumab, men with NM-CRPC who are at high risk for development of bone metastasis have a median overall survival of 44.8 months and a median bone-metastasis-free survival of 25.2 months (placebo arm).[27] To date there are no approved treatments or standard of care for men with high risk NM-CRPC and thus this patient population represents an area of unmet medical need.

In consideration of the relatively long median OS [redacted] in this population and the opportunity to assess for the development of distant metastasis as a clinically important milestone, metastasis-free survival (MFS) was chosen as the primary objective of this study. Metastasis from prostate cancer, especially bone metastases, is the source of prostate cancer specific morbidity and mortality.[15,22] In addition, it triggers the need for a change in therapy that can be associated with an increase in morbidity (e.g., toxicity associated with chemotherapy), and in some cases leading to a change in quality of life for that patient.

Delaying the emergence of radiographically detectable distant metastasis, or prolongation of MFS as proposed, is a clinically relevant outcome that can be robustly and reliably assessed for determination of the true impact of a treatment on the NM-CRPC disease. In a high risk cohort of patients with biochemical recurrence after radical prostatectomy and a PSADT < 15 months, metastatic prostate cancer was reported to account for an estimated 90% of all deaths[7,8] it is therefore conceivable that delaying the development of metastases would likely translate to delaying prostate cancer-specific death. The proposed MFS definition incorporates not only time to (bone or soft tissue) distant metastasis but also time to death and thus MFS can be a reasonable correlate to overall survival. Of note, based on retrospective landmark analyses at 3 and 5 years from the Radiation Therapy and Oncology Group 92-02 randomized trial in patients with locally advanced prostate cancer who had been treated with ADT and external beam radiation therapy, distant metastasis has been shown to be consistent with all four of Prentice’s criteria for being a potential surrogate endpoint for prostate cancer-specific survival at 10 years.[21]

3.2 STUDY OUTCOMES

3.2.1 Primary Endpoint

- Metastasis-Free Survival (MFS)

3.2.2 Secondary Endpoints

- OS
- Time to symptomatic progression
- Time to initiation of cytotoxic chemotherapy
- PFS
- TTM

3.2.3 Other Evaluations

- Health-related quality of life and prostate cancer-specific symptoms
- Type, incidence, severity, timing, seriousness, and relatedness of adverse events and laboratory abnormalities
- PSA response
- Time to PSA progression
- Population PK
- Exploratory biomarkers
- Assessment of ventricular repolarization
- Second progression-free survival (PFS2)
- Medical Resource Utilization (MRU)

4. PATIENT SELECTION

This study can only fulfill its objectives if appropriate patients are enrolled. The following eligibility criteria are designed to select patients for whom protocol treatment is considered appropriate. All relevant medical and non-medical conditions should be taken into consideration when deciding whether this protocol is suitable for a particular patient. It is expected that all patients will be treated according to local standard of care, including radiation therapy if needed for local disease, prior to enrolling.

No waivers will be granted for eligibility criteria deviations.

Patients considered screen failures may be subsequently rescreened. Rescreening must be discussed with and approved by the Sponsor on a case-by-case basis. Patients who are determined to be eligible for the study after rescreening must sign a new informed consent form (ICF) and be assigned a new patient number.

Treatment criteria for placebo subjects who crossover to active treatment with apalutamide (in the event of a positive study result and unblinding) are described in Appendix 10.

4.1 INCLUSION CRITERIA

1. Criterion modified per Amendment INT-3

    1.1 Criterion modified per Amendment INT-5

    1.2 Histologically or cytologically confirmed adenocarcinoma of the prostate without neuroendocrine differentiation or small cell features, with high risk for development of metastases, defined as PSADT ≤ 10 months. PSADT is calculated using at least 3 PSA values obtained during continuous ADT (see Section 5.1).

2. Criterion modified per Amendment INT-5

    2.1 Castration-resistant prostate cancer demonstrated during continuous ADT, defined as 3 PSA rises at least 1 week apart, with the last PSA >2 ng/mL

3. Criterion modified per Amendment INT-3

    3.1 Criterion modified per Amendment INT-6

    3.2 Surgically or medically castrated, with testosterone levels of <50 ng/dL. If the patient is medically castrated, continuous dosing with GnRHa must have been initiated at least 4 weeks prior to randomization and must be continued throughout the study to maintain castrate levels of testosterone.

4. Criterion modified per Amendment INT-3

    4.1 Patients receiving bone loss prevention treatment with bone-sparing agents indicated for the treatment of osteoporosis at doses and dosing schedule appropriate for the treatment of osteoporosis (e.g., denosumab [Prolia®], zoledronic acid [Reclast®]) must be on stable doses for at least 4 weeks prior to randomization.

5. Criterion modified per Amendment INT-3

    5.1 Criterion modified per Amendment INT-5

    5.2 Patients who received a first generation anti-androgen (e.g., bicalutamide, flutamide, nilutamide) must have at least a 4-week washout prior to randomization AND must show continuing disease (PSA) progression (an increase in PSA) after washout.

6. Criterion modified per Amendment INT-3

    6.1 Criterion modified per Amendment INT-5

    6.2 At least 4 weeks must have elapsed from the use of 5-α reductase inhibitors (e.g., dutasteride, finasteride), estrogens (irrespective of dose used), and any other anti-cancer

therapy prior to randomization, including chemotherapy given in the adjuvant/neoadjuvant setting (e.g., clinical trial)

7. At least 4 weeks must have elapsed from major surgery or radiation therapy prior to randomization

8. Age ≥ 18 years

9. Eastern Cooperative Oncology Group (ECOG) Performance Status grade 0 or 1

10. Resolution of all acute toxic effects of prior therapy or surgical procedure to Grade 1 or baseline prior to randomization

11. Criterion modified per Amendment INT-3

 11.1 Criterion modified per Amendment INT-6

 11.2 Adequate organ function as defined by the following criteria:

- Serum aspartate transaminase (AST; serum glutamic oxaloacetic transaminase [SGOT]) and serum alanine transaminase (ALT; serum glutamic pyruvic transaminase [SGPT]) ≤ 2.5 x upper limit of normal (ULN)
- Total serum bilirubin ≤1.5 x ULN. Total serum bilirubin >1.5 x ULN is allowed if Gilbert's disease is documented prior to end of screening procedures.
- Serum creatinine ≤ 2 x ULN
- Absolute neutrophil count (ANC) ≥ 1500/μL
- Platelets ≥ 100,000/μL
- Hemoglobin ≥ 9.0 g/dL
  - Administration of growth factors or blood transfusions will not be allowed within 4 weeks of the hematology labs required to confirm eligibility

12. Signed and dated informed consent document indicating that the patient (or legally acceptable representative) has been informed of all pertinent aspects of the trial prior to randomization

13. Criterion modified per Amendment INT-6

 13.1 Criterion modified per Amendment 7

 13.2 Willingness and ability to comply with scheduled visits, treatment plans, laboratory and radiographic assessments, and other study procedures, including ability to swallow study drug tablets, the completion of patient reported outcomes questionnaires and long-term follow-up

4.2 EXCLUSION CRITERIA

1. Criterion modified per Amendment INT-3

   1.1 Presence of distant metastases confirmed by blinded independent central review (BICR), including CNS and vertebral or meningeal involvement, or history of distant metastases. Exception: Pelvic lymph nodes <2 cm in short axis (N1) located below the iliac bifurcation are allowed

2. Symptomatic loco-regional disease requiring medical intervention, such as moderate or severe urinary obstruction or hydronephrosis, due to primary tumor (e.g., tumor obstruction of bladder trigone)

3. Prior treatment with second generation anti-androgens (e.g., enzalutamide)

4. Criterion modified per Amendment INT-5

   4.1 Prior treatment with CYP17 inhibitors (e.g., abiraterone acetate, orteronel, galerterone, ketoconazole, aminoglutethimide)

5. Prior treatment with radiopharmaceutical agents (e.g., Strontium-89), immunotherapy (e.g., sipuleucel-T), or any other investigational agent for NM-CRPC

6. Prior chemotherapy for prostate cancer, except if administered in the adjuvant/neoadjuvant setting

7. History of seizure or condition that may pre-dispose to seizure (e.g., prior stroke within 1 year prior to randomization, brain arteriovenous malformation, Schwannoma, meningioma, or other benign CNS or meningeal disease which may require treatment with surgery or radiation therapy)

8. Criterion modified per Amendment INT-3

   8.1 Criterion modified per Amendment INT-6

   8.2 Concurrent therapy with any of the following (all must have been discontinued or substituted for at least 4 weeks prior to randomization):

   - Medications known to lower the seizure threshold (for a complete list please see Appendix 5)
   - Herbal (eg, saw palmetto) and non-herbal (eg, pomegranate) products that may decrease PSA levels
   - Systemic (oral/IV/IM) corticosteroids. Short term use (≤ 4 weeks) of corticosteroids during the study is allowed if clinically indicated, but it should be tapered off as soon as possible
   - Any other experimental treatment on another clinical trial

- Agents indicated for the prevention of skeletal-related events in patients with solid tumors (e.g., denosumab [Xgeva®])

9. Criterion modified per Amendment INT-3

9.1 Criterion modified per Amendment INT-6

9.2 History or evidence of any of the following conditions:

- Any prior malignancy (other than adequately treated basal cell or squamous cell skin cancer, superficial bladder cancer, or any other cancer in situ currently in complete remission) within 5 years prior to randomization

- Any of the following within 6 months prior to randomization: Severe/unstable angina, myocardial infarction, symptomatic congestive heart failure, arterial or venous thromboembolic events (e.g., pulmonary embolism, cerebrovascular accident including transient ischemic attacks), or clinically significant ventricular arrhythmias

- Uncontrolled hypertension (systolic blood pressure ≥160 mmHg or diastolic BP ≥100 mmHg). Patients with a history of uncontrolled hypertension are allowed provided blood pressure is controlled by anti-hypertensive treatment.

- Gastrointestinal disorder affecting absorption

- Active infection, such as human immunodeficiency virus (HIV)

- Any other condition that, in the opinion of the Investigator, would impair the patient's ability to comply with study procedures.

5. STUDY TREATMENTS

5.1 RANDOMIZATION CRITERIA

After patients have provided their written informed consent, completed all Screening assessments and received confirmation of eligibility, they will be randomized into the study using an Interactive Voice Randomization System (IVRS) and stratified based on:

- PSADT: > 6 months vs. ≤6 months
- Bone-sparing agent use: Yes vs. No
- Loco-regional disease: N0 vs. N1

In order to ensure accurate and consistent determination of PSADT across all sites, the IVRS will also provide PSADT calculations (using a linear regression model of the normal logarithm of PSA and time) based on at least 3 PSA values obtained during continuous ADT. All available consecutive PSA values obtained within 24 months prior to randomization beginning with the earliest value chosen for the PSADT calculation must be entered in the IVRS. The first and last PSA values used in the calculation must be separated by at least 8 weeks.

Those same PSA values should be used during Screening to determine whether the patient is eligible for the study (inclusion criterion #1.2). In order to pre-screen patients for possible enrollment into the study, PSADT can be calculated using the Memorial Sloan-Kettering Cancer Center (MSKCC) PSA Doubling Time prediction tool, available at the following website:

http://nomograms.mskcc.org/Prostate/PsaDoublingTime.aspx

5.2 BLINDING

Due to the objectives of the study, the identity of test and control treatments will not be known to investigators, research staff, or patients.

Only selected individuals not affiliated with the protocol will be unblinded to individual patient treatment assignment during the trial for the purposes of efficacy analyses and safety review. The randomization codes and all data sets will be stored in a secure area accessible only to these individuals, and only released on completion of the study and after the study database has been locked.

In emergency situations for reasons of patient safety (e.g., a serious unexpected/unlisted drug-related event; a medical emergency; a potentially life-threatening drug interaction), the blinding code may need to be broken. In those cases, whenever possible, a request for unblinding should be discussed with the Sponsor (or designee) prior to unblinding. Detailed instructions on the method for breaking the blind will be provided during site training and in the Investigator Site File.

5.3 FORMULATION

5.3.1 Apalutamide/Matched Placebo

The apalutamide tablet supplied for this study contains 60 mg of JNJ-56021927. It will be manufactured and provided under the responsibility of the Sponsor. Refer to the Investigator's Brochure for a list of excipients. Placebo will be provided as a tablet formulation and will be matched in size, color, and shape to active study drug in order to maintain the study blind.

5.3.2 Packaging, Storage, and Labeling

The apalutamide 60-mg tablets will packaged in 120-count, 160 cc high-density polyethylene (HDPE) bottles with child-resistant caps. Bottles will include desiccant.

The study drug will be periodically tested and monitored for its acceptable shelf life for at least the duration of the study. Any study drug that fails to comply with the manufactured specifications will be promptly removed from the clinical study site and replaced with new supplies by the Sponsor (or designee).

Each bottle of study drug will be labeled with the required regulatory agency warning statement, the protocol number, the Sponsor's name, and directions for patient use and storage. The Investigator will ensure that the study drug is stored in appropriate conditions in a secure location with controlled access. For clinical formulation-specific and batch-specific storage instructions, see the packaging label.

5.3.3 Drug Administration

Apalutamide/matched placebo tablets will be administered orally on a continuous daily dosing regimen at a dose of 240 mg per day (4 x 60-mg tablets) with or without food.

5.3.4 Cycle Management

For the purposes of the study, a treatment cycle will consist of 4 weeks (28 days).

It is anticipated that individual patients may occasionally forget to take a dose. In those cases, missed doses should only be replaced if the patient remembers within a 12-hour window. After that, patients should just take the next dose the following day, without compensating for the missed dose (including vomited doses). In the event of dose delays due to transient toxicity, tumor assessments should remain on schedule independent of cycle length.

5.3.5 Dose Modifications

Intrapatient dose interruptions and/or reductions will be permitted provided that study discontinuation criteria have not been met (please see Section 10).

- Patients experiencing treatment-related seizure of any grade will have study drug permanently discontinued.

- For patients experiencing Grades 1-2 treatment-related adverse events, short treatment breaks can be instituted as per the discretion of the Investigator until the severity of the toxicity decreases to Grade 1 or returns to baseline. If toxicity recurs, dose reductions to the next lower dose level will be allowed as per the discretion of the Investigator.
- For patients experiencing Grade ≥ 3 treatment-related adverse events other than seizure, study drug should be held until the severity of the toxicity decreases to Grade 1 or returns to baseline. If toxicity recurs at Grade 3 or higher, the dose of apalutamide should be reduced to the next lower dose level. A maximum of 2 dose level reductions will be allowed (Table 1).
- Any patient requiring > 28 days delay in treatment due to AEs may meet one of the criteria for study treatment discontinuation (see Section 10), which must be discussed with Sponsor.

Table 1 Apalutamide Dose Levels

Dose Level	Total Daily Dose	Number of 60-mg Tablets (QD)
0	240 mg	4
-1	180 mg	3
-2	120 mg	2

Doses reduced for study treatment-related toxicities should generally not be re-escalated, however, re-escalation back to the previous dose level may be permitted in consultation with the Sponsor (or designee).

5.4 STUDY DRUG ACCOUNTABILITY

The Sponsor (or designee) will ship study drug to the investigational sites. The initial study drug shipment will be shipped after all required regulatory documentation has been received by the Sponsor and a Clinical Trial Agreement fully executed. Subsequent study drug shipments will be made according to an automated resupply algorithm managed by the IVRS.

The study drug will only be dispensed to patients who meet the eligibility criteria and are randomized to a treatment arm in the trial. An accurate and current accounting of the dispensing and return of study drug for each patient will be maintained on an ongoing basis by the Investigator or his/her designated personnel. The number of study drug dispensed and returned by the patient will be recorded on the Investigational Product Accountability Log. The study monitor will verify these documents throughout the course of the study.

At the end of the study, the Sponsor will provide instructions as to the disposition of any unused study drug. If destruction at the site is authorized, the Investigator must ensure that all investigational product is destroyed in compliance with the applicable environmental regulations, institutional policy, and any other special instructions provided by the Sponsor. Drug destruction must be adequately documented.

5.5 MEASURES OF TREATMENT COMPLIANCE

At each clinic visit, patients will be asked to return any remaining study drug from the previous dosing cycle as well as all used and unused study drug containers.

The overall treatment compliance will be defined as the total dose in mg taken during the study divided by the expected total dose in mg. Patients completing their last cycle on capsules should continue with a maximum of 8 capsules per day and patients on tablets should have a maximum of 4 tablets per day.

Capsules or tablets that are not returned will be considered to have been taken, unless otherwise specified in the case report form (CRF).

6. CONCURRENT MEDICATIONS

All patients should be maintained on the same medications throughout the entire study period, as medically feasible, with minimum introduction of new chronic therapies. Every medication or treatment taken by the patient during the trial and the reason for its administration must be recorded on the CRF. Standard medical treatment as applicable is allowed except for treatments noted in the eligibility criteria and/or listed in the prohibited medications section below.

Continuous treatment with a GnRHa or surgical castration is mandatory for all patients in order to maintain castrate concentrations of testosterone (<50 ng/dL). The choice of GnRHa is at the discretion of the investigator. Dose and dose schedule (without interruption) will be consistent with the prescribing information and should only be adjusted if clinically indicated to maintain castrate concentrations of testosterone.

Salvage radiation for loco-regional pelvic disease and surgical procedures (eg, transurethral resection of the prostate [TURP], urethral and ureteral stent placement) to treat localized progression or symptoms are allowed. Patients receiving these therapies may continue on study drug .

6.1 PROHIBITED MEDICATIONS AND TREATMENTS

As a class effect, androgen receptor antagonists have been associated with seizures due to an off-target mechanism of action ($GABA_A$ inhibition).[6,16] In preclinical studies, at very high doses, dogs treated with apalutamide had tremors and generalized seizures. Patients will be closely monitored for seizures, but as a precautionary measure, drugs known to decrease the seizure threshold and/or cause seizure will be prohibited while on study. A list of these medications can be found in Appendix 5.

6.2 RESTRICTED THERAPIES

Investigators should refer to the apalutamide Investigator's Brochure (Sections 4.3.3.3 and 5.10) and associated addenda for complete details on the drug interaction potential of apalutamide. Highlights of drug interaction are summarized below.

- Strong CYP3A4 inducers: the potential for drug-drug interactions with apalutamide has not been tested clinically. Strong inducers of CYP3A4 (eg, phenytoin, carbamazepine, rifampin, rifabutin, rifapentine, phenobarbital, efavirenz, tipranavir, St. John's wort) should be avoided as much as possible. Additional information is provided in Appendix 5.
- Apalutamide may also induce CYP3A4; therefore, caution should be taken when administered in conjunction with CYP3A4 substrates that have a narrow therapeutic index
- Strong CYP2C8 inhibitors (eg, gemfibrozil) should be used with caution with apalutamide

- The potential for drug-drug interaction between apalutamide and warfarin (e.g., Coumadin) is unknown at present. If a patient is taking warfarin, re-assess (prothrombin) PT/(international normalized ratio) INR as clinically indicated and adjust the dose of warfarin accordingly.
- Corticosteroids (Oral, IV, or IM): due to possible resistance mechanisms, which may be contributed by glucocorticoid receptor signaling, concurrent use of corticosteroids during the study is not recommended; short term use (≤ 4 weeks) will be allowed if clinically indicated, however, its use must be tapered off as soon as possible.

6.3 LIFE STYLE GUIDELINES

To avoid risk of drug exposure through the ejaculate (even men with vasectomies), patients must use a condom during sexual activity while on study drug and for 3 months following the last dose of study drug. Donation of sperm is not allowed during the study and for 3 months following the last dose of study drug.

There are no data on the use of apalutamide in pregnancy. Maternal use of an anti-androgen is expected to produce changes in hormone levels that may affect fetal development. It is not known if apalutamide or its metabolites are present in semen.

If the patient is engaged in sexual activity with a woman of childbearing potential, a condom is required along with another effective contraceptive method consistent with local regulations regarding the use of birth control methods for patients participating in clinical studies and their partners. Highly effective forms of contraception include:

- established use of oral, injected or implanted hormonal methods of contraception; placement of an intrauterine device (IUD) or intrauterine (IUS) system;
- barrier methods: condom with spermicidal foam/gel/film/cream/suppository or occlusive cap (diaphragm or cervical/vault caps) with spermicidal foam/gel/film/cream/suppository;
- vasectomy;

- true abstinence (an option when this is in line with the preferred and usual lifestyle of the patient).

Two highly effective forms of contraception are required during the study and for 3 months after the last dose of study drug.

7. STUDY PROCEDURES AND GUIDELINES

A Schedule of Activities representing the required testing procedures to be performed during the study is diagrammed in Appendix 1.

Prior to conducting any study-related activities, written informed consent and any other authorizations must be signed and dated by the patient or patient's legal representative.

Every effort should be made to ensure that the protocol required tests and procedures are completed as described. However, it is anticipated that from time to time there may be circumstances outside of the control of the Investigator that may make it unfeasible to perform the test. In those cases, the Investigator should take all steps necessary to ensure the safety and wellbeing of the patient. When a protocol required test cannot be performed, the Investigator will document the reason for this and any corrective and preventive actions which he/she has taken to ensure that normal processes are adhered to as soon as possible. The study team should be informed of these incidents in a timely fashion.

7.1 CLINICAL ASSESSMENTS

7.1.1 Demographics

Demographic information (e.g., date of birth, gender, race) will be recorded at Screening, as allowed per local country privacy law regulations.

7.1.2 Medical History

Relevant medical history, including history of current disease, other pertinent clinical conditions, and information regarding underlying diseases will be recorded at Screening.

7.1.3 Physical Examination

A complete physical examination will be performed by either the Investigator or a sub-Investigator at Screening. Qualified staff (e.g., nurses or physician assistants) may complete either a full or abbreviated physical exam at all other visits. New abnormal physical exam findings must be documented and followed by a physician or other qualified staff at the next scheduled visit.

The complete physical examination should include, but not limited to, general appearance, skin, neck, eyes, ears, nose, throat, breast, lungs, heart, abdomen, back, lymph nodes, extremities, and nervous system, as well as examination of known and suspected sites of disease. Height will be recorded at Screening only. Body weight will be recorded at Screening and every scheduled visit during treatment and at the end of treatment.

7.1.4 Vital Signs

Body temperature, blood pressure, pulse and respiratory rate will be performed after resting for 5 minutes at Screening and every scheduled visit during treatment and at the end of treatment.

7.1.5 Performance Status

The Eastern Cooperative Oncology Group (ECOG) performance status scale will be used (Appendix 3) and will be assessed at Screening and every subsequent clinic visit.

7.1.6 Adverse Events

Assessment of adverse events will include type, incidence, severity (graded by the National Cancer Institute [NCI] Common Terminology Criteria for Adverse Events [CTCAE], Version 4.0), timing, seriousness, and relatedness (Appendix 4). Adverse events will be assessed at every clinic visit.

7.1.7 Concomitant Medications/Therapies

All concomitant medication and concurrent therapies will be documented from the first dose of study drug until 28 days after the last dose of study drug. Name, indication for administration, and dates of medication or therapy will be captured.

7.1.8 Tumor Assessments

Disease assessments are to be performed as scheduled according to the calendar, regardless of treatment delays resulting from toxicity. Care must be taken in scheduling disease assessments to prevent the introduction of bias based on treatment delays.

Disease assessments will be performed at baseline (Screening), at 16-week intervals from Cycle 1 Day 1, whenever disease progression is suspected, and at the end of treatment. Imaging studies will include a CT scan of the chest, abdomen, and pelvis, plus a bone scan. At Screening, there will be an additional CT of the brain to rule out the presence of CNS metastases.

Radiographic confirmation of disease progression (appearance of distant metastasis) will be based on RECIST 1.1 and assessed by blinded independent central review (see below).[4] For new bone lesions detected on bone scans, a second imaging modality (e.g., CT or MRI) will be required to confirm progression.

The same method of assessment and the same technique should be used at Screening and during follow-up. Intravenous (IV) contrast is required when not medically contraindicated. Patients who have a contraindication to IV contrast may have MRI exams of the brain, abdomen and pelvis performed in lieu of CTs and a non-contrast CT of the chest. Tumor evaluation by positron emission tomography (PET) scan or by ultrasound may not substitute for CT or MRI scans, but the CT portion of a PET/CT may be submitted in lieu of a dedicated CT. Additional requirements are provided in the Imaging Site Manual.

7.1.8.1 Blinded Independent Central Review (BICR)

All scans will be submitted to a third-party core imaging laboratory for independent review of patient eligibility (within 3 days of receipt of imaging scans that pass quality assessment) and disease progression during the study according to an Independent Review Charter to be prepared by the core imaging laboratory in consultation with the Sponsor.

It is important to the integrity of the study that all imaging studies and pertinent clinical information (e.g., bone trauma, fracture, or infection) are forwarded to the core imaging laboratory throughout the study.

Further details regarding materials to be forwarded for central review can be found in the Imaging Manual and/or Investigator Site File.

7.2 CLINICAL LABORATORY MEASUREMENTS

Blood will be obtained at the time points described in the Schedule of Activities and sent to a central lab for hematology and blood chemistry profile. Appendix 2 lists all of the specific tests that will be performed. Complete details with regards to sample collection and shipment processes can be found in the Laboratory Manual and/or Investigator Site File.

Investigators may have additional blood tests performed for the purpose of planning treatment administration, dose modification, or following adverse events.

7.2.1 PSA

Optional prescreening PSA evaluations will be performed by local laboratories.

All other PSA evaluations will be performed at the central laboratory. Results during treatment (until the time of the primary analysis) will be kept blinded to the patients, the Investigators, and the Sponsor, in order to preserve the double-blind nature of this study.

7.2.2 Electrocardiogram (ECG)

A standard 12-lead ECG (with a 10-second rhythm strip) will be collected at Screening and as clinically indicated. ECGs should be collected after the patient has rested quietly and is awake in a fully supine (or semi-recumbent, if supine is not tolerated) position for 10 minutes, and prior to any blood draw collection. Subsequent ECG readings should be collected with the patient in the same position (e.g., fully supine or semi-recumbent). For patients participating in the ventricular repolarization substudy see Appendix 8 for specific instructions.

7.3 PHARMACOKINETIC MEASUREMENTS

Blood for determination of plasma concentrations of apalutamide and its metabolite, ARN000308, will be collected as described in the Laboratory Manual on Day 1 of Cycles 1, 2, 3, 6, 11, 17, and 25. All samples will be assayed using an analytical method validated for apalutamide and metabolite.

On Cycle 1 Day 1, the blood sample must be collected between 0.5 and 4 hours post the first dose of study drug. For all other PK blood collections, the blood sample must be collected prior to study drug administration. No sparse PK samples will be collected from patients participating in the ventricular repolarization study. All reasonable measures must be taken to ensure accurate recording of information on dosing, including the time of the doses of study drug administered on the 2 days preceding the PK sampling day and whether the doses were taken based on a once daily or twice daily schedule (see also Section 8).

7.4 PATIENT-REPORTED OUTCOMES

At each scheduled visit -and during Long-term Follow-up (see Appendix 1), patients will be required to complete two self-administered quality of life instruments: the Functional Assessment of Cancer Therapy-Prostate (FACT-P) and the Euro-QoL Group EQ-5D.[5,30]

The FACT-P will be used to assess health-related quality of life and prostate cancer-specific symptoms. The FACT-P consists of the 27-item FACT-General (FACT-G) and 12 items for the prostate cancer specific concerns. The 27 items in FACT-G are grouped into 4 domains: physical well-being (PWB), social/family well-being (SWB), emotional well-being (EWB) and functional well-being (FWB). The 12 prostate cancer symptoms items focus on pain (3 items), urination problems (3 items), and sexual functions (2 items). In addition, it also contains items for weight loss, appetite, overall comfort, and bowel movement. The FACT-P can be completed in 15 minutes.

The EQ-5D is a validated and reliable self-administered instrument used to assess health status. It contains 6 items designed to assess health status in terms of a single index value or health utility score. The EQ-5D can be completed in less than 5 minutes.

7.5 EXPLORATORY BIOMARKERS

Results from a Phase 1 study in patients with metastatic CPRC provide evidence that acquired genetic anomalies in the AR may be associated with resistance to apalutamide treatment.[10] In this study, 3 of the 29 patients who developed the F876L mutation were considered non-responders. Preclinical data show that changes in expression or development of mutations in genes in the AR-axis may lead to resistance to drug treatment.[12,13] Therefore, blood samples will be collected at various treatment time points from approximately 400 patients to determine the number of patients who develop this mutation and who have resistance to drug treatment. Additional tests will be performed to detect gene expression changes and appearance of other mutations from a panel of preselected RNA and DNA biomarker candidates. Archival formalin-fixed paraffin-embedded (FFPE) tumor blocks or tumor slides will be collected from at least 400 patients in this study to investigate whether genomic classifiers can be used to identify a more homogeneous population of high-risk patients. This will allow selection of the appropriate high-risk patient population for future studies of apalutamide. Other markers associated with the disease and treatment may also be evaluated based on emerging evidence from ongoing studies or published data. Planned biomarker analyses will be deferred if emerging study data show no likelihood of providing useful scientific information.

8. STUDY ASSESSMENTS BY VISIT (APPENDIX 1)

8.1 PRESCREENING

- For this optional Prescreening Phase, review the study with the patient (or patient's legal representative) and obtain written informed consent. The optional prescreening PSA evaluations will be performed by the local laboratories. This Prescreening Phase will allow additional time to obtain the required number of PSA values for determining eligibility. The patient must then sign another informed consent before any additional study-related procedures are conducted (see Section 8.2).

8.2 SCREENING (WITHIN 35 DAYS OF RANDOMIZATION)

- Review the study with the patient (patient's legal representative) and obtain written informed consent
- Calculate PSADT using IVRS to confirm patient eligibility
- Obtain CT of the brain, chest, abdomen, and pelvis, plus bone scan and submit for BICR to confirm patient eligibility (scans obtained prior to signing informed consent will be allowed provided the timing of the scans fall within the Screening window)
- Record demographics data
- Record medical history, including history of prostate cancer, diagnosis date, and prior treatments
- Record concomitant medications
- Perform a complete physical examination (*adverse events must be recorded from the time of signed informed consent*)
- Perform and record vital signs and ECOG performance status grade
- Perform and record standard 12-lead ECG
- Collect blood for clinical laboratory assessments
- Submit patient eligibility form to medical monitor
- If patient is confirmed eligible by the medical monitor, randomize patient and proceed to Cycle 1 Day 1 visit (*can be same day or within 4 days of randomization*)

8.3 CYCLE 1 DAY 1

- Administer FACT-P and EQ-5D questionnaires
- Record changes to concomitant medications
- If available, retrieve archival FFPE tumor blocks or tumor slides from consenting patients for exploratory biomarker analysis
- Record any adverse events
- Perform abbreviated physical examination
- Perform and record vital signs and ECOG performance status

- Collect blood for hematology, blood chemistry, TSH, fasting lipid profile, testosterone, and PSA (Screening evaluations can be used if done within 4 days of Cycle 1 Day 1)
- Collect blood for exploratory biomarker analysis from consenting patients prior to study drug administration
- **Administer study drug in clinic**
- Collect blood for PK sample between 0.5 and 4 hours post-dose and record actual time of collection relative to dosing (not required for patients participating in the ventricular repolarization substudy; see Appendix 8).

8.4 DAY 1 OF CYCLES 1-6, THEN DAY 1 OF EVERY 2 CYCLES STARTING FROM CYCLE 7 UP TO C13 THEN DAY 1 OF EVERY 4 CYCLES (±2 DAYS)

- Administer FACT-P and EQ-5D questionnaires
- Record any adverse events
- Record changes to concomitant medications
- Assess study drug compliance
- Perform abbreviated physical examination
- Perform and record vital signs and ECOG performance status
- Collect blood for hematology, blood chemistry, and PSA
- For Cycles 2, 3, 6, 11, 17, and 25 collect blood for PK sample **prior to study drug administration***(record time of administration of study drug on the 2 days preceding the day of the PK sampling and the dosing regimen [once daily or twice daily]). Not required for subjects participating in the ventricular repolarization study (see Appendix 8).*
- Collect blood for exploratory biomarker analysis from consenting patients (Cycles 11, 17, 25, and 37) prior to study drug administration

8.5 EVERY 16 WEEKS STARTING FROM CYCLE 1 DAY 1 (±2 DAYS)

- Collect blood for TSH, fasting lipid panel, and testosterone
- If TSH is abnormal; total T3, free T4 (direct), and total T4 are required

8.6 EVERY 16 WEEKS STARTING FROM CYCLE 1 DAY 1 (±7 DAYS)

- Obtain CT of the chest, abdomen, and pelvis, plus bone scan and submit for BICR to evaluate for disease progression (ie, distant metastasis)

8.7 END-OF-TREATMENT VISIT

- Administer FACT-P and EQ-5D questionnaires (*optional if performed within 2 weeks of the last dose of study drug*)
- Assess study drug compliance

- Obtain CT of the chest, abdomen, and pelvis, plus bone scan and submit for BICR to evaluate for disease progression (*optional if performed with 8 weeks of the last dose of study drug*)
- Record any adverse events
- Record changes to concomitant medications

The following are optional if the End-of-Treatment Visit is performed within 1 week of the last dose of study drug:

- Perform abbreviated physical examination
- Perform and record vital signs and ECOG performance status
- Collect blood for clinical laboratory assessments
- Collect blood for exploratory biomarker analysis from consenting patients

8.8 SAFETY FOLLOW-UP (28 DAYS FOLLOWING THE LAST DOSE OF STUDY DRUG)

- Record any adverse events
- Record changes to concomitant medications

8.9 LONG-TERM FOLLOW-UP

- Obtain survival status, recording of development of symptomatic progression, initiation of any new systemic anti-cancer therapies, progression on first subsequent therapy (PFS2), and MRU every 4 months via clinic visit, telephone contact, or an alternative contact method per institution policy/practice. The FACT-P and EQ-5D questionnaires will be collected up to 12 months post-progression.
- **In addition, if patients discontinue study treatment prior to documented disease progression (ie, distant metastasis, see Section 7.1.8)**, obtain CT of the chest, abdomen, and pelvis, plus bone scan and submit for BICR to evaluate for disease progression (every 16 weeks, until documentation of disease progression)

8.10 SUBSEQUENT THERAPY WITH ABIRATERONE ACETATE

Due to local restrictions in Japan, the Sponsor will not provide abiraterone acetate and the criteria outlined in this section do not apply.

The Sponsor will provide abiraterone acetate to all other patients who meet the following criteria:

- patient signs informed consent to receive treatment with abiraterone acetate
- documented disease progression (ie, meet criteria for the primary endpoint [distant metastasis], see Section 13.2.1). If the study is unblinded, the requirement for meeting disease progression by BICR in Section 13.2.2 will not be required.

- the patient resides in a country in which abiraterone acetate and prednisone (or prednisolone) are indicated for the treatment of metastatic CRPC before chemotherapy
- the investigator caring for the patient decides that abiraterone acetate is the appropriate first subsequent treatment after discontinuation of study drug; and
- no other subsequent treatment has been prescribed after the progression event and before abiraterone acetate is prescribed.

Abiraterone acetate should be used according to the product label-prescribing information in the country of residence.

Prednisone (or prednisolone) should be used according to the product label-prescribing information in the country of residence. Prednisone (or prednisolone) will not be provided by the Sponsor, but should be prescribed as part of the regimen.

In countries where abiraterone acetate in combination with prednisone (or prednisolone) is not approved for the treatment of asymptomatic or mildly symptomatic mCRPC before chemotherapy, patients should be treated according to the local standard of care. Provision of abiraterone acetate will continue until the patient or investigator decides not to continue further treatment for any reason, unacceptable toxicity or the study is terminated by the Sponsor.

Serious adverse events will be collected during subsequent therapy with abiraterone acetate.

9. ADVERSE EVENT REPORTING REQUIREMENTS

9.1 DEFINITIONS

9.1.1 Adverse Events (AE)

An AE is any untoward medical occurrence in a clinical study patient administered a medicinal (investigational or non investigational) product. An AE does not necessarily have a causal relationship with the treatment. An adverse event can therefore be any unfavorable and unintended sign (including an abnormal finding), symptom, or disease temporally associated with the use of a medicinal (investigational or non investigational) product, whether or not related to that medicinal (investigational or non investigational) product. (Definition per International Conference on Harmonisation [ICH])

This includes any occurrence that is new in onset or aggravated in severity or frequency from the baseline condition, or abnormal results of diagnostic procedures, including laboratory test abnormalities.

Note: The Sponsor collects AEs starting with the signing of the ICF (refer to Section 9.2, for time of last AE recording).

Examples of AEs include but are not limited to:

- Abnormal test findings
- Clinically significant signs and symptoms
- Changes in physical examination findings
- Worsening of signs and symptoms of the malignancy under study. *Disease progression assessed by measurement of malignant lesions on radiographs or other methods should **not** be reported as an adverse event, unless the outcome is fatal during the study or within the safety reporting period – see definition of serious adverse event below.*
- Signs or symptoms resulting from dose overdose, dependency, withdrawal, abuse, and/or misuse
- Drug interactions
- Exposure in utero (pregnancy)

For laboratory abnormalities, the criteria for determining whether an abnormal test finding should be reported as an adverse event are as follows:

- Test result is associated with accompanying symptoms, and/or
- Test result requires additional diagnostic testing or medical/surgical intervention, and/or
- Test result leads to a change in study dosing outside of protocol-stipulated dose adjustments or discontinuation from the study, significant additional concomitant drug treatment, or other therapy, and/or

- Test result is considered to be an adverse event by the Investigator

9.1.2 Serious Adverse Events (SAE)

A serious adverse event (SAE) based on ICH and EU Guidelines on Pharmacovigilance for Medicinal Products for Human Use is any untoward medical occurrence that at any dose:

- Results in death. *If the malignancy under study has a fatal outcome during the study or within the safety reporting period, the event leading to death should be reported as a Grade 5 SAE; death is an outcome and not the adverse event in itself.*
- Is life-threatening (i.e., immediate risk of death from the reaction as it occurred). *It does* ***not*** *include a reaction which hypothetically might have caused death had it occurred in a more severe form.*
- Requires or prolongs inpatient hospitalization (i.e., the event required at least a 24-hour hospitalization or prolonged a hospitalization beyond the expected length of stay). Hospitalization admissions and/or surgical operations scheduled to occur during the study period, but planned prior to study entry are not considered SAEs if the illness or disease existed before the person was enrolled in the trial, provided that it did not deteriorate in an unexpected manner during the trial (e.g., surgery performed earlier than planned)
- Results in persistent or significant disability/incapacity. Disability is defined as a substantial disruption of a person's ability to conduct normal life functions
- Results in a congenital anomaly or birth defect
- Is a suspected transmission of any infectious agent via a medicinal product
- Is an important medical event when, based upon appropriate medical judgment, it may jeopardize the participant and require medical or surgical intervention to prevent one of the outcomes listed above. Examples of such medical events include allergic bronchospasm requiring intensive treatment in an emergency room or at home; blood dyscrasias or convulsions that do not result in inpatient hospitalization, or the development of drug dependency or drug abuse

Events **not** considered to be SAEs are hospitalizations for:

- Routine treatment or monitoring of the studied indication, not associated with any deterioration in condition
- Elective or pre-planned treatment for a pre-existing condition that did not worsen
- Emergency outpatient treatment for an event not fulfilling the serious criteria outlined above and not resulting in inpatient admission
- Respite care or social admissions

9.1.3 Expectedness

An adverse event or suspected adverse reaction is considered "unexpected" if it is not listed in the Investigator's Brochure or is not listed at the specificity or severity that has been observed.

Unexpected, as used in this definition, also refers to adverse events or suspected adverse reactions that are mentioned in the Investigator's Brochure as occurring with a class of drugs or as anticipated from the pharmacological properties of the drug, but are not specifically mentioned as occurring with the particular drug under investigation. Such events would be considered unexpected until they have been observed with the drug under investigation.

9.1.4 Attribution

A suspected adverse reaction means any adverse event for which there is reasonable possibility that the study drug caused the adverse event. For the purposes of IND safety reporting, "reasonable possibility" means there is evidence to suggest a causal relationship between the drug and the adverse event.

The Investigator will assign attribution of the possible association of the event with the study drug using the following definitions:

- **Unrelated to study drug**: The adverse event is *clearly not related* or is *doubtfully related* to the study drug
- **Related to study drug**: The adverse event *may be related,* is *likely related,* or is *clearly related* to the study drug

9.1.5 Severity

Signs or symptoms should be graded and recorded by the Investigator according to the National Cancer Institute's Common Terminology Criteria for Adverse Events (CTCAE) Version 4.0 (Appendix 4). When specific adverse events are not listed in the CTCAE, they are to be graded as mild, moderate, severe, or life-threatening according to the following grades and definitions:

Table 2 AE Severity Grading

**Severity (Toxicity Grade)**	**Description**
Grade 1	Mild; asymptomatic or mild symptoms; clinical or diagnostic observations only; intervention not indicated
Grade 2	Moderate; minimal, local or noninvasive intervention indicated; limiting age-appropriate instrumental activities of daily living (ADL)
Grade 3	Severe or medically significant but not immediately life-threatening; hospitalization or prolongation of hospitalization indicated; disabling; limiting self-care ADL
Grade 4	Life-threatening consequences; urgent intervention indicated
Grade 5	Death related to AE

9.1.6 Pregnancy

Because the effect of the blinded study drug on sperm is unknown, pregnancies in partners of male subjects included in the study will be reported by the investigational staff within 24 hours of their knowledge of the event using the appropriate pregnancy notification form.

Follow-up information regarding the outcome of the pregnancy and any postnatal sequelae in the infant will be required.

9.2 REPORTING REQUIREMENTS

All AEs and SAEs whether reported by the patient, discovered during questioning, directly observed, or detected by physical examination, laboratory test or other means, will be recorded in the patient's medical record and on the appropriate study-specific CRFs. Anticipated events will be recorded and reported as described in Appendix 9 (see also Section 9.2.3).

9.2.1 SAE Reporting

During the Prescreening Phase only, the SAE reporting would be limited to SAEs related to the PSA blood draw. At screening, the reporting period for SAEs begins from the time the patient provides informed consent and prior to the patient's participation in the study, i.e., prior to undergoing any study-related procedure through and including 28 days after the last dose of study drug. Serious adverse events will also be collected during subsequent therapy with abiraterone acetate (see Section 8.10).

All SAEs occurring during the study must be reported to the appropriate Sponsor contact person by study-site personnel within 24 hours of their knowledge of the event.

Information regarding SAEs will be transmitted to the Sponsor using the Serious Adverse Event Form, which must be completed and signed by a physician from the study site, and transmitted to the Sponsor within 24 hours. The initial and follow-up reports of an SAE should be made by facsimile (fax) or email. The names (and corresponding telephone numbers) of the individuals who should be contacted regarding safety issues or questions regarding the study will be provided as a separate document.

The Investigator is also responsible for notifying the Institutional Review Board/Independent Ethics Committee (IRB/IEC) in accordance with local regulations.

All events should be followed to their resolution, until the Investigator assesses them as stable, irreversible, or until the patient is lost to follow-up, whichever comes first. Any SAEs occurring any time after the reporting period must be promptly reported if a causal relationship to apalutamide is suspected.

Reporting Deaths: Regardless of relationship to study drug, all deaths on study should be reported through and including 28 days after the last dose of study. Deaths occurring after the safety follow-up period do not have to be reported as SAEs unless considered related to study drug.

For all SAEs, the Investigator is obligated to pursue and provide information to the Sponsor in accordance with the timeframes for reporting specified above. In addition, an Investigator may be requested to obtain specific additional follow-up information in an expedited fashion. This information may be more detailed than that captured on the AE CRF. In general, this will include a description of the adverse event in sufficient detail to allow for a complete medical assessment of the case and independent determination of possible causality. Information on other possible causes of the event, such as concomitant medications and illnesses must be provided. In the case of a patient death, a summary of available autopsy findings must be submitted as soon as possible to the Sponsor or its designated representative.

9.2.2 Non-Serious AE Reporting

Adverse events should be recorded on the AE CRF from the time the patient has signed the informed consent at screening (see Section 8.2) until 28 days after the last dose of study drug. All events should be followed to their resolution, until the Investigator assesses them as stable, irreversible, or until the patient is lost to follow-up, whichever comes first. If a patient begins a new systemic anti-cancer therapy, the AE reporting period for non-SAEs ends at the time the new treatment is started.

9.2.3 Sponsor Reporting Requirements to Regulatory Authorities

Adverse events reporting, including suspected serious unexpected adverse reactions, will be carried out in accordance with applicable local regulations. The Sponsor or its designee will be responsible for determining whether or not the suspected adverse reaction meets the criteria for expedited reporting.

- For anticipated events reported as individual serious adverse events the Sponsor will make a determination of relatedness in addition to and independent of the investigator's assessment. The Sponsor will periodically evaluate the accumulating data and, when there is sufficient evidence and the sponsor has determined there is a reasonable possibility that the drug caused a serious anticipated event, they will submit a safety report in narrative format to the investigators (and the head of the institute).
- The Sponsor or its designee will be responsible for reporting relevant SAEs to the Competent Authority, other applicable regulatory authorities, and participating investigators, in accordance with ICH guidelines, FDA regulations, European Clinical Trials Directive (Directive 2001/20/EC), and/or local regulatory requirements.
- The Sponsor or its designee will be responsible for reporting unexpected fatal or life-threatening events associated with the use of the study drug to the regulatory authorities by telephone or fax within 7 calendar days after being notified of the event.
- The Sponsor or its designee will report other relevant SAEs associated with the use of the study drug to the appropriate regulatory authorities (according to local guidelines) and Investigators by a written safety report within 15 calendar days of notification.

9.2.4 Product Quality Complaint Handling

A product quality complaint (PQC) is defined as any suspicion of a product defect related to manufacturing, labeling, or packaging, ie, any dissatisfaction relative to the identity, quality, durability, or reliability of a product, including its labeling or package integrity. A PQC may have an impact on the safety and efficacy of the product. Timely, accurate, and complete reporting and analysis of PQC information from studies are crucial for the protection of subjects, investigators, and the sponsor, and are mandated by regulatory agencies worldwide. The sponsor has established procedures in conformity with regulatory requirements worldwide to ensure appropriate reporting of PQC information; all studies conducted by the sponsor or its affiliates will be conducted in accordance with those procedures.

9.2.4.1 Procedures

All initial PQCs must be reported to the sponsor by the study-site personnel within 24 hours after being made aware of the event.

If the defect is combined with a SAE, the study-site personnel must report the PQC to the sponsor according to the SAE reporting timelines (refer to Section 9.2.1). A sample of the suspected product should be maintained for further investigation if requested by the sponsor.

9.2.4.2 Contacting Sponsor Regarding Product Quality

The names (and corresponding telephone numbers) of the individuals who should be contacted regarding product quality issues are listed on the Contact Information page(s), which will be provided as a separate document.

10. END OF TREATMENT

A patient may be discontinued from study treatment at any time if the patient, the Investigator, or the Sponsor feels that it is not in the patient’s best interest to continue on study. If a patient’s study treatment must be discontinued, this will not result in automatic withdrawal of the patient from the study.

The following is a list of possible reasons for early discontinuation of study treatment:

- Disease progression (*patients should be highly encouraged to stay on study treatment until there is BICR-confirmed radiographic progression; treatment decisions should not be based on PSA alone)*
- Any episode of seizure
- Any other adverse event that cannot be adequately managed with dose modifications, including dose interruption for ≥ 28 days may require study drug discontinuation, which must be discussed with the Sponsor.
- Protocol violation requiring discontinuation of study treatment
- Patient is not compliant with study procedures
- Lost to follow-up
- Patient withdrawal of consent

- Sponsor request for early termination of study

All patients discontinuing study treatment will enter the Long-term Follow-up Phase and will be followed for the development of symptomatic progression and initiation of subsequent anti-cancer therapies every 4 months until death, loss of follow-up, or withdrawal of consent, whichever comes first.

If a patient is lost to follow-up, every reasonable effort must be made by the study site personnel to contact the patient and determine the reason for discontinuation/withdrawal. The measures taken to follow up must be documented.

When a subject withdraws before completing the study, the reason for withdrawal is to be documented in the CRF and in the source document. Study drug assigned to the withdrawn subject may not be assigned to another subject. Subjects who withdraw will not be replaced.

Data to be collected for the end of treatment visit are described in Section 8.7. Patients will be followed for at least 28 days after the last dose of study drug. If a patient is withdrawn from treatment due to an adverse event, the patient will be followed until the adverse event has resolved or stabilized as per Section 9.2.

For information on subsequent therapy with abiraterone acetate see Section 8.10

11. PROTOCOL VIOLATIONS

A protocol violation occurs when the patient or Investigator fails to adhere to significant protocol requirements affecting the inclusion, exclusion, patient safety and primary endpoint criteria. Protocol violations for this study include, but are not limited to, the following:

- Failure to meet inclusion/exclusion criteria [*no waivers will be granted to meet the eligibility criteria*]
- Use of a prohibited concomitant medication
- Dose modifications that are not within the protocol specifications
- Any other deviation that presents significant risk or safety concerns to the patient

Failure to comply with Good Clinical Practice (GCP) guidelines will also result in a protocol violation. The Sponsor, in consultation with the Investigator, will determine if a protocol violation should result in withdrawal of a patient.

12. DATA MONITORING COMMITTEE

An Independent Data Monitoring Committee (IDMC) will be established to monitor data on an ongoing basis to ensure the safety of the subjects enrolled in this study. The committee will meet periodically to review interim safety data. After the review, the IDMC will make recommendations regarding the conduct of the study. The IDMC will serve as the primary reviewers of the efficacy analysis. Further details will be provided in a separate IDMC charter.

The IDMC will consist of at least 2 medical experts in the relevant therapeutic area and at least 1 statistician. The IDMC responsibilities, authorities, and procedures will be documented in the IDMC charter.

13. STATISTICAL METHODS AND CONSIDERATIONS

A detailed methodology for summary and statistical analyses of the data collected in this study will be documented in a Statistical Analysis Plan (SAP) that will be dated, version-controlled and maintained by the Sponsor.

13.1 ANALYSIS POPULATIONS

Full Analysis (Intent-to-Treat) Population [ITT]: All eligible patients who are randomized into the study, with study drug assignments designated according to initial randomization, regardless of whether patients receive study drug or receive a different drug from that to which they were randomized to will be included in the analyses of all efficacy and clinical benefit endpoints and patient characteristics.

Safety Analysis Population [SAFETY]: All patients who receive at least one dose of study drug, with treatment assignments designated according to actual study treatment received will be the primary population for evaluating safety and treatment compliance and administration.

- **Patient Report Outcomes Population [PRO]**: Subset of the safety analysis population that has completed at least the baseline assessment (Cycle 1 Day 1) of either FACT-P or EQ-5D questionnaires.
- **Population Pharmacokinetics Populations [PK]**: Subset of the safety analysis population that was randomized to the apalutamide treatment arm and that has at least one PK sample collected.
- **Biomarker Population**: Subset of the safety analysis populations that has at least 1 biomarker sample collected.

13.2 EFFICACY ANALYSES

Efficacy analyses will be performed on the ITT population, incorporating the randomization stratification factors as documented on the CRF, unless otherwise specified.

Analyses of efficacy endpoints which are based on radiographic tumor assessments (MFS, PFS, and TTM) will be based on the results of the BICR. Investigator assessments may be used for sensitivity analyses, as described in the Statistical Analysis Plan.

13.2.1 Analysis of Primary Endpoint

The primary endpoint for the study is metastasis-free survival (MFS) which is defined as the time from randomization to first evidence of BICR-confirmed radiographically detectable bone or soft tissue distant metastasis (simply referred to as “metastasis” from this point forward) or death due to any cause (whichever occurs earlier) + 1 day.

The MFS data for patients without metastasis or death will be censored on the date of the last tumor assessment (or, if no tumor assessment was performed after the baseline visit, at the date of randomization + 1 day). Additional censoring rules will vary according to whether the analysis is performed for US or ex-US regulatory purposes, as shown below; both results will be provided in the clinical study report.

**Scenario**	**US regulatory guidance**	**ex-US regulatory guidance**[1]
Data from patients who are lost to follow-up or whose disease progression (development of metastasis) or death occurs after 2 or more consecutively missing or unevaluable tumor assessments	Censored on the date of the last tumor assessment that the patient was known to be metastasis-free	Time of progression will be determined using the first date when there is documented evidence of progression or death (whichever occurs earlier) regardless of missed or unevaluable tumor assessments
Patients that receive new systemic anti-cancer therapy prior to documented disease progression (development of metastasis) or death	Censored on the date of the last tumor assessment prior to the start of the new systemic anti-cancer therapy	Time of progression will be determined using the first date when there is documented evidence of progression or death (whichever occurs earlier) regardless of change of therapy

The primary efficacy analysis will be completed when approximately [redacted] MFS events have occurred. The primary analysis will compare the MFS distributions in the two treatment arms using a two-sided log-rank test, stratified by PSADT (> 6 months vs. ≤ 6 months), the use of a bone-sparing agent (Yes vs. No), and the presence of loco-regional disease (N0 vs. N1) at the 0.05 significance level. The unstratified log-rank test will be provided as a sensitivity analysis. A complete list of sensitivity analyses is provided in the Statistical Analysis Plan.

Kaplan-Meier methods will be used to estimate median MFS for each treatment arm. Cox proportional-hazard models, including the same factors as above, will be used to estimate the hazard ratio and its 95% confidence interval (CI). Additional analyses by formulation

subgroups will be performed for the MFS endpoint as described in the Statistical Analysis Plan.

13.2.2 Analyses of Secondary Endpoints

For the secondary endpoints, a hierarchical testing will be performed in the following order:

- [redacted]
- [redacted]
- [redacted]
- [redacted]
- [redacted]

each at alpha=0.05 (2-sided). Each secondary endpoint will have a [redacted] analysis but there will be [redacted] for [redacted] and [redacted] There will be [redacted] The testing of [redacted] endpoints will utilize [redacted] method, according to the [redacted] alpha spending function with [redacted] number of events necessary for the [redacted] analysis to maintain the [redacted] power. [redacted] otherwise there will be 2 interim analyses for [redacted] and [redacted]

The final analysis of [redacted] OS and [redacted] will [redacted] be performed at the [redacted] the primary analysis of MFS (approximately [redacted] events).

Full details about the [redacted] procedure will be provided in the statistical analysis plan.

Time-to-event-based secondary endpoint analyses ([redacted] will be performed using a two-sided log-rank test, stratified by PSADT (> 6 months vs. ≤ 6 months), the use of a bone-sparing agent (Yes vs. No), and the presence of loco-regional disease (N0 vs. N1). Unstratified log-rank tests will also be provided as sensitivity analyses.

Kaplan-Meier methods will be used to estimate medians for each treatment arm. Cox proportional-hazard models, including the same factors as above, will be used to estimate the hazard ratio and its 95% confidence interval (CI). One year, 2-year, 3-year, and 5-year survival rates will be estimated using the Kaplan-Meier method.

13.2.2.1 OS

Overall survival will be defined as the time from randomization to the date of death due to any cause + 1 day. Patients who are alive at the time of the analysis will be censored on the last known date that they were alive. In addition, the following censoring rules will apply:

Scenario	Date of Censoring
Patients with no post-baseline information	Censored on the date of randomization + 1 day
Patients who are lost to follow-up or who withdraw consent for further follow-up	Censored on the last known date that they were alive
Sensitivity Analysis: Patients that receive new systemic anti-cancer therapy	Censored on the day before the start date of the new systemic anti-cancer therapy

13.2.2.2 Time to Symptomatic Progression

Time to symptomatic progression will be defined as the time from randomization to documentation in the CRF of any of the following (whichever occurs earlier) + 1 day:

- Development of a skeletal-related event (SRE): pathologic fracture, spinal cord compression, or need for surgical intervention or radiation therapy to the bone.
- Pain progression or worsening of disease-related symptoms requiring initiation of a new systemic anti-cancer therapy.
- Development of clinically significant symptoms due to loco-regional tumor progression requiring surgical intervention or radiation therapy.

Adverse event, concomitant medication, concomitant treatment, or survival follow-up CRFs will be the source of these findings.

Time to symptomatic progression for patients who do not experience any of the events described above will be censored on the date on which they were last known to be event-free.

13.2.2.3 Time to Initiation of Cytotoxic Chemotherapy

Time to initiation of cytotoxic chemotherapy will be defined as the time from randomization to documentation in the CRF of a new cytotoxic chemotherapy being administered to the patient (e.g., survival follow-up CRF) + 1 day.

Time to initiation of cytotoxic chemotherapy for patients who do not start cytotoxic chemotherapy will be censored on the date of last contact.

13.2.2.4 Progression-Free Survival

In order to capture loco-regional disease progression, a secondary endpoint of progression-free survival (PFS) will be assessed and defined as the time from randomization to first documentation of BICR-confirmed radiographic progressive disease or death due to any cause (whichever occurs first) + 1 day.

Progressive disease will be determined based on RECIST v1.1, and further defined as follows:

- For patients with at least one measurable lesion, disease progression will be defined as at least a 20% increase in the sum of diameters of target lesions taking as reference the smallest sum on study (this includes the baseline sum if that is the smallest on study). In addition to the relative increase of 20%, the sum must also demonstrate an absolute increase of at least 5 mm. Furthermore, the appearance of one or more new lesions is also considered progression.
- For patients with only non-measurable disease observed on CT or MRI scans, unequivocal progression (representative of overall disease status change) or the appearance of one or more new lesions will be considered progression. For new bone lesions detected on bone scans, a second imaging modality (e.g., CT or MRI) will be required to confirm progression.

Progression-free survival data for patients without loco-regional disease will be censored on the date of the last tumor assessment (or, if no tumor assessment was performed after the baseline visit, at the date of randomization + 1 day). Additional censoring rules will vary according to whether the analysis is performed for US or ex-US regulatory purposes, as shown below; both results will be provided in the clinical study report.

Scenario	US regulatory guidance	ex-US regulatory guidance
Data from patients who are lost to follow-up or whose disease progression or death occurs after 2 or more consecutively missing or unevaluable tumor assessments	Censored on the date of the last tumor assessment that the patient was known to be progression-free	Time of progression will be determined using the first date when there is documented evidence of progression or death (whichever occurs earlier) regardless of missed or unevaluable tumor assessments
Patients that receive new systemic anti-cancer therapy prior to documented disease progression or death	Censored on the date of the last tumor assessment prior to the start of the new systemic anti-cancer therapy	Time of progression will be determined using the first date when there is documented evidence of progression or death (whichever occurs earlier)

		regardless of change of therapy

13.2.2.5 Time to Metastasis

Time to metastasis will be defined as the time from randomization to first evidence of BICR-confirmed radiographically detectable bone or soft tissue distant metastasis (simply referred to as "metastasis" from this point forward) + 1 day.

Time to metastasis data for patients without metastasis will be censored on the date of the last tumor assessment (or, if no tumor assessment was performed after the baseline visit, at the date of randomization + 1 day). Additional censoring rules will vary according to whether the analysis is performed for US or ex-US regulatory purposes, as shown below; both results will be provided in the clinical study report.

Scenario	US regulatory guidance	ex-US regulatory guidance
Data from patients who are lost to follow-up or whose disease progression (development of metastasis) occurs after 2 or more consecutively missing or unevaluable tumor assessments	Censored on the date of the last tumor assessment that the patient was known to be metastasis-free	Time of progression will be determined using the first date when there is documented evidence of progression regardless of missed or unevaluable tumor assessments
Patients that receive new systemic anti-cancer therapy prior to documented disease progression (development of metastasis)	Censored on the date of the last tumor assessment prior to the start of the new systemic anti-cancer therapy	Time of progression will be determined using the first date when there is documented evidence of progression regardless of change of therapy

13.3 SAFETY EVALUATIONS

The SAFETY population will be the primary population for evaluating safety.

13.3.1 Analysis of Adverse Events

Adverse events (AEs) will be graded according to the NCI Common Terminology Criteria for Adverse Events (CTCAE) Version 4.0 and coded to preferred term and system organ class (SOC) using the most recent version of MedDRA.

All AEs reported from the first dose of study drug until 28 days after the last dose of study drug will be considered as treatment-emergent AEs and will be summarized by treatment arm.

For each treatment arm, adverse event incidence rates will be summarized with frequency and percentage by MedDRA SOC and preferred term, with all patients treated in that treatment arm as the denominator, unless otherwise specified. In addition, AE incidence rates will also be summarized by severity and relationship to study drug. Treatment-related AEs are those judged by the Investigator to be at least possibly related to the blinded study drug. Adverse events with missing severity or relationship to study drug will be classified as severe and treatment-related, respectively. Patients with multiple occurrences of events will only be counted once at the maximum severity to study drug for each preferred team, SOC, and overall. Deaths that occur within 28 days after the last dose of study drug are defined as on-study deaths.

Summary tables and individual patient listings will be prepared as per the Statistical Analysis Plan. An additional analysis by formulation subgroups will be performed as outlined in the Statistical Analysis Plan.

13.3.2 Analysis of Clinical Laboratory Results

Only data collected by the central laboratory will be summarized. Local laboratory data collected for the purposes of planning treatment administration, dose modification, or monitoring adverse events, will not be summarized.

Normal ranges will be used to identify values that are outside the normal ranges and abnormal laboratory results will be graded according to the NCI CTCAE Version 4.0. Descriptive statistics will be provided for each test result and for the change from baseline by visit.

A shift summary of baseline grade by maximum post-baseline CTCAE grade will be presented, as appropriate. For each laboratory parameter, the baseline laboratory value will be defined as the last laboratory value collected on or prior to the first dose of study drug.

Patients who develop toxicities of Grade ≥ 3 will be summarized. Laboratory test results not having CTCAE grade will also be summarized. Parameters that have criteria available for both low and high values (e.g., hypercalcaemia vs. hypocalcaemia) will be summarized for both criteria. Patients will only be counted once for each criterion.

13.3.3 Analysis of Vital Signs

Each vital sign (temperature, blood pressure (systolic and diastolic), respiration rate, and heart rate) and respective change from baseline will be summarized and presented by treatment arm and study visit. Patients with clinically significant abnormalities in vital signs as compared to baseline will be listed.

13.3.4 Concomitant Medications/Treatments

All medications and/or treatments received during the protocol Treatment Phase will be considered as concomitant medications and/or concomitant treatments and will be coded by WHO medical dictionary; patients who received concomitant medications and/or treatments will be listed.

13.4 OTHER EVALUATIONS

13.4.1 Second Progression-free Survival (PFS2)

This endpoint is defined as the time from randomization to second documentation of investigator-assessed disease progression (PSA, radiographic, symptomatic, or any combination) or death from any cause.

13.4.2 PSA

PSA kinetics (e.g., 12-week PSA response and time to PSA progression) will be assessed according to the Prostate Cancer Clinical Trials Working Group (PCWG2) recommendations.[24]

Summary tables and waterfall plots describing change in PSA relative to baseline will be reported at 12 weeks (or earlier for those who discontinue study treatment prior to 12 weeks), and separately, the maximum change at any time on study will also be reported for each patient using summary tables and waterfall plots.

The time to PSA progression will be calculated as the time from randomization to the time when the criteria for PSA progression according to PCWG2 are met + 1 day. Kaplan-Meier methods will be used to estimate the median time to PSA progression and 95% confidence intervals for each treatment arm.

13.4.3 Health-Related Quality of Life and Prostate Cancer-Specific Symptoms Analysis

The FACT-P and EQ-5D data will be scored and handled as recommended in their respective User's manuals, including handling of missing data both within the subscales and overall. All the analysis for FACT-P and EQ-5D data will be performed in the PRO population.

A 10-point change in the FACT-P total score is considered clinically meaningful. Therefore, any patient experiencing a 10-point decrement in FACT-P total scores from baseline will be considered to have experienced clinically meaningful deterioration in functional status. The proportion of patients with at least a 10-point decrement in FACT-P total score will be summarized by treatment arm. The decrement in the FACT-P total score between treatment arms will be compared using a Mantel-Haenszel test, stratified by PSADT (>6 months vs.≤6 months), the use of a bone-sparing agent (Yes vs. No), and the presence of loco-regional disease (N0 vs. N1) at a two-sided 0.05 significance level.

The EQ-5D data will be summarized descriptively by treatment group and study visit.

13.4.4 Population Pharmacokinetics (Pop PK) Analysis

Population PK analysis will utilize patient covariates to identify sub-populations where possible; the effect of the formulations will also be explored in the covariate analysis. The relationship of exposure to apalutamide and active metabolite (ARN000308) to measures of efficacy and adverse events will also be modeled to the extent possible. The PK population will be the primary population used for population PK analysis, while placebo data will also be included for PK-efficacy or PK-adverse events analyses.

Population analysis methods will be utilized as applicable. Population PK analysis of plasma concentration-time data of apalutamide will be performed using nonlinear mixed-effects modeling. The population PK analysis results will be presented in a separate report.

13.4.5 Exploratory Biomarkers Analysis

Blood and plasma samples will be collected at multiple time points and archived FFPE tumor blocks or tumor slides may be analyzed for development of the F876L mutation (plasma) and for high risk features (FFPE tumor blocks or tumor slides) and associations may be made with clinical endpoints as follows.

- F876L mutation and other DNA mutations from cfDNA in plasma collected at Day 1 of Cycles 1, 11, 17, 25, 37, and End-of-Treatment Visit.
- AR splice variants or other RNA anomalies in cfRNA in blood collected at Day 1 of Cycles 1 and 11, and End-of-Treatment Visit.
- Global mRNA expression levels in FFPE tumor blocks or tumor slides to identify expression levels of 'high-risk' classifier genes.

High-risk genomic classifiers may be evaluated if it can be used to identify a more homogeneous population of high-risk patients using appropriate categorical or regression methods.

The association of the rest of the biomarkers with clinical response or relevant survival endpoints may be assessed using appropriate statistical methods (e.g. analysis of variance [ANOVA], categorical or survival models), depending on the endpoints. Analyses may be performed within and between each treatment group. Other clinical covariates (such as baseline tumor characteristics and patient demographics) may also be included in the model. Correlation of baseline biomarker expression levels with clinical response or relevant time-to-event endpoints may be performed to identify responsive (or resistant) subgroups. Association of biomarkers with clinical response or relevant time-to-event endpoints will also be explored in the overall population to identify "high-risk" biomarker profiles that are correlated with poor outcome. Appropriate details of these exploratory analyses will be included in the statistical analysis plan. Results of these exploratory analyses will be presented in separate technical reports.

13.4.6 Assessment of Ventricular Repolarization

The assessment of ventricular repolarization will be a substudy conducted in a subset of patients from selected clinical sites and analyzed by an independent cardiac safety laboratory (Appendix 8).

13.4.7 Medical Resource Utilization Analysis

Protocol-mandated procedures, tests, and encounters are excluded. The MRU data may be used to conduct economic analyses.

13.5 INTERIM ANALYSIS

There will be [redacted]; see Section 13.2.2 for an outline of analysis methods.

13.6 DETERMINATION OF SAMPLE SIZE

The primary efficacy analysis will be event-driven and will take place when approximately [redacted] MFS events have occurred. The study provides 90% power to detect a [redacted] reduction in the risk of developing metastases (HR = [redacted]) for patients receiving apalutamide, with a 2-sided α of 0.05. Based on an assumed median MFS of 25 months in the placebo arm, this treatment effect represents an increase in the median MFS of approximately [redacted] months (from 25 months to [redacted] months). Assuming an accrual period of 24 months (with [redacted] of the patients accrued in the second year), approximately 1,200 patients will need to be enrolled.

The study was also sized to provide 85% power to detect a [redacted] reduction (HR = [redacted]) in the risk of death for patients receiving apalutamide, based on an assumed median OS of [redacted] months in the placebo arm. This treatment effect represents an increase in the median OS of approximately [redacted] months (from [redacted] to [redacted] months).

14. DATA COLLECTION, RETENTION AND MONITORING

14.1 DATA COLLECTION INSTRUMENTS

The Investigator will prepare and maintain adequate and accurate source documents designed to record all observations and other pertinent data for each patient treated with the study drug.

Study personnel at each site will enter data from source documents corresponding to a patient's visit into the protocol-specific electronic case report form (eCRF) when the information corresponding to that visit is available. Patients will not be identified by name in the study database or on any study documents to be collected by the Sponsor (or designee), but will be identified by a site number, patient number.

If a correction is required for an eCRF, the time and date stamps track the person entering or updating eCRF data and creates an electronic audit trail.

The Investigator is responsible for reviewing all information collected on patients enrolled in this study for completeness and accuracy. A copy of the eCRF will remain at the Investigator's site at the completion of the study.

14.2 DATA MANAGEMENT PROCEDURES

The data will be entered into a validated database. The Sponsor-designated data management group will be responsible for data processing, in accordance with procedural documentation. Database lock will occur once quality assurance procedures have been completed.

All procedures for the handling and analysis of data will be conducted using good computing practices meeting FDA guidelines for the handling and analysis of data for clinical trials.

14.3 DATA QUALITY CONTROL AND REPORTING

After data have been entered into the study database, a system of computerized data validation checks will be implemented and applied to the database on a regular basis. Queries are entered, tracked, and resolved through the electronic data capture (EDC) system directly. The study database will be updated in accordance with the resolved queries. All changes to the study database will be documented.

14.4 ARCHIVAL OF DATA

The database is safeguarded against unauthorized access by established security procedures; appropriate backup copies of the database and related software files will be maintained. Databases are backed up by the database administrator in conjunction with any updates or changes to the database.

At critical junctures of the protocol (e.g., production of interim and final reports), data for analysis is locked and cleaned per established procedures.

14.5 AVAILABILITY AND RETENTION OF INVESTIGATIONAL RECORDS

To enable evaluations and/or audits from regulatory authorities or the Sponsor, the Investigator agrees to keep records, including the identity of all participating subjects (sufficient information to link records, e.g., CRFs and hospital records), all original signed informed consent forms, copies of all CRFs, safety reporting forms, source documents, and detailed records of treatment disposition, and adequate documentation of relevant correspondence (e.g., letters, meeting minutes, telephone calls reports). The records should be retained by the Investigator according to ICH, local regulations, or as specified in the Clinical Trial Agreement, whichever is longer, but at a minimum, all study documentation must be retained for 2 years after the last marketing application approval in an ICH region or after at least 2 years have elapsed since formal discontinuation of clinical development of apalutamide.

If the Investigator becomes unable for any reason to continue to retain study records for the required period (e.g., retirement, relocation), the Sponsor should be prospectively notified. The study records must be transferred to a designee acceptable to the Sponsor, such as another investigator, another institution, or to the Sponsor itself. The Investigator must obtain the Sponsor's written permission before disposing of any records, even if retention requirements have been met.

14.6 MONITORING

Monitoring visits will be conducted by representatives of the Sponsor according to the US CFR Title 21 Parts 50, 56, and 312 and ICH Guidelines for GCP (E6). By signing this protocol, the Investigator grants permission to the Sponsor (or designee), and appropriate regulatory authorities to conduct on-site monitoring and/or auditing of all appropriate study documentation.

14.7 PATIENT CONFIDENTIALITY

In order to maintain patient confidentiality, only a site number and patient number will identify all study patients on CRFs and other documentation submitted to the Sponsor. Additional patient confidentiality issues (if applicable) are covered in the Clinical Trial Agreement.

Blood samples from approximately 400 patients and archived tumor samples (FFPE tumor blocks or tumor slides) from approximately 400 patients may be collected from consenting patients for scientific research where local regulations permit.

Long-term Storage of Samples for Future Research

Samples collected for biomarker assessments are planned to be stored until testing and for up to 15 years after the end of the study based on local regulations.

15. ADMINISTRATIVE, ETHICAL, REGULATORY CONSIDERATIONS

15.1 INVESTIGATOR RESPONSIBILITIES

The investigator is responsible for ensuring that the study is performed in accordance with current ICH guidelines on Good Clinical Practice (GCP), and applicable regulatory and country-specific requirements.

Good clinical practice is an international ethical and scientific quality standard for designing, conducting, recording, and reporting studies that involve the participation of human subjects. Compliance with this standard provides public assurance that the rights, safety, and well-being of the study subjects are protected, consistent with the principles that originated in the Declaration of Helsinki, and that the study data are credible.

To maintain confidentiality, all laboratory specimens, evaluation forms, reports and other records will be identified by a coded number. All study records will be kept in a locked file cabinet and code sheets linking a patient's name to a patient identification number will be stored separately in another locked file cabinet. Clinical information will not be released without written permission of the patient, except as necessary for monitoring by the FDA. The Investigator must also comply with all applicable privacy regulations (e.g., Health Insurance Portability and Accountability Act of 1996, EU Data Protection Directive 95/46/EC).

15.2 PROTOCOL AMENDMENTS

Any amendment to the protocol will be written by the Sponsor. Protocol amendments cannot be implemented without prior written IRB/IEC approval except as necessary to eliminate immediate safety hazards to patients. A protocol amendment intended to eliminate an apparent immediate hazard to patients may be implemented immediately, provided the IRB/IECs are notified within five working days.

15.3 INSTITUTIONAL REVIEW BOARDS AND INDEPENDENT ETHICS COMMITTEES

The protocol, Investigator's Brochure, the consent forms, any information to be given to the patient (including patient recruitment materials) and relevant supporting information must be submitted to the IRB/IEC by the Investigator for review and approval before the study is initiated. Any member of the IRB/IEC who is directly affiliated with this study as an Investigator or as site personnel must abstain from the IRB/IEC vote on the approval of the protocol. The IRB/IECs unconditional approval statement will be transmitted by the Investigator to the Sponsor (or designee) prior to the shipment of study supplies to the site. This approval must refer to the study by exact protocol title and number and should identify the documents reviewed and the date of review.

Protocol and/or informed consent modifications or changes may not be initiated without prior written IRB/IEC approval except when necessary to eliminate immediate hazards to the patients or when the change(s) involves only logistical or administrative aspects of the study. Such modifications will be submitted to the IRB/IEC and written verification that the modification was submitted and subsequently approved should be obtained.

Investigators are required to promptly report to their respective IRB/IEC all unanticipated problems involving risk to human patients. Some IRBs/IECs may want prompt notification of all SAEs, whereas others require notification only about events that are serious, assessed to be related to study treatment, and are unexpected. Investigators may receive written IND safety reports or other safety-related communications from the Sponsor. Investigators are responsible for ensuring that such reports are reviewed and processed in accordance with regulatory requirements and with the policies and procedures established by their IRB/IEC and archived in the site's study file.

Finally, the Investigator will keep the IRB/IEC informed as to the progress of the study, revisions to documents originally submitted for review, annual updates and/or request for re-approvals, and when the study has been completed.

15.4 INFORMED CONSENT FORM

Informed consent will be obtained in accordance with principles that originated in the Declaration of Helsinki, current ICH and GCP guidelines, applicable regulatory requirements, and Sponsor policy.

The Sponsor's master ICF will be provided to each site. Sponsor or its designee must review and approve any proposed deviations from the master ICF or any alternate consent forms proposed by the site before IRB/IEC submission. Patients must be re-consented to the most current version of the consent forms during their participation in the study. The final IRB/IEC-approved consent forms must be provided to Sponsor for regulatory purposes.

The ICFs must be signed by the patient or the patient's legal representative before his participation in the study. The case history for each patient shall document the informed consent process and that written informed consent was obtained prior to participation in the study. A copy of each signed ICF must be provided to the patient or the patient's legal

representative. If applicable, it will be provided in a certified translation of the local language.

All signed and dated consent forms must remain in each patient's study file and must be available for verification by study monitors at any time.

The ICF should be revised whenever there are changes to procedures outlined in the informed consent or when new information becomes available that may affect the willingness of the patient to participate.

For any updated or revised consent forms, the case history for each patient shall document the informed consent process and that written informed consent was obtained for the updated/revised consent form for continued participation in the study. The final revised IRB/IEC-approved Informed Consent Form must be provided to Sponsor for regulatory purposes.

15.5 REPORTING OF SAFETY ISSUES AND SERIOUS BREACHES OF THE PROTOCOL OR ICH GCP

In the event of any prohibition or restriction imposed (i.e., clinical hold) by an applicable Competent Authority in any area of the World, or if the Investigator is aware of any new information which might influence the evaluation of the benefits and risks of the investigational product, the Sponsor should be informed immediately.

In addition, the Investigator will inform the Sponsor immediately of any urgent safety measures taken by the Investigator to protect the study subjects against any immediate hazard, and of any serious breaches of this protocol or of ICH GCP that the Investigator becomes aware of.

15.6 END OF TRIAL IN ALL PARTICIPATING COUNTRIES

Patients will remain on study treatment until BICR-confirmed disease progression, development of unacceptable toxicity, or withdrawal of consent. Patients discontinuing study treatment will enter the Long-term Follow-up Phase and remain on study until death, loss of follow-up, or withdrawal of consent, whichever comes first.

With an estimated accrual duration of 24 months, it is assumed that patients are expected to be followed for a minimum of approximately [redacted] months beyond Last Patient In (LPI) for the primary endpoint of MFS, to approximately [redacted] months beyond LPI for the key secondary endpoint of OS. This corresponds to total projected study duration of approximately 65 months.

If the study is not terminated beforehand per the recommendation of the IDMC, the end of trial in all participating countries will be defined as the time at which the secondary endpoint of OS has been met.

15.7 SPONSOR DISCONTINUATION CRITERIA

Premature termination of this study may occur because of a regulatory authority decision, change in opinion of the IRB/IEC, drug safety problems, or at the discretion of the Sponsor.

In addition, the Sponsor retains the right to discontinue development of apalutamide at any time.

If a study is prematurely terminated or discontinued, the Sponsor will promptly notify the Investigator. After notification, the Investigator must notify the respective IRB/IEC, and contact all participating subjects and the hospital pharmacy (if applicable) within a 4-week time period. As directed by the Sponsor, all study materials must be collected and all CRFs completed to the greatest extent possible.

15.8 PUBLICATIONS

Publication of study results is discussed in the Clinical Trial Agreement. Details regarding production of manuscripts and conference presentations will adhere to the International Committee of Medical Journal Editors (ICMJE) requirements for authorship and contributorship.

http://www.icmje.org/ethical_1author.html

16. REFERENCES

1. Appendix 1 to the guideline on the evaluation of anticancer medicinal products in man: Methodological consideration for using progression-free survival (PFS) or disease-free survival (DFS) in confirmatory trials. European Medicines Agency 2011; Doc. Ref. EMA/CHMP/27994/2008.
2. Chen CD, Welsbie DS, Tran C, et al. Molecular determinants of resistance to antiandrogen therapy. Nat Med 2004;10:33-9.
3. Clegg N, Wongvipat J, Joseph J, et al. Discovery and development of ARN-509, a novel anti-androgen for the treatment of prostate cancer. Cancer Res 2012; 2:1-10.
4. Eisenhauer EA, Therasse P, Bogaerts J, et al. New response evaluation criteria in solid tumors: revised RECIST guideline (version 1.1). Eur J Cancer 2009;45:228-47.
5. Esper P, Mo F, Chodak G, et al. Measuring quality of life in men with prostate cancer using the Functional Assessment of Cancer Therapy-Prostate (FACT-P) instrument. Urology 1997;50(6):920-8.
6. Foster WR, Car BD, Shi H, et al. Drug safety is a barrier to the discovery and development of new androgen receptor antagonists. Prostate 2011;71:480-8.
7. Freedland SJ, Humphreys EB, Mangold LA, et al. Risk of prostate cancer-specific mortality following biochemical recurrence after radical prostatectomy. JAMA 2005;294:433-439.
8. Freedland SJ, Humphreys EB, Mangold LA, et al. Death in patients with recurrent prostate cancer after radical prostatectomy: prostate-specific antigen doubling time subgroups and their associated contributions to all-cause mortality. J Clin Oncol 2007;25:1765-71.
9. Jemal A, Bray F, Center MM, et al. Global cancer statistics. Ca Cancer J Clin 2011;61:69-90.
10. Joseph JD, Lu N, Qian J, et al., A clinically relevant androgen receptor mutation confers resistance to 2nd generation anti-androgens enzalutamide and ARN-509. Cancer Disc 2013;3:1020-1029.
11. Kelly WK, Slovin S, Scher HI. Steroid hormone withdrawal syndromes: pathophysiology and clinical significance. Urol Clin North Am 1997;24:421-33.
12. Korpal M, Korn JM, Gao X, et al. An F876L mutation in the androgen receptor confers genetic and phenotypic resistance to MDV3100 (enzalutamide). Cancer Disc 2013;3:1030-1043.
13. Li Y, Chan SC, Brand LJ, et al. Androgen receptor splice variants mediate enzalutamide resistance in castration-resistant prostate cancer cell lines. Cancer Res 2013;73:483-489.

14. National Comprehensive Cancer Network (NCCN) Practice Guidelines in Oncology – v.1.2010.

15. Norgaard M, Jensen AO, Jacobsen JB et al. Skeletal related events, bone metastasis and survival of prostate cancer: a population-based cohort study in Denmark (1999 to 2007). J Urol 2010;184:162-67.

16. Pinover WH, Horwitz EM, Hanlon AL et al. Validation of a Treatment Policy for Patients with Prostate Specific Antigen Failure after Three-Dimensional Conformal Prostate Radiation Therapy. Cancer 2003;97:1127-33.

17. Pound CR, Partin AW, Eisenberger MA, et al. Natural history of progression after PSA elevation following radical prostatectomy. JAMA 1999;281:1591-7.

18. Rathkopf D, Liu G, Carducci MA, et al. Phase I dose-escalation study of the novel antiandrogen BMD-641988 in patients with castration-resistant prostate cancer. Clin Cancer Res 2011;17:880-7.

19. Rathkopf DE, Morris MJ, Fox JJ et al. Phase I study of ARN-509, a novel antiandrogen, in the treatment of castration-resistant prostate cancer. J Clin Oncol 2013;31:3525-3530.

20. Rathkopf DE, Antonarakis ES, Shore ND, et al. ARN-509 in men with metastatic castration-resistant prostate cancer. J Clin Oncol 2013;31(suppl 6; abstact 48).

21. Ray ME, Bae K, Hussain MH et al. Potential surrogate endpoints for prostate cancer survival: Analysis of a Phase III randomized trial. J Natl Cancer Inst 2009;101:228-236.

22. Sathiakumar N, Delzell E, Morrisey MA et al. Mortality following bone metastasis and skeletal-related events among men with prostate cancer: a population-based analysis of U.S. Medicare beneficiaries, 1999-2006. Prostate Cancer Prostat Dis 2011;14:177-83.

23. Scher HI, Sawyers CL. Biology of progressive, castration-resistant prostate cancer: directed therapies targeting the androgen-receptor signaling axis. J Clin Oncol 2005; 23:8253-61.

24. Scher HI, Halabi S, Tannock I, et al. Design and end points of clinical trials for patients with progressive prostate cancer and castrate levels of testosterone: recommendations of the prostate cancer clinical trials working group. J Clin Oncol 2008;26:1148-59.

25. Smith MR, Kabbinavar F, Saad F, et al. Natural history of rising serum prostate-specific antigen in men with castrate nonmetastatic prostate cancer. J Clin Oncol 2005;23:2918-25.

26. Smith MR, Cook R, Lee KA, Nelson JB. Disease and host characteristics as predictors of time to first bone metastasis and death in men with progressive castration-resistant nonmetastatic prostate cancer. Cancer 2011;117:2077-85.

27. Smith MR, Saad F, Coleman R, et al. Denosumab and bone-metastasis-free survival in men with castration-resistant prostate cancer: results of a phase 3, randomised, placebo-controlled trial. Lancet 2012;379:39-46.

28. Smith M, Antonarakis ES, Ryan CJ, et al. ARN-509 in men with high-risk non-metastatic castration-resistant prostate cancer (CRPC). J Clin Oncol 2013;31(suppl 6, abstract 7).

29. Stephenson AJ, Kattan MW, Eastham JA, et al. Defining biochemical recurrence of prostate cancer after radical prostatectomy: a proposal for a standardized definition. J Clin Oncol 2005;23:8253-61.

30. The EuroQol Group. EuroQol-a new facility for the measurement of health-related quality of life. Health Policy 1990;16:199-208.

17. APPENDICES

Appendix 1 Schedule of Activities

Appendix 2 Required Laboratory Tests

Appendix 3 ECOG Performance Status

Appendix 4 National Cancer Institute (NCI) Common Terminology Criteria for Adverse Events (CTCAE)

Appendix 5 Prohibited or Restricted Medications or Supplements While On Study

Appendix 6 FACT-P Questionnaire

Appendix 7 EQ-5D

Appendix 8 Ventricular Repolarization Substudy at Selected Sites

Appendix 9 Anticipated Events

Appendix 10 Crossover to Open Label Apalutamide After Study Unblinding

APPENDIX 1: SCHEDULE OF ACTIVITIES

	Prescreening [1]	Screening	Treatment Phase			Posttreatment		
**Activities and Forms to be Completed**		**≤35 Days Prior to Randomization**	**Cycle 1 Day 1**	**D1 C1-C6, D1 of every 2 cycles starting at C7 to C13, then D1 of every 4 cycles unless otherwise specified**	**Every 16 weeks (Starting on C1D1)**	**End-of-Treatment [2]**	**Safety Follow-up [3]**	**Long-term Follow-up [4]**
**Screening**								
Informed Consent	X	X						
Medical/Oncological History [5]		X						
Inclusion/Exclusion Criteria		X						
Randomization [6]		X						
**Study Drug Administration**								
Apalutamide/Matched Placebo Administration [7]			X→	→				
Study Drug Compliance [8]				X		X		
**Laboratory Studies**								
Hematology [9]		X	X	X		X		
Blood Chemistry [9]		X	X	X		X		
PSA [10]	X	X	X	X		X		
Testosterone [11]		X	X		X	X		
Thyroid-Stimulating Hormone [11]		X	X		X	X		
Fasting Lipid Panel [11]		X	X		X	X		
**Efficacy**								
ECOG		X	X	X		X		
CT brain [12]		X						
CT chest, abdomen, and pelvis [4,13]		X			X	X		X
Bone scan [4, 13]		X			X	X		

	Prescreening [1]	Screening	Treatment Phase			Posttreatment		
**Activities and Forms to be Completed**		**≤35 Days Prior to Randomization**	**Cycle 1 Day 1**	**D1 C1-C6, D1 of every 2 cycles starting at C7 to C13, then D1 of every 4 cycles unless otherwise specified**	**Every 16 weeks (Starting on C1D1)**	**End-of-Treatment [2]**	**Safety Follow-up [3]**	**Long-term Follow-up [4]**
Medical Resource Utilization			X	X				X
Progression on first subsequent therapy [14]								X
Survival [4]								X
**Safety**								
Physical Examination [15]		X	X	X		X		
Vital Signs [16]		X	X	X		X		
12-lead ECG [17]		X						
Adverse Events	X [18]	Continuous from informed consent until 28 days after the last dose of study drugs						
Concomitant Medications [19]		Continuous until 28 days after the last dose of study drugs						
**Population Pharmacokinetics**								
PK plasma sample [20]			Between 0.5 and 4 hours post-dose	D1 of C2, C3, C6, C11, C17, and C25				
**Patient Reported Outcomes**								
FACT-P and EQ-5D [21]			X	X		X		**X**
**Exploratory Biomarkers**								
Archival FFPE blocks/slides [22]			X					
Biomarker blood sample [22]			X	D1 of C11, C17, C25, and C37		X		

C1D1=Cycle 1 Day 1; BICR=blinded independent central review; CT=computed tomography; ECG=electrocardiogram; ECOG=Eastern Cooperative Oncology Group; EQ-5D=Euro QoL; FACT-P=functional assessment of cancer therapy-prostate; FFPE=formalin-fixed paraffin-embedded; PSA=prostate specific antigen

Footnotes

1.	**Prescreening:** Prior to study entry, additional PSA evaluations may be necessary to determine eligibility. This Prescreening Phase is optional; please also refer to prescreen informed consent.
2.	**End-of-Treatment:** These assessments do not need to be completed if they have been performed within 1 week of the last dose of study drug (Exception: within the last 8 weeks for tumor assessments and 2 weeks for PROs, respectively).
3.	**Safety Follow-Up:** Patients should be evaluated for safety up to 28 days after the last dose of study drug. All AEs should be followed to their resolution, until the Investigator assesses them as stable, irreversible, or until the patient is lost to follow-up, whichever comes first. If a patient begins a new systemic anti-cancer therapy, the AE reporting period for non-SAEs ends at the time the new treatment is started. Serious adverse events will be collected during treatment with abiraterone acetate (as first subsequent therapy). Note: See Section 8.10 for additional details. Treatment with abiraterone acetate does not apply in Japan.
4.	**Long-term Follow-up:** Obtain survival status, collect information of development of symptomatic progression, initiation of any new systemic anti-cancer therapies, and progression on first subsequent therapy (every 4 months via clinic visit, telephone contact or an alternative contact method per institution policy/practice). The FACT-P and EQ-5D questionnaires will be collected up to 12 months post-progression (see also Footnote 21). In addition, if patients discontinued study treatment prior to documented disease progression, obtain CT of the chest, abdomen, and pelvis, plus bone scan and submit for BICR to evaluate for disease progression (every 16 weeks ±7 days), until documentation of disease progression).
5.	**Medical/Oncological History:** Includes oncologic history, demographics, history of other disease processes (active or resolved) and concomitant illnesses.
6.	**Study Randomization:** The Cycle 1 Day 1 visit can occur on the same day or within 4 days of randomization. Patient number and treatment assignment will be obtained via centralized randomization through the IVRS. PSADT will be calculated by the IVRS to ensure correct patient stratification.
7.	**Apalutamide/Matched Placebo:** Patients will receive oral daily apalutamide or matched placebo continuously, starting on Cycle 1 Day 1. One cycle consists of 28 days.
8.	**Study Drug Compliance:** Apalutamide and placebo bottle(s) including any unused tablets/capsules will be returned at the beginning of every cycle starting at Cycle 2 Day 1 and at end of treatment for drug accountability.
9.	**Samples for Hematology, Blood Chemistry:** All laboratory assessments will be performed by a central laboratory. PSA results will be kept blinded until the analysis of the primary endpoint. On Cycle 1 Day 1 only, clinical laboratory tests do not need to be repeated if the Screening tests were done within 4 days of Cycle 1 Day 1. Sites may perform additional local hematology and/or blood chemistry assays for the purposes of planning treatment administration, dose modification, or monitoring adverse events.
10.	**Samples for PSA:** Optional prescreening PSA evaluations will be performed by local laboratories. All other PSA valuations will be collected by the central laboratory; patients and investigators will be kept blinded until the analysis of the primary endpoint. On Cycle 1 Day 1 only, the PSA evaluation does not need to be repeated if the Screening tests were done within 4 days of Cycle 1 Day 1. For all subjects, at the time of unblinding and crossover, PSA data submitted to the central laboratory will no longer be blinded.
11.	**Samples for TSH, fasting lipid panel, and testosterone:** All laboratory assessments will be performed by a central laboratory every 16 weeks (±2 days) from Cycle 1 Day 1. On Cycle 1 Day 1 only, clinical laboratory tests do not need to be repeated if the Screening tests were done within 4 days of Cycle 1 Day 1. Sites may perform additional local assays for the purposes of planning treatment administration, dose modification, or monitoring adverse events. See Appendix 2 for details on additional testing if TSH is abnormal.
12.	**Brain Imaging:** CT (or MRI, if use of contrast agent is contraindicated) scan of the brain will be performed at Screening and submitted for BICR to confirm patient eligibility (absence of brain metastasis).

**13.**	**Tumor Imaging:** CT scans of chest, abdomen, and pelvis plus bone scan will be performed at Screening (scans obtained prior to signing informed consent will be allowed provided the timing of the scans fall within the Screening window). Scans must be submitted for BICR to confirm patient eligibility. During treatment, CT scans of chest, abdomen and pelvis plus bone scan will be performed to assess disease status every 16 weeks (±7 days) from Cycle 1 Day 1, or whenever disease progression is suspected, and at the end of treatment. Note that tumor imaging should continue on this calendar schedule regardless of any delays in dosing. Patients who have a contraindication to IV contrast may have MRI exams of the brain, abdomen, and pelvis performed in lieu of CTs and a non-contrast CT of the chest. All scans will be submitted for blinded independent central review to confirm patient eligibility and assess for disease progression on study. Subjects who discontinue treatment before documented disease progression should continue with disease assessments during Long-term Follow-up every 16 weeks (±7 days) until disease progression (see also Footnote 4). For all subjects, at the time of unblinding and crossover, radiographic scans will no longer be to be submitted for blinded central independent review. Subjects will be followed for progression per Investigator decision including symptoms, radiographic scans or PSA.
**14.**	**Progression on First Subsequent Therapy:** The date of progression on first subsequent therapy as assessed by the investigator and method of assessment of disease progression (radiographic, PSA, or both) will be collected.
**15.**	**Physical Examination:** At Screening, a complete physical examination of major body systems, including known and suspected sites of disease, should be performed. During subsequent visits, either a full or abbreviated physical exam will be performed. New abnormal physical exam findings must be documented and followed by a physician or other qualified staff at the next scheduled visit. Height will be recorded at Screening only. Body weight will be recorded at Screening and at every scheduled visit during treatment and at the end of treatment.
**16.**	**Vital Signs:** Body temperature, blood pressure, pulse and respiratory rate will be performed after resting for 5 minutes at Screening and every scheduled visit during treatment and at the end of treatment.
**17.**	**ECG:** A standard 12-lead ECG will be collected at Screening and as clinically indicated. For patients on QTcF substudy, please refer to Appendix 8 for instructions on obtaining a screening ECG.
**18.**	**Adverse Events**: During this Prescreening Phase, the SAE reporting would be limited to SAEs related to the PSA blood draws.
**19.**	**Concomitant Medications/Treatments**: Concomitant medications and/or treatments will be recorded during the 28-day Screening Phase (prior to the start of study treatment), during the study, and up to 28 days post the last dose of study treatment.
**20.**	**PK Samples for Population PK Analyses**: The Day 1 Cycle 1 sample will be collected between 0.5 and 4 hours postdose. All samples on Day 1 of the other cycles will be collected prior to study drug administration, see Section 8.4). Record the time for the doses administered on the 2 days preceding the PK sample day and whether the dose was administered on a once daily or twice daily schedule. No sparse PK samples will be collected from patients participating in the ventricular repolarization study (see Appendix 8). For all subjects, at the time of unblinding and crossover, PK samples will no longer need to be collected at the time of unblinding.
**21.**	**FACT-P and EQ-5D questionnaires:** Patients will complete the FACT-P and EQ-5D at the clinic during the Treatment Phase **PRIOR to any other clinical activity**. During Long-term Follow-up contact every 4 months via clinic visit or an alternative contact method per institution policy/practice up to 12 months post-progression.
**22.**	**Biomarker samples:** Archived FFPE tumor samples or tumor slides will be requested from a subset of approximately 400 patients and can be submitted at any time after Cycle 1 Day 1. Blood samples, 12.5 mL (on Day 1 of Cycles 1, 11 and end of treatment) and 10 mL (on Day 1 of Cycles 17, 25, and 37) will be collected prior to study drug administration from a subset of approximately 400 patients. Samples should be frozen and sent to the central laboratory.

APPENDIX 2: REQUIRED LABORATORY TESTS

Hematology (See Appendix 1 for timing)	Chemistry and PSA (See Appendix 1 for timing)	Other (every 16 weeks from C1D1)
Hemoglobin<br>Platelet count<br>Red blood cell count<br>White blood cell count<br>White blood cell differential	Total bilirubin (Note: if > 1.5 x ULN, include analysis of direct and indirect bilirubin)<br>Alanine transaminase (ALT)<br>Aspartate transaminase (AST)<br>Alkaline phosphatase<br>Albumin<br>Sodium<br>Potassium<br>Calcium<br>Magnesium<br>Blood urea nitrogen (BUN) or urea<br>Creatinine<br>Glucose<br>Prostate specific antigen (PSA)	Testosterone<br>Thyroid stimulating hormone (TSH); If TSH is abnormal; total T3, free T4 (direct), and total T4 are required.<br><br>Fasting lipid panel

APPENDIX 3: ECOG PERFORMANCE STATUS GRADES

0 Fully active, able to carry on all pre-disease activities without restriction

1 Restricted in physically strenuous activity but ambulatory and able to carry out work of a light or sedentary nature, e.g., light house work or office work

2 Ambulatory and capable of all selfcare but unable to carry out any work activities. Up and about more than 50% of waking hours

3 Capable of only limited selfcare, confined to bed or chair more than 50% of waking hours

4 Completely disabled. Cannot carry on any selfcare. Totally confined to bed or chair

5 Dead

APPENDIX 4: NATIONAL CANCER INSTITUTE (NCI) COMMON TERMINOLOGY CRITERIA FOR ADVERSE EVENTS (CTCAE)

The NCI CTCAE (Version 4.0) may be reviewed on-line at the following NCI website:

http://ctep.cancer.gov/reporting/ctc.html

APPENDIX 5: PROHIBITED OR RESTRICTED MEDICATIONS OR SUPPLEMENTS WHILE ON STUDY

Medications that are PROHIBITED while on study:

- Aminophylline/theophylline
- Atypical antipsychotics (e.g., clozapine, olanzapine, risperidone, ziprasidone)
- Buproprion
- Lithium
- Meperidine and pethidine
- Phenothiazine antipsychotics (e.g., chlorpromazine, mesoridazine, thioridazine)
- Tricyclic and tetracyclic antidepressants (e.g., amitriptyline, desipramine, doxepin, imipramine, maprotiline, mirtazapine)

Supplements that are RESTRICTED while on study:

- Pomegranate

Medications that are RESTRICTED while on study:

Investigators should refer to the apalutamide Investigator's Brochure (Sections 4.3.3.3 and 5.10) and associated addenda for complete details on the drug interaction potential of apalutamide. Highlights of drug interaction are summarized below.

- Strong CYP3A4 inducers: the potential for drug-drug interactions with apalutamide has not been tested clinically. Strong inducers of CYP3A4 (eg, phenytoin, carbamazepine, rifampin, rifabutin, rifapentine, phenobarbital, efavirenz, tipranavir, St. John's wort) should be avoided as much as possible.
- Apalutamide may also induce CYP3A4; therefore, caution should be taken when administered in conjunction with CYP3A4 substrates that have a narrow therapeutic index
- Strong CYP2C8 inhibitors (eg, gemfibrozil) should be used with caution with apalutamide
- The potential for drug-drug interaction between apalutamide and warfarin (e.g., Coumadin) is unknown at present. If a patient is taking warfarin, re-assess (prothrombin) PT/(international normalized ratio) INR as clinically indicated and adjust the dose of warfarin accordingly.
- Corticosteroids (Oral, IV, or IM): due to possible resistance mechanisms, which may be contributed by glucocorticoid receptor signaling, concurrent use of corticosteroids during the study is not recommended; short term use (≤ 4 weeks) will be allowed if clinically indicated, however, its use must be tapered off as soon as possible.

Additional Information on CYP450 Drug Interactions

http://www.fda.gov/drugs/developmentapprovalprocess/developmentresources/druginteractionslabeling/ucm093664.htm

http://medicine.iupui.edu/clinpharm/ddis/table.aspx

APPENDIX 6: FACT-P QUESTIONNAIRE

Version 4, US English, Copyright 1987, 1997

Below is a list of statements that other people with your illness have said are important. By circling one (1) number per line, please indicate how true each statement has been for you <u>during the past 7 days.</u>

	**PHYSICAL WELL –BEING**	**Not at all**	**A little bit**	**Some-what**	**Quite a bit**	**Very much**
GP1	I have a lack of energy	0	1	2	3	4
GP2	I have nausea	0	1	2	3	4
GP3	Because of my physical condition, I have trouble meeting the needs of my family	0	1	2	3	4
GP4	I have pain	0	1	2	3	4
GP5	I am bothered by side effects of treatment	0	1	2	3	4
GP6	I feel ill	0	1	2	3	4
GP7	I am forced to spend time in bed	0	1	2	3	4

	**SOCIAL/FAMILY WELL-BEING**	**Not at all**	**A little bit**	**Some-what**	**Quite a bit**	**Very much**
GS1	I feel close to my friends	0	1	2	3	4
GS2	I get emotional support from my family	0	1	2	3	4
GS3	I get support from my friends	0	1	2	3	4
GS4	My family has accepted my illness	0	1	2	3	4
GS5	I am satisfied with family communication about my illness	0	1	2	3	4
GS6	I feel close to my partner (or the person who is my main support)	0	1	2	3	4
Q1	*Regardless of your current level of sexual activity, please answer the following question. If you prefer not to answer it, please check this box ☐ and go to the next section.*	0	1	2	3	4
GS7	I am satisfied with my sex life	0	1	2	3	4

	EMOTIONAL WELL –BEING	Not at all	A little bit	Some-what	Quite a bit	Very much
GE1	I feel sad	0	1	2	3	4
GE2	I am satisfied with how I am coping with my illness	0	1	2	3	4
GE3	I am losing hope in the fight against my illness	0	1	2	3	4
GE4	I feel nervous	0	1	2	3	4
GE5	I worry about dying	0	1	2	3	4
GE6	I worry that my condition will get worse	0	1	2	3	4

	FUNCTIONAL WELL –BEING	Not at all	A little bit	Some-what	Quite a bit	Very much
GF1	I am able to work (include work at home)	0	1	2	3	4
GF2	My work (include work at home) is fulfilling	0	1	2	3	4
GF3	I am able to enjoy life	0	1	2	3	4
GF4	I have accepted my illness	0	1	2	3	4
GF5	I am sleeping well	0	1	2	3	4
GF6	I am enjoying the things I usually do for fun	0	1	2	3	4
GF7	I am content with the quality of my life right now	0	1	2	3	4

	ADDITIONAL CONCERNS	Not at all	A little bit	Some-what	Quite a bit	Very much
C2	I am losing weight	0	1	2	3	4
C6	I have a good appetite	0	1	2	3	4
P1	I have aches and pains that bother me	0	1	2	3	4
P2	I have certain parts of my body where I experience significant pain	0	1	2	3	4
P3	My pain keeps me from doing things I want to do	0	1	2	3	4
P4	I am satisfied with my present comfort level	0	1	2	3	4
P5	I am able to feel like a man	0	1	2	3	4
P6	I have trouble moving my bowels	0	1	2	3	4
P7	I have difficulty urinating	0	1	2	3	4
BL2	I urinate more frequently than usual		1	2	3	4
P8	My problems with urinating limit my activities	0	1	2	3	4
BL5	I am able to have and maintain an erection	0	1	2	3	4

APPENDIX 7: EQ-5D

By placing a checkmark in one box in each group below, please indicate which statements best describe your own health state today.

Mobility

I have no problems in walking about ❑

I have some problems in walking about ❑

I am confined to bed ❑

Self-Care

I have no problems with self-care ❑

I have some problems washing or dressing myself ❑

I am unable to wash or dress myself ❑

Usual Activities *(e.g., work, study, house work, family, or leisure activities)*

I have no problems with performing my usual activities ❑

I have some problems with performing my usual activities ❑

I am unable to perform my usual activities ❑

Pain/Discomfort

I have no pain or discomfort ❑

I have moderate pain or discomfort ❑

I have extreme pain or discomfort ❑

Anxiety/Depression

I am not anxious or depressed ❑

I am moderately anxious or depressed ❑

I am extremely anxious or depressed ❑

To help people say how good or bad a health state is, we have drawn a scale (rather like a thermometer) on which the best state you can imagine is marked 100 and the worst state you can imagine is marked 0.

We would like you to indicate on this scale how good or bad your own health is today, in your opinion. Please do this by drawing a line from the box below to whichever point on the scale indicates how good or bad your health state is today.

Your own health state today

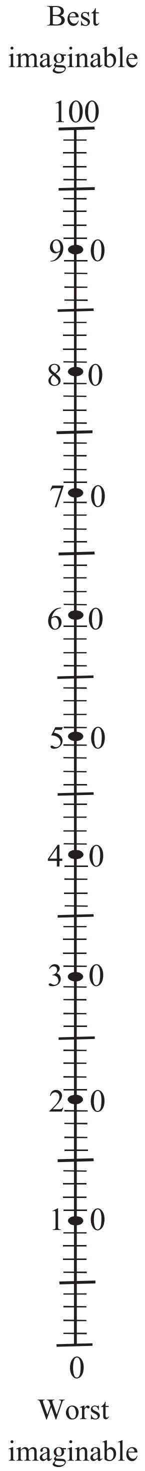

APPENDIX 8: VENTRICULAR REPOLARIZATION SUBSTUDY AT SELECTED SITES

Study Objectives

To evaluate the effect of apalutamide on ventricular repolarization in a subset of patients from selected clinical sites:

- The primary objective of this substudy is to evaluate whether apalutamide has a threshold pharmacologic effect on cardiac repolarization, as detected by changes in electrocardiogram (ECG) QT intervals corrected for heart rate by Fridericia's correction method (QTcF)
- The secondary objectives of this study substudy are as follows:
    - To investigate the effect of apalutamide on the following ECG parameters: PR, RR, QRS, QT, QTcB (Bazett's correction method), and T-Wave morphology
    - To determine the relationship between the plasma concentrations of apalutamide and metabolite ARN000308, and QT/QTc changes

Safety will be assessed as part of the main study. At the time of the analysis of this substudy, all safety data from the main study will be provided along with data specific to this subset of patients. In particular, any cardiac-related adverse events will be summarized in both main study and substudy populations.

Study Design and Patient Population

The effect of apalutamide on ventricular repolarization will be centrally analyzed by a third-party cardiac safety laboratory in a subset of 100 patients enrolled at selected sites that will be participating in the main protocol. Both apalutamide and placebo patients will be enrolled in a blinded manner as per the main protocol randomization criteria.

The subset of 100 patients will undergo the same screening procedures as the main protocol in order to be randomized into the study, following the same Inclusion/Exclusion criteria as per Section 4 of the protocol, **with the following additional enrollment criteria**:

Additional Inclusion Criteria	Additional Exclusion Criteria
▪ Enrollment in the main study<br>▪ Obtain separate informed consent for participation in the substudy<br>▪ Must agree to fast at least 3 hours prior to dose and continue fasting (except for approved snack) until completion of the 4 hour post dose assessments on Cycle 1 Day 1 and Cycle 3 Day 1.	▪ Heart rate outside of 50 to 100 beats/minute<br>▪ QTcF > 480 msec, determined by central assessment<br>▪ Diagnosed or suspected congenital long QT syndrome, or family history of congenital long QT syndrome or sudden death<br>▪ History of Mobitz II second degree or third degree heart block<br>▪ Implantable pacemaker or automatic implantable cardioverter defibrillator<br>▪ Complete Bundle Branch Block or ventricular conduction delay (QRS > 119 msec)<br>▪ Chronic or persistent atrial arrhythmia, including atrial fibrillation and atrial flutter.<br>▪ Concurrent therapy with medications known to prolong the QT interval and/or associated with TdP (Torsade de Pointes) arrhythmia [please refer to www.qtdrugs.org for the list of drugs to avoid]<br>▪ Smokers and planned nicotine replacement therapy users

Note: Sites will receive a notification report for any ECGs received above QTcF > 480msec. If patients do not qualify for the ventricular repolarization substudy, they can still participate in the main study provided they meet all other inclusion/exclusion criteria as per Section 4 of the protocol.

Patient Withdrawal: A patient may withdraw his consent to participate in the substudy at any time. If a patient withdraws such consent, the Investigator should inform the Sponsor (or designee) in writing and document in the Investigator Site File. The patient may continue participating in the main protocol.

Rationale for the Study Design

This substudy has been designed to evaluate the potential of apalutamide to prolong the QTc interval in a subset of patients with high-risk, NM-CRPC participating in the main protocol, Study ARN-509-003.

In accordance with ICH E14 guideline, QT evaluation is now expected to be routine in oncology drug development, and a Thorough QT (TQT) study should be conducted, if possible. In the case of apalutamide, in view of previous FDA advice and the observations to date that nonclinical (hERG and CV safety study) and clinical data (ECG collections in the Phase I/II Study ARN-509-001) suggest no apparent relationship between apalutamide and QT prolongation, an alternative design to the TQT study has been chosen. In this substudy, changes in QT interval following drug administration will be evaluated relative to the baseline measurement.

Rationale for the ECG Collection Time Points and PK Sampling Schedule

ECG time points have been selected to match the expected PK profile of apalutamide and metabolite (ARN000308). The ECG time points have also been selected to explore a potential shift between exposure and effect on QT/QTc.

In order to assess the QT interval prior to exposure to apalutamide, baseline triplicate ECG assessments will be performed twice prior to first study drug administration on Cycle 1 Day 1 (at hour -1 and hour 0 pre-dose). A baseline PK sample will also be collected at the hour 0, immediately after the ECGs have been collected and just prior to the first dose of apalutamide or placebo.

Subsequently, the purpose of all other ECGs is to assess for potential prolongation of the QT interval and other ECG changes as a result of apalutamide administration, with ECG collection coinciding with PK measurements to establish correlations between ECG changes and drug exposure. Triplicate ECGs, followed by blood samples for PK assessment, will be collected at 2 and 4 hours postdose on Cycle 1 Day 1 and Cycle 3 Day 1 (once steady-state can be assured). The 2- and 4-hour time points were selected because the oral plasma T_{max} of apalutamide is generally between 2 and 4 hours post-dose. Although the primary metabolite of apalutamide (ARN000308) typically has a later C_{max} than that of apalutamide (i.e., [redacted] to [redacted] hours), the peak to trough fluctuation ratio of this metabolite in patient plasma was shown to be minimal (less than [redacted] for ARN000308). Therefore, the potential for significant effects from this metabolite at later time points not assessed by ECG is deemed very small.

Methods

Digital 12-lead ECG equipment will be provided to each clinical site participating in this substudy by the central laboratory for the duration of the substudy.

ECGs should be collected after the patient has rested quietly and is awake in a fully supine (or semi-recumbent, if supine is not tolerated) position for 10 minutes, and prior to any blood collection. For each patient the same position (e.g., supine or semi-recumbent) should be used for all ECGs collected. Starting on Cycle 1, Day 1, time point matched blood samples for PK analyses will be collected immediately following the collection of ECGs and before the collection of blood for all other clinical evaluations. ECGs will be read by independent cardiologists from the central laboratory in a blinded manner and via single reader paradigm.

A detailed list of the required assessments is provided below. All other assessments at the other time points will follow the main protocol as per the Schedule of Activities in Appendix 1.

Schedule of Activities Specific to the Ventricular Repolarization Substudy:

- ☐ **Screening**
  - Signed informed consent for participation in this substudy
  - Collect a set of triplicate 12-lead ECGs, 2 minutes apart (Holter Monitoring using a 12-lead ambulatory device)

- ☐ **Cycle 1, Day 1**
  - One hour prior to administering the first dose of study drug, collect a set of triplicate 12-lead ECGs, 2 minutes apart
  - At time 0, just prior to administering the first dose of study drug, collect a second set of triplicate 12-lead ECGs, 2 minutes apart, and one blood sample for PK analysis
  - Administer study drug in clinic
  - At 2 and 4 hours postdose, collect a set of triplicate ECGs, 2 minutes apart, and 1 blood sample for PK analysis for each time point, respectively.

- ☐ **Cycle 3, Day 1 (Administer Study Drug in Clinic)**
  - At time 0, just prior to administering the dose of study drug, collect a set of triplicate 12-lead ECGs, 2 minutes apart, and one blood sample for trough PK analysis
  - Administer study drug in clinic
  - At 2 and 4 hours postdose, collect a set of triplicate ECGs, 2 minutes apart, and one blood sample for PK analysis for each time point, respectively

Table 1: Schedule of Events

	Screening	Cycle 1, Day 1				Cycle 3, Day 1		
**Activities and Forms to be Completed Specific to the Subtudy Only:**	-	**Hour -1 pre-dose**	**Hour 0 pre-dose**	**2 Hours post-dose**	**4 Hours post-dose**	**Hour 0 pre-dose**	**2 Hours post-dose**	**4 Hours post-dose**
Informed consent	X							
Triplicate 12-lead ECG	X	X	X	X	X	X	X	X
PK samples*			X	X	X	X	X	X

*PK samples are to be collected immediately after each ECG. Sparse PK samples for the population PK analysis will not be collected for patients participating in this substudy.

Statistical Methods

A detailed Statistical Analysis Plan will be prepared prior to the planned analyses.

Two analyses will be performed: a primary analysis using the active treatment arm only, and a secondary analysis comparing the active and placebo arms, both accompanied by a pharmacokinetic/pharmacodynamics analysis.

Standard ECG parameters will be determined for each ECG recording. Corrected QTc intervals will be determined using Fridericia's formula (QTcF) and Bazett's formula (QTcB).

Changes in ECG intervals from baseline will be calculated. Baseline will be defined as the mean of the values for the triplicate ECG measurements taken pre-dose on Cycle 1 Day 1 (Hour -1 and Hour 0). In addition, QTcFs will be categorized based on ICH E14 guidelines. Tables will present the number and percentage of patients meeting or exceeding the following categories:

- QTc interval prolongation:
  - Absolute values > 450 to ≤ 480 msec
  - Absolute values > 480 to ≤ 500 msec
  - Absolute values > 500 msec
- QTc interval change from baseline:
  - Increase from baseline > 30 to ≤ 60 msec
  - Increase from baseline > 60 msec

Pharmacokinetic/Pharmacodynamic analyses will be performed using data from all subjects who have ECG data from at least one time point following the first dose. A linear modeling approach will be used to quantify the relationship (if any) between the plasma concentration of drug (apalutamide) and its principal metabolite (ARN000308), and the changes from baseline in the QTcF interval.

Sample Size Determination

A sample size of at least 100 patients will ensure that at least 60 patients treated with apalutamide will be enrolled on the substudy and 60 patients will provide at least 98.7% power to detect a true effect of 10 milliseconds (msec) change from baseline considering only the active group. Details for the power calculation and statistical assumptions are provided below.

Criteria for a "negative" result

It is assumed that the FDA criterion for regarding the outcome as negative (i.e., no QT prolongation concerns) will be that the mean QTcF increase observed in the active group should be significantly lower than a threshold of 20 msec at all on-study time points. "Significantly lower" means that the upper one-sided 95% confidence limit should be below the threshold.

For the primary analysis, the quantity of interest is the mean change from baseline (ΔQTcF). For the secondary analysis, the quantity of interest is the difference between the mean changes from baseline in the active and placebo groups (ΔΔQTcF), which should remove any fluctuations due to diurnal variation and/or changes due to time-on-study (e.g., disease progression).

Statistical Assumptions

[redacted]

[redacted]

APPENDIX 9: ANTICIPATED EVENTS

An anticipated event is an adverse event (serious or non-serious) that commonly occurs as a consequence of the underlying disease or condition under investigation (disease-related) or background regimen. For the purposes of this study, the following events will be considered anticipated events:

Disease-specific Events

Cauda equina syndrome
Erectile dysfunction
Haematospermia
Haematuria
Incontinence
Nocturia
Painful ejaculation
Pathologic fracture
Pollakiuria
Spinal cord compression
Ureteral obstruction
Urethral obstruction
Urinary flow decreased
Urinary retention
Urinary tract obstruction
Urinary hesitation
Lymphoedema
PSA Increased

ADT-Related Events

Depression
Gynaecomastia
Hot flush
Libido decreased
Osteoporosis
Sexual dysfunction
Testicular atrophy

Reporting of Anticipated Events

All adverse events will be recorded in the CRF regardless of whether they are considered to be anticipated events and will be reported to the sponsor as described in Section 9.2. Any anticipated event that meets serious adverse event criteria will be reported to the sponsor within the appropriate timeline as described in Section 9.2.3. These anticipated events are exempt from expedited reporting as individual single cases to Health Authorities. (Note: Japan will not identify anticipated events for the Health Authorities). However if based on an aggregate review, it is determined that an anticipated event is possibly related to study drug, the sponsor will report these events in an expedited manner.

Anticipated Event Review Committee (ARC)

An Anticipated Event Review Committee (ARC) will be established to perform reviews of pre-specified anticipated events at an aggregate level. The ARC is a safety committee within the sponsor's organization that is independent of the sponsor's study team. The ARC will meet to aid in the recommendation to the sponsor's study team as to whether there is a reasonable possibility that an anticipated event is related to the study drug.

Statistical Analysis

Details of statistical analysis of anticipated events, including the frequency of review and threshold to trigger an aggregate analysis of anticipated events will be provided in a separate Anticipated Events Safety Monitoring Plan (ASMP).

APPENDIX 10: CROSSOVER TO OPEN LABEL APALUTAMIDE AFTER STUDY UNBLINDING

At the time the decision is made to unblind the study, investigators will be notified by the sponsor. Subjects randomized to placebo who are currently on study treatment will be offered treatment with open-label apalutamide at 240 mg/day.

Subjects previously randomized to apalutamide will continue on protocol and follow the current schedule of activities (Appendix 1, with modifications below) and will be given open-label apalutamide.

For subjects who crossover from placebo to apalutamide, radiographic scans will no longer be required for the study and submitted for blinded central independent review. Subjects will be followed for progression per Investigator decision including symptoms, radiographic scans or PSA. PSA data submitted to the central laboratory will no longer be blinded. In addition, for all subjects, PK samples will no longer need to be collected at the time of unblinding.

Eligibility Criteria for Placebo Subjects to Crossover to Open Label Apalutamide

Subjects randomized to placebo must meet the criteria below to be eligible to crossover to open label apalutamide. The blood work for organ function criteria can be taken from the last study cycle evaluation prior to unblinding as long as not more than 2 months have elapsed between the last cycle evaluation and Cycle 1 Day 1 of the crossover phase.

1a. Subject is willing and able to provide written informed consent to crossover to open-label apalutamide

2a. Subject has adequate organ function as defined by the following criteria:

- Serum aspartate transaminase (AST; serum glutamic oxaloacetic transaminase [SGOT]) and serum alanine transaminase (ALT; serum glutamic pyruvic transaminase [SGPT]) ≤ 2.5 x upper limit of normal (ULN)
- Total serum bilirubin ≤1.5 x ULN. Total serum bilirubin >1.5 x ULN is allowed if Gilbert's disease has been previously documented
- Serum creatinine ≤ 2 x ULN
- Absolute neutrophil count (ANC) ≥ 1500/μL
- Platelets ≥ 100,000/μL
- Hemoglobin ≥ 9.0 g/dL

3a. Subject has not received any other systemic therapy for non-metastastic castrate resistant prostate cancer other than blinded study drug and androgen deprivation therapy.

4a. Subjects who have previously had an end of treatment visit and evidence of distant metastasis from the blinded independent central reviewer will not be eligible for open label apalutamide.

5a. Subjects who had study treatment withheld for ≥28 consecutive days at the time of unblinding will need approval from the medical monitor to be eligible for crossover.

Study Procedures for Subjects Previously on Placebo Who Crossover

Subjects will start open-label apalutamide with Cycle 1 and will be evaluated every cycle for 6 cycles, every other cycle for the next 6 cycles and then every 4 cycles thereafter. Refer to the Time and Events Schedule for placebo subjects who crossover to open-label apalutamide (Table 3). See Section 7 for the description of study assessments in Table 3.

Discontinuation Criteria for Subjects Who Crossover

Subjects who discontinue open label apalutamide will continue in Long-Term Follow-up per Section 8.9.

If a subject meets criteria as defined in Section 10 of the protocol, apalutamide must be discontinued.

Table 3 Time and Events Schedule for Subjects on Placebo Crossing Over to Open Label Apalutamide

	Screening	Crossover Phase			Posttreatment		
**Activities and Forms to be Completed**	**Visit from last treatment cycle on main study can be used**	**Cycle 1 Day 1**	**D1 C1-C6, D1 of every 2 cycles starting at C7 to C13, then D1 of every 4 cycles unless otherwise specified**	**Every 16 weeks**	**End-of-Treatment [1]**	**Safety Follow-up [2]**	**Long-term Follow-up [3]**
Informed Consent	X						
Eligibility Criteria	X						
**Study Drug Administration**							
Apalutamide [4]		X→	→				
Study Drug Compliance [5]			X		X		
**Laboratory Studies**							
Hematology [6]	X	X{6]	X		X		
Blood Chemistry [6]	X	X[6]	X		X		
PSA [7]	X	X[6]	X		X		
Testosterone [7]	X	X[7]		X	X		
Thyroid-Stimulating Hormone [7]	X	X[7]		X	X		
Fasting Lipid Panel [7]	X	X[7]		X	X		
**Efficacy**							
ECOG	X	X	X		X		
Medical Resource Utilization		X	X				X
Progression on first subsequent therapy [8]	X			X			X
Survival [3]							X
**Patient Reported Outcomes**							
FACT-P and EQ-5D[11]		X		X	X		X
**Safety**							
Physical Examination [9]	X	X	X		X		
Vital Signs [10]	X	X	X		X		
Adverse Events	Continuous until 28 days after the last dose of apalutamide						
Concomitant Medications	Continuous until 28 days after the last dose of apalutamide						

CT=computed tomography; ECOG=Eastern Cooperative Oncology Group; PSA=prostate specific antigen

Footnotes

**1.**	**End-of-Treatment:** These assessments do not need to be completed if they have been performed within 4 weeks of the last dose of study drug
**2.**	**Safety Follow-Up:** Patients should be evaluated for safety up to 28 days after the last dose of study drug. All AEs should be followed to their resolution, until the Investigator assesses them as stable, irreversible, or until the patient is lost to follow-up, whichever comes first. If a patient begins a new systemic anti-cancer therapy, the AE reporting period for non-SAEs ends at the time the new treatment is started.
**3.**	**Long-term Follow-up:** Obtain survival status, collect information of development of symptomatic progression, initiation of any new systemic anti-cancer therapies, and progression on first subsequent therapy (every 4 months via clinic visit, telephone contact or an alternative contact method per institution policy/practice).
**4.**	**Apalutamide:** Patients will receive open—label oral daily apalutamide continuously; a cycle consists of 28 days.
**5.**	**Study Drug Compliance:** Apalutamide bottle(s) including any unused tablets/capsules will be returned at the beginning of every cycle starting at Cycle 2 Day 1 and at end of treatment for drug accountability.
**6.**	**Samples for Hematology, Blood Chemistry:** All laboratory assessments will be performed by a central laboratory. On Cycle 1 Day 1 only, clinical laboratory tests do not need to be repeated if the Screening tests were done within 28 days of Cycle 1 Day 1. Sites may perform additional local hematology and/or blood chemistry assays for the purposes of planning treatment administration, dose modification, or monitoring adverse events.
**7.**	**Samples for PSA, TSH, fasting lipid panel, and testosterone:** These laboratory assessments will be performed by a central laboratory every 16 weeks. On Cycle 1 Day 1 only, clinical laboratory tests do not need to be repeated if the Screening tests were done within 28 days of Cycle 1 Day 1. Sites may perform additional local assays for the purposes of planning treatment administration, dose modification, or monitoring adverse events. See Appendix 2 for details on additional testing if TSH is abnormal.
**8.**	**Progression on First Subsequent Therapy:** The date of progression on first subsequent therapy as assessed by the investigator and method of assessment of disease progression (radiographic, PSA, or both) will be collected.
**9.**	**Physical Examination:** At Screening, a complete physical examination of major body systems. During subsequent visits, either a full or abbreviated physical exam will be performed. New abnormal physical exam findings must be documented and followed by a physician or other qualified staff at the next scheduled visit. Body weight will be recorded at Screening and at every scheduled visit during treatment and at the end of treatment.
**10.**	**Vital Signs:** Body temperature, blood pressure, pulse and respiratory rate will be performed after resting for 5 minutes at every scheduled visit during treatment and at the end of treatment.
**11.**	**FACT-P and EQ-5D questionnaires:** Patients will complete the FACT-P and EQ-5D at the clinic **PRIOR to any other clinical activity** on Cycle 1 Day 1, then Day 1 of every Cycle up to Cycle 13, then Day 1 of every other cycle, and at the End-of-Treatment Visit. During survival follow-up contact every 4 months via clinic visit or telephone up to 12 months post-progression

Statistical Analysis Plan (SAP)

Version 1.0

05 November 2012

SPARTAN

(Selective **P**rostate **AR T**argeting with **ARN-509)**

A Multicenter, Randomized, Double-Blind, Placebo-Controlled, Phase III Study of ARN-509 in Men with Non-Metastatic (M0) Castration-Resistant Prostate Cancer

Authors:

[redacted], PhD, Senior Director, Clinical Development

[redacted], PhD, Biostatistician Consultant to Aragon Pharmaceuticals

Aragon Pharmaceuticals - Confidential

STATISTICAL ANALYSIS PLAN APPROVAL

Protocol: ARN-509-003, Version 1.0 (05 November 2012)

Protocol Title: SPARTAN: A Multicenter, Randomized, Double-Blind, Placebo-Controlled, Phase III Study of ARN-509 in Men with Non-Metastatic (M0) Castration-Resistant Prostate Cancer

______________________________ ______________

[redacted], MD Date
Chief Medical Officer

TABLE OF CONTENTS

1. INTRODUCTION....4
2. STUDY DESIGN....4
2.1 STUDY OBJECTIVES....4
2.2 STUDY ENDPOINTS....5
3. RANDOMIZATION....6
4. SAMPLE SIZE DETERMINATION....6
5. INTERIM ANALYSIS....6
6. DATA MONITORING COMMITTEE....6
7. STATISTICAL METHODS....7
7.1 ANALYSIS POPULATIONS....7
7.2 DEFINITIONS....8
7.3 ANALYSIS OF STUDY CONDUCT....8
7.4 ANALYSIS OF TREATMENT GROUP COMPARABILITY....8
7.4.1 Demographics and Baseline Characteristics....9
7.4.2 Disease Characteristics and Prior Therapy....9
7.4.3 Study Drug Administration....9
7.4.4 Concomitant Medications and Treatments....10
7.4.5 Subsequent Anti-Cancer Therapy....10
7.5 EFFICACY ANALYSES....10
7.5.1 Blinded Independent Central Review (BICR)....10
7.5.2 Primary Endpoint (MFS)....10
7.5.3 Key Secondary Endpoint (OS)....12
7.5.4 Other Secondary Endpoints....12
7.6 SAFETY ANALYSES....15
7.6.1 Adverse Events....15
7.6.2 Laboratory Abnormalities....16
7.6.3 PSA....17
7.6.4 Vital Signs....17
7.7 HEALTH-RELATED QUALITY OF LIFE AND PROSTATE CANCER-SPECIFIC SYMPTOMS....18
7.8 POPULATION PHARMACOKINETIC ANALYSES....18
7.9 ASSESSMENT OF VENTRICULAR REPOLARIZATION....18
7.10 SENSITIVITY ANALYSES....19
7.10.1 PFS and MFS....19
7.10.2 BICR- and Investigator-Derived MFS....19
7.10.3 BICR- and Investigator-Derived PFS....19
7.10.4 BICR- and Investigator-Derived TTM....19
7.11 SUBGROUP ANALYSES OF COVARIATES....19
8. REFERENCES....21

1. INTRODUCTION

This document describes the planned statistical analyses for Protocol ARN-509-003 (Version 1.0, 05 November 2012): the **SPARTAN** Study **(**A Multicenter, Randomized, Double-Blind, Placebo-Controlled, Phase III Study of ARN-509 in Men with Non-Metastatic (M0) Castration-Resistant Prostate Cancer). This analysis plan is meant to supplement the study protocol. Any deviations from this analysis plan will be described in the clinical study report.

2. STUDY DESIGN

This is a randomized (2:1), multicenter, double-blind, placebo-controlled, Phase III clinical trial evaluating the efficacy and safety of ARN-509 (treatment arm A) versus placebo (treatment arm B) in approximately 1200 men with high risk non-metastatic (M0) castration-resistant prostate cancer (NM-CRPC), defined as PSA Doubling Time (PSADT) ≤ 10 months.

ARN-509 will be administered orally on a continuous daily dosing schedule, at a starting dose of 240 mg per day in treatment arm A. Matched placebo will be administered orally on a continuous daily dosing schedule, at a starting dose of 240 mg per day in treatment arm B.

Patients will be followed for safety and efficacy as per the schedule of assessments and will remain on study treatment until documented progression (development of metastases as assessed by blinded independent central review) or unacceptable toxicity.

Patients discontinuing treatment due to documented radiographic progression will enter the survival follow-up period, where they will be followed for the development of symptomatic progression and initiation of subsequent anti-cancer therapies (in particular, cytotoxic chemotherapy) every 4 months until death, loss of follow-up, or withdrawal of consent, whichever comes first.

Patients discontinuing treatment prior to documented radiographic progression will also enter the survival follow-up period where they will continue to have scheduled disease assessments every 4 months until documented radiographic progression, and will be followed for the development of symptomatic progression and initiation of subsequent anti-cancer therapies (in particular, cytotoxic chemotherapy) every 4 months until death, loss of follow-up, or withdrawal of consent, whichever comes first.

2.1 STUDY OBJECTIVES

Primary Objective

- To demonstrate superiority in the metastasis-free survival (MFS) of men with high risk NM-CRPC treated with ARN-509 versus placebo

Key Secondary Objective

- To compare the overall survival (OS) of men with high risk NM-CRPC treated with ARN-509 versus placebo

Other Secondary Objectives

- To compare the time to symptomatic progression in men with high risk NM-CRPC treated with ARN-509 versus placebo
- To compare the time to initiation of cytotoxic chemotherapy in men with high risk NM-CRPC treated with ARN-509 versus placebo
- To compare the radiographic progression-free survival (PFS) of men with high risk NM-CRPC treated with ARN-509 versus placebo
- To compare the time to metastasis (TTM) in men with high risk NM-CRPC treated with ARN-509 versus placebo
- To compare patient reported outcomes (PROs) of health-related quality of life and prostate cancer-specific symptoms in men with high risk NM-CRPC treated with ARN-509 versus placebo
- To evaluate the safety and tolerability of ARN-509
- To evaluate the population pharmacokinetics of ARN-509
- To evaluate the effect of ARN-509 on ventricular repolarization in a subset of patients from selected clinical sites [Appendix 8 of the protocol]

2.2 STUDY ENDPOINTS

Primary Endpoint

- Metastasis-Free Survival (MFS)

Key Secondary Endpoint

- Overall Survival (OS)

Other Secondary Endpoints

- Time to symptomatic progression
- Time to initiation of cytotoxic chemotherapy
- Radiographic Progression-Free Survival (PFS)
- Time to Metastasis (TTM)

Other Evaluations

- Health-related quality of life and prostate cancer-specific symptoms
- Type, incidence, severity, timing, seriousness, and relatedness of adverse events and laboratory abnormalities
- Population pharmacokinetics
- Assessment of ventricular repolarization [Appendix 8 of the protocol]

3. RANDOMIZATION

Upon verification of inclusion and exclusion criteria, eligible patients will be centrally randomized in a 2:1 ratio to either ARN-509 or placebo. The randomization will be stratified as follows:

- PSADT (> 6 months vs. ≤ 6 months)
- Bone-sparing agent use: Yes vs. No
- Loco-regional disease: N0 vs. N1

In order to ensure accurate and consistent determination of PSADT, the Interactive Voice Recognition System (IVRS) will also provide PSADT calculations (using a linear regression model of the natural logarithm of PSA and time) based on PSA values entered by the sites prior to randomization.[1]

4. SAMPLE SIZE DETERMINATION

The primary efficacy analysis will be event-driven and will take place when approximately [redacted] MFS events have occurred. The study provides 90% power to detect a [redacted] reduction in the risk of developing metastases (HR = [redacted]) for patients receiving ARN-509, with a two-sided α of 0.05.[2] Based on an assumed median MFS of 25 months in the placebo arm, this treatment effect represents an increase in the median MFS of approximately [redacted] months (from 25 months to [redacted] months). Assuming an accrual period of 24 months (with [redacted] of the patients accrued in the second year), approximately 1200 patients will need to be enrolled.

The study is also designed to provide 85% power to detect a [redacted] reduction (HR = [redacted]) in the risk of death for patients receiving ARN-509, based on an assumed median OS of [redacted] months in the placebo arm. This treatment effect represents an increase in the median OS of approximately [redacted] months (from [redacted] months). The final analysis of OS will occur after approximately [redacted] deaths have occurred.

The total study duration (including the time it takes to reach the secondary endpoint of OS) will be approximately 59 months.

5. INTERIM ANALYSIS

There will be no interim analysis of the primary endpoint (MFS).

A single interim efficacy analysis of the key secondary endpoint (OS) will be carried out at the time of the efficacy analysis for MFS. At that time, approximately [redacted] OS events will be observed, representing [redacted] of the total number of events [redacted] at the final OS analysis. Based on this information fraction [redacted]) and using the Lan-DeMets method for group sequential trials with O'Brien-Fleming boundaries, the levels of significance for the interim and final analyses of OS will be [redacted] and [redacted], respectively.[3]

6. DATA MONITORING COMMITTEE

An independent third-party Data Monitoring Committee (DMC) will be established to ensure the overall integrity and conduct of the study.

The DMC will review the progress of the study and cumulative unblinded safety data on a periodic basis (e.g., a minimum of two face to face review meetings per year) as well as serve as the primary reviewers of the efficacy analysis. In addition to the formal face to face meetings, unblinded listings of serious adverse events will be provided to the DMC on a monthly basis.

Following each review meeting, the DMC will recommend to the Sponsor whether to continue the trial unchanged, modify the conduct of the study, or terminate the study early. Rules for early termination, modification and/or continuation of the study, as well as how these recommendations will be made to the Sponsor and Health Authorities will be outlined in a separate DMC Charter.

The DMC will be composed of 3 external members [2 physicians and 1 biostatistician] not associated with the conduct of the study. The Sponsor will also designate an independent biostatistician not affiliated with the project to prepare and provide study data to the DMC. Complete details regarding the composition and governance of the DMC will be outlined in the DMC Charter.

Periodic adverse event data review will also be performed by designated members of the Sponsor's primary study team and will be blinded to treatment assignment with adverse event data from both treatment groups combined. Any safety issues of concern identified by the primary study team that require notification of the DMC will be communicated as described in the DMC Charter.

7. STATISTICAL METHODS

The primary objective of the study is to evaluate the efficacy of ARN-509 compared to placebo in patients with high risk NM-CRPC as measured by metastasis-free survival (MFS), based on blinded independent central review (BICR) of tumor assessments.

7.1 ANALYSIS POPULATIONS

Full Analysis (Intent-to-Treat) Population [ITT]: All eligible patients who are randomized into the study, with study drug assignments designated according to initial randomization, regardless of whether patients receive study drug or receive a different drug from that to which they were randomized to will be included in the analyses of all efficacy and clinical benefit endpoints and patient characteristics.

Safety Analysis Population [SAFETY]: All patients who receive at least one dose of study drug, with treatment assignments designated according to actual study treatment received will be the primary population for evaluating safety and treatment compliance and administration.

- **Patient Report Outcomes Population [PRO]**: Subset of the safety analysis population that has completed at least the baseline assessment (Cycle 1 Day 1) of either FACT-P or EQ-5D questionnaires.
- **Population Pharmacokinetics Populations [PK]**: Subset of the safety analysis population that was randomized to the ARN-509 treatment arm and that has at least one PK sample collected.

7.2 DEFINITIONS

Study Day: Study day will be calculated in reference to the date of randomization. Study Day 1 corresponds to the date the patient was randomized into the study.

Baseline Value: Unless otherwise specified, the baseline value will be defined as the closest measurement prior to the first dose of study drug. Change from baseline will be defined as (post-baseline value – baseline value).

Treatment Duration: Treatment duration will be defined as the duration of time from the date of the first dose of study drug to the date of last dose of study drug + 1 day.

Time to event: Time to event calculations will be defined as the time from randomization to date of event + 1 day. Time to event or duration of event endpoints will be based on the actual date of the event, not visit number or visit label.

Survival Follow-Up Phase: The survival follow-up phase will start from the safety follow-up visit (28 days following the last dose of study drug) and continue through the end of the study.

7.3 ANALYSIS OF STUDY CONDUCT

All patients randomized into the study will be summarized by treatment arm as randomized. The randomization stratification factors will be listed and tabulated as documented on the case report form (CRF) and as recorded in the IVRS by treatment arm as randomized. Discrepancies between the CRF and the IVRS will be identified and summarized.

The number of patients who are in the ITT and SAFETY populations and the reason for exclusion from the SAFETY population will be summarized by treatment arm as randomized and overall.

Study treatment administration, duration of follow-up, discontinuation from study treatment and the reasons for discontinuation will be summarized by treatment arm for all randomized patients. In addition, protocol deviations and eligibility violations will also be summarized by treatment arm. Protocol violations for this study include, but are not limited to, the following:

- Failure to meet inclusion/exclusion criteria
- Use of a prohibited concomitant medication
- Dose modifications that are not within the protocol specifications
- Any other deviation that presents significant risk or safety concerns to the patient

7.4 ANALYSIS OF TREATMENT GROUP COMPARABILITY

The evaluation of treatment group comparability between the 2 treatment arms will include summary of demographics, baseline disease characteristics, medical history, and patient treatment history.

Descriptive statistics (mean, standard deviation, median, range) will be presented by treatment arm for continuous variables such as age and time from initial diagnosis. Categorical variables will be summarized using frequencies and percentages.

7.4.1 Demographics and Baseline Characteristics

The following parameters will be summarized overall by treatment arm as randomized for all patients in the ITT population:

- Age, race, and baseline weight
- Baseline ECOG performance status
- Baseline PSA value and PSA Doubling Time (PSADT)

The above parameters will also be summarized by geographic region: North America (NA), Europe (EU) and Rest of World (ROW).

7.4.2 Disease Characteristics and Prior Therapy

The following parameters will be summarized overall by treatment arm as randomized for all patients in the ITT population:

- Time (months) from initial diagnosis of prostate cancer to randomization
- Total Gleason Score at initial diagnosis
- Clinical tumor stage (T) and pathologic tumor stage (pT) at initial diagnosis
- Regional lymph nodes at initial diagnosis: clinical stage (N) and pathologic stage (pN)
- Number of prior hormonal therapies
- Use of bone-sparing agent (Yes/No) at baseline
- History of surgical prostate cancer procedures (Yes/No)
- Type of surgical procedures (prostatectomy, orchiectomy, transurethral resection of the prostate (TURP), and other)
- History of radiotherapy (Yes/No)
- Type of radiotherapy (external beam only, brachytherapy only, and both)
- History of prior adjuvant/neoadjuvant chemotherapy (Yes/No)

The above parameters will also be summarized by geographic region: North America (NA), Europe (EU) and Rest of World (ROW).

7.4.3 Study Drug Administration

The SAFETY population will be used to summarize drug exposure, treatment compliance, and dose modifications by treatment arm as treated.

Treatment duration will be defined as the duration of time from the date of the first dose of study drug to the date of last dose of study drug + 1 day. The number of capsules taken will be calculated based on the number of capsules dispensed at the study visits minus the number of capsules indicated as having been returned. The total cumulative dose in milligrams (mg) will be calculated as 30 mg multiplied by the number of capsules taken.

The percent overall treatment compliance will be defined as the number of capsules taken during the study divided by the expected number of capsules, multiplied by 100. Each patient should be taking 8 capsules per day maximum while on the study. A patient's expected number of capsules will be calculated as the number of assigned capsules per day multiplied by treatment duration.

For patients with dose reductions, the expected number of capsules will be reflective of the new dose with a reduced number of total capsules.

Patients with at least one dose modification and the reason for the dose modification will be summarized by treatment arm as treated.

7.4.4 Concomitant Medications and Treatments

Concomitant medications and treatments taken prior to starting study treatment and those administered during the study will be summarized for all patients in the SAFETY population by treatment arm as treated. Medications are considered concomitant if taken during the treatment-emergent period. Medications will be summarized by WHO Drug therapeutic class and generic medication name.

7.4.5 Subsequent Anti-Cancer Therapy

New systemic anti-cancer therapy taken after the patient has discontinued study drug will be summarized by treatment arm and overall. Medications will be summarized by WHO Drug therapeutic class and generic medication name.

7.5 EFFICACY ANALYSES

The following section outlines the planned analyses of the primary and secondary efficacy outcomes of the study.

Efficacy analyses will be performed on the ITT population, incorporating the randomization stratification factors as documented on the CRF, unless otherwise specified.

Time-to-event endpoints will be summarized using the Kaplan-Meier method and displayed graphically where appropriate. Median event times and 2-sided 95% confidence interval for each median will be provided.

7.5.1 Blinded Independent Central Review (BICR)

Analyses of efficacy endpoints which are based on radiographic tumor assessments (MFS, TTM, and PFS) will be based on the results of the blinded independent central review (BICR), provided via electronic data transfer by the third-party core imaging laboratory.

All scans will be submitted for independent review of disease progression during the study according to an Independent Review Charter to be prepared by the core imaging laboratory in consultation with the Sponsor.

7.5.2 Primary Endpoint (MFS)

The primary efficacy endpoint is metastasis-free survival (MFS), defined as the time from randomization to first evidence of BICR-confirmed radiographically detectable bone or soft

tissue distant metastasis (simply referred to as "metastasis" from this point forward) or death due to any cause (whichever occurs earlier) + 1 day.

MFS data for patients without metastasis or death will be censored on the date of the last tumor assessment (or, if no tumor assessment was performed after the baseline visit, at the date of randomization + 1 day).

Additional censoring rules will vary according to whether the analysis is performed for US or ex-US regulatory purposes, as follows; both results will be provided in the clinical study report.

Scenario	US regulatory guidance	ex-US regulatory guidance[4]
Data from patients who are lost to follow-up or whose disease progression (development of metastasis) or death occurs after 2 or more consecutively missing or unevaluable tumor assessments	Censored on the date of the last tumor assessment that the patient was known to be metastasis-free	Time of progression will be determined using the first date when there is documented evidence of progression or death (whichever occurs earlier) regardless of missed or unevaluable tumor assessments
Patients that receive new systemic anti-cancer therapy prior to documented disease progression (development of metastasis) or death	Censored on the date of the last tumor assessment prior to the start of the new systemic anti-cancer therapy	Time of progression will be determined using the first date when there is documented evidence of progression or death (whichever occurs earlier) regardless of change of therapy

Disease progression (development of metastasis) will be assessed by an independent core imaging laboratory using the Response Evaluation Criteria in Solid Tumors (RECIST v1.1).[5] The appearance of new (or distant) metastatic lesions denotes disease progression. For new bone lesions detected on bone scans, a second imaging modality (e.g., CT or MRI) will be required to confirm progression.

The primary efficacy analysis will be completed when approximately [redacted] MFS events have occurred. The primary analysis will compare the MFS distributions in the two treatment arms using a two-sided log-rank test, stratified by PSADT (> 6 months vs. ≤ 6 months), the use of a bone-sparing agent (Yes vs. No), and the presence of loco-regional disease (N0 vs. N1) at the 0.05 significance level. The unstratified log-rank test will be provided as a sensitivity analysis. Additional sensitivity analyses are provided in Section 7.10.

Kaplan-Meier methods will be used to estimate median MFS for each treatment arm.[6] Cox proportional-hazard models, including the same factors as above, will be used to estimate the hazard ratio and its 95% confidence interval (CI).

7.5.3 Key Secondary Endpoint (OS)

Overall survival (OS) is the key secondary endpoint. In order to control the overall level of significance, the analysis of OS will be carried out only if the analysis of MFS is statistically significant.[7]

OS will be defined as the time from randomization to the date of death due to any cause + 1 day. Patients who are alive at the time of the analysis will be censored on the last known date that they were alive. In addition, the following censoring rules will apply:

Scenario	Date of Censoring
Patients with no post-baseline information	Censored on the date of randomization + 1 day
Patients who are lost to follow-up or who withdraw consent for further follow-up	Censored on the last known date that they were alive
Sensitivity Analysis: Patients that receive new systemic anti-cancer therapy	*Censored on the day before the start date of the new systemic anti-cancer therapy*

Data analysis will be similar to that for the primary endpoint (stratified two-sided log-rank test). The unstratified log-rank test will be provided as a sensitivity analysis.

The final analysis of OS will occur after approximately [redacted] deaths have occurred. In order to adjust for a single interim analysis of OS, which will occur at the time of the analysis for MFS, the levels of significance for the interim and final analyses of OS will be [redacted] and [redacted], respectively.

As additional analyses, the 1- and 2-year survival rates will be estimated using the Kaplan-Meier method.

7.5.4 Other Secondary Endpoints

Following the primary analysis of MFS (and interim analysis of OS), the analyses of the other secondary endpoints will be prioritized in the order listed below such that if one of the endpoints does not reach statistical significance (two-sided $p < 0.05$), the results for all other secondary endpoints of a lower rank will be considered to be exploratory:

[redacted]

Time-to-event-based secondary analyses [redacted] will be performed using a two-sided log-rank test, stratified by PSADT (> 6 months vs. ≤ 6 months), the use of a bone-sparing agent (Yes vs. No), and the presence of loco-regional

disease (N0 vs. N1) at the 0.05 significance level. Unstratified log-rank tests will also be provided as sensitivity analyses.

Kaplan-Meier methods will be used to estimate medians for each treatment arm. Cox proportional-hazard models, including the same factors as above, will be used to estimate the hazard ratio and its 95% confidence interval (CI).

7.5.4.1 Time to Symptomatic Progression

Time to symptomatic progression will be defined as the time from randomization to documentation in the CRF of any of the following (whichever occurs earlier) + 1 day:

- Development of a skeletal-related event (SRE): pathologic fracture, spinal cord compression, or need for surgical intervention or radiation therapy to the bone.
- Pain progression or worsening of disease-related symptoms requiring initiation of a new systemic anti-cancer therapy.
- Development of clinically significant symptoms due to loco-regional tumor progression requiring surgical intervention or radiation therapy.

Adverse event, concomitant medication, concomitant treatment, or survival follow-up CRFs will be the source of these findings.

Time to symptomatic progression for patients who do not experience any of the events described above will be censored on the date on which they were last known to be event-free.

7.5.4.2 Time to Initiation of Cytotoxic Chemotherapy

Time to initiation of cytotoxic chemotherapy will be defined as the time from randomization to documentation of a new cytotoxic chemotherapy being administered to the patient (e.g., survival follow-up CRF) + 1 day.

Time to initiation of cytotoxic chemotherapy for patients who do not start a cytotoxic chemotherapy will be censored on the date of last contact.

7.5.4.3 Radiographic Progression-Free Survival

In order to capture loco-regional disease progression, a secondary endpoint of progression-free survival (PFS) will be assessed and defined as the time from randomization to first documentation of BICR-confirmed radiographic progressive disease or death due to any cause (whichever occurs first) + 1 day.

Progressive disease (PD) will based on RECIST v1.1, and further defined as follows:

- For patients with at least one measurable lesion, PD will be defined as at least a 20% increase in the sum of diameters of target lesions taking as reference the smallest sum on study (this includes the baseline sum if that is the smallest on study). In addition to the relative increase of 20%, the sum must also demonstrate an absolute increase of at least 5 mm. Furthermore, the appearance of one or more new lesions is also considered progression.
- For patients with only non-measurable disease observed on CT or MRI scans, unequivocal progression (representative of overall disease status change) or the

appearance of one or more new lesions will be considered progression. For new bone lesions detected on bone scans, a second imaging modality (e.g., CT or MRI) will be required to confirm progression.

Radiographic PFS data for patients without loco-regional disease will be censored on the date of the last tumor assessment (or, if no tumor assessment was performed after the baseline visit, at the date of randomization + 1 day). Additional censoring rules will vary according to whether the analysis is performed for US or ex-US regulatory purposes, as shown below; both results will be provided in the clinical study report.

**Scenario**	**US regulatory guidance**	**ex-US regulatory guidance**
Data from patients who are lost to follow-up or whose disease progression or death occurs after 2 or more consecutively missing or unevaluable tumor assessments	Censored on the date of the last tumor assessment that the patient was known to be progression-free	Time of progression will be determined using the first date when there is documented evidence of progression or death (whichever occurs earlier) regardless of missed or unevaluable tumor assessments
Patients that receive new systemic anti-cancer therapy prior to documented disease progression or death	Censored on the date of the last tumor assessment prior to the start of the new systemic anti-cancer therapy	Time of progression will be determined using the first date when there is documented evidence of progression or death (whichever occurs earlier) regardless of change of therapy

7.5.4.4 Time to Metastasis

Time to Metastasis (TTM) will be defined as the time from randomization to first evidence of BICR-confirmed radiographically detectable bone or metastasis + 1 day.

TTM data for patients without metastasis will be censored on the date of the last tumor assessment (or, if no tumor assessment was performed after the baseline visit, at the date of randomization + 1 day). Additional censoring rules will vary according to whether the analysis is performed for US or ex-US regulatory purposes, as shown below; both results will be provided in the clinical study report.

Scenario	US regulatory guidance	ex-US regulatory guidance
Data from patients who are lost to follow-up or whose disease progression (development of metastasis) occurs after 2 or more consecutively missing or unevaluable tumor assessments	Censored on the date of the last tumor assessment that the patient was known to be metastasis-free	Time of progression will be determined using the first date when there is documented evidence of progression regardless of missed or unevaluable tumor assessments
Patients that receive new systemic anti-cancer therapy prior to documented disease progression (development of metastasis)	Censored on the date of the last tumor assessment prior to the start of the new systemic anti-cancer therapy	Time of progression will be determined using the first date when there is documented evidence of progression regardless of change of therapy

7.6 SAFETY ANALYSES

7.6.1 Adverse Events

Patients will be assessed for adverse events at each monthly clinic visit while on the study. Adverse events (AEs) will be graded according to the NCI Common Terminology Criteria for Adverse Events (CTCAE) Version 4.0 and coded to preferred term and system organ class (SOC) using the most recent version of MedDRA.

All AEs reported during the AE reporting period (inclusive of the 28-day post last dose of study drug period) will be considered as treatment-emergent adverse events and will be summarized by treatment arm as treated using all patients in the SAFETY population.

For each treatment arm, adverse event incidence rates will be summarized with frequency and percentage by MedDRA SOC and preferred term, with all patients treated in that treatment arm as the denominator, unless otherwise specified. In addition, AE incidence rates will also be summarized by severity and relationship to study drug. Treatment-related AEs are those judged by the Investigator to be at least possibly related to the blinded study drug. AEs with missing severity or relationship to study drug will be classified as severe and treatment-related, respectively. Patients with multiple occurrences of events will only be counted once at the maximum severity to study drug for each preferred team, SOC, and overall. Deaths that occur within 28 days after the last dose of study drug are defined as on-study deaths.

Summary tables of the following AEs will be provided:

- Overall summary of AEs: the number and percentage of patients who experienced any AE, any serious adverse event (SAE), any treatment-related AE, any treatment-related SAE, any discontinuations due to an AE, and any deaths
- All AEs by SOC and preferred term

- All AEs by SOC, preferred term, and maximum severity
- All AEs by decreasing frequency of preferred term
- Grades 3 or 4 AEs by SOC and preferred term
- Grades 3 or 4 AEs by decreasing frequency of preferred term
- Treatment-related AEs by SOC and preferred term
- Treatment-related AEs by SOC, preferred term, and maximum severity
- Treatment-related AEs by decreasing frequency of preferred term
- Treatment-related Grades 3 or 4 AEs by SOC and preferred term
- Treatment-related Grades 3 or 4 AEs by decreasing frequency of preferred term
- AEs that led to study drug discontinuation by SOC and preferred term. Study drug discontinuation will be determined from the End of Treatment CRF (where reason for termination is "Adverse Event") and the specific AE will be determined from the AE CRF page (where action taken is "Withdrawn from Study")
- AEs that led to study drug discontinuation by SOC, preferred term, and maximum severity
- All SAEs by SOC and preferred term
- All SAEs by SOC, preferred term, and maximum severity
- Deaths will be summarized by time period (on-study vs. during follow-up) and cause of death.

Patient listings of all Grades 3 or 4 AEs, all SAEs, AEs that led to study drug discontinuation and all deaths will be provided as well.

Narratives will be written for the following patients in the final clinical study report:

- Patients who die due to treatment-related adverse events 28 or more days after the last dose of study drug
- Patients who discontinue study drug due to adverse events
- Patients who have a treatment-related serious adverse event
- Patients who experience a seizure
- Patients who develop secondary malignancies

7.6.2 Laboratory Abnormalities

Only data collected by the central laboratory will be summarized. Local laboratory data collected for the purposes of planning treatment administration, dose modification, or monitoring adverse events, will not be summarized.

Normal ranges will be used to identify values that are outside the normal ranges and abnormal laboratory results will be graded according to the NCI CTCAE Version 4.0.

Descriptive statistics will be provided for each test result and for the change from baseline by visit.

A shift summary of baseline grade by maximum post-baseline CTCAE grade will be presented, as appropriate. For each laboratory parameter, the baseline laboratory value will be defined as the last laboratory value collected on or prior to the date of the first dose of study drug.

Patients who develop toxicities of Grade ≥ 3 will be summarized. Laboratory test results not having CTCAE grade will also be summarized. Parameters that have criteria available for both low and high values (e.g., hypercalcaemia vs. hypocalcaemia) will be summarized for both criteria. Patients will only be counted once for each criterion.

7.6.3 PSA

PSA kinetics (e.g., PSA response and time to PSA progression) will be assessed at the time of the primary analysis of MFS according to the Prostate Cancer Clinical Trials Working Group (PCWG2) recommendations.[10]

PSA laboratory results will be kept blinded and not released until the time of the primary analysis of MFS. Summary tables and waterfall plots describing change in PSA relative to baseline will be reported at 12 weeks (or earlier for those who discontinue study treatment prior to 12 weeks), and separately, the maximum change at any time on study will also be reported for each patient using summary tables and waterfall plots.

The time to PSA progression will be calculated as the time from randomization to the time when the criteria for PSA progression according to PCWG2 are met + 1 day. Kaplan-Meier methods will be used to estimate the median time to PSA progression and 95% confidence intervals for each treatment arm.

7.6.4 Vital Signs

Each vital sign (temperature, blood pressure (systolic and diastolic), respiration rate, and heart rate) and respective change from baseline will be summarized and presented by treatment arm and study visit. Patients with clinically significant abnormalities in vital signs as compared to baseline will be listed.

Data will be summarized and presented according to the following categories:

Parameter	Criteria for Clinically Significant Abnormality
Systolic Blood Pressure	Absolute result > 180 mmHg and increase from baseline > 40 mmHg
	Absolute result < 90 mmHg and decrease from baseline > 30 mmHg
Diastolic Blood Pressure	Absolute result > 105 mmHg and increase from baseline > 30 mmHg
	Absolute result < 50 mmHg and decrease from baseline > 20 mmHg
Heart Rate	Absolute result > 120 bpm and increase from baseline > 30 bpm
	Absolute result < 50 bpm and decrease from baseline > 20 bpm

7.7 HEALTH-RELATED QUALITY OF LIFE AND PROSTATE CANCER-SPECIFIC SYMPTOMS

The FACT-P and EQ-5D data will be scored and handled as recommended in their respective User's manuals, including handling of missing data both within the subscales and overall.[8,9] All FACT-P and EQ-5D data analyses will be performed in the PRO population. On-study scores and change from baseline scores will be summarized and displayed graphically as appropriate.

Health-related quality of life response will be defined as a 16-point or higher improvement in the global FACT-P score as compared to baseline. The proportions of patients with a quality of life response will be summarized by treatment arm. The response rates between treatment arms will be compared using a Mantel-Haenszel test, stratified by PSADT (> 6 months vs. ≤ 6 months), the use of a bone-sparing agent (Yes vs. No), and the presence of loco-regional disease (N0 vs. N1) at a two-sided 0.05 significance level.

The EQ-5D data will be summarized descriptively by treatment group and study visit.

7.8 POPULATION PHARMACOKINETIC ANALYSES

Blood for determination of plasma concentrations of ARN-509 and its metabolites ARN000308 and ARN000066 will be collected at various times post-dose from all patients on Day 1 of Cycles 1, 2, 3, 6, 12, 18, 24, 36, yearly thereafter, and at the end of treatment.

Population PK analysis will utilize patient covariates to identify sub-populations where possible. The relationship of exposure to ARN-509 and its metabolites (ARN000308 and ARN000066) to measures of efficacy and adverse events will also be modeled to the extent possible. The PK population will be the primary population used for these analyses.

Descriptive statistics, including mean and median values will be summarized. Concentrations below the lower limit of quantification of the assay will be excluded or assigned a numeric value based on the lower reporting limit of the assay. Plasma levels of ARN-509 and its metabolites ARN000308 and ARN000066 will be listed by patient and summarized descriptively (mean, standard deviation, percent coefficient of variation, minimum, maximum). Individual and mean concentration versus time plots will be presented on both linear and logarithmic scales where possible. Non-compartmental, compartmental, and population analysis methods will be utilized as applicable.

Samples may also be used for metabolite identification, but the results of such analyses will be used for exploratory purposes and will not be included in the clinical study report. Selected samples may also be analyzed for concentrations of concurrent medications, and these data may be used to assess drug-drug interactions. Blood samples for PK will be collected from all patients due to the blinded nature of the study, but samples collected from placebo patients will not be analyzed in most cases.

7.9 ASSESSMENT OF VENTRICULAR REPOLARIZATION

The assessment of ventricular repolarization will be a sub-study conducted in a subset of patients from selected clinical sites and analyzed by an independent cardiac safety laboratory. Description of the sub-study is provided in Appendix 8 of the study protocol.

7.10 SENSITIVITY ANALYSES

The main efficacy analyses include secondary analyses that take into consideration potential bias introduced by missed visits and the use of new systemic anti-cancer therapy. In addition to unstratified log-rank tests, other sensitivity analyses will be conducted to account for the potential impact of loco-regional disease progression on the primary endpoint of MFS as well as the potential impact of differences between BICR- and investigator-derived endpoints.

7.10.1 PFS and MFS

A sensitivity analysis of the primary endpoint will be performed where MFS, based on BICR-derived disease progression will be defined as the time from randomization to documented disease progression, either loco-regional tumor progression (BICR-derived PFS) or distant metastasis (BICR-derived MFS), or death at any time (whichever occurs earlier) + 1 day. Patients without a MFS or PFS event will be censored on the last BICR assessment date.

7.10.2 BICR- and Investigator-Derived MFS

A sensitivity analysis will be performed on the primary endpoint of MFS to compare BICR- versus investigator-derived progression (development of metastasis). Patients without a MFS event will be censored on the last BICR or investigator tumor assessment date.

7.10.3 BICR- and Investigator-Derived PFS

A sensitivity analysis will be performed on the secondary endpoint of PFS to compare BICR- versus investigator-derived progression (radiographic loco-regional progressive disease). Patients without a PFS event will be censored on the last BICR or investigator tumor assessment date.

7.10.4 BICR- and Investigator-Derived TTM

A sensitivity analysis will be performed on the secondary endpoint of TTM to compare BICR- versus investigator-derived progression (development of metastasis). Patients without a TTM event will be censored on the last BICR or investigator tumor assessment date.

7.11 SUBGROUP ANALYSES OF COVARIATES

In order to assess the consistency of treatment benefit with respect to the primary efficacy endpoint of MFS and secondary endpoints of OS across important subgroups, forest plots will be provided for the following variables:

- ECOG performance status (0 vs. 1) at baseline
- Age category (< 65 vs. ≥ 65 years **and** < 75 vs. ≥ 75 years)
- Race/ethnicity (white, black, Asian, and others)
- Geographic region (NA, EU, and ROW)
- Number of prior hormonal therapies (1 vs. ≥ 2)
- Baseline PSA value (at or below median vs. above median)
- PSADT (> 6 months vs. ≤ 6 months)

- Bone-sparing agent use (Yes vs. No)
- Loco-regional disease (N0 vs. N1)

The comparison between treatment arms will be evaluated by a single hazard ratio with its 95% CI based on an unstratified Cox regression model for each subgroup.

8. REFERENCES

1. Pound CR, Partin AW, Eisenberger MA, et al. Natural history of progression after PSA elevation following radical prostatectomy. JAMA 1999; 281:1591-7
2. Collett D. Modeling Survival Data in Medical Research. Boca Raton: Chapman and Hall/CRC, 1994, pp 254-5
3. Reboussin DM, DeMets DL, Kim KM, and Lan KKG. Computations for group sequential boundaries using the Lan-DeMets spending function method. Controlled Clinical Trials 2000; 21:190-207
4. Appendix 1 to the guideline on the evaluation of anticancer medicinal products in man: Methodological consideration for using progression-free survival (PFS) or disease-free survival (DFS) in confirmatory trials. European Medicines Agency 2011; Doc. Ref. EMA/CHMP/27994/2008
5. Eisenhauer EA, Therasse P, Bogaerts J, et al. New response evaluation criteria in solid tumours: Revised RECIST guideline (version 1.1). Eur J Cancer 2009; 45:228-47
6. Kaplan EL and Meier P. Nonparametric estimation from incomplete observations. J Am Stat Assoc 1958; 53:457-81
7. Westfall PH and Krishen A. Optimally weighted, fixed sequence and gatekeeper multiple testing procedures. Journal of Statistical Planning and Inference 2001; 99:25-40
8. Esper P, Mo F, Chodak G, et al. Measuring quality of life in men with prostate cancer using the Functional Assessment of Cancer Therapy-Prostate (FACT-P) instrument. Urology 1997; 50:920-8
9. The EuroQol Group. EuroQol – a new facility for the measurement of health-related qualify of life. Health Policy 1990; 16:199-208
10. Scher HI, Halabi S, Tannock I, et al. Design and end points of clinical trials for patients with progressive prostate cancer and castrate levels of testosterone: Recommendations of the Prostate Cancer Clinical Trials Working Group. J Clin Oncol 2008; 26:1148-59

Statistical Analysis Plan (SAP)

Version 7.0

26 June 2017

SPARTAN

(Selective **P**rostate **AR T**argeting with **ARN-509)**

A Multicenter, Randomized, Double-Blind, Placebo-Controlled, Phase III Study of ARN-509 in Men with Non-Metastatic (M0) Castration-Resistant Prostate Cancer

ARN-509-003; Phase 3

Sponsor: Aragon Pharmaceuticals, Inc*

*Aragon Pharmaceuticals, Inc. is a wholly-owned subsidiary of Johnson & Johnson. Janssen Research & Development, LLC is part of the Janssen Pharmaceutical Companies of Johnson & Johnson and provides various services to its affiliated company, Aragon Pharmaceuticals, Inc.

Status: Approved
Date: 26 June 2017
Prepared by: Janssen Research & Development, LLC
Document No.: EDMS-ERI-80096900; 7.0

Compliance: The study described in this report was performed according to the principles of Good Clinical Practice (GCP).

Confidentiality Statement
The information in this document contains trade secrets and commercial information that are privileged or confidential and may not be disclosed unless such disclosure is required by applicable law or regulations. In any event, persons to whom the information is disclosed must be informed that the information is privileged or confidential and may not be further disclosed by them. These restrictions on disclosure will apply equally to all future information supplied to you that is indicated as privileged or confidential.

TABLE OF CONTENTS

1. INTRODUCTION.....4
2. STUDY DESIGN.....4
2.1 STUDY OBJECTIVES.....4
2.2 STUDY ENDPOINTS.....5
3. RANDOMIZATION.....6
4. SAMPLE SIZE DETERMINATION.....6
5. INTERIM ANALYSIS.....6
6. INDEPENDENT DATA MONITORING COMMITTEE.....6
7. STATISTICAL METHODS.....7
7.1 ANALYSIS POPULATIONS.....7
7.2 DEFINITIONS.....7
7.3 ANALYSIS OF STUDY CONDUCT.....8
7.4 ANALYSIS OF TREATMENT GROUP COMPARABILITY.....8
7.4.1 Demographics and Baseline Characteristics.....8
7.4.2 Disease Characteristics and Prior Therapy.....9
7.4.3 Study Drug Administration.....9
7.4.4 Pre-study and Concomitant Medications.....10
7.4.5 Subsequent Anti-Cancer Therapy.....10
7.5 EFFICACY ANALYSES.....10
7.5.1 Blinded Independent Central Review (BICR).....10
7.5.2 Primary Endpoint (MFS).....10
7.5.3 Secondary Endpoints.....11
7.6 SAFETY ANALYSES.....16
7.6.1 Adverse Events.....16
7.6.2 Laboratory Abnormalities.....17
7.6.3 Vital Signs.....18
7.7 OTHER EVALUATIONS.....18
7.7.1 Second Progression-Free Survival (PFS2).....18
7.7.2 PSA.....18
7.7.3 Health-Related Quality of Life and Prostate Cancer-Specific Symptoms.....19
7.7.4 Population Pharmacokinetic Analyses.....19
7.7.5 Exploratory Biomarker Analyses.....19
7.7.6 Assessment of Ventricular Repolarization.....19
7.7.7 Medical Resource Utilization Analysis.....19
7.8 SENSITIVITY ANALYSES.....20
7.8.1 Investigator-Derived MFS.....20
7.8.2 OS.....20
7.8.3 Subgroup Analyses.....21

7.9 ANALYSIS BY FORMULATION 21
7.9.1 Subject Distribution by Formulation 21
7.9.2 Demographics and Baseline characteristics by Formulation 21
7.9.3 Study Drug Administration by Formulation 22
7.9.4 Safety Analyses by Formulation 22
7.9.5 Efficacy (MFS and PSA Response) Analyses by Formulation 22
8. REFERENCES 23
9. APPENDICES 25
9.1 APPENDIX 1: OUTLINE OF THE ADAPTIVE GROUP SEQUENTIAL TESTING OF [redacted] 25
9.2 APPENDIX 2: TABLE SHELL FOR OVERALL SUMMARY OF ADVERSE EVENTS BY FORMULATION 28
9.3 APPENDIX 3: MODIFIED MEDDRA QUERIES AS SEARCH CRITERIA FOR AE OF SPECIAL INTEREST 29

1. INTRODUCTION

This document describes the planned statistical analyses for Protocol ARN-509-003 the **SPARTAN** Study **(**A Multicenter, Randomized, Double-Blind, Placebo-Controlled, Phase III Study of ARN-509 in Men with Non-Metastatic (M0) Castration-Resistant Prostate Cancer). This analysis plan is meant to supplement the study protocol. Any deviations from this analysis plan will be described in the clinical study report.

2. STUDY DESIGN

This is a randomized (2:1), multicenter, double-blind, placebo-controlled, Phase III clinical trial evaluating the efficacy and safety of ARN-509 versus placebo in approximately 1200 men with high risk non-metastatic (M0) castration-resistant prostate cancer (NM-CRPC), defined as PSA Doubling Time (PSADT) ≤ 10 months.

ARN-509 will be administered orally on a continuous daily dosing schedule, at a starting dose of 240 mg per day in the treatment group. Matched placebo will be administered orally on a continuous daily dosing schedule, at a starting dose of 240 mg per day in the placebo group.

Patients will be followed for safety and efficacy as per the schedule of assessments and will remain on study treatment until documented radiographic progression (development of distant metastases as assessed by blinded independent central review [BICR]) or the development of unacceptable toxicity.

Patients discontinuing treatment due to documented radiographic progression will enter the survival follow-up period, where they will be followed for the development of symptomatic progression, initiation of subsequent anti-cancer therapies (in particular, cytotoxic chemotherapy) every 4 months until death, loss of follow-up, or withdrawal of consent, whichever comes first.

Patients discontinuing treatment prior to documented radiographic progression will also enter the survival follow-up period where they will continue to have scheduled disease assessments every 4 months until documented radiographic progression, and will be followed for the development of symptomatic progression and initiation of subsequent anti-cancer therapies (in particular, cytotoxic chemotherapy) every 4 months until death, loss of follow-up, or withdrawal of consent, whichever comes first.

2.1 STUDY OBJECTIVES

Primary Objective

- To demonstrate superiority in the metastasis-free survival (MFS) of men with high-risk NM-CRPC treated with ARN-509 versus placebo

Secondary Objectives

- To compare the overall survival (OS) of men with high risk NM-CRPC treated with ARN-509 versus placebo

- To compare the time to symptomatic progression in men with high-risk NM-CRPC treated with ARN-509 versus placebo
- To compare the time to initiation of cytotoxic chemotherapy in men with high-risk NM-CRPC treated with ARN-509 versus placebo
- To compare the progression-free survival (PFS) of men with high-risk NM-CRPC treated with ARN-509 versus placebo
- To compare the time to metastasis (TTM) in men with high-risk NM-CRPC treated with ARN-509 versus placebo
- To evaluate the safety and tolerability of ARN-509

Other Objectives

- To compare patient reported outcomes (PROs) of health-related quality of life and prostate cancer-specific symptoms in men with high risk NM-CRPC treated with ARN-509 versus placebo
- To evaluate the population pharmacokinetics of ARN-509
- To evaluate the effect of ARN-509 on ventricular repolarization in a subset of patients from selected clinical sites [Appendix 8 of the protocol]
- To evaluate exploratory biomarkers predictive of response and resistance to ARN-509 treatment

2.2 STUDY ENDPOINTS

Primary Endpoint

- Metastasis-Free Survival (MFS)

Secondary Endpoints

- Overall Survival (OS)
- Time to symptomatic progression
- Time to initiation of cytotoxic chemotherapy
- Progression-Free Survival (PFS)
- Time to Metastasis (TTM)

Other Evaluations

- Health-related quality of life and prostate cancer-specific symptoms
- Type, incidence, toxicity, timing, seriousness, and relatedness of adverse events and laboratory abnormalities
- PSA response
- Time to PSA progression
- Population pharmacokinetics
- Exploratory biomarkers

- Assessment of ventricular repolarization [Appendix 8 of the protocol]
- Second progression-free survival (PFS2)

3. RANDOMIZATION

Upon verification of inclusion and exclusion criteria, eligible patients will be centrally randomized in a 2:1 ratio to either ARN-509 or placebo. The randomization will be stratified as follows:

- PSADT (≤ 6 months vs. > 6 months)
- Bone-sparing agent use: Yes vs. No
- Loco-regional disease: N0 vs. N1

In order to ensure accurate and consistent determination of PSADT, the Interactive Voice Recognition System (IVRS) will also provide PSADT calculations (using a linear regression model of the natural logarithm of PSA and time) based on PSA values entered by the sites prior to randomization.[1]

4. SAMPLE SIZE DETERMINATION

The primary efficacy analysis will be event-driven and will take place when approximately [redacted] MFS events have occurred. The study provides 90% power to detect a [redacted] reduction in the risk of developing metastases (HR = [redacted]) for patients receiving ARN-509, with a 2-sided α of 0.05.[2] Based on an assumed median MFS of 25 months in the placebo group, this treatment effect represents an increase in the median MFS of approximately [redacted] months (from 25 months to [redacted] months). Assuming an accrual period of 24 months (with [redacted] of the patients accrued in the second year), approximately 1200 patients will need to be randomized.

The study was also designed to provide 85% power to detect a [redacted] reduction (HR = [redacted]) in the risk of death for patients receiving ARN-509, based on an assumed median OS of [redacted] months in the placebo group. This treatment effect represents an increase in the median OS of approximately [redacted] months (from [redacted] months).

5. INTERIM ANALYSIS

There will be no interim analysis for the primary endpoint (MFS) and for the secondary endpoints of [redacted]. There will be 1 interim analysis for [redacted], and up to 2 interim analyses for [redacted]; see Section 7.5.3 for details.

6. INDEPENDENT DATA MONITORING COMMITTEE

An independent third-party Data Monitoring Committee (IDMC) will be established to ensure the overall integrity and conduct of the study.

The IDMC will review the progress of the study and cumulative unblinded safety data on a periodic basis (e.g., a minimum of two review meetings per year) as well as review the efficacy analysis when necessary. In addition to the review meetings, blinded (or unblinded,

if necessary) listings of serious adverse events will be provided to the IDMC on a monthly basis.

Following each review meeting, the IDMC will recommend to the Sponsor whether to continue the trial unchanged, modify the conduct of the study, or terminate the study early. Rules for early termination, modification or continuation of the study, as well as how these recommendations will be made to the Sponsor and Health Authorities will be outlined in a separate IDMC Charter.

The IDMC will be composed of 3 external members [2 physicians and 1 biostatistician] not associated with the conduct of the study. The Sponsor will also designate an independent biostatistician not affiliated with the project to prepare and provide study data to the IDMC. Complete details regarding the composition and governance of the IDMC will be outlined in the IDMC Charter.

Periodic adverse event data review will also be performed by designated members of the Sponsor's primary study team and will be blinded to treatment assignment with adverse event data from both treatment groups combined. Any safety issues of concern identified by the primary study team that require notification of the IDMC will be communicated as described in the IDMC Charter.

7. STATISTICAL METHODS

The primary objective of the study is to evaluate the efficacy of ARN-509 compared to placebo in patients with high risk NM-CRPC as measured by metastasis-free survival (MFS), based on blinded independent central review (BICR) of tumor assessments.

7.1 ANALYSIS POPULATIONS

Full Analysis (Intent-to-Treat) Population [ITT]: All eligible patients who are randomized into the study, with study drug assignments designated according to initial randomization, regardless of whether patients receive study drug or receive a different drug from that to which they were randomized to will be included in the analyses of all efficacy and clinical benefit endpoints and patient characteristics.

Safety Analysis Population [SAFETY]: All patients who receive at least one dose of study drug, with treatment assignments designated according to actual study treatment received will be the primary population for evaluating safety and treatment compliance and administration.

- **Population Pharmacokinetics Population [PK]**: Subset of the safety analysis population that was randomized to the ARN-509 treatment group and that has at least one PK sample collected.
- **Biomarker Population**: Subset of the safety analysis population that has at least 1 biomarker sample collected.

7.2 DEFINITIONS

Study Day: Study day will be calculated in reference to the date of randomization for randomized untreated subjects and in reference to the date of first dose for treated subjects.

Study Day 1 corresponds to the date the subject was randomized into the study or to the date of first dose the subject was treated.

Baseline Value: Unless otherwise specified, the baseline value will be defined as the closest measurement prior to the first dose of study drug. Change from baseline will be defined as (post-baseline value – baseline value).

Treatment Duration: Treatment duration will be defined as the duration of time from the date of the first dose of study drug to the date of last dose of study drug + 1 day.

Time to event: Time to event calculations will be defined as the time from randomization to date of event + 1 day. Time to event or duration of event endpoints will be based on the actual date of the event, not visit number or visit label.

Survival Follow-Up Phase: The survival follow-up phase will start from the safety follow-up visit (28 days following the last dose of study drug) and continue through the end of the study.

7.3 ANALYSIS OF STUDY CONDUCT

All patients randomized into the study will be summarized by treatment group as randomized. The randomization stratification factors will be listed and tabulated as recorded in the IVRS by treatment group as randomized.

The number of patients who are in the ITT and SAFETY populations will be summarized by treatment group and overall.

Study treatment administration, duration of follow-up, discontinuation from study treatment and the reasons for discontinuation will be summarized by treatment group for all randomized patients. In addition, major protocol deviations and eligibility violations will also be summarized by treatment group. Protocol violations for this study include, but are not limited to, the following:

- Failure to meet inclusion/exclusion criteria
- Use of a prohibited concomitant medication
- Dose modifications that are not within the protocol specifications
- Any other deviation that presents significant risk or safety concerns to the patient

7.4 ANALYSIS OF TREATMENT GROUP COMPARABILITY

The evaluation of treatment group comparability between the 2 treatment groups will include summary of demographics, baseline disease characteristics, medical history, and patient treatment history.

Descriptive statistics (mean, standard deviation, median, range) will be presented by treatment group for continuous variables such as age and time from initial diagnosis. Categorical variables will be summarized using frequencies and percentages.

7.4.1 Demographics and Baseline Characteristics

The following parameters will be summarized overall by treatment group as randomized for all patients in the ITT population: Age, race, ethnicity and baseline weight and height.

7.4.2 Disease Characteristics and Prior Therapy

The following parameters will be summarized overall by treatment group as randomized for all patients in the ITT population:

- ECOG performance status
- Baseline PSA value and PSA Doubling Time (PSADT)
- Time (months) from initial diagnosis of prostate cancer to randomization
- Total Gleason Score at initial diagnosis
- Tumor stage at initial diagnosis
- Lymph nodes stage at initial diagnosis
- Number of prior hormonal therapies
- Use of bone-sparing agent (Yes/No) at baseline
- History of surgical prostate cancer procedures (Yes/No)
- Type of surgical procedures (prostatectomy, orchiectomy, transurethral resection of the prostate (TURP), and other)
- History of radiotherapy (Yes/No)
- Type of radiotherapy
- History of prior adjuvant/neoadjuvant chemotherapy (Yes/No)

7.4.3 Study Drug Administration

The SAFETY population will be used to summarize drug exposure, treatment compliance, and dose modifications by treatment group as treated.

Treatment duration will be defined as the duration of time from the date of the first dose of study drug to the date of last dose of study drug + 1 day.

The total cumulative dose in milligrams (mg) will be calculated as 30 or 60 mg multiplied by the number of capsules or tablets taken. The number of capsules or tablets taken will be calculated based on the number of capsules or tablets dispensed at the study visits minus the number of capsules or tablets indicated as having been returned.

The overall treatment compliance will be defined as the total dose in mg taken during the study divided by the expected total dose in mg. A subject's expected total dose will be calculated as the assigned dose per day multiplied by treatment duration. Each patient should be taking 8 capsules or 4 tablets per day maximum while on the study. For patients with dose reductions, the expected number of capsules or tablets will be reflective of the new dose with a reduced number of total capsules or tablets.

Dose reduction or interruption and the reason for the dose reduction or interruption will be summarized by treatment group as treated.

7.4.4 Pre-study and Concomitant Medications

Concomitant medications and medications taken prior to starting study treatment will be summarized for all patients in the SAFETY population by treatment group as treated. Medications are considered concomitant if taken during the treatment-emergent period. Prior medications are medications with the start date and/or end date before study drug date. Medications will be summarized by WHO Drug therapeutic class and generic medication name.

7.4.5 Subsequent Anti-Cancer Therapy

Subsequent systemic anti-cancer therapy taken after the patient has discontinued study drug will be summarized by treatment group and overall. Medications will be summarized by WHO Drug therapeutic class and generic medication name.

7.5 EFFICACY ANALYSES

The following section outlines the planned analyses of the primary and secondary efficacy outcomes of the study.

Efficacy analyses will be performed on the ITT population, incorporating the randomization stratification factors as documented on the IVRS, unless otherwise specified.

Time-to-event endpoints will be summarized using the Kaplan-Meier method [3] and displayed graphically where appropriate. Median event times and 2-sided 95% confidence interval for each median will be provided. Cox proportional-hazard models, including the stratification factors at baseline, will be used to estimate the hazard ratio (HR) and its 95% confidence interval (CI).

Response endpoints (eg, PSA response rate) will be summarized using descriptive statistics for categorical data by treatment group. The relative risk (treatment:control) will be reported along with the associated 2-tailed 95% CIs. The two treatment groups will be compared using the stratified Mantel-Haenszel test; Fisher's exact test may be used if the expected counts in some cells are small.

7.5.1 Blinded Independent Central Review (BICR)

Analyses of efficacy endpoints which are based on radiographic tumor assessments (MFS, TTM, and PFS) will be based on the results of the blinded independent central review (BICR), provided via electronic data transfer by the third-party core imaging laboratory.

All scans will be submitted for independent review of disease progression during the study according to an Independent Review Charter to be prepared by the core imaging laboratory in consultation with the Sponsor.

7.5.2 Primary Endpoint (MFS)

The primary efficacy endpoint is metastasis-free survival (MFS), defined as the time from randomization to first evidence of BICR-confirmed radiographically detectable bone or soft tissue distant metastasis (simply referred to as "metastasis" from this point forward) or death due to any cause (whichever occurs earlier) + 1 day.

MFS data for patients without metastasis or death will be censored on the date of the last tumor assessment (or, if no tumor assessment was performed after the baseline visit, at the date of randomization + 1 day).

Additional censoring rules will vary according to whether the analysis is performed for US or ex-US regulatory purposes, as follows; both results will be provided in the clinical study report.

Scenario	US regulatory guidance	ex-US regulatory guidance[4]
Data from patients who are lost to follow-up or whose disease progression (development of metastasis) or death occurs after 2 or more consecutively missing or unevaluable tumor assessments	Censored on the date of the last tumor assessment that the patient was known to be metastasis-free	Time of progression will be determined using the first date when there is documented evidence of progression or death (whichever occurs earlier) regardless of missed or unevaluable tumor assessments
Patients that receive new systemic anti-cancer therapy prior to documented disease progression (development of metastasis) or death	Censored on the date of the last tumor assessment prior to the start of the new systemic anti-cancer therapy	Time of progression will be determined using the first date when there is documented evidence of progression or death (whichever occurs earlier) regardless of change of therapy

Disease progression (development of metastasis) will be assessed by an independent core imaging laboratory using the Response Evaluation Criteria in Solid Tumors (RECIST v1.1).[5] The appearance of new (or distant) metastatic lesions denotes disease progression. For new bone lesions detected on bone scans, a second imaging modality (e.g., CT or MRI) will be required to confirm progression.

The primary efficacy analysis will be completed when approximately [redacted] MFS events have occurred. The primary analysis will compare the MFS distributions in the two treatment groups using a two-sided log-rank test, stratified by PSADT (≤ 6 months vs. > 6 months), the use of a bone-sparing agent (Yes vs. No), and the presence of loco-regional disease (N0 vs. N1) at the 0.05 significance level. The non-stratified log-rank test will be provided as a sensitivity analysis. Additional sensitivity analyses are provided in Section7.8

7.5.3 Secondary Endpoints

Time-to-event-based secondary endpoint analyses [redacted] ill be performed using a two-sided stratified log-rank test. Non-stratified log-rank tests will be provided as sensitivity analyses for OS.

For ease of notation, in the sequel, [redacted] is abbreviated as [redacted] and [redacted] is abbreviated as [redacted]
A hierarchical adaptive group sequential procedure will be used to test the secondary endpoints (Figure). The method allows re-estimation of the number of events necessary for the next best opportunity to achieve the desired conditional power. The method controls the familywise type I error rate for the primary and all secondary endpoints.

[redacted]

Specifically, a hierarchical testing will be performed in the following order: [redacted], each at alpha=0.05 (2-sided). As depicted in Figure 1, each endpoint will have a final analysis (FA) but there will be no interim analysis (IA) for TTM [redacted], 1 IA for [redacted], and up to 2 IAs for [redacted]. The testing of [redacted] endpoints will utilize an adaptive group sequential method, according to the pre-specified O'Brien-Fleming (OBF)-type alpha spending function[6] with possible re-estimation of the required number of events necessary for the next analysis to maintain the desired conditional power. If [redacted] is significant at the IA, then there will be only 1 IA for [redacted] (ie, with "IA #2" boxes removed from Figure 1); otherwise there will be 2 IAs for [redacted].

The FA of [redacted], IA of [redacted], and the first IAs of [redacted] will all be performed at the same time as the primary analysis of MFS (approximately [redacted] events). At the time of the MFS analysis, testing of the [redacted] endpoints will be carried out using the OBF-type alpha spending function and nominal p-values will be provided.

Assuming that the primary test for MFS is significant at alpha=0.05, [redacted] will then be tested at alpha=0.05. If significant, then [redacted] will be tested at alpha=0.05. If [redacted] is significant, then the Sponsor will perform the IA for [redacted]. Assuming HR [redacted] for [redacted], then [redacted] events are required to yield 80% power at alpha=0.05. Suppose that *X1* events will have accumulated at the IA for [redacted], the information fraction would be *t=X1/*[redacted]. Efficacy of [redacted] will be concluded if the stratified log-rank test statistic exceeds the critical value derived from the OBF-type alpha spending function.

The [redacted] events re-estimation will be based on a conditional power of 90% for the next stage, calculated using the observed HR. Note that because of the variability associated with the observed HR, a conditional power of 90% is used in order to maintain an overall power of approximately 80% if the true HR is [redacted]. The recommended number of [redacted] events for the FA should be in the range of [redacted] (additional [redacted]-*X1* events set as the minimum required for the next stage) to *X1*+([redacted]-*X1*)*1.1 (not more than 10% increase on the pre-planned number of events for the next stage). Because the true HR for [redacted] is unknown, the minimum number of [redacted] is chosen to yield at least 80% power to detect an HR of [redacted] at an alpha level of 0.05.

The [redacted] events re-estimation will be performed by the independent statistician who supports the IDMC activities. The dissemination and review of the specific results of the IA will be limited to the IDMC.

In order to maintain a strong control of the type I error rate for the [redacted] analysis, an inverse normal p-value combination method will be used as the final test. The inverse normal p-value combination method allows flexible adaptations at an IA and creates a valid test that controls the type I error rate in a strong sense analytically. In this proposed design the adaptation is the potential adjustment of the required number of events for the next stage.

The final test statistics for the null hypothesis H_0: HR for [redacted] ≥ 1 is defined as

$$Z = w_1 F^{-1}(1 - p_1) + w_2 F^{-1}(1 - p_2),$$

where $F^{-1}(x)$ is the inverse of the standard normal cumulative distribution function, $w_1 = \sqrt{t}$, $w_2 = \sqrt{1 - t}$, p_1 denotes the first stage p-value and p_2 denotes the second stage p-value.

Critical values for success are calculated based on the OBF-type alpha spending function. The study may be stopped early for efficacy if the interim test statistic exceeds the first stage critical value z_1, or stops for success at the second stage, if the final test statistic exceeds the second stage critical value z_2. Therefore, the null hypothesis H_0 will be rejected either at the first analysis if $F^{-1}(1 - p_1) > z_1$, or at the FA if $Z > z_2$.

The adaptive group sequential testing of [redacted] will be similar to that of [redacted] as described above, except that there is a possibility of 1 more IA to be incorporated in the inverse normal p-value combination method. Due to the hierarchical

structure of the testing procedure, an outline of some additional technical details is provided in Appendix 1.

7.5.3.1 Overall Survival

OS will be defined as the time from randomization to the date of death due to any cause + 1 day. Patients who are alive at the time of the analysis will be censored on the last known date that they were alive. In addition, the following censoring rules will apply:

Scenario	Date of Censoring
Patients with no post-baseline information	Censored on the date of randomization + 1 day
Patients who are lost to follow-up or who withdraw consent for further follow-up	Censored on the last known date that they were alive

Survival rate at 1-, 2-, 3- and 5-year will be estimated using the Kaplan-Meier method. Additional sensitivity analyses are provided in Section 7.8.

7.5.3.2 Time to Symptomatic Progression

Time to symptomatic progression will be defined as the time from randomization to documentation in the CRF of any of the following (whichever occurs earlier) + 1 day:

- Development of a skeletal-related event (SRE): pathologic fracture, spinal cord compression, or need for surgical intervention or radiation therapy to the bone.
- Pain progression or worsening of disease-related symptoms requiring initiation of a new systemic anti-cancer therapy.
- Development of clinically significant symptoms due to loco-regional tumor progression requiring surgical intervention or radiation therapy.

Adverse event, concomitant medication, or survival follow-up CRFs may also be the source of these findings.

Time to symptomatic progression for patients who do not experience any of the events described above will be censored on the date on which they were last known to be event-free.

7.5.3.3 Time to Initiation of Cytotoxic Chemotherapy

Time to initiation of cytotoxic chemotherapy will be defined as the time from randomization to documentation of a new cytotoxic chemotherapy being administered to the patient (e.g., survival follow-up CRF) + 1 day.

Time to initiation of cytotoxic chemotherapy for patients who do not start a cytotoxic chemotherapy will be censored on the date of last contact.

7.5.3.4 Progression-Free Survival

In order to capture loco-regional disease progression, a secondary endpoint of progression-free survival (PFS) will be assessed and defined as the time from randomization to first

documentation of BICR-confirmed radiographic progressive disease or death due to any cause (whichever occurs first) + 1 day.

Progressive disease (PD) will be based on RECIST v1.1, and further defined as follows:

- For patients with at least one measurable lesion, PD will be defined as at least a 20% increase in the sum of diameters of target lesions taking as reference the smallest sum on study (this includes the baseline sum if that is the smallest on study). In addition to the relative increase of 20%, the sum must also demonstrate an absolute increase of at least 5 mm. Furthermore, the appearance of one or more new lesions is also considered progression.
- For patients with only non-measurable disease observed on CT or MRI scans, unequivocal progression (representative of overall disease status change) or the appearance of one or more new lesions will be considered progression. For new bone lesions detected on bone scans, a second imaging modality (e.g., CT or MRI) will be required to confirm progression.

Progression-free survival data for patients without loco-regional disease will be censored on the date of the last tumor assessment (or, if no tumor assessment was performed after the baseline visit, at the date of randomization + 1 day). Additional censoring rules will vary according to whether the analysis is performed for US or ex-US regulatory purposes, as shown below; both results will be provided in the clinical study report.

**Scenario**	**US regulatory guidance**	**ex-US regulatory guidance**
Data from patients who are lost to follow-up or whose disease progression or death occurs after 2 or more consecutively missing or unevaluable tumor assessments	Censored on the date of the last tumor assessment that the patient was known to be progression-free	Time of progression will be determined using the first date when there is documented evidence of progression or death (whichever occurs earlier) regardless of missed or unevaluable tumor assessments
Patients that receive new systemic anti-cancer therapy prior to documented disease progression or death	Censored on the date of the last tumor assessment prior to the start of the new systemic anti-cancer therapy	Time of progression will be determined using the first date when there is documented evidence of progression or death (whichever occurs earlier) regardless of change of therapy

7.5.3.5 Time to Metastasis

Time to Metastasis (TTM) will be defined as the time from randomization to first evidence of BICR-confirmed radiographically detectable bone or soft tissue distant metastasis + 1 day.

TTM data for patients without metastasis will be censored on the date of the last tumor assessment (or, if no tumor assessment was performed after the baseline visit, at the date of randomization + 1 day). Additional censoring rules will vary according to whether the analysis is performed for US or ex-US regulatory purposes, as shown below; both results will be provided in the clinical study report.

**Scenario**	**US regulatory guidance**	**ex-US regulatory guidance**
Data from patients who are lost to follow-up or whose disease progression (development of metastasis) occurs after 2 or more consecutively missing or unevaluable tumor assessments	Censored on the date of the last tumor assessment that the patient was known to be metastasis-free	Time of progression will be determined using the first date when there is documented evidence of progression regardless of missed or unevaluable tumor assessments
Patients that receive new systemic anti-cancer therapy prior to documented disease progression (development of metastasis)	Censored on the date of the last tumor assessment prior to the start of the new systemic anti-cancer therapy	Time of progression will be determined using the first date when there is documented evidence of progression regardless of change of therapy

7.6 SAFETY ANALYSES

7.6.1 Adverse Events

Patients will be assessed for adverse events at each monthly clinic visit while on the study. Adverse events (AEs) will be graded according to the NCI Common Terminology Criteria for Adverse Events (CTCAE) Version 4.0 and coded to preferred term and system organ class (SOC) using the most recent version of MedDRA.

All AEs reported during the AE reporting period (inclusive of the 28-day post last dose of study drug period) will be considered as treatment-emergent adverse events and will be summarized by treatment group as treated using all patients in the SAFETY population.

For each treatment group, adverse event incidence rates will be summarized with frequency and percentage by MedDRA SOC and preferred term, with all patients treated in that treatment group as the denominator, unless otherwise specified. In addition, AE incidence rates will also be summarized by severity and relationship to study drug. Treatment-related AEs are those judged by the Investigator to be at least possibly related to the blinded study drug. Patients with multiple occurrences of events will only be counted once at the maximum severity to study drug for each preferred team, SOC, and overall. Deaths that occur within 28 days after the last dose of study drug are defined as on-study deaths.

Summary tables of the following AEs will be provided:

- Overall summary of AEs: the number and percentage of patients who experienced any AE, any serious adverse event (SAE), any treatment-related AE, any treatment-related SAE, any discontinuations due to an AE, and any deaths
- All AEs by SOC and preferred term
- All AEs by SOC, preferred term, and toxicity grade
- All AEs by decreasing frequency of preferred term
- Grades 3 or 4 AEs by SOC, preferred term and toxicity grade
- Drug-related AEs by SOC and preferred term
- Drug-related AEs by SOC, preferred term, and toxicity grade
- Drug-related Grades 3 or 4 AEs by SOC, preferred term and toxicity grade
- AEs that led to study drug discontinuation by SOC and preferred term. Study drug discontinuation will be determined from the End of Treatment CRF (where reason for termination is “Adverse Event”) and the specific AE will be determined from the AE CRF page (where action taken is “Withdrawn from Study”)
- AEs that led to study drug discontinuation by SOC, preferred term, and toxicity grade.
- All SAEs by SOC and preferred term
- All SAEs by SOC, preferred term, and maximum severity
- Deaths will be summarized by time period (on-study vs. during follow-up) and cause of death.

Patient listings of all Grades 3 or 4 AEs, all SAEs, AEs that led to study drug discontinuation and all deaths will be provided as well.

Narratives will be written for the following patients in the final clinical study report:

- Patients who die within 28 days of the last dose of study drug
- Patients who discontinue study drug due to adverse events
- Patients who have a serious adverse event
- Patients who experience a seizure
- Grade 3 or higher adverse events of special interest

7.6.2 Laboratory Abnormalities

Only data collected by the central laboratory will be summarized. Local laboratory data collected for the purposes of planning treatment administration, dose modification, or monitoring adverse events, will not be summarized.

Normal ranges will be used to identify values that are outside the normal ranges and abnormal laboratory results will be graded according to the NCI CTCAE Version 4.0.

Descriptive statistics will be provided for selected test results and for the change from baseline by visit.

A shift summary of baseline grade by maximum post-baseline CTCAE grade will be presented, as appropriate. For each laboratory parameter, the baseline laboratory value will be defined as the last laboratory value collected on or prior to the date of the first dose of study drug. Patients who develop toxicities of Grade ≥ 3 will be listed.

7.6.3 Vital Signs

Each vital sign (temperature, blood pressure (systolic and diastolic), respiration rate, and heart rate) at baseline will be summarized and presented by treatment group. The number and percentage of subjects with marked abnormalities in vital signs as compared to baseline will be summarized and listed.

Data will be summarized and presented according to the following categories:

Parameter	Criteria for Marked Abnormality
Systolic Blood Pressure	Absolute result > 160 mmHg and increase from baseline > 20 mmHg
	Absolute result < 90 mmHg and decrease from baseline > 20 mmHg
Diastolic Blood Pressure	Absolute result > 100 mmHg and increase from baseline > 10 mmHg
	Absolute result < 50 mmHg and decrease from baseline > 10 mmHg
Heart Rate	Absolute result > 100 bpm and increase from baseline > 30 bpm
	Absolute result < 60 bpm and decrease from baseline > 20 bpm

7.7 OTHER EVALUATIONS

7.7.1 Second Progression-Free Survival (PFS2)

PFS2 is defined as the time from randomization to second documentation of investigator-assessed disease progression (PSA, radiographic, symptomatic, or any combination) or death (any cause) on subsequent treatment (whichever occurs first) + 1 day. Subjects without a documented progression will be censored at the last date known to be progression-free or death whichever occur first.

PFS2 will be analyzed using time-to-event analysis methods outlined in section 7.5.

7.7.2 PSA

PSA kinetics (e.g., PSA response and time to PSA progression) will be assessed at the time of the primary analysis of MFS according to the Prostate Cancer Clinical Trials Working Group (PCWG2) criteria.[8]

Summary tables and waterfall plots describing change in PSA relative to baseline will be reported at 12 weeks (or earlier for those who discontinue study treatment prior to 12 weeks), and separately, the maximum change at any time on study will also be reported for each patient using summary tables and waterfall plots.

PSA response rate will be summarized by treatment group. The relative risk (treatment:control) will be reported along with the associated 2-tailed 95% CIs. The two treatment groups will be compared using the stratified Mantel-Haenszel test.

The time to PSA progression will be calculated as the time from randomization to the time when the criteria for PSA progression according to PCWG2 are met + 1 day. Kaplan-Meier methods will be used to estimate the median time to PSA progression and 95% confidence intervals for each treatment group

7.7.3 Health-Related Quality of Life and Prostate Cancer-Specific Symptoms

The FACT-P and EQ-5D data will be scored and handled as recommended in their respective User's manuals, including handling of missing data both within the subscales and overall.[9,10] All FACT-P and EQ-5D data analyses will be performed in the ITT population. On-study scores and change from baseline scores will be summarized and displayed graphically as appropriate.

A 10-point change in the FACT-P total score is considered clinically meaningful.[11] Therefore, any patient experiencing a 10-point decrease in FACT-P total scores from baseline at any post baseline time point will be considered to have experienced clinically meaningful deterioration in functional status. The proportions of patients with a 10-point decrement in FACT-P total score will be summarized by treatment group, and the two treatment groups will be compared using a Mantel-Haenszel test, stratified by PSADT (> 6 months vs. ≤ 6 months), the use of a bone-sparing agent (Yes vs. No), and the presence of loco-regional disease (N0 vs. N1) at a two-sided 0.05 significance level.

The EQ-5D data will be summarized descriptively by treatment group and study visit.

Details on the PRO analyses will be provided in a separate PRO SAP.

7.7.4 Population Pharmacokinetic Analyses

A separate Population PK analysis plan will be prepared prior to database lock. A separate report will be generated.

7.7.5 Exploratory Biomarker Analyses

Biomarker analyses are exploratory and data generated will be reported separately.

7.7.6 Assessment of Ventricular Repolarization

The assessment of ventricular repolarization will be a sub-study conducted in a subset of patients from selected clinical sites and analyzed by an independent cardiac safety laboratory. Description of the sub-study is provided in Appendix 8 of the study protocol. A separate analysis plan will be prepared.

7.7.7 Medical Resource Utilization Analysis

Protocol-mandated procedures, tests, and encounters are excluded. The MRU data may be used to conduct economic analyses.

7.8 SENSITIVITY ANALYSES

Sensitivity analyses will be performed on the primary efficacy endpoint of MFS and secondary endpoint of OS to support the results in the primary analysis.

7.8.1 Investigator-Derived MFS

A sensitivity analysis will be performed on the primary endpoint of MFS using the investigator-derived progression (development of metastasis or death). Patients without a MFS event will be censored on the known date of progression-free according to the investigator tumor assessment.

7.8.2 OS

Sensitivity analyses for the OS may be carried out as appropriate if it is deemed useful to aid in the interpretation of the results.

7.8.2.1 Covariate Effects

A non-stratified multivariate analysis will be performed on the OS endpoint to estimate treatment effect adjusting for important baseline factors. The following baseline covariates will be considered:

- PSADT (≤ 6 months vs. > 6 months)
- Bone-sparing agent use (Yes vs. No)
- Loco-regional disease (N0 vs. N1)
- ECOG Performance status (0 vs. 1)
- Number of prior hormonal therapies (1 vs. ≥ 2)
- Gleason score (≤ 7 vs. ≥ 8)
- Age (continuous)
- Logarithm of PSA (continuous)

The adjusted hazard ratio and its 95% confidence interval for treatment and each factor will be estimated using Cox regression.

7.8.2.2 Other Sensitivity Analyses for OS

A large number of subjects are expected to receive life-extending subsequent therapies, the following analyses may be used in estimating the true treatment effect.

1. Inverse Probability of Censoring Weighted (IPCW) log-rank Tests by Robins et al[12,13,14,15] will be used to estimate the treatment effect and its associated confidence interval.

2. Using a time-dependent Cox regression; the HR prior to receiving subsequent anticancer therapy and after receiving the subsequent anticancer therapy will be estimated, the associated 95% confidence interval will also be calculated.

Additional analyses may be performed for OS if appropriate.

7.8.3 Subgroup Analyses

In order to assess the consistency of treatment benefit with respect to the primary efficacy endpoint of MFS and secondary endpoint of OS across important subgroups, forest plots will be provided for the following variables:

- ECOG performance status (0 vs. 1) at baseline
- Age category (< 65 vs. ≥ 65 years and < 75 vs. ≥ 75 years)
- Race (white, black, Asian, and others)
- Geographic region (NA, EU, and ROW)
- Number of prior hormonal therapies (1 vs. ≥ 2)
- Baseline PSA value (at or below median vs. above median)
- PSADT (> 6 months vs. ≤ 6 months)
- Bone-sparing agent use (Yes vs. No)
- Loco-regional disease (N0 vs. N1)

The comparison between treatment groups will be evaluated by a single hazard ratio with its 95% confidence interval based on a non-stratified Cox regression model for each subgroup.

7.9 ANALYSIS BY FORMULATION

During the conduct of the study, treatment formulation was switched from capsule to tablet, at which time a significant number of subjects have been already enrolled and have received capsule formulation. The randomization and blinding remained unchanged during the formulation switch. Newly enrolled subjects began treatment using tablets while subjects already enrolled and receiving capsules made the switch to tablets at the start of a new cycle.

As outlined in previous sections, the primary analyses will be based on the Safety and ITT Populations irrespective of the formulation switch. However, to show that the two formulations have no clinically relevant impact on key efficacy and safety data, supplemental descriptive analyses will be performed. For safety analyses, the descriptive analysis by formulation subgroups (capsule only, capsule/tablet, and tablet only) will be performed.

7.9.1 Subject Distribution by Formulation

For each formulation subgroup the following summaries by treatment group will be generated:

- Number of subjects randomized, treated, discontinued and ongoing
- Primary reasons for study treatment discontinuation

7.9.2 Demographics and Baseline characteristics by Formulation

The parameters outlined in section 7.4.1 will be summarized by treatment group and formulation. The summary will include subjects as randomized.

7.9.3 Study Drug Administration by Formulation

Descriptive statistics will be provided on dose modifications by treatment group and formulation and the subjects will be included as treated.

7.9.4 Safety Analyses by Formulation

The by-formulation safety analysis will use subjects as treated and will include the following AEs summaries:

- Overall summary of AEs (See Appendix 2 for table shell)
- All AEs by System Organ Class (SOC), Preferred Term (PT) and toxicity grade
- Grade 3/4 AEs by SOC, PT and toxicity grade

7.9.5 Efficacy (MFS and PSA Response) Analyses by Formulation

The primary MFS analysis and statistical inference will be based on the ITT population regardless of the formulation as defined in section 7.5.2. In order to assess the consistency of treatment benefit (relative to ITT analysis), a supplemental analysis by formulation subgroup will be performed on the BICR derived MFS endpoint and PSA response.

The following formulation subgroups will be used in the analysis:

1. Capsule only, tablet only and capsule + tablet
2. Two groups based on greater duration of exposure on specific formulation: greater duration on capsule versus greater duration on tablet.

The MFS subgroup analysis will include the following:

- Number of events and censored
- Distribution of MFS endpoint using the Kaplan-Meier method
- Non-stratified estimate of the hazard ratio and its associated 95% confidence interval using the Cox model.

8. REFERENCES

1. Pound CR, Partin AW, Eisenberger MA, et al. Natural history of progression after PSA elevation following radical prostatectomy. JAMA 1999; 281:1591-7.
2. Collett D. Modeling Survival Data in Medical Research. Boca Raton: Chapman and Hall/CRC, 1994, pp 254-5.
3. Kaplan EL and Meier P. Nonparametric estimation from incomplete observations. J Am Stat Assoc 1958; 53:457-81.
4. Appendix 1 to the guideline on the evaluation of anticancer medicinal products in man: Methodological consideration for using progression-free survival (PFS) or disease-free survival (DFS) in confirmatory trials. European Medicines Agency 2011; Doc. Ref. EMA/CHMP/27994/2008.
5. Eisenhauer EA, Therasse P, Bogaerts J, et al. New response evaluation criteria in solid tumours: Revised RECIST guideline (version 1.1). Eur J Cancer 2009; 45:228-47.
6. Reboussin DM, DeMets DL, Kim KM, and Lan KKG. Computations for group sequential boundaries using the Lan-DeMets spending function method. Controlled Clinical Trials 2000; 21:190-207.
7. Wassmer, G. Planning and analyzing adaptive group sequential survival trials. Biometrical Journal 2006; 48:714-729.
8. Scher HI, Halabi S, Tannock I, et al. Design and end points of clinical trials for patients with progressive prostate cancer and castrate levels of testosterone: Recommendations of the Prostate Cancer Clinical Trials Working Group. J Clin Oncol 2008; 26:1148-59.
9. Esper P, Mo F, Chodak G, et al. Measuring quality of life in men with prostate cancer using the Functional Assessment of Cancer Therapy-Prostate (FACT-P) instrument. Urology 1997; 50:920-8.
10. The EuroQol Group. EuroQol – a new facility for the measurement of health-related qualify of life. Health Policy 1990; 16:199-208.
11. Cella D, Nichol MB, Eton D et al. Estimating clinically meaningful changes for the Functional Assessment of Cancer Therapy-Prostate: Results from a clinical trial of patients with metastatic hormone-refractory prostate cancer. Value in Health 2009; 12:124-129.
12. Herna M, Brumback B, Robins J. Marginal structural models to estimate the causal effect of zidovudine on the survival of HIV-positive men. Epidemiology 2000; 11: 561-570.
13. Robins J, Finkelstein D. Correcting for noncompliance and dependent censoring in an AIDS clinical trial with inverse probability of censoring wieighted (IPCW) log-rank tests. Biometrics 2000; 56:779-788.

14. Robins J, Hernan N, Brumback B. Marginal structural models and causal inference in epidemiology. Epidemilogy 2010; 11: 550-560.

15. Westreich D, Cole S et al. Time scale and adjusted survival curves for marginal structural cox models. Am J Epidemiology 2010; 171: 691-700.

9. APPENDICES

[redacted]

[redacted]

[redacted]

9.2 APPENDIX 2: TABLE SHELL FOR OVERALL SUMMARY OF ADVERSE EVENTS BY FORMULATION

	**Placebo**			**Apalutamide**		
	Capsule	Tablet	Capsule+Tablet	Capsule	Tablet	Capsule+Tablet
Analysis Set: Safety population	###	###	###	###	###	###
Number of subjects with AEs	### (xx.x%)	### (xx.x%)	### (xx.x%)	### (xx.x%)	### (xx.x%)	### (xx.x%)
Drug-related[a]	### (xx.x%)	### (xx.x%)	### (xx.x%)	### (xx.x%)	### (xx.x%)	### (xx.x%)
Number of subjects with grade 3-4 AEs	### (xx.x%)	### (xx.x%)	### (xx.x%)	### (xx.x%)	### (xx.x%)	### (xx.x%)
Drug-related[a]	### (xx.x%)	### (xx.x%)	### (xx.x%)	### (xx.x%)	### (xx.x%)	### (xx.x%)
Number of subjects with SAEs	### (xx.x%)	### (xx.x%)	### (xx.x%)	### (xx.x%)	### (xx.x%)	### (xx.x%)
Drug-related[a]	### (xx.x%)	### (xx.x%)	### (xx.x%)	### (xx.x%)	### (xx.x%)	### (xx.x%)
Grade 3-4	### (xx.x%)	### (xx.x%)	### (xx.x%)	### (xx.x%)	### (xx.x%)	### (xx.x%)
Number of subjects with AEs leading to treatment discontinuation	### (xx.x%)	### (xx.x%)	### (xx.x%)	### (xx.x%)	### (xx.x%)	### (xx.x%)
Drug-related[a]	### (xx.x%)	### (xx.x%)	### (xx.x%)	### (xx.x%)	### (xx.x%)	### (xx.x%)
Number of subjects with AEs leading to death	### (xx.x%)	### (xx.x%)	### (xx.x%)	### (xx.x%)	### (xx.x%)	### (xx.x%)
Drug-related	### (xx.x%)	### (xx.x%)	### (xx.x%)	### (xx.x%)	### (xx.x%)	### (xx.x%)
All deaths within 28 days of last dose	### (xx.x%)	### (xx.x%)	### (xx.x%)	### (xx.x%)	### (xx.x%)	### (xx.x%)
Adverse event	### (xx.x%)	### (xx.x%)	### (xx.x%)	### (xx.x%)	### (xx.x%)	### (xx.x%)
Death due to prostate cancer	### (xx.x%)	### (xx.x%)	### (xx.x%)	### (xx.x%)	### (xx.x%)	### (xx.x%)
other						

[a] Adverse events reported as related.

Note: Percent is based on the Safety population.

Note: Treatment-emergent adverse events are those that occurred between the date of 1st dose of study drug and date of last dose of study drug+28 days. For each category, subjects are counted only once, even if they experienced multiple events in that category

9.3 APPENDIX 3: MODIFIED MEDDRA QUERIES AS SEARCH CRITERIA FOR AE OF SPECIAL INTEREST

The search criteria for adverse events of special interest are based on adverse event preferred terms from MedDRA version 19.1 dictionary.

Most categories are based on a MedDRA SMQ, but if one does not exist, a compilation of terms that reflect the event will be proposed for extraction and analysis of the data. Each of these events is defined below.

**Adverse Event of Special Interest Category= Seizure**	
**Search Criteria Category= Selected PTs**	
Acquired epileptic aphasia	Idiopathic generalised epilepsy
Acute encephalitis with refractory, repetitive partial seizures	Lafora's myoclonic epilepsy
Alcoholic seizure	Lennox-Gastaut syndrome
Amygdalohippocampectomy	Migraine-triggered seizure
Atonic seizures	Molybdenum cofactor deficiency
Atypical benign partial epilepsy	Myoclonic epilepsy
Aura	Myoclonic epilepsy and ragged-red fibres
Automatism epileptic	Narcolepsy
Autonomic seizure	Partial seizures
Baltic myoclonic epilepsy	Partial seizures with secondary generalisation
Benign rolandic epilepsy	Petit mal epilepsy
Biotinidase deficiency	Polymicrogyria
Change in seizure presentation	Post stroke epilepsy
Clonic convulsion	Post stroke seizure
Complex partial seizures	Post-traumatic epilepsy
Convulsion prophylaxis	Postictal headache
Convulsions local	Postictal paralysis
Convulsive threshold lowered	Postictal psychosis
Corpus callosotomy	Postictal state
Deja vu	Preictal state
Double cortex syndrome	Psychomotor seizures
Dreamy state	Schizencephaly
Drop attacks	Seizure

Drug withdrawal convulsions	Seizure anoxic
Epilepsy	Seizure cluster
Epileptic aura	Seizure like phenomena
Epileptic psychosis	Severe myoclonic epilepsy of infancy
Febrile convulsion	Simple partial seizures
Foaming at mouth	Status epilepticus
Frontal lobe epilepsy	Sudden unexplained death in epilepsy
Generalised non-convulsive epilepsy	Temporal lobe epilepsy
Generalised tonic-clonic seizure	Tongue biting
Glucose transporter type 1 deficiency syndrome	Tonic clonic movements
Hemimegalencephaly	Tonic convulsion
Hyperglycaemic seizure	Tonic posturing
Hypocalcaemic seizure	Topectomy
Hypoglycaemic seizure	Uncinate fits
Hyponatraemic seizure	
**Adverse Event of Special Interest Category= Skin rash**	
**Search Criteria Category= Selected PTs**	
Acquired epidermolysis bullosa	Noninfective conjunctivitis
Acute generalised exanthematous pustulosis	Oculomucocutaneous syndrome
Administration site hypersensitivity	Oral mucosal blistering
Administration site rash	Oral mucosal exfoliation
Administration site recall reaction	Oral papule
Administration site urticaria	Oropharyngeal blistering
Application site rash	Papule
Blau syndrome	Paraneoplastic rash
Blister	Pemphigoid
Blister rupture	Pemphigus
Bullous impetigo	Penile exfoliation
Butterfly rash	Perineal rash
Catheter site rash	Pogosta disease
Conjunctivitis	Rash
Corneal exfoliation	Rash erythematous
Cutaneous vasculitis	Rash follicular
Dermatitis bullous	Rash generalised

Dermatitis exfoliative	Rash macular
Dermatitis exfoliative generalised	Rash maculo-papular
Drug eruption	Rash maculovesicular
Drug reaction with eosinophilia and systemic symptoms	Rash morbilliform
Epidermal necrosis	Rash papular
Epidermolysis	Rash papulosquamous
Epidermolysis bullosa	Rash pruritic
Eruptive pseudoangiomatosis	Rash pustular
Erythema multiforme	Rash rubelliform
Exfoliative rash	Rash scarlatiniform
Eyelid rash	Rash vesicular
Familial cold autoinflammatory syndrome	Sea bather's eruption
Fixed drug eruption	Skin erosion
Genital rash	Skin exfoliation
Genital ulceration	Skin necrosis
HLA-B*1502 assay positive	Skin reaction
HLA-B*5801 assay positive	Skin swelling
Hyper IgD syndrome	Staphylococcal scalded skin syndrome
Hypopharyngeal synechiae	Stevens-Johnson syndrome
Implant site rash	Stoma site rash
Incision site rash	Stomatitis
Infusion site rash	Symmetrical drug-related intertriginous and flexural exanthema
Injection site rash	Systemic lupus erythematosus rash
Instillation site rash	Tongue exfoliation
Lip exfoliation	Toxic epidermal necrolysis
Lupus miliaris disseminatus faciei	Toxic skin eruption
Medical device site rash	Urticaria
Mouth ulceration	Urticaria papular
Mucocutaneous rash	Vaccination site rash
Mucocutaneous ulceration	Vaginal exfoliation
Mucosa vesicle	Vaginal ulceration
Mucosal erosion	Vascular access site rash
Mucosal exfoliation	Vessel puncture site rash
Mucosal necrosis	Viral rash

Mucosal ulceration	Vulval ulceration
Nikolsky's sign	Vulvovaginal rash
Nodular rash	Vulvovaginal ulceration
**Adverse Event of Special Interest Category= Changes in thyroid function**	
**Search Criteria Category= Selected PTs**	
Anti-thyroid antibody	Thyroglobulin present
Anti-thyroid antibody positive	Thyroid atrophy
Atrophic thyroiditis	Thyroid disorder
Autoimmune hypothyroidism	Thyroid dysfunction in pregnancy
Autoimmune thyroiditis	Thyroid electron radiation therapy
Biopsy thyroid gland abnormal	Thyroid function test abnormal
Blood thyroid stimulating hormone abnormal	Thyroid gland scan abnormal
Blood thyroid stimulating hormone decreased	Thyroid hemiagenesis
Blood thyroid stimulating hormone increased	Thyroid hormone replacement therapy
Butanol-extractable iodine decreased	Thyroid hormones increased
Butanol-extractable iodine increased	Thyroid operation
Congenital hypothyroidism	Thyroid pain
Congenital thyroid disorder	Thyroid releasing hormone challenge test abnormal
Euthyroid sick syndrome	Thyroid size decreased
Free thyroxine index abnormal	Thyroid stimulating hormone deficiency
Free thyroxine index decreased	Thyroid therapy
Free thyroxine index increased	Thyroidectomy
Gamma radiation therapy to thyroid	Thyroiditis
Generalised resistance to thyroid hormone	Thyroiditis acute
Goitre	Thyroiditis chronic
Hashimoto's encephalopathy	Thyroiditis fibrous chronic
Hypothyroidic goitre	Thyroiditis subacute
Hypothyroidism	Thyroxin binding globulin abnormal
Infectious thyroiditis	Thyroxin binding globulin decreased
Iodine uptake abnormal	Thyroxin binding globulin increased
Iodine uptake decreased	Thyroxine abnormal
Iodine uptake increased	Thyroxine decreased
Myxoedema	Thyroxine free abnormal
Myxoedema coma	Thyroxine free decreased

Photon radiation therapy to thyroid	Thyroxine free increased
Polyglandular autoimmune syndrome type II	Thyroxine increased
Polyglandular autoimmune syndrome type III	Thyroxine therapy
Post procedural hypothyroidism	Transient hypothyroxinaemia of prematurity
Primary hypothyroidism	Tri-iodothyronine abnormal
Protein bound iodine decreased	Tri-iodothyronine decreased
Protein bound iodine increased	Tri-iodothyronine free abnormal
Radioactive iodine therapy	Tri-iodothyronine free decreased
Radiotherapy to thyroid	Tri-iodothyronine free increased
Reverse tri-iodothyronine decreased	Tri-iodothyronine free normal
Reverse tri-iodothyronine increased	Tri-iodothyronine increased
Secondary hypothyroidism	Tri-iodothyronine uptake abnormal
Tertiary hypothyroidism	Tri-iodothyronine uptake decreased
Thyreostatic therapy	Tri-iodothyronine uptake increased
Thyroglobulin absent	Ultrasound thyroid abnormal
Thyroglobulin decreased	X-ray therapy to thyroid
Thyroglobulin increased	
**Adverse Event of Special Interest Category= Fall**	
**Search Criteria Category= Selected PTs**	
Fall	
**Adverse Event of Special Interest Category= Fracture**	
**Search Criteria Category= Selected PTs**	
Acetabulum fracture	Hip fracture
Ankle fracture	Humerus fracture
Atypical femur fracture	Ilium fracture
Atypical fracture	Impacted fracture
Avulsion fracture	Internal fixation of fracture
Cervical vertebral fracture	Jaw fracture
Chance fracture	Limb fracture
Clavicle fracture	Loss of anatomical alignment after fracture reduction
Closed fracture manipulation	Lower limb fracture
Comminuted fracture	Lumbar vertebral fracture

Complicated fracture	Multiple fractures
Compression fracture	Open fracture
Costal cartilage fracture	Open reduction of fracture
Craniofacial fracture	Open reduction of spinal fracture
Elevation skull fracture	Osteochondral fracture
Epiphyseal fracture	Osteoporotic fracture
External fixation of fracture	Patella fracture
Facial bones fracture	Pathological fracture
Femoral neck fracture	Pelvic fracture
Femur fracture	Periprosthetic fracture
Fibula fracture	Pubis fracture
Foot fracture	Radius fracture
Forearm fracture	Rib fracture
Fracture	Sacroiliac fracture
Fracture debridement	Scapula fracture
Fracture delayed union	Skull fracture
Fracture displacement	Skull fractured base
Fracture malunion	Spinal compression fracture
Fracture nonunion	Spinal fracture
Fracture of clavicle due to birth trauma	Spinal fusion fracture
Fracture of penis	Sternal fracture
Fracture pain	Stress fracture
Fracture reduction	Surgical fixation of rib fracture
Fracture treatment	Thoracic vertebral fracture
Fractured coccyx	Tibia fracture
Fractured ischium	Tooth fracture
Fractured maxilla elevation	Torus fracture
Fractured sacrum	Traumatic fracture
Fractured skull depressed	Ulna fracture
Fractured zygomatic arch elevation	Upper limb fracture
Greenstick fracture	Wrist fracture
Hand fracture	

ARN-509-003 (SPARTAN): Summary of Major Changes and Rationale for Each Statistical Analysis Plan Amendment

**Major Changes**	**Rationale**
**v1.0 (dated 05 November 2012) to v2.0 (dated 27 February 2014)**	
Overall survival (OS) was changed from being the only key secondary endpoint to being one of the 5 secondary endpoints that included [redacted] The statistical testing of secondary endpoints were to be performed by allocating [redacted] to OS and [redacted] for the rest of the endpoints using Bonferroni method to control the overall familywise type I error rate at 0.05.	To incorporate multiple testing method in agreement with the FDA proposal.
The number of deaths required for the OS final analysis was revised to reflect the change in the alpha allocation of 0.05 in the original analysis plan to the new alpha allocation of 0.04. One additional IA of OS at approximately [redacted] of deaths was added.	Modification of the interim analysis of OS, 2 interim analyses and 1 final analysis were planned for OS.
The definition and the analysis plan of PFS2 was added.	Added second progression-free survival (PFS2) as an evaluation per agreement with health authorities.
**v2.0 (dated 27 February 2014) to v3.0 (dated 15 April 2015)**	
A new section of Analysis by Formulation was added to include additional analysis plans for subject distribution, demographic and baseline characteristics, study drug administration, safety, and the primary endpoint	The protocol was amended to switch softgel capsules to tablets (commercial formulation) for patients receiving the softgel capsules and to administer tablets to newly enrolled patients.
**v3.0 (dated 15 April 2015) to v4.0 (dated 15 March 2017)**	
Changed the multiple testing procedure for the secondary endpoints to a hierarchical testing procedure that also included a sample size re-estimation for the required number of events for time to symptomatic progression and overall survival; Details provided in Section 7.5.3 and Appendix 1.	The Bonferroni testing procedure as described in v3.0 of the SAP for secondary endpoints is conservative and lacked power, especially for some key secondary endpoints with low event rates, at the time of the primary analysis.
Simplified and removed some unnecessary	Deemed unnecessary in the presence of

sensitivity analyses: Removed multivariate analysis of MFS and the analysis of the impact of subsequent therapy on MFS; removed investigator derived PFS and TTM analysis	several other sensitivity analyses of MFS and OS already in the SAP.
Removed the by-formulation analysis using the arbitrary cutoff of 6 months (>6 vs <= 6 months of duration of treatment), added analysis by greater tablet duration or greater capsule duration	FDA feedback: Analysis by greater tablet duration or greater capsule duration would be more meaningful
Added Appendix 2, table shell for overall summary of AE by formulation	Added for clarity, as to what exactly will be summarized for AE by formulation.
**v4.0 (dated 15 March 2017) to v5.0 (dated 28 March 2017)**	
Minor revision in Section 7.5.3 and Appendix 1. Clarified that under the hierarchical testing procedure framework, "not tested" means that no statistical significance can be claimed if the endpoint above in the hierarchy is not found to be positive.	Based on FDA Type A meeting feedback and correspondences.
**v5.0 (dated 28 March 2017) to v6.0 (dated 22 June 2017)**	
Removed the definition of the PRO population	ITT population will be used for PRO analysis and hence no need to define a PRO population
Revised the criteria for markedly abnormal vital signs in Section 7.6.3	Clinical proposed new cutoff values based on CTCAE grade and change within grade
Added Appendix 3 “Modified MedDRA queries as search criteria for AE of special interest”	For clarity
**v6.0 (dated 22 June 2017) to v7.0 (dated 26 June 2017)**	
Minor changes in Appendix 1- clarification on the choice of weights in p-value combination methods.	Fixed weights to be used.
