# Supplementary material for: Prior Local Therapy and First-Line Apalutamide in Patients With Nonmetastatic Castration-Resistant Prostate Cancer: A Secondary Analysis of the SPARTAN Randomized Clinical Trial
Source: JAMA Netw Open. 2024 Oct 15;7(10):e2439434. doi: 10.1001/jamanetworkopen.2024.39434 (PMC11581638; doi:10.1001/jamanetworkopen.2024.39434)
Supplement: Supplement 2. — eFigure 1. CONSORT Diagram eTable 1. Baseline Characteristics by Exposure to Prior Radiation Therapy and Randomized Treatment Regimen eTable 2. Baseline Characteristics by Exposure to Prior Radical Prostatectomy and Randomized Treatment Regimen eResults eFigure 2. Love Plot Summarizing the Balance of Covariables Between the Unadjusted and Weighted Dataset for Prior Local Therapy eFigure 3. Histogram Summarizing the Distribution of Propensity Scores (Left Panel) and Weights (Right Panel) Among Patients With and Without Prior Prostate-Directed Local Therapy eFigure 4. Love Plot Summarizing the Balance of Covariables Between the Unadjusted and Weighted Dataset for Prior Local Therapy (LT) Modalities (Prior Radical Prostatectomy [RP], Prior Radiation Therapy [RT], or Prior RP Plus RT Compared to Prior LT) [file jamanetwopen-e2439434-s002.pdf]

Supplementary Online Content

Roy S, Malone S, Wing K, et al. Prior local therapy and first-line apalutamide in patients with nonmetastatic castration-resistant prostate cancer: a secondary analysis of the SPARTAN randomized clinical trial. *JAMA Netw Open*. 2024;7(10):e2439434. doi:10.1001/jamanetworkopen.2024.39434

eFigure 1. CONSORT Diagram

eTable 1. Baseline Characteristics by Exposure to Prior Radiation Therapy and Randomized Treatment Regimen

eTable 2. Baseline Characteristics by Exposure to Prior Radical Prostatectomy and Randomized Treatment Regimen

eResults

eFigure 2. Love Plot Summarizing the Balance of Covariables Between the Unadjusted and Weighted Dataset for Prior Local Therapy

eFigure 3. Histogram Summarizing the Distribution of Propensity Scores (Left Panel) and Weights (Right Panel) Among Patients With and Without Prior Prostate-Directed Local Therapy

eFigure 4. Love Plot Summarizing the Balance of Covariables Between the Unadjusted and Weighted Dataset for Prior Local Therapy (LT) Modalities (Prior Radical Prostatectomy [RP], Prior Radiation Therapy [RT], or Prior RP Plus RT Compared to Prior LT)

This supplementary material has been provided by the authors to give readers additional information about their work.

eFigure 1. CONSORT Diagram

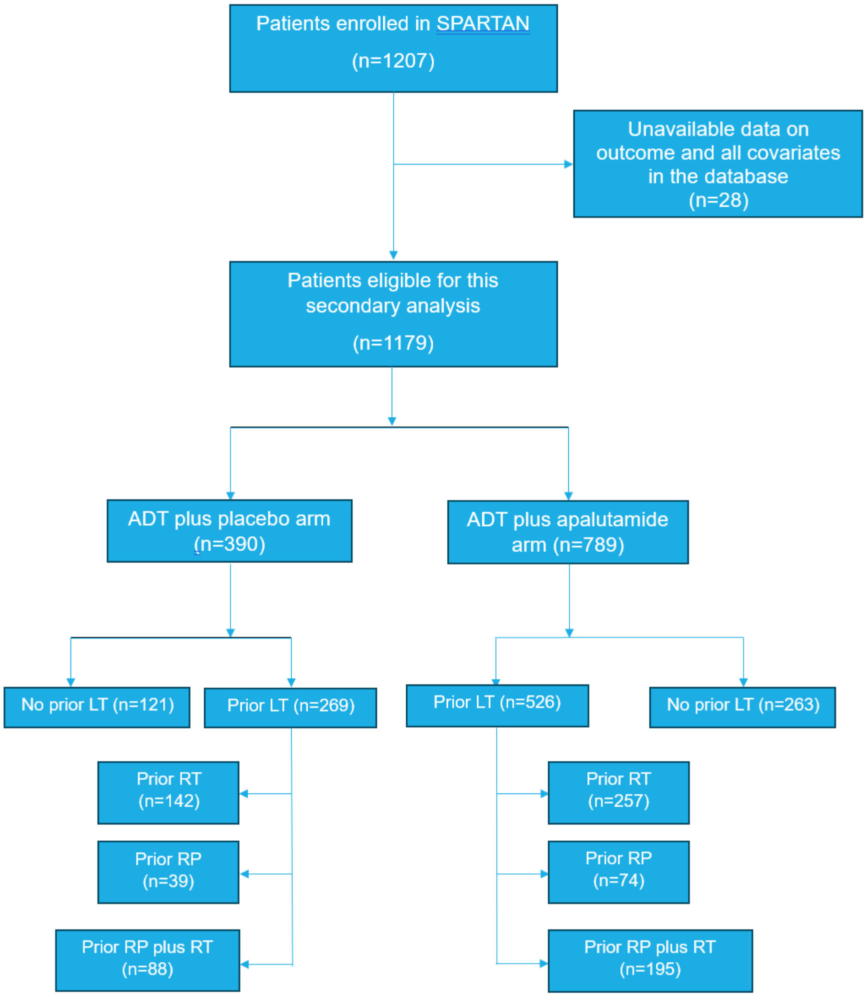

© 2024 Roy S et al. *JAMA Network Open.*

eTable 1. Baseline Characteristics by Exposure to Prior Radiation Therapy and Randomized Treatment Regimen

	No Prior Radiation Therapy		Prior Radiation Therapy	
	**Placebo**	**Apalutamide**	**Placebo**	**Apalutamide**
**N**	160	337	230	452
**Age at randomization (median [IQR])**	75 [69, 80]	75 [65, 80]	70 [65, 75]	70 [65, 75]
**PSA doubling time in months (median [IQR])**	4.5 [3.0, 6.6]	4.7 [3.2, 6.8]	4.5 [2.8, 6.2]	4.0 [2.7, 6.1]
**Time since diagnosis in years (median [IQR])**	6.0 [4.0, 9.0]	5.0 [3.0, 9.0]	9.0 [5.0, 12.0]	9.0 [6.0, 13.0]
**Race (%)**				
Black	9 (5.6)	14 (4.2)	9 (3.9)	32 (7.1)
Asian	29 (18.1)	59 (17.5)	18 (7.8)	32 (7.1)
White	99 (61.9)	212 (62.9)	170 (73.9)	302 (66.8)
Others*	23 (14.4)	52 (15.4)	33 (14.3)	86 (19.0)
**N1 nodal stage (%)**	29 (18.1)	65 (19.3)	35 (15.2)	67 (14.8)
**Use of bone sparing agents (%)**	13 (8.1)	29 (8.6)	26 (11.3)	50 (11.1)
**Prior RP exposure (%)**	39 (24.4)	74 (22.0)	88 (38.3)	195 (43.1)
**Primary Gleason score (%)**				
1-3	48 (30.0)	112 (33.2)	93 (40.4)	213 (47.1)
4-5	104 (65.0)	208 (61.7)	128 (55.7)	224 (49.6)
Unassigned	8 (5.0)	17 (5.0)	9 (3.9)	15 (3.3)
**Secondary Gleason score (%)**				
1-3	60 (37.5)	96 (28.5)	83 (36.1)	168 (37.2)
4-5	92 (57.5)	223 (66.2)	138 (60.0)	266 (58.8)
Unassigned	8 (5.0)	18 (5.3)	9 (3.9)	18 (4.0)
**Tumor stage at diagnosis (%)**				
T1-T2	77 (48.1)	140 (41.5)	108 (47.0)	265 (58.6)
T3-T4	67 (41.9)	161 (47.8)	110 (47.8)	166 (36.7)
TX	16 (10.0)	36 (10.7)	12 (5.2)	21 (4.6)
**Use of prior ADT (%)**	106 (66.2)	234 (69.4)	163 (70.9)	345 (76.3)

*Others included American Indian or Alaskan Native, unreported, or others, respectively.

eTable 2. Baseline Characteristics by Exposure to Prior Radical Prostatectomy and Randomized Treatment Regimen

	No Prior RP		Prior RP	
	**Placebo**	**Apalutamide**	**Placebo**	**Apalutamide**
**N**	263	520	127	269
**Age (median [IQR])**	75.0 [65.0, 80.0]	75.0 [65.0, 80.0]	70.0 [65.0, 75.0]	70.0 [65.0, 75.0]
**PSA doubling time in months (median [IQR])**	4.6 [3.0, 6.5]	4.6 [3.0, 6.7]	4.1 [2.6, 6.1]	4.0 [2.6, 5.9]
**Time since diagnosis in years (median [IQR])**	7.0 [4.0, 10.0]	7.0 [4.0, 10.2]	9.0 [6.0, 13.0]	10.0 [6.0, 15.0]
**Race (%)**				
Black	11 (4.2)	28 (5.4)	7 (5.5)	18 (6.7)
Asian	29 (11.0)	73 (14.0)	18 (14.2)	18 (6.7)
White	184 (70.0)	329 (63.3)	85 (66.9)	185 (68.8)
Others*	39 (14.8)	90 (17.3)	17 (13.4)	48 (17.8)
**N1 nodal stage (%)**	37 (14.1)	83 (16.0)	27 (21.3)	49 (18.2)
**Bone sparing agent use (%)**	26 (9.9)	52 (10.0)	13 (10.2)	27 (10.0)
**Prior radiation therapy exposure (%)**	142 (54.0)	257 (49.4)	88 (69.3)	195 (72.5)
**Primary Gleason score (%)**				
1-3	97 (36.9)	199 (38.3)	44 (34.6)	126 (46.8)
4-5	153 (58.2)	300 (57.7)	79 (62.2)	132 (49.1)
Unassigned	13 (4.9)	21 (4.0)	4 (3.1)	11 (4.1)
**Secondary Gleason Score (%)**				
1-3	87 (33.1)	176 (33.8)	56 (44.1)	88 (32.7)
4-5	163 (62.0)	320 (61.5)	67 (52.8)	169 (62.8)
Unassigned	13 (4.9)	24 (4.6)	4 (3.1)	12 (4.5)
**Tumor stage at diagnosis (%)**				
T1-T2	133 (50.6)	274 (52.7)	52 (40.9)	131 (48.7)
T3-T4	109 (41.4)	203 (39.0)	68 (53.5)	124 (46.1)
TX	21 (8.0)	43 (8.3)	7 (5.5)	14 (5.2)
**Prior ADT use (%)**	176 (66.9)	384 (73.8)	93 (73.2)	195 (72.5)

*Others included American Indian or Alaskan Native, unreported, or others, respectively.

eResults

List of minimally sufficient confounders:

Prior local therapy and MFS

Minimally sufficient list of confounders included time since cancer diagnosis, PSA doubling time, and primary Gleason score, respectively.

Prior radiation therapy and MFS

Minimally sufficient list of confounders included PSA doubling time, time since diagnosis, primary Gleason score, and prior RP, respectively.

Prior radical prostatectomy and MFS

Minimally sufficient list of confounders included PSA doubling time, time interval since cancer diagnosis, and exposure to prior RT, respectively.

Prior local therapy and OS:

Minimally sufficient set of confounders included PSA doubling time and age at random assignment.

Prior radiation therapy and OS:

Minimally sufficient set of confounders included PSA doubling time and age at random assignment.

Prior radical prostatectomy and OS:

Minimally sufficient set of confounders included PSA doubling time and age at random assignment.

© 2024 Roy S et al. *JAMA Network Open.*

Results from the Sensitivity Analyses:

As sensitivity analyses, we tested causal interaction for each pair of potential effect modifier and time-to-event outcome where we included all available variables from the trial database as confounders.

Fully adjusted model for MFS to evaluate interaction of randomized treatment regimen with prior LT, prior RP, and prior RT, respectively.

For prior LT:

There was a significant difference (p-interaction=0.007) in treatment effect from ADT plus apalutamide among patients stratified by exposure to prior LT with 79% reduction in the risk of distant metastasis or death in those with prior LT (adjusted HR: 0.21; 95% CI: 0.17-0.26) and 67% reduction in the risk of distant metastasis or death in those without prior LT (adjusted HR: 0.33; 95% CI: 0.23-0.48), respectively.

For prior RT:

Treatment with ADT plus apalutamide was associated with significant improvement in MFS among patients with (adjusted HR: 0.21; 95% CI: 0.16-0.27) and without prior RT (adjusted HR: 0.28; 95% CI: 0.20-0.39) with no statistically significant difference in the treatment effect across the subgroups (p-interaction=0.07).

For prior RP:

The findings from the fully adjusted analysis were consistent with the partially adjusted analysis with a significantly different (p-interaction=0.003) treatment effect from ADT plus apalutamide on MFS among patients stratified by exposure to prior RP. ADT plus apalutamide was associated with 87% reduction in the hazard for distant metastasis or deaths among patients with prior RP (HR: 0.13; 95% CI: 0.09-0.19) compared to 71%

© 2024 Roy S et al. *JAMA Network Open.*

reduction in the hazard for distant metastasis or deaths among patients with no prior RP (HR: 0.29; 95% CI: 0.23-0.37).

Fully adjusted model for OS to evaluate interaction of randomized treatment regimen with prior LT, prior RP, and prior RT, respectively.

For prior LT:

The findings from the fully adjusted model were congruent with the partially adjusted model. There was no heterogeneity in the treatment effect among patients with or without prior LT (p-interaction=0.22) based on a fully adjusted multivariable model. Treatment with ADT plus apalutamide was associated with improved OS in both subgroups – i.e., patients with (adjusted HR: 0.70; 95% CI: 0.54-0.89) and without prior LT (adjusted HR: 0.90; 95% CI: 0.63-1.29), respectively.

For prior RT:

The findings of the fully adjusted model were consistent with the partially adjusted model with no significant evidence of differential treatment effect from ADT plus apalutamide on OS across subgroups stratified by exposure to prior RT (p=0.62). Treatment with apalutamide was associated with 25% reduction in the hazard for death (adjusted HR: 0.75; 95% CI: 0.57-0.98) among patients with prior RT and 15% reduction in the hazard for deaths among those without prior RT (adjusted HR: 0.85; 95% CI: 0.62-1.17).

For prior RP:

The findings from the fully adjusted models were consistent with that from partially adjusted model with no significant evidence of differential treatment effect from ADT

© 2024 Roy S et al. *JAMA Network Open.*

plus apalutamide (p=0.11) across subgroups stratified by prior RP. Treatment with ADT plus apalutamide was associated with superior OS among patients with (adjusted HR: 0.53; 95% CI: 0.35-0.78) and without (adjusted HR: 0.83; 95% CI: 0.65-1.06) prior RP.

© 2024 Roy S et al. *JAMA Network Open.*

eFigure 2. Love Plot Summarizing the Balance of Covariables Between the Unadjusted and Weighted Dataset for Prior Local Therapy

The dashed vertical line represents the absolute SMD of 0.1.

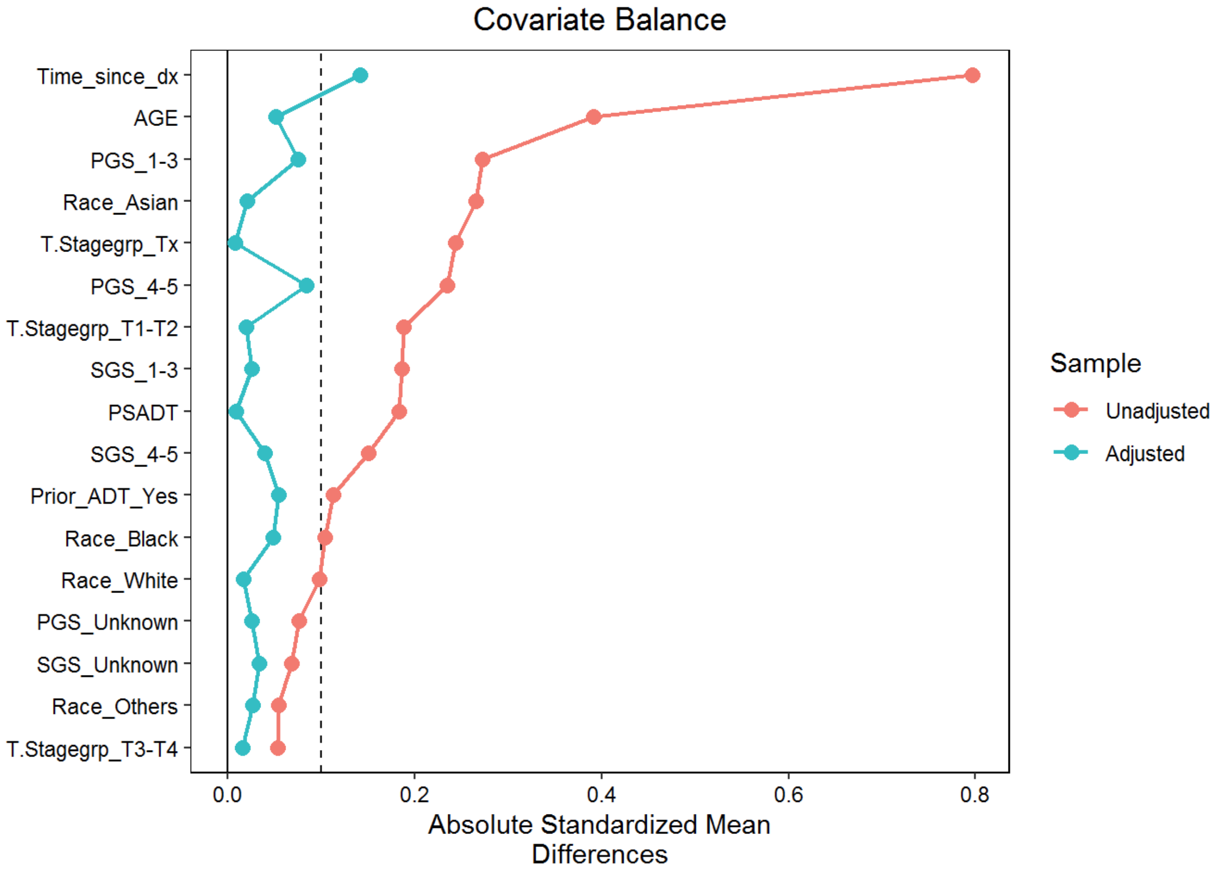

[Time_since_dx: Time since diagnosis; PGS: Primary Gleason score; SGS: Secondary Gleason score; T.Stagegrp: Tumor stage at diagnosis; PSADT: Prostate-specific antigen doubling time]

© 2024 Roy S et al. *JAMA Network Open.*

eFigure 3. Histogram Summarizing the Distribution of Propensity Scores (Left Panel) and Weights (Right Panel) Among Patients With and Without Prior Prostate-Directed Local Therapy

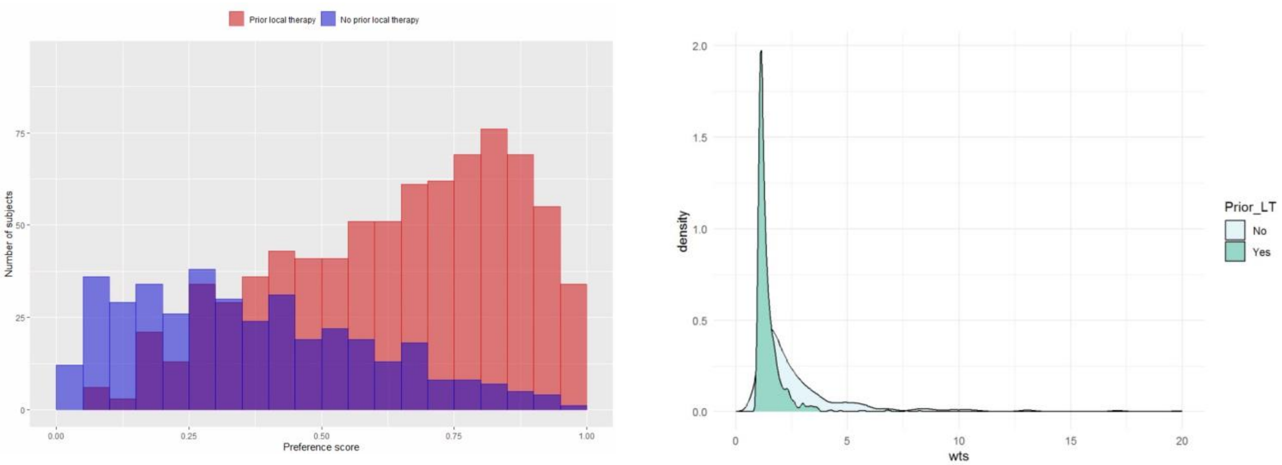

© 2024 Roy S et al. *JAMA Network Open.*

eFigure 4. Love Plot Summarizing the Balance of Covariables Between the Unadjusted and Weighted Dataset for Prior Local Therapy (LT) Modalities (Prior Radical Prostatectomy [RP], Prior Radiation Therapy [RT], or Prior RP Plus RT Compared to Prior LT)

The dashed vertical line represents the absolute SMD of 0.1.

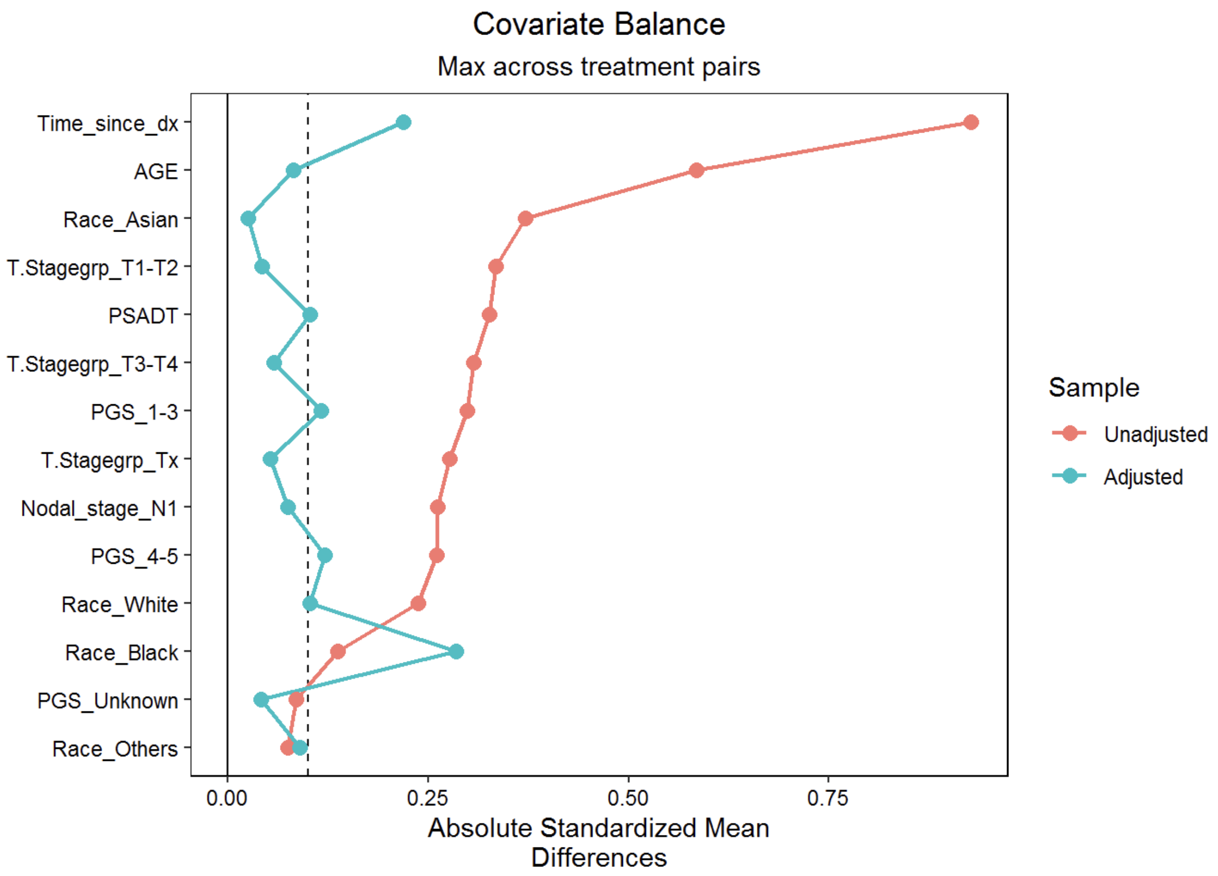

[Time_since_dx: Time since diagnosis; PGS: Primary Gleason score; SGS: Secondary Gleason score; T.Stagegrp: Tumor stage at diagnosis; PSADT: Prostate-specific antigen doubling time]

© 2024 Roy S et al. *JAMA Network Open.*
