# Supplementary material for: Prior Local Therapy and First-Line Apalutamide in Patients With Nonmetastatic Castration-Resistant Prostate Cancer: A Secondary Analysis of the SPARTAN Randomized Clinical Trial
Source: JAMA Netw Open. 2024 Oct 15;7(10):e2439434. doi: 10.1001/jamanetworkopen.2024.39434 (PMC11581638; doi:10.1001/jamanetworkopen.2024.39434)
Supplement: Supplement 3. — Data Sharing Statement [file jamanetwopen-e2439434-s003.pdf]

Data Sharing Statement

Roy. Prior Local Therapy and First-Line Apalutamide in Patients With Nonmetastatic Castration-Resistant Prostate Cancer. *JAMA Netw Open*. Published October 15, 2024. doi:10.1001/jamanetworkopen.2024.39434

Data
Additional Information: ClinicalTrials.gov number, NCT01946204
Data available: No

Additional Information
Explanation for why data not available: This study was carried out under Yale Open Data Access (YODA) Project # 2023-5137 and used data obtained from the Yale University Open Data Access Project, which has an agreement with Janssen Research & Development, L.L.C, and the primary sponsor of the SPARTAN trial. Thus, the analysis is based on provided data by YODA and Janssen under the project 2023-5137 and the data would not be shared by the authors. The interpretation and reporting of research using this data are solely the responsibility of the authors and do not necessarily represent the official views of the Yale University Open Data Access Project or JANSSEN RESEARCH & DEVELOPMENT, L.L.C.
